# Surfactant Lipidomics in Healthy Children and Childhood Interstitial Lung Disease

**DOI:** 10.1371/journal.pone.0117985

**Published:** 2015-02-18

**Authors:** Matthias Griese, Hannah G. Kirmeier, Gerhard Liebisch, Daniela Rauch, Ferdinand Stückler, Gerd Schmitz, Ralf Zarbock, ILD-BAL working group of the Kids-Lung-Register

**Affiliations:** 1 Department of Pediatric Pulmonology, Hauner Children’s Hospital, Ludwig Maximilians University, Member of the German Center for Lung Research (DZL), Lindwurmstr. 4a, D-80337 Munich, Germany; 2 Institute of Computational Biology, Helmholtz Center Munich, Neuherberg, Germany; 3 Institute of Clinical Chemistry and Laboratory Medicine, University of Regensburg, Franz-Josef-Strauss-Allee 11, 93053 Regensburg, Germany; The Hospital for Sick Children and The University of Toronto, CANADA

## Abstract

**Background:**

Lipids account for the majority of pulmonary surfactant, which is essential for normal breathing. We asked if interstitial lung diseases (ILD) in children may disrupt alveolar surfactant and give clues for disease categorization.

**Methods:**

Comprehensive lipidomics profiles of broncho-alveolar lavage fluid were generated in 115 children by electrospray ionization tandem mass spectrometry (ESI-MS/MS). Two reference populations were compared to a broad range of children with ILD.

**Results:**

Class and species composition in healthy children did not differ from that in children with ILD related to diffuse developmental disorders, chronic tachypnoe of infancy, ILD related to lung vessels and the heart, and ILD related to reactive lymphoid lesions. As groups, ILDs related to the alveolar surfactant region, ILD related to unclear respiratory distress syndrome in the mature neonate, or in part ILD related to growth abnormalities reflecting deficient alveolarisation, had significant alterations of some surfactant specific phospholipids. Additionally, lipids derived from inflammatory processes were identified and differentiated. In children with ABCA3-deficiency from two ILD causing mutations saturated and monounsaturated phosphatidylcholine species with 30 and 32 carbons and almost all phosphatidylglycerol species were severely reduced. In other alveolar disorders lipidomic profiles may be of less diagnostic value, but nevertheless may substantiate lack of significant involvement of mechanisms related to surfactant lipid metabolism.

**Conclusions:**

Lipidomic profiling may identify specific forms of ILD in children with surfactant alterations and characterized the molecular species pattern likely to be transported by ABCA3 in vivo.

## Background

In the lungs, the alveolar space forms a vital and metabolically highly active compartment responsible for gas-exchange and host defense [1]. A thin layer of a surface-active material called surfactant covers the epithelium towards the air spaces, where its role is to lower the surface tension and prevent end-expiratory collapse of the alveoli. Pulmonary surfactant is a complex mixture of lipids (about 80–90%) and surfactant specific proteins [2]. The composition and function can variably be affected by many respiratory diseases [3]. A precise characterization of the lipids of this compartment, which are accessible by broncho-alveolar lavages, may yield clues for the diagnostic categorizing of pulmonary diseases affecting the alveolar space, for insights into the metabolism of its numerous components, and for therapeutic interventions to correct disease induced alterations.

Up to now neither in healthy children nor in the large group of human diseases affecting the alveolar surfactant compartment, comprehensive lipidomic analysis with state of the art technology has been performed. Lipids and phospholipid classes have been investigated in a number of studies in healthy subjects und many lung diseases [3]. Analysis of the species composition of these lipids has been achieved for a very limited number of conditions and phospholipid classes. These include phosphatidylcholine species in the bronchoalveolar lavage fluid from control children and children with acute lung injury [4], children with asthma [5], infection and cystic fibrosis [6,7], premature infants with respiratory distress syndrome who went on to develop chronic lung disease [8–10] and in adults with Pneumocystis pneumonia and acquired immunodeficiency syndrome [11]. Species composition of phosphatiylglycerol and phosphatidylinositol were reported in some of the diseases. Most closely extensive lipidomics of cellular and secreted phospholipids has been analyzed in isolated and differentiated human fetal type II alveolar epithelial cells [12].

Chronic childhood interstitial lung diseases (chILD) represent a large spectrum of mostly rare diffuse parenchymatous diseases, prevalent in children of all ages [13,14]. Many of these diseases directly affect varying components the pulmonary surfactant system, including genetically caused deficiency of the lipid transporter ABCA3 [15], surfactant protein (SP) SP-C and SP-B deficiency [16], and deficiency of the transcription factor TTF1 [17,18]. There exist many more diseases with the typical clinical presentation of chILD, however without characterization as yet. In addition, all the other forms of ILD might have an impact on the pulmonary surfactant lipid system. We hypothesized that several of these conditions are affected and that lipidomic analysis may help to identify such entities. By this candidates might be identified for genetic abnormalities leading to disturbances of the pulmonary surfactant system. In addition we expect that grouping of diseases into clinical subcategories might also generate relatively homogenous and distinct lipid profiles which may significantly differ between groups, supporting the value of the classification system.

To address these hypothesis experimentally, a cohort of more than 100 children suffering from diffuse parenchymatous diseases, which had been diagnosed clinically to varying extent at the discretion of the attending physician and for the most part according to the principles of the recent ATS state of the art proposal [19], was grouped into an extended classification system, based on the one originally proposed by Deutsch et al and Langston et al. [13,20]. Comprehensive lipidomic analysis of their lavage lipids, including the alveolar surfactant system was performed and compared to that of a healthy control group. In addition a second non-chILD comparison group, i.e. children with bronchitis, was used.

## Methods

### Patient selection

Between 1997 to 2007 all cases sent to our lab for biochemical and surfactant analysis were carefully diagnosed to have diffuse parenchymal lung disease, i.e. interstitial lung disease, by history, findings, course and sometimes histological and genetic means, as part of the routine work up of these patients. Of these, 111 had chronic (> 4 weeks) diffuse parenchymal lung disease affecting both sides of the lungs by radiology, gave informed consent and had lavage material available for analysis. The children were further categorized diagnostically into one of the ILD categories listed in Table 1 and a definite clinical diagnosis was obtained at the highest level possible in each case. These patients were compared to 16 healthy controls (controls-healthy) and 16 disease controls, i.e. children assessed for chronic cough and exclusion of cystic fibrosis, structural abnormalities, immune deficiencies, allergic airway disease and primary ciliary dyskinesia (controls-bronchitis). The individual diagnoses and lipidomic results are listed in S1 Table. Patients 264, 644, 1180 [21], 641 [22], 90 [23], 497 [24] were included in the previous manuscripts indicated. More details are indicated in S1 Methods.

**Table 1:** Subjects included into the study, their allocation to pulmonary disease categories and subcategories, subjects excluded and final number of subjects.

Group No	Disease category	Subcategories	Mean age at start of lung disease (y)	Mean age at last follow up (y)	Number of subjects	Exclusion due to low lipid concentration /insufficient clinical information	Final number of subjects
1	Controls—healthy	Healthy	n. a.	7.1	16	5	11
2	Controls—Bronchitis	Normal alveolar cell count and differential in BAL	n. a.	4.7	16	6	10
3	Diffuse development disorders	Alveolar capillary dysplasia with misalignment pulmonary veins	0.0	0.1	2		2
4	Growth abnormalities reflecting deficient alveolarisation	Intrauterine growth retardation (due to alcohol)(1), Pulmonary hypoplasia (5), Related to chromosomal disorders (4), Related to preterm birth (BPD-cLDI, Mikity-Wilson-Syndrome)(13)	0.1	2.2	23	5	18
5	Immuno-intact host	Eosinophilic alveolitis (2), Exogen allergic alveo1itis/Hypersensitivity pneumonitis (5)	8.3	10.9	7	2	5
6	Immuno-compromised host	Infections—Antibody deficiency (2), Phagocyte defects (1), T-cell deficiency (2), Miscellaneous (4)	3.5	5.8	9	1	8
7	Chronic tachypnoe of infancy	No further differentiation	0.4	3.6	10	1	9
8	Reactive lymphoid lesions	Nodular lymphoid hyperplasia of the lung (2), Follicular bronchitis/bronchiolitis (1), Lymphocytic interstitial pneumonia (LIP)(1)	3.3	8.9	4		4
9	Related to alveolar surfactant region	ABCA 3 (1 mutation)(5), ABCA 3 (2 mutations)(6), Chronic pneumonitis of infancy (CPI)(1), Desquamative interstitial pneumonitis (DIP)(1), Lipoidpneumonitis/Cholesterol pneumonia (2), Nkx 21 gene defect (1), Nonspecific interstitial pneumonia (NSIP)(1), NSIP + pulmonary alveolar proteinosis (PAP) + Microvasculopathy (1), Surfactant protein B mutation (3), Surfactant protein C mutation (3)	1.0	5.5	24	3	21
10	Related to lung vessels /heart	Congestive changes related to cardiac dysfunction (2), Lymphatic disorders (1), M. Osler (1), Pulmonary capillary hemangiomatosis (1), Pulmonary hypertension (2)	2.8	7.8	7		7
11	Related to systemic diseases	Alagille-Syndrome (arterohepatic dysplasia) (1), Familial dysautonomia (Chr. 9q31 encoding ICAP)(1), Hoyeral-Hreidasson-Syndrome (Dyskeratosis congenita)(1), Idiopathic pulmonary hemosiderosis (2), Immune mediated/Collagen vascular disorders (4), Sarcoidosis (1)	5.5	9.1	10	3	7
12	Unclear RDS in the mature neonate	No/low SP-C biochemically (3), No/low SP-B biochemically (4), Familial (1), Pulmonary hypertension (2), No further classification (5)	0.0	1.0	15	2	13
All					143	28	115

### Broncho-alveolar lavages, sample preparation and biochemical analysis

Broncho-alveolar lavage supernatant was extracted in the presence of not naturally occurring lipid species as internal standards and crude lipid extracts were quantified by direct flow injection electrospray ionization tandem mass spectrometry (ESI-MS/MS) in positive ion mode [25,26] and more detailed in the supplement. Lipid species were annotated according to the recently published proposal for shorthand notation of lipid structures [27].

### Calculation and expression of lipid results

**Fig 1:**
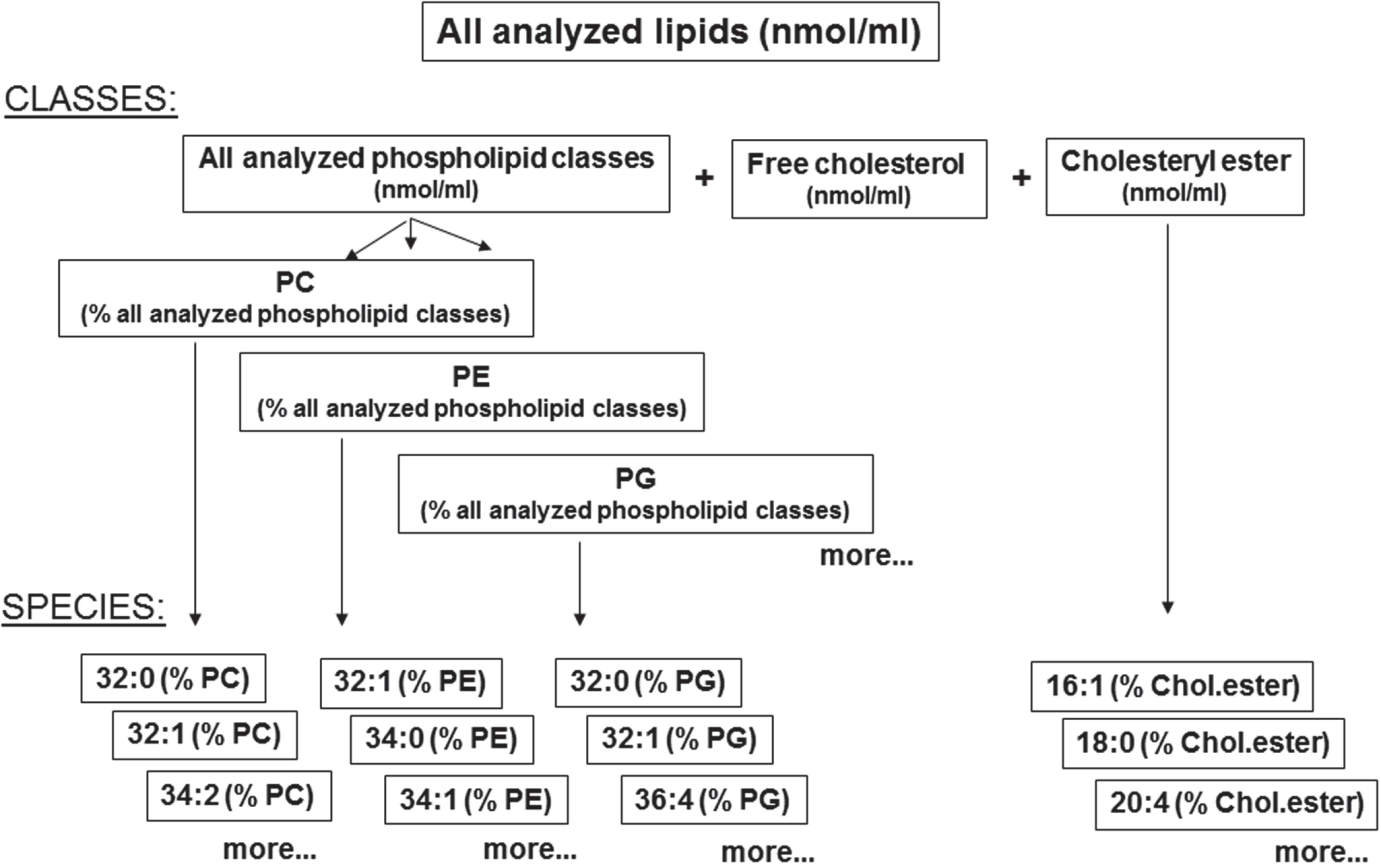
Calculation and expression of the results of the lipid analysis. All analyzed lipids are the sum of all analyzed phospholipid classes, cholesteryl ester and free cholesterol (all expressed as nmol/ml). Phospholipid classes are expressed as % of all analyzed phospholipids and the species as percentage of that phospholipid class. In the body of the manuscript mainly the results of all lipid classes and the composition of one of its classes, i.e. phosphatidylcholine, are indicated. Composition of the other classes are presented in the supplemental materials. For overview see also panel A in S1 Fig.

Results are presented as total lipids, total phospholipids, cholesteryl ester and free cholesterol (all expressed as nmol/ml) (Fig. 1, panel A in S1 Fig.). Species were only taken into consideration for presentation if the species had an abundance of ≥ 0.5%. To guarantee quality of analysis and to eliminate a sample factor, diluted bronchoalveolar lavages with a total lipid concentration < 15 μmol/l or a phospholipid concentration < 10 μmol/l were excluded from calculations, as most lipid species are below or close to the limit of detection. In total 28 of the 143 subjects were excluded from the final analysis, as detailed in Table 1.

### Ethics Statement

The study was approved by the institutional review board, the Ethikkommission der Med. Fakultät der LMU München, Pettenkoferstr. 8, 80336 München (EK 026–06) and all parents or guardians gave their written informed consent, and the children gave assent. Samples from the controls-healthy were collected during a previous study [28].

### Statistical analysis

For quality control and multivariate, unsupervised data analysis we performed a principle component analysis (PCA) using the NIPALS method from the pcaMethod package in the R 3.1.1 statistical software (http://www.r-project.org) [29]. The analysis was based on the concentration on log scale of 143 lipid subspecies for 115 patients. No significant batch effects were identified (panel B in S1 Fig.), additionally this was confirmed by calculating PCA using absolute and relative lipid data (not shown). Group comparisons were primarily made between healthy controls and the other groups (A) and B) in panel A in S1 Fig.). As the data were mostly but not always normally distributed non-parametric Kruskal-Wallis ANOVA and Dunn’s post hoc tests were used; groups with 3 or less subjects were excluded from the analysis (Prism 4 software (San Diego, CA, U.S.A). We accounted for multiple testing by false discovery rate (FDR) correction for p-values from 153 ANOVA tests (for lipid classes and species). For FDR correction at a global significance level of 0.05, the new significance cut-off was 0.0182. Data in the tables are expressed as means and standard deviations, in the supplemental material additional descriptors are given in tables in S3 Table.

Lastly, the results were displayed on the level of molecularly or clinically defined diseases (C) in panel A in S1 Fig.). Here no statistical comparisons were made except for phosphatidylcholine and phosphatidylglycerol in patients with two ABCA3 mutations (S2 Table). FDR was used for correction of the multiple lipid classes and species compared.

## Results

### A) Lavage lipidomics in healthy children

**Fig 2:**
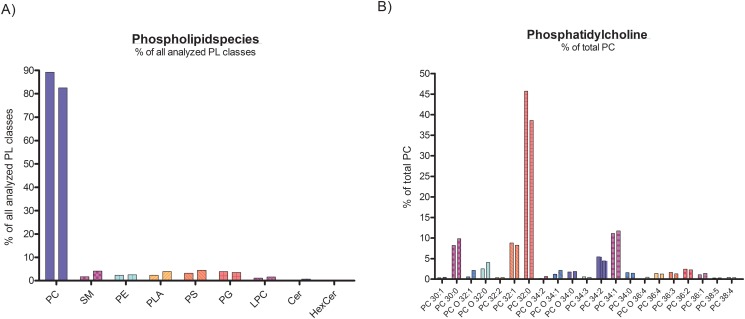
A) Phospholipid classes of the control groups (healthy (n = 11), left column of pair; bronchitis (n = 10), right column of pair). Lipid class composition is expressed as % of the analyzed displayed lipid classes (left graph). B) Phosphatidylcholine species composition is expressed as % phosphatidylcholine (right graph). The species of the other phospholipid classes are displayed in the S2 Fig. Data are means. Species present at an abundance of < 0.5% were not displayed.

**Fig 3:**
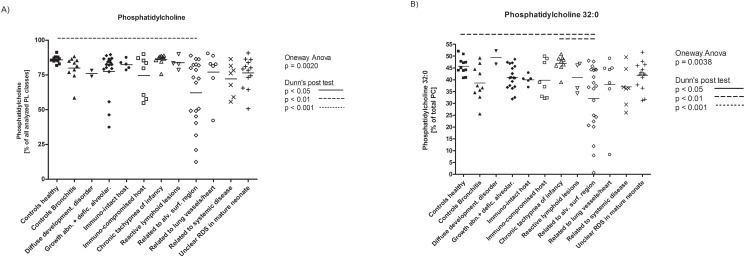
Display of results at the disease category level. A) Phosphatidylcholine, the major surfactant phospholipid class and B) its species dipalmitoylphosphatidylcholine (PC 32:0) are given as individual results of all patients included in the study according the disease category they belong to. The statistical comparisons were done by ANOVA and Dunn’s post hoc test; all significant results are displayed in each figure. To take multiple comparisons into consideration, an overall cut-off level of P<0.0182 was calculated. For A) 1-way ANOVA gave a P = 0.0020, for B) P < 0.0038; the significant results of Dunn’s post hoc tests are indicated as following: ____ = P < 0.05, _ _ _ = P < 0.01, and …. = P < 0.001. The species of the other phosphatidylcholines, the other phospholipid classes and their species composition are displayed in S3 Fig. Horizontal bar indicates median.

For the first time extensive BAL lipidomics profile of children with a normal alveolar surfactant, i.e. healthy children and those with bronchitis and as such with no or very little impact on the alveolar space were determined comprehensively. We analyzed lavage for many of its lipids (Fig. 1) and found that total phospholipids constituted more than 85%; free cholesterol was about 10% and a small amount were cholesteryl esters (Table 2). Among the phospholipid species, phosphatidylcholine was by far the most abundant, as expected for pulmonary surfactant (Fig. 2A), with dipalmitoyl-phosphatiylcholine (32:0) as the main species (Fig. 2B). When compared to the healthy children, the children with bronchitis had a similar pattern with no differences in classes; however there were some difference in minor species of the non-surfactant lipid classes phosphatidylethanolamine, phosphatidylserine, plasmalogens and cholesteryl esters. This underscores the susceptibility of lipid species composition to influences from bronchial inflammatory processes (S2 Fig.), even if only few and not all cell lipids typical for cells are altered. To readily detect the extent, single values are displayed (Fig. 3, S3 Fig.).

**Table 2:** Concentrations of all lipids classes, phospholipids, cholesteryl ester and free cholesterol in all disease categories investigated.

	Controls healthy	Controls Bronchitis	Diffuse developmental disorder	Growth abnormalities refl. def. alveolar.	Immuno- intact host	Immunocompromised host	Chronic tachypnea of infancy	Reactive lymphoid lesions	Related to alveolar surfactant region	Related to lung vessels/heart	Related to systemic disease	Unclear RDS in mature neonate	ANOVA P
Disease category	1	2	3	4	5	6	7	8	9	10	11	12	
Number of subjects	11	10	2	18 (18)	5	8	9 (9)	4	21 (21)	7	7	13	
All Lipid Classes	74 ± 128	167 ± 219	62 ± 63	246 ± 450	44 ± 26	96 ± 73	158 ± 1145	46 ± 42	331 ± 1123	332 ± 578	78 ± 51	103 ± 103	0.2451
Phospholipids	65 ± 117	128 ± 180	47 ± 47	212 ± 382	34 ± 23	75 ± 61	141 ± 136	38 ± 36	196 ± 642	291 ± 539	55 ± 37	84 ± 89	0.1760
Cholesteryl Ester	1 ± 1	11 ± 24	2 ± 1	4 ± 7	2 ± 1	3 ± 1	2 ± 1	2 ± 2	69 ± 269	11 ± 23	6 ± 4	3 ± 3	0.0270
Free Cholesterol	7 ± 10	28 ± 34	14 ± 14	30 ± 64	7 ± 4	18 ± 14	16 ± 20	6 ± 5	69 ± 227	31 ± 40	18 ± 16	16 ± 14	0.1927

Data are mean ± standard deviation and expressed as [nmol/ml]. ANOVA was used to detect differences between the various groups; if P < 0.05, Dunn’s post-hoc analysis was done. No significant differences were found at the corrected P value of 0.0182.

### B) Covering the whole clinical spectrum of childhood interstitial lung disease

In our large disease cohort we present lavage lipidomics data of all disease categories created so far in the currently used classification system of chILD [13,30]; these include in particular disease groups predominantly prevalent in infancy, like diffuse development disorders, growth abnormalities reflecting deficient alveolarisation, chronic tachypnoe of infancy, and disorders related to alveolar surfactant region. We introduced the category “children with unclear RDS (respiratory distress syndrome) in the mature neonate” for cases where no histologic or genetic investigations were done (Table 1, S1 Table for individual values and outcomes). By this we were able to include these patients for lipidomic analysis and we could test the hypothesis if their lipid profile would give a clue to their disease category.

**Total lipids, total phospholipids, free cholesterol and cholesteryl esters** classes were not statistically different between different disease categories (Table 2).

Differences of **phospholipid classes** were only identified in ILDs related to the alveolar surfactant region and ILD related to unclear RDS in the mature neonate. Phosphatidylcholine, the major surfactant phospholipid, was low in ILDs related to the alveolar surfactant region, and phosphatidylglycerol, the other major surfactant phospholipid was low in this disease group and in unclear RDS in the mature neonate (Table 3, second column; Fig. 3 and S3 Fig.).

**Table 3:** Changes of phospholipid classes and species in different patient groups in comparison to the controls-healthy.

No	Group to which Controls-healthy were compared:	Lipid class	Sphingomyeline	Phosphatidylcholine	Phosphatidylethanolamine	Plasmalogen	Phosphatidylserine	Phosphatidylglycerol	Lysophosphatidylcholine	Ceramide	Cholesteryl ester
2	Controls—Bronchitis					16:0/18:1, high					
3	Diffuse development disorders										
4	Growth abnormalities reflecting deficient alveolarisation		38:2, low;38:1, high	32:2, high;; O-34:0 low	40: 4, high	18:0/20:4, low	38:3, high	35:1, low;36:1, low			
5	Immuno-intact host					16:0/22:5, high					
6	Immuno-compromised host		42:2, high			16:0/20:4, high16:0/22:5, high	38:3, high;40:5, high				
7	Chronic tachypnoe of infancy										
8	Reactive lymphoid lesions										
9	Related to alveolar surfactant region	PC, low;SM, high;PS, high;PG, low;Cer, high;HexCer, high	38:1, high	32:0, low;O-34:1, high;O-34:2, high	36: 2, low; 36:3, low;40: 4, high		36:2, low;40:6, high;40:4, high	34:1, low;35:1, low;36:1, low;38:4, low			
10	Related to lung vessels /heart										
11	Related to systemic diseases			O-32:1, high;32:2, high;O-34: 2, high;O-36:4, high;	40:4, high	16:0/22:5, high	38:3, high40:4, high 40:5, high				20:0, low;22:4, low
12	Unclear RDS in the mature neonate	PG, low;Cer, high;HexCer, high		O-34:0, low	38:3, high;40:6, high;40:4, high	16:0/18:1, high;16:0/22:6, high;18:0/18:2, low	40:4, high 40:6, high	35:1, low;36:1, low		22:0, low	16:0, high;18:1, high

All indicated changes were significant by ANOVA/ Dunn’s post hoc test, after correction for multiple comparisons.

On the **species level**, in ILD related to alveolar surfactant region, dipalmitoyl-phosphatidylcholine (PC 32:0), the most abundant phosphatidylcholine species, was low. Characteristic changes were demonstrated for phosphatidylglycerol species 35:1 and 36:1, which were low in addition to the aforementioned disease groups, in ILD related to Growth abnormalities reflecting deficient alveolarisation. A separation mainly of ILD related to the alveolar surfactant region, but also cases of ILD related to unclear RDS in the mature neonate and ILD related to Growth abnormalities reflecting deficient alveolarisation from the other samples was observed during unsupervised data analysis using principal component analysis (panel B in S1 Fig.).

In these three disease groups with deviations of surfactant characteristic lipids, specific alterations of species with long fatty acid chains of sphingomyeline, phosphatidylethanolamine, plasmalogens, phosphatidylserine and cholesteryl esters were noted to various degrees, as were mainly increases of ether phosphatidylcholines, abundant in white blood cells. Such changes were also observed in ILD related to systemic diseases.

Apart from only very mild degree as in Controls-Bronchitis, changes of the plasmalogens were identified in ILD in the Immuno-intact or Immuno-compromised host (Table 3, S3 Fig.) no changes of the lipid profile were demonstrated in the four ILD groups: ILD related to Chronic tachypnoe of infancy, ILD related to reactive lymphoid lesions, ILD related to lung vessels/heart and ILD related to diffuse developmental disorders.

Taken together these findings demonstrate that on the level of ILD-disease categories alterations of the lipid profile related to pulmonary surfactant are present only in few disease categories, which are also associated with changes in non-surfactant lipids.

### C) Lavage lipidomics of molecularly and clinically defined diseases

**Fig 4:**
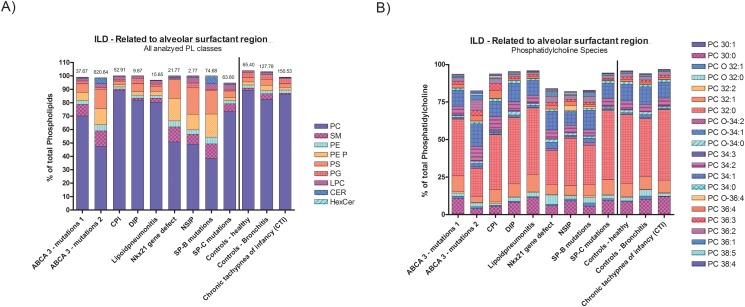
Display of results at the individual diagnosis level for the disease category “ILD-related to the alveolar surfactant region”. Lipid class composition (graph A) on left side) and phosphatidylcholine species composition (graph B) on right side) are indicated as means for each diagnosis. The number of subjects is detailed in Table 1. The numbers above the columns indicate the mean phospholipid concentrations (μmol/l). No statistical comparisons were done; the graphical display should allow a rapid identification of deviations from the controls. The other species are provided in S4 Fig. Only lipid species present at an abundance of > 0.5% are displayed.

**Fig 5:**
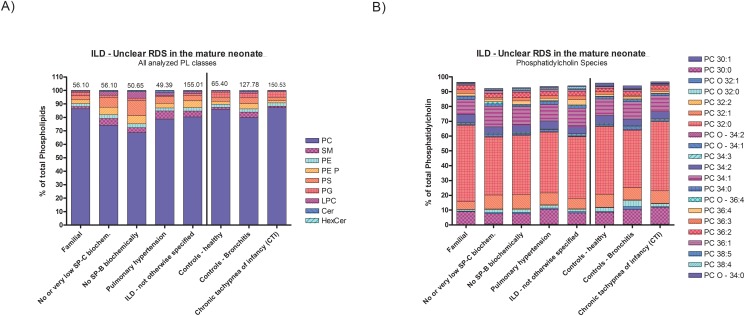
Display of results at the individual diagnosis level for the disease category “ILD related to unclear RDS in the mature neonate”. Lipid class composition (graph A) on left side) and phosphatidylcholine species composition (graph B) on right side) are indicated as means for each diagnosis. The number of subjects is detailed in Table 1. The numbers above the columns indicate the mean phospholipid concentrations (μmol/l). No statistical comparisons were done; the graphical display should allow a rapid identification of deviations from the controls. The other species are provided in S5 Fig. Only lipid species present at an abundance of > 0.5% are displayed.

We next resolved the level of the disease categories now considering the molecularly or clinically defined diseases (Table 1, S1 Table). As visual inspection of the data shows, in many of the diseases no differences of the phospholipid classes as well as their species composition were identified in comparison to the controls. Such diseases included all those within the categories Diffuse developmental disorders, Immuno-intact host, Immuno-compromised host, Chronic tachypnoe of infancy, Reactive lymphoid lesions, Related to lung vessels/heart and Related to systemic diseases (Table 3, S3 Fig.). We took a closer look at the two categories with diseases likely involving the pulmonary surfactant system. These were Figure SILDs related to the alveolar surfactant region (Fig. 4, S4 Fig.), and ILDs due to unclear RDS in the mature neonate (Fig. 5, S5 Fig.). Due to the low number of cases in many specific diseases statistical analysis was omitted; lipid composition of the latter should be appreciated on an individual basis (S1 Table), except for the group of children with two ABCA3 mutations.

**ABCA3-mutations**. Patients with ABCA3-deficiency from two disease causing mutations had their level of phosphatidylcholine reduced by 50% with pronounced changes of its species profiles, i.e. PC 32:0 lower and increased PC 38:5 (S2 Table). Phosphatidylglycerols PG 32:1, PG 36:1, PG 36:4, PG 38:4 and PG 38:5 were all decreased (S2 Table, Fig. 5, S5 Fig.).

**Fig 6:**
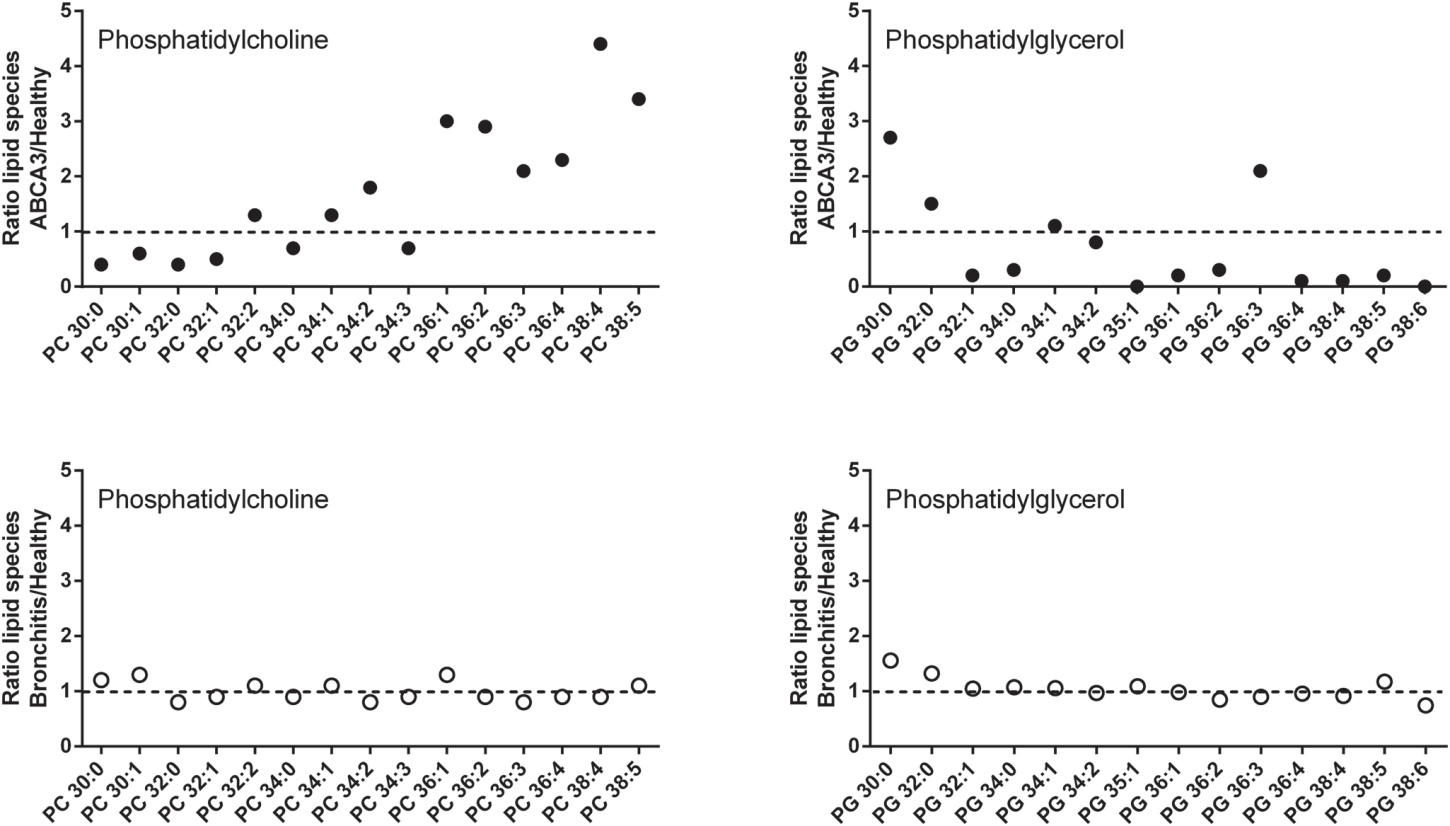
Display of results at the individual diagnosis level “ABCA3 transporter deficiency, two mutations/Controls-healthy” (panel A) and B)) and “Controls-bronchitis/Controls-healthy” (panel C) and panel D)). A) and C) Phosphatidylcholine and B) and D) Phosphatidylglycerol species of the children with ABCA3 transporter deficiency due to two disease causing mutations (n = 5) or controls-bronchitis (n = 10) in relation to that of the control children (n = 11). Mean values of each group were taken for calculation of the ratio. A ratio below 1 indicates lower values in the children with ABCA3 transporter deficiency, i.e. lack of transport of such species into the alveolar space; a ratio above 1 indicates higher values in the children with ABCA3 transporter deficiency, i.e. accumulation of such species in the alveolar space.

We hypothesized that the molecular species composition of broncho-alveolar lavage could give us a clue to the specificity of the failing transporting activity of ABCA3. Thus we normalized lipid species to those found in the control-healthy group and compared to those in the control-bronchitis patients with the same ratio (Fig. 6). Phosphatidylcholine species with the shorter fatty acid chains and none or one double bond (PC 30: to 32:1) including dipalmitoyl-PC (PC 32:0) had ratios significantly (P < 0.01) below 1, indicating reduced delivery to the alveolar compartment. For phosphatidylglycerol the same observation was made for species PG 32:1, PG 34:0 and those with longer fatty acid chains (PG 36 to 38, except PG 36:3), irrespective of their degree of saturation.

## Discussion

Here we present a comprehensive lipidomics profiling of the broncho-alveolar lipids, including surfactant lipids of healthy children. In addition to composition reported for phosphatidylcholine and phosphatidylglycerol [6,31], we inform detailed on the species composition of normal surfactant, except for phosphatidylinositol, which we did not assess. We introduced a second comparison group, controls-bronchitis. From these children with limited bronchial inflammation in the absence of alveolar disease, surfactant material was recovered which had almost the same composition. The large number of lipids assessed differed merely in few species, most of these lipids typically found in cellular membranes or plasma compartments, including phosphatidylethanolamine, phosphatidylserine, plasmalogens, and cholestryl esters (Table 3, Fig. 3, S3 Fig.). Taken together we had built two robust comparison groups for future reference and for the assessment of the children with interstitial lung diseases.

Samples of such children were collected with the help of the biobank of the kinds lung register (www.kids-lung-register.eu), diagnosed in an approach very similar to that described recently [19,32]. Depending on the degree of diagnostic confidence reached and the diagnosis obtained, the children were grouped into categories. These categories were based on the disease classification for biopsied cases [13], and extended to accommodate all entities encountered, as described previously [14]. Within a category, the children with the same diagnosis were sub-categorized. A consistent, methodological sound and simultaneous analysis of the biochemical samples allowed testing of the hypothesis if certain categories may have similar lipid compositions and also comparisons to other studies in the future.

Our hypothesis that the different categories of diagnosis will also capture patient groups with similar lung lavage lipid compositions was confirmed to large extent but not throughout. Clearly the four categories diffuse developmental disorders, chronic tachypnoe of infancy, ILD related to lung vessels and the heart, or ILD related to reactive lymphoid lesions were not associated with any significant changes in their surfactant lipid profile. This was consistent with the supposition that in these interstitial lung diseases the pulmonary surfactant system is not involved.

The two categories ILD in the immune intact host and ILD in the immuno-compromised host contained groups of diseases which proved to have different lipid pattern than others from within the same category. With no changes in the surfactant typical phospholipid classes, plasmalogen, phosphatidylserine, ceramide or cholesteryl esters were present, indicating a role of admixing inflammatory lipoprotein components. In the substantial group of children included here with hypersensitivity pneumonitis, we did not observe small alterations reported in adult patients with this condition, i.e. about 2 fold increased sphingomyelin levels [33,34].

A major finding was the ascertainment that the children in disease categories having in common a clear and significant morphological involvement of the alveoli must not all have changes of the surfactant phospholipids. On the other hand, lipidomic analysis may give deep diagnostic and functional insights, as exemplified in subjects with ABCA3 mutations.

ABCA3-deficiency from two disease causing mutations results in the derangement of type 2 cell surfactant lipid transport into lamellar bodies and subsequent secretion of surfactant into the alveolar space [35,36]. Previously the surfactant phospholipid profile was determined using 2-dimensional thin-layer chromatography and phosphatidylcholine comprised 41% of the total phospholipids in the BAL fluid of 8 patients with ABCA3 mutations [37]. Phosphatidylglycerol was also reduced. This result is consistent to what we found in the patients investigated here. In addition we detail the species changes, which give a clue to the specificity of the failing transporting activity of ABCA3 (Fig. 6). Clearly the major surfactant phosphatidylcholine species PC 30 and 32 and the phosphatidylglycerol species PG 32:1, PG 34:0, PG 36 to 38 irrespective of their degree of saturation, except PG 36:3, were severely depleted in surfactant of these children. Our data confirm and extend results in ABCA3 -/- knockout mice, which showed reduced PC 30:0, 32:1, 32:0, 34:1, 38:6, as well as PG 32:1, 32:0, 34:1, 36:4, 38:6 [35]. In those animals phosphatidylethanolamine was reduced, whereas in newborn and adult mice with conditional deletion of Abca3 phosphatidylethanolamine was increased [35]. In our patients total phosphatidylethanolamine was not altered. Taken together our data demonstrate the lipid species likely to be transported by ABCA3 in vivo.

Lipidomic analysis of BAL from patients with only a single mutation in ABCA3 detected and without lung biopsy to investigate lamellar body morphology by electron microscopy, may give a clue for the presence or absence of ABCA3 deficiency (S1 Table). Two (patients 391, 541) of the 5 patients who died having a single disease causing mutation, had a lipid pattern similar to infants with two mutations, suggesting a non-detected mutation may have been missed by the exon analysis done. On the other, hand patient 719 had a disease causing mutation, a normal surfactant lipid profile and a healthy long term outcome. Thus it is likely that the mutation may have increased the risk for respiratory distress as described [38], without further impact on course. Similarly in patient 1300, who had a single R288K mutation detected, a normal lipid profile and a favorable course make the causal involvement of this relatively frequent but not clearly benign mutation less likely [39]. Lastly, patient 199 had a single disease causing mutation identified and died of respiratory distress. However the lipid profile was normal, making ABCA3-related surfactant dysfunction an unlikely cause of death.

These conclusions may be broadened to children with histologically or clinically significant derangement of the alveolar surfactant space. Of interest, in proven chronic pneumonitis of infancy (CPI), desquamative interstitial pneumonitis and children with familial unclear RDS in the mature neonate, and the diagnostic exclusion of mutations in SFTPB, SFTPC and ABCA3, no major deviations from normal surfactant composition were observed, suggesting the presence of causes outside the surfactant system but resulting in severe alveolar tissue disintegration (Fig. 5, S5 Fig.). Conversely, a subject (507) with the histological diagnosis of NSIP (non-specific interstitial pneumonitis) and in the absence of TTF1, SP-C or ABCA3 mutations had considerably reduced level of phosphatidylcholine and such changes of their lipid species profile suggesting the presence of as yet undetermined defects of the pulmonary surfactant system. Such a defect was proven in a child with a mutation in the TTF1/NKX2–1 gene (659) [17].

Thus lipidomic profiles can yield useful diagnostic or pathophysiologic information in some alveolar diseases. In others they may be of less diagnostic value, but nevertheless may substantiate lack of significant involvement of mechanisms related to lipid metabolism. It is beyond the scope of this manuscript to discuss all individual cases presented here; however when revisiting such patients, recall of the lipidomic profile may be helpful.

Despite the overall large sample size, a weakness of the study was a relatively small case number in several ultra-rare diseases. Thus all statistical results have to be interpreted with great caution. In addition, to overcome this shortage we provide an open access repository of the individual data of all subjects included (S1 Table). It is envisioned that similar and new cases of these rare entities will be merged internationally, to increase the pool of specific diseases available for analysis. This will also make possible to investigate other confounding factors like age or diet [40].

Major strengths of this study are the presentation of the lavage lipidomic profile in two robust non-chILD control and comparison populations, together with inclusion of almost the entire spectrum of children’s interstitial lung disease. In such a group, for the first time patho-physiologically relevant disease markers sampled by standardized technique were comprehensively analyzed in one batch. The species differentiation of the lipids recovered by bronchoalveolar lavage allows an improved differentiation of interfering endogenous and exogenous factors over analysis using thin layer chromatography. Admixture of inflammatory cells as well as of plasma can be identified because of their characteristic lipid composition. Granulocytes are rich in plasmalogenes [41] and plasma leakage into the alveolar space may lead to increased cholesteryl esters due to their high concentration in lipoproteins [42]. More importantly, information on surfactant composition can be obtained with relatively little invasiveness from the lungs and may directly proof involvement of genetically aberrant surfactant metabolic pathways. This study clearly indicated the complexity of BAL lipidomics which may be a useful research tool. The results also define the stage for further selecting investigations in promising subgroups of chILD, as exemplified by our results in patients with ABCA3 mutations. The potential pathophysiological or metabolic role of specific lipid analytes may be proven in suitably sized and homogenous patient groups. Such an ex vivo approach may then complement the recently successfully used in vitro characterization of novel lipid pathways in cultured fibroblasts, identified by next generation sequencing [43].

## Conclusions

Lipidomic profiling identified specific forms of ILD in children with surfactant alterations and characterized the molecular species pattern likely to be transported by the lipid transporter ABCA3 in vivo.

## Supporting Information

S1 Fig **A**: Overview of the presentation arrangement of the lipid results as reported in the manuscript and online supplemental material. **B**: Score plot of a principal component analysis based on the lipid concentrations of 143 lipid subspecies for 115 patients. Groups 1 through 12 are defined in Table 1. Mainly patients from group 9 (ILD related to alveolar surfactant region) separate from the other patients. Percentage numbers in brackets denote the explained variance of the respective principal component.
(PDF)

S2 Fig Phospholipid classes of the control groups (healthy (n = 11), left column of pair; bronchitis (n = 10), right column of pair). Lipid class composition is expressed as % of the analysed displayed lipid classes. The species of the different lipid classes are alo displayed. Data are means. Deviations from 100% are the result minor lipid species present at an abundance of < 0.5%; these were included in the calcualtions, but were not displayed in the graphs. Phosphatidylcholine species annotation was based on the assumption of even numbered carbon chains only. Other glycerophospholipid species were annotated based on the assumption that diacyl species are present. SM species annotation is based on the assumption that a sphingoid base with two hydroxyl groups is present.
(PDF)

S3 Fig Display of results at the disease category level. All lipid classes and their species composition are given as individual results of all patients included in the study according the disease category they belong to. Phosphatidylcholine species annotation was based on the assumption of even numbered carbon chains only. Other glycerophospholipid species were annotated based on the assumption that diacyl species are present. SM species annotation is based on the assumption that a sphingoid base with two hydroxyl groups is present. The statistical comparisons were done by ANOVA and Dunn’s post hoc test; all significant results are displayed in each figure. The significance of ANOVA was determined by comparison of the raw P value given with the one calculated to take multiple comparisons into consideration, (P<0.0182 instead of P<0.05). The significant results of Dunn’s post hoc tests are indicated as following: ____ = P < 0.05, _ _ _ = P < 0.01, and …. = P < 0.001.
(PDF)

S4 Fig Display of results at the individual diagnosis level for the disease category “ILD-related to the alveolar surfactant region”. Phospholipid class composition and all species compositions of all classes assessed are indicated as means for each diagnosis. The number of subjects per group is detailed in Table 1. The numbers above the columns indicate the phospholipid concentration analysed (μmol/l). No statistical comparisons were done. Deviations from 100% are the result minor lipid species present at an abundance of < 0.5%; these were included in the calculations, but were not displayed in the graphs.
(PDF)

S5 Fig Display of results at the individual diagnosis level for the disease category “Unclear RDS in the mature neonate”. Phospholipid class composition and all species compositions of all classes assessed are indicated as means for each diagnosis. The number of subjects per group is detailed in Table 1. The numbers above the columns indicate the phospholipid concentration analysed (μmol/l). No statistical comparisons were done. Deviations from 100% are the result minor lipid species present at an abundance of < 0.5%; these were included in the calcualtions, but were not displayed in the graphs.
(PDF)

S1 Methods (PDF)

S1 Table All individual values ordered by patients included in the final analysis, disease category and subcategory. All lipid results are indicated. Note that in 7 of the patients PE P and in 2 PG were not measured. Results are indicated in the units above the columns, for the different lipid species, as percent of the lipid class. Phosphatidylcholine species annotation was based on the assumption of even numbered carbon chains only. Other glycerophospholipid species were annotated based on the assumption that diacyl species are present. SM species annotation is based on the assumption that a sphingoid base with two hydroxyl groups is present.
(PDF)

S2 Table Phosphatidylcholine and phosphatidylglycerol and their species composition in BAL from healthy children (n = 11) in comparison to children with two disease causing ABCA3 mutations (n = 5). The individual values of the other phospholipid classes and their species are depicted in the S1 Table.
(PDF)

S3 Table Column statistics of the different patient groups depicted in the S3 Fig. indicating parameters in addition to means. (PDF)
