# Supplementary material for: Surfactant Lipidomics in Healthy Children and Childhood Interstitial Lung Disease
Source: PLoS One. 2015 Feb 18;10(2):e0117985. doi: 10.1371/journal.pone.0117985 (PMC4333572; doi:10.1371/journal.pone.0117985)
Supplement: S1 Fig — B: Score plot of a principal component analysis based on the lipid concentrations of 143 lipid subspecies for 115 patients. Groups 1 through 12 are defined in Table 1. Mainly patients from group 9 (ILD related to alveolar surfactant region) separate from the other patients. Percentage numbers in brackets denote the explained variance of the respective principal component. (PDF) [file pone.0117985.s001.pdf]

A)

Overview of the presentation of lipid results

**Patient group**	**Goal**	**Body of manuscript**		**Supplemental data**
		**Lipid classes**	**Phosphatidylcholine species**	**All Lipid classes and species**
A) Controls healthy, Controls bronchitis	Show normal values and their variation between the two compa-rison groups	Fig. 2<br>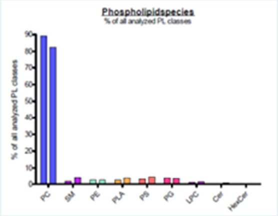 	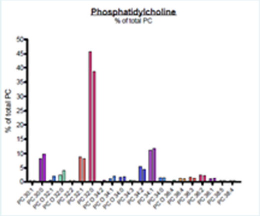 	Figure S2<br>Same as Fig. 2 plus species of SM, PE, Plasmalogens, PS, PG, lysoPC, Ceramide and Cholesteryl esters
B) Disease categories	Compare to con-trols, show indivi-dual scatter, <u>displa-yed are only those with significant dif-ferences to controls</u>	Fig. 3<br>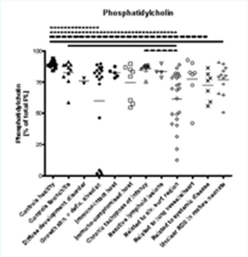 	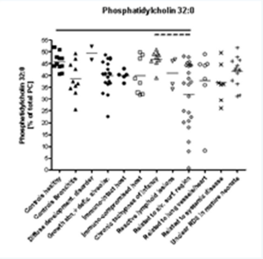 	Figure S3<br>Same as Fig. 3 plus species of SM, PE, Plasmalogens, PS, PG, lysoPC, Ceramide and Cholesteryl-esters
C) Molecular or clinically defined diseases	Show disease specific results; <u>displayed are only those sub-species with sign. differen-ces to Controls</u>	Figs. 4, 5<br>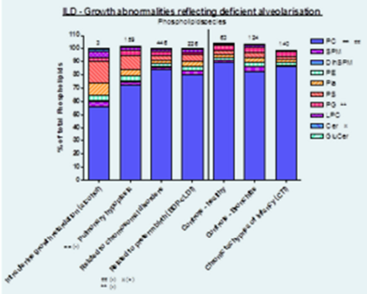 	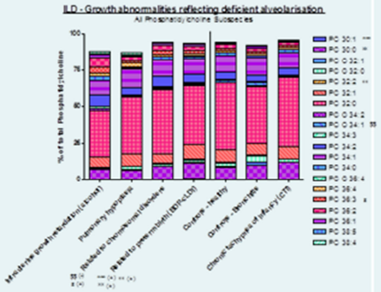 	Figures S4, S5<br>Same as Fig. 4, 5 plus species of SM, PE, Plasmalogens, PS, PG, lysoPC, Ceramide and Cholesteryl-esters
D) All	Show all individual data			Table S1<br>Tables with one row per subject

B)

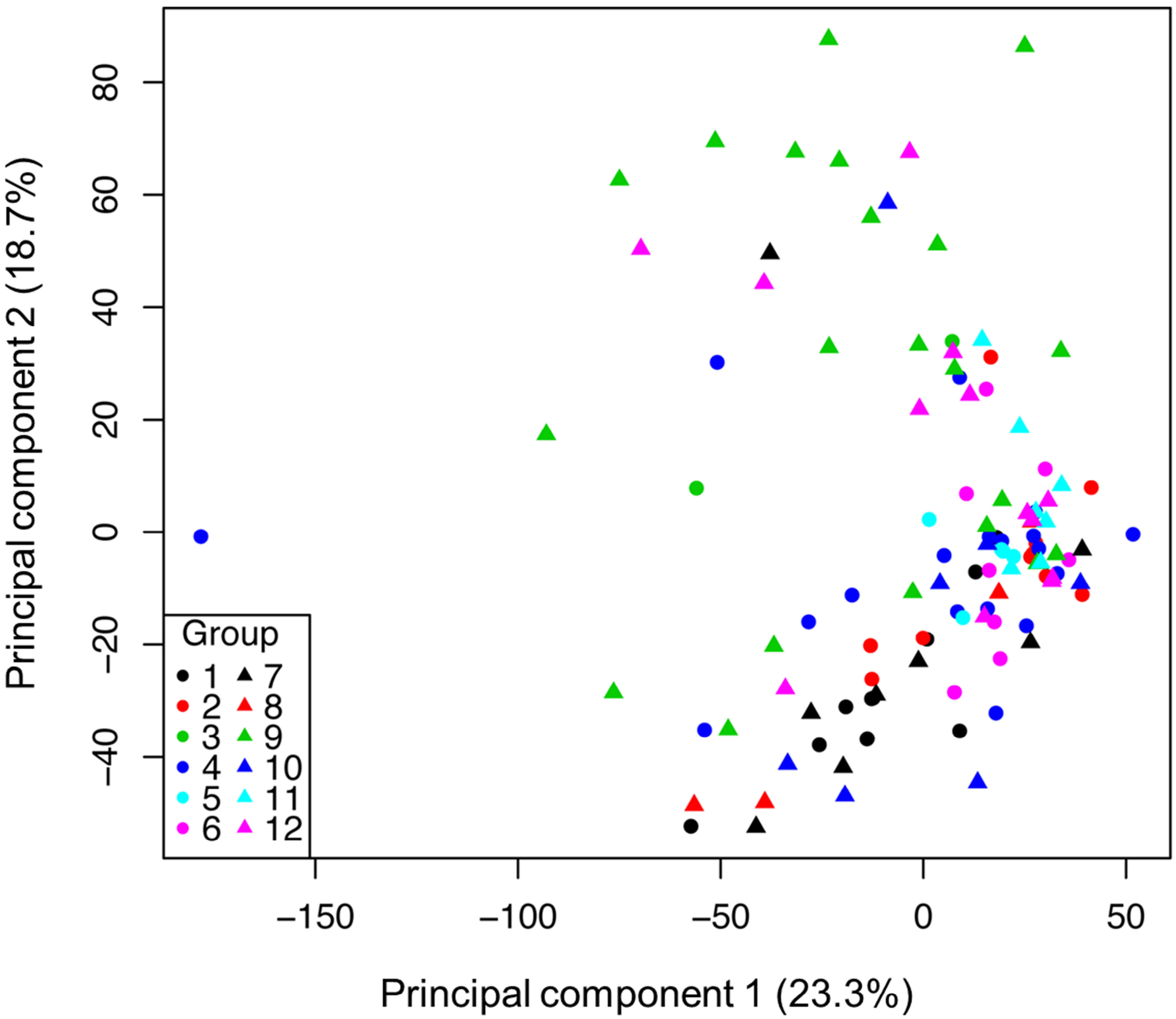
Principal component 2 (18.7%)
80
60
40
20
0
−20
−40
Group
1
2
3
4
5
6
7
8
9
10
11
12
−150
−100
−50
0
50
Principal component 1 (23.3%)
