# Supplementary material for: Surfactant Lipidomics in Healthy Children and Childhood Interstitial Lung Disease
Source: PLoS One. 2015 Feb 18;10(2):e0117985. doi: 10.1371/journal.pone.0117985 (PMC4333572; doi:10.1371/journal.pone.0117985)
Supplement: S2 Fig — Lipid class composition is expressed as % of the analysed displayed lipid classes. The species of the different lipid classes are alo displayed. Data are means. Deviations from 100% are the result minor lipid species present at an abundance of < 0.5%; these were included in the calcualtions, but were not displayed in the graphs. Phosphatidylcholine species annotation was based on the assumption of even numbered carbon chains only. Other glycerophospholipid species were annotated based on the assumption that diacyl species are present. SM species annotation is based on the assumption that a sphingoid base with two hydroxyl groups is present. (PDF) [file pone.0117985.s002.pdf]

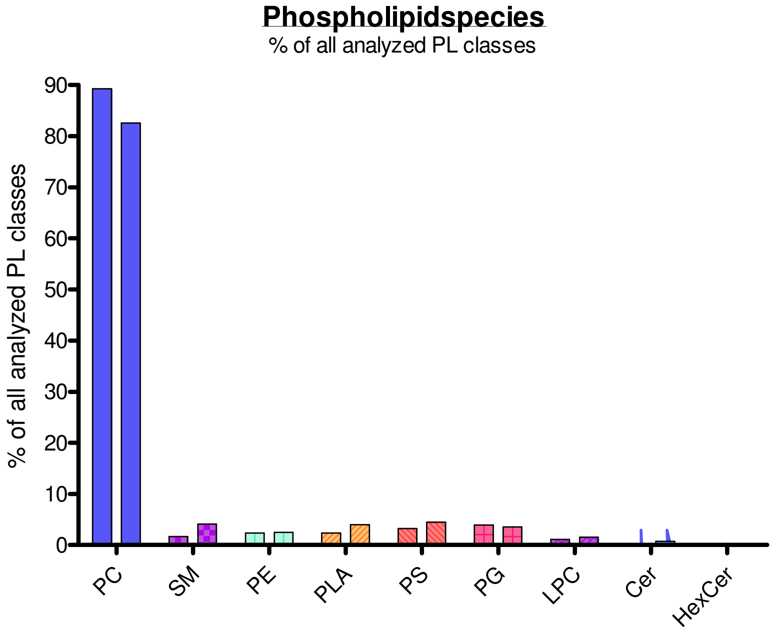
Phospholipidspecies
% of all analyzed PL classes
% of all analyzed PL classes
0
10
20
30
40
50
60
70
80
90
PC
SM
PE
PLA
PS
PG
LPC
Cer
HexCer

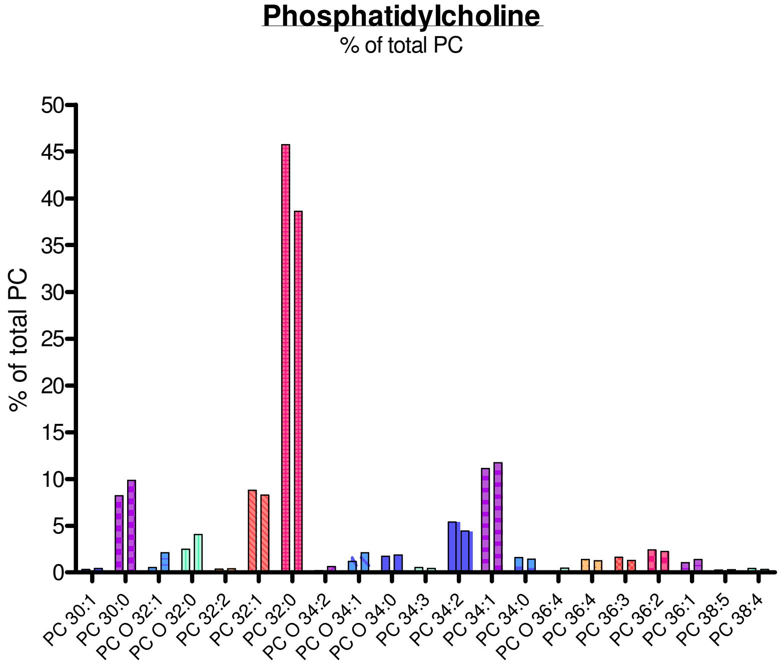
Phosphatidylcholine
% of total PC
% of total PC
0
5
10
15
20
25
30
35
40
45
50
PC 30:1
PC 30:0
PC O 32:1
PC O 32:0
PC 32:2
PC 32:1
PC 32:0
PC O 34:2
PC O 34:1
PC O 34:0
PC 34:3
PC 34:2
PC 34:1
PC 34:0
PC O 36:4
PC 36:4
PC 36:3
PC 36:2
PC 36:1
PC 38:5
PC 38:4

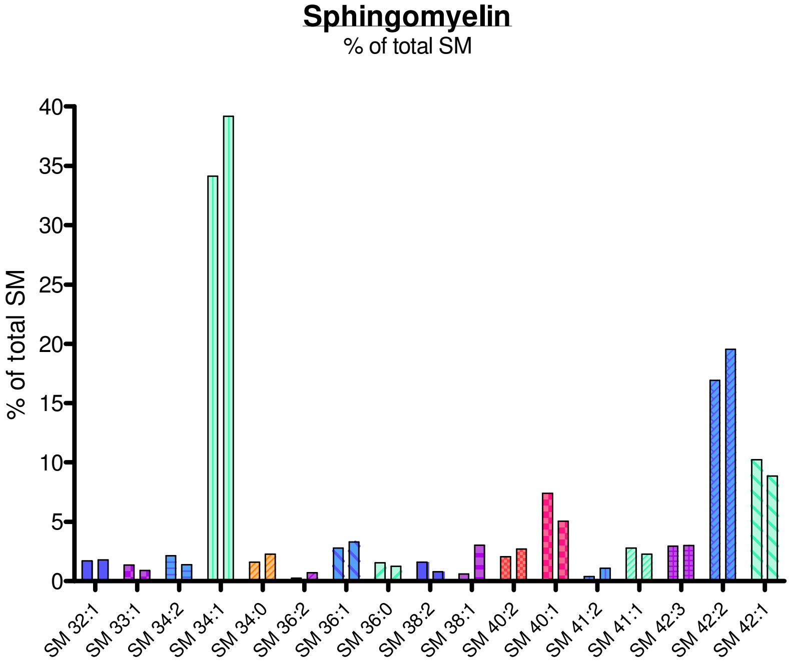
Sphingomyelin
% of total SM
% of total SM
0
5
10
15
20
25
30
35
40
SM 32:1
SM 33:1
SM 34:2
SM 34:1
SM 34:0
SM 36:2
SM 36:1
SM 36:0
SM 38:2
SM 38:1
SM 40:2
SM 40:1
SM 41:2
SM 41:1
SM 42:3
SM 42:2
SM 42:1

Phosphatidylethanolamine

% of total PE

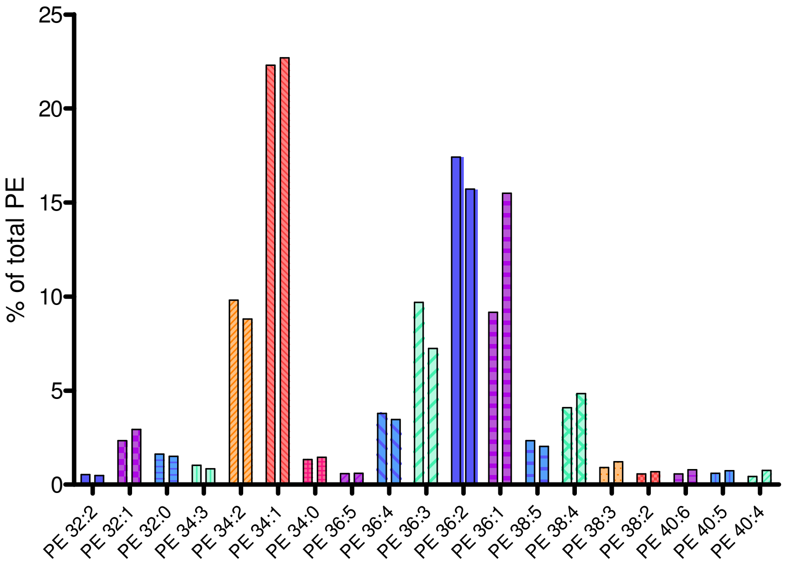

Plasmalogens

% of total PE P

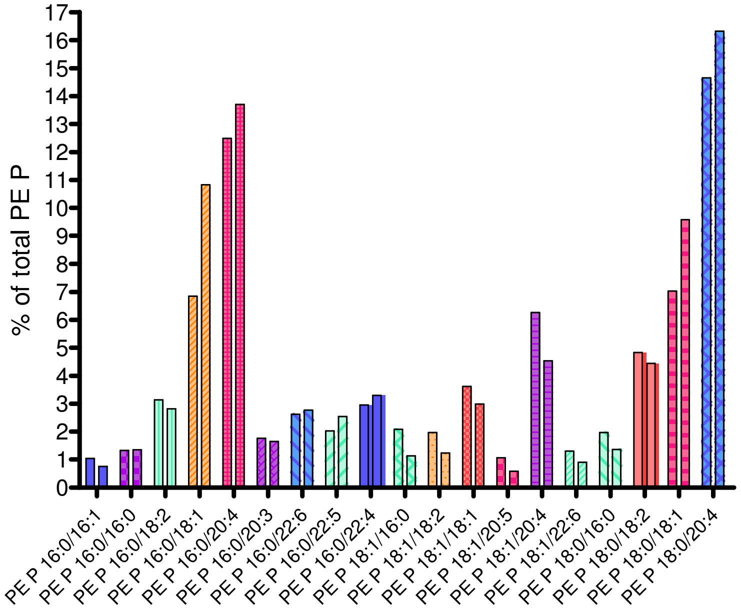

Phosphatidylserine

% of total PS

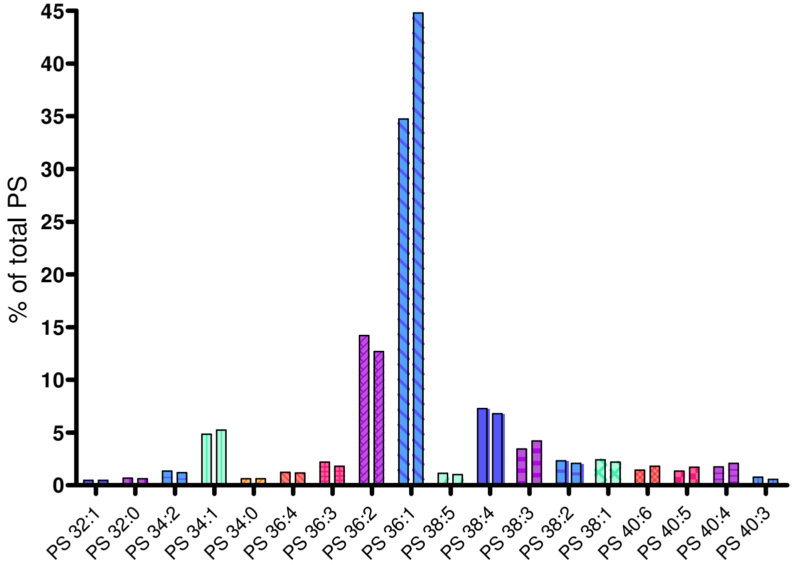

Phosphatidylglycerol

% of total PG

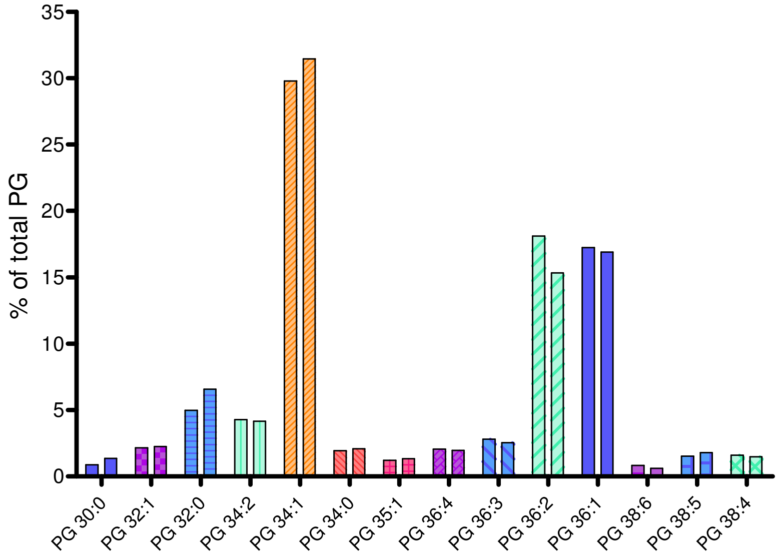

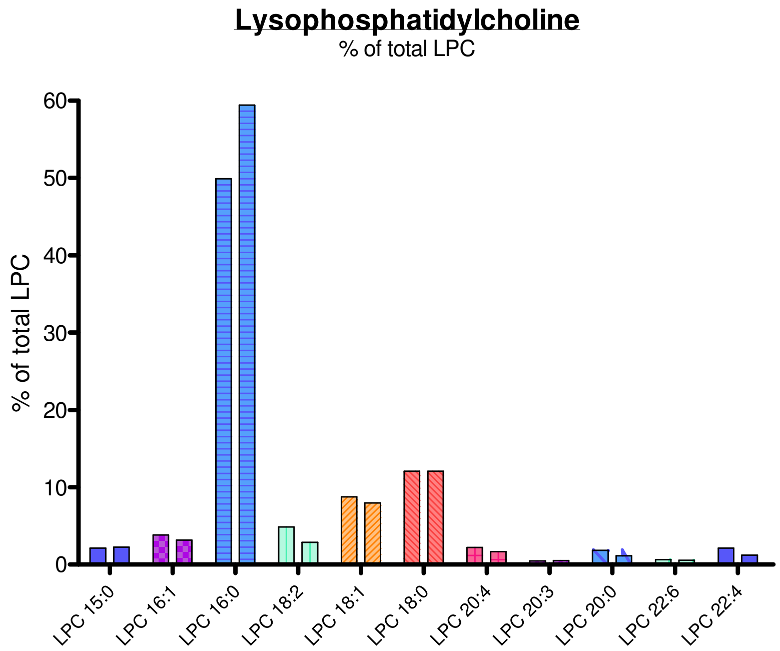
Lysophosphatidylcholine
% of total LPC
% of total LPC
0
10
20
30
40
50
60
LPC 15:0
LPC 16:1
LPC 16:0
LPC 18:2
LPC 18:1
LPC 18:0
LPC 20:4
LPC 20:3
LPC 20:0
LPC 22:6
LPC 22:4

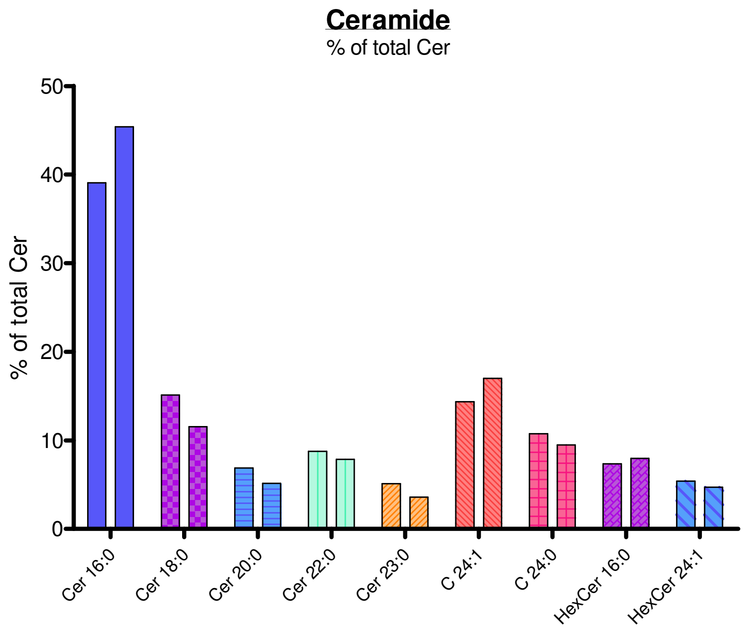
Ceramide
% of total Cer
% of total Cer
0
10
20
30
40
50
Cer 16:0
Cer 18:0
Cer 20:0
Cer 22:0
Cer 23:0
C 24:1
C 24:0
HexCer 16:0
HexCer 24:1

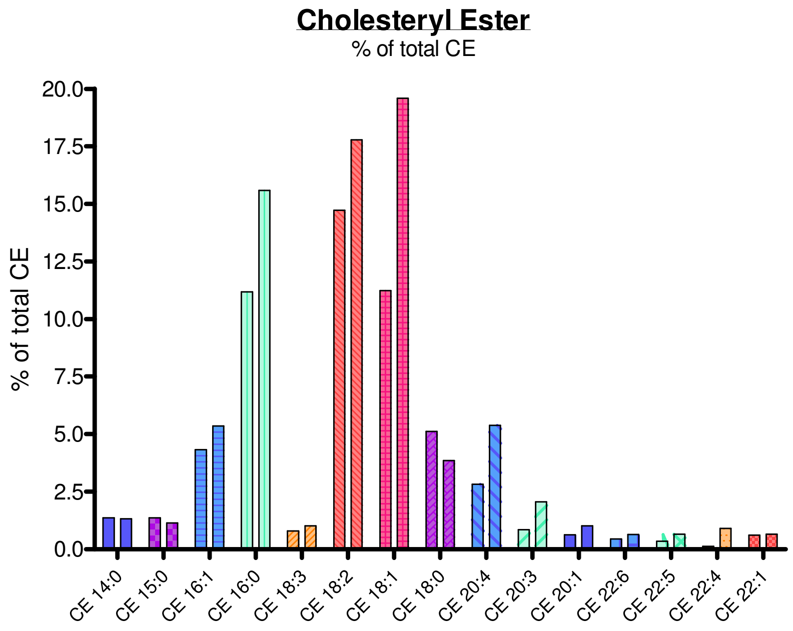
Cholesteryl Ester
% of total CE
% of total CE
0.0
2.5
5.0
7.5
10.0
12.5
15.0
17.5
20.0
CE 14:0
CE 15:0
CE 16:1
CE 16:0
CE 18:3
CE 18:2
CE 18:1
CE 18:0
CE 20:4
CE 20:3
CE 20:1
CE 22:6
CE 22:5
CE 22:4
CE 22:1
