# Supplementary material for: Surfactant Lipidomics in Healthy Children and Childhood Interstitial Lung Disease
Source: PLoS One. 2015 Feb 18;10(2):e0117985. doi: 10.1371/journal.pone.0117985 (PMC4333572; doi:10.1371/journal.pone.0117985)

Phospholipid classes

All analyzed Phospholipid classes (without Cholesterol)

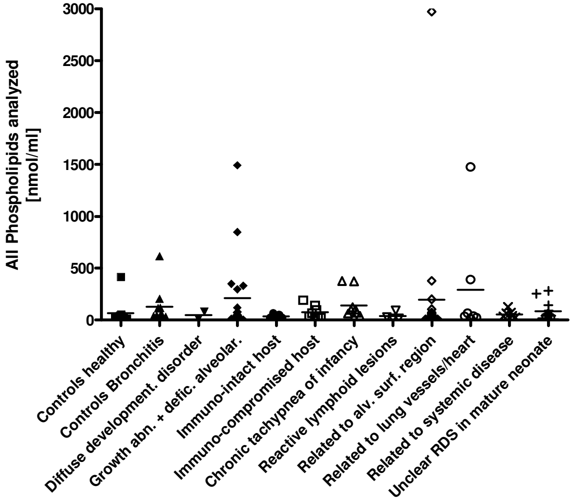

Oneway Anova
p = 0.3202

Dunn's Post-Test
$p < 0.05$
$p < 0.01$
$p < 0.001$

Phosphatidylcholine

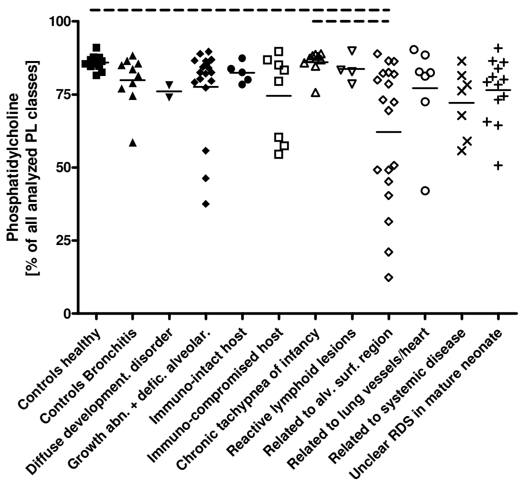

Oneway Anova
p = 0.0020

Dunn's post test
$p < 0.05$
$p < 0.01$
$p < 0.001$

Sphingomyelin

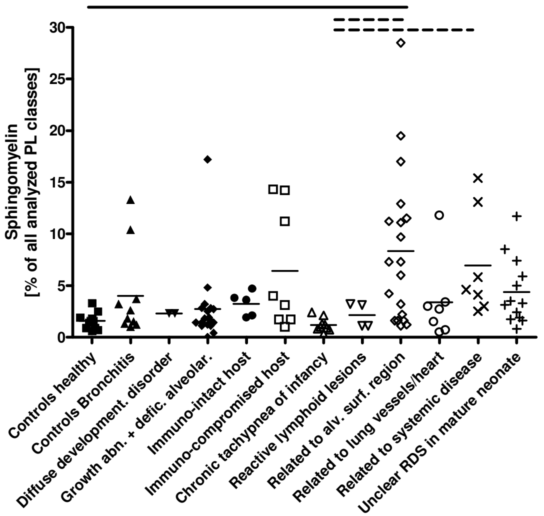

Oneway Anova
p = 0.0002

Dunn's post test
$p < 0.05$
$p < 0.01$
$p < 0.001$

Phosphatidylethanolamine

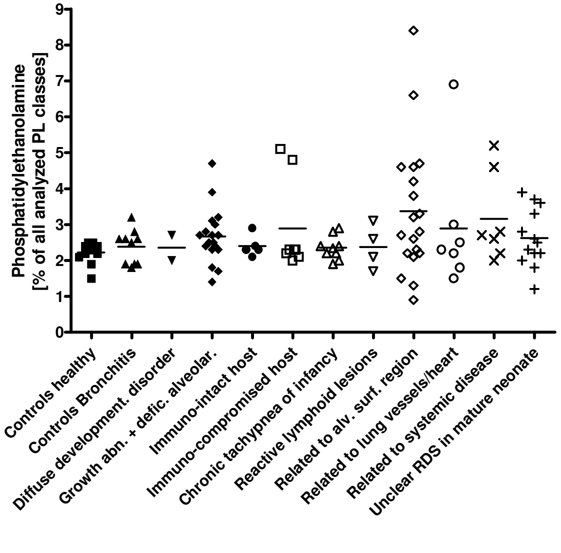

Oneway Anova
p = 0.6421

Dunn's post test
$p < 0.05$
$p < 0.01$
$p < 0.001$

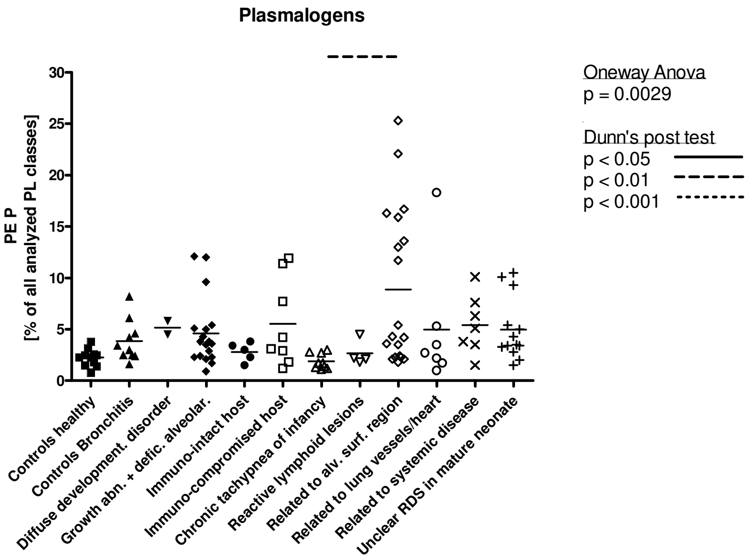
Plasmalogens
PE P
[% of all analyzed PL classes]
Controls healthy
Controls Bronchitis
Diffuse development. disorder
Growth abn. + defic. alveolar.
Immuno-intact host
Immuno-compromised host
Chronic tachypnea of infancy
Reactive lymphoid lesions
Related to alv. surf. region
Related to lung vessels/heart
Related to systemic disease
Unclear RDS in mature neonate
Oneway Anova
p = 0.0029
Dunn's post test
p < 0.05
p < 0.01
p < 0.001

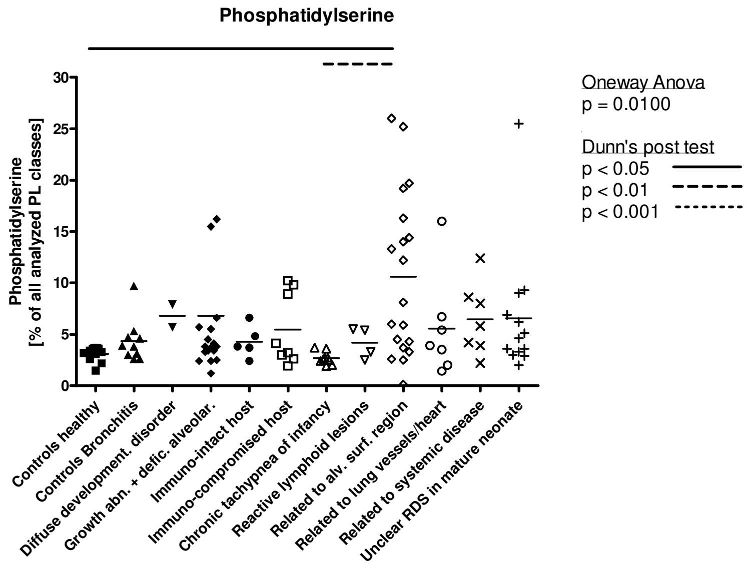
Phosphatidylserine
Phosphatidylserine
[% of all analyzed PL classes]
Controls healthy
Controls Bronchitis
Diffuse development. disorder
Growth abn. + defic. alveolar.
Immuno-intact host
Immuno-compromised host
Chronic tachypnea of infancy
Reactive lymphoid lesions
Related to alv. surf. region
Related to lung vessels/heart
Related to systemic disease
Unclear RDS in mature neonate
Oneway Anova
p = 0.0100
Dunn's post test
p < 0.05
p < 0.01
p < 0.001

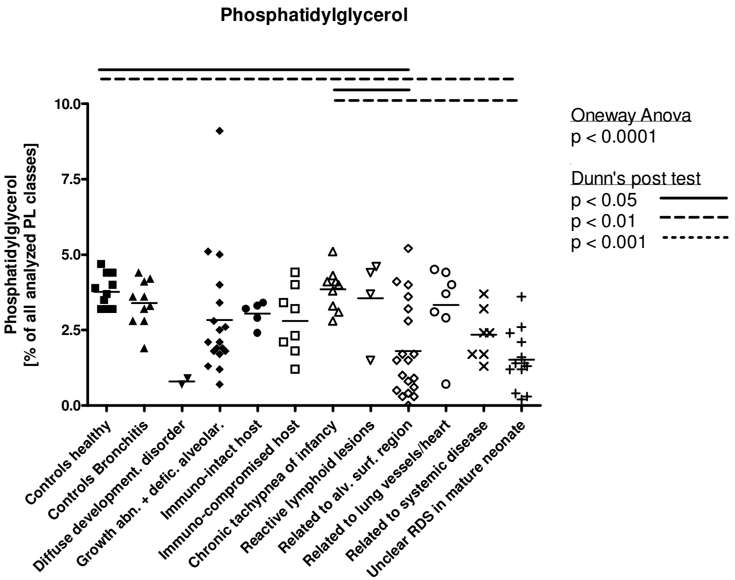
Phosphatidylglycerol
Phosphatidylglycerol
[% of all analyzed PL classes]
Controls healthy
Controls Bronchitis
Diffuse development. disorder
Growth abn. + defic. alveolar.
Immuno-intact host
Immuno-compromised host
Chronic tachypnea of infancy
Reactive lymphoid lesions
Related to alv. surf. region
Related to lung vessels/heart
Related to systemic disease
Unclear RDS in mature neonate
Oneway Anova
p < 0.0001
Dunn's post test
p < 0.05
p < 0.01
p < 0.001

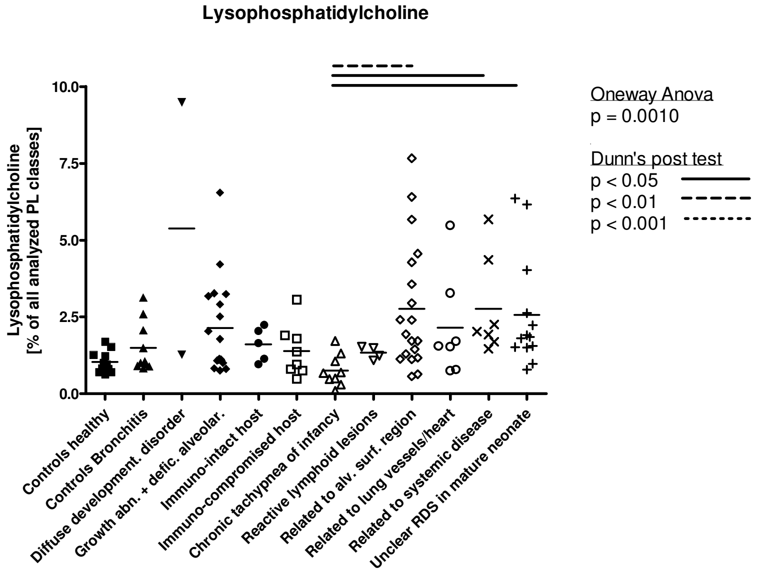
Lysophosphatidylcholine
Lysophosphatidylcholine
[% of all analyzed PL classes]
Controls healthy
Controls Bronchitis
Diffuse development. disorder
Growth abn. + defic. alveolar.
Immuno-intact host
Immuno-compromised host
Chronic tachypnea of infancy
Reactive lymphoid lesions
Related to alv. surf. region
Related to lung vessels/heart
Related to systemic disease
Unclear RDS in mature neonate
Oneway Anova
p = 0.0010
Dunn's post test
p < 0.05
p < 0.01
p < 0.001

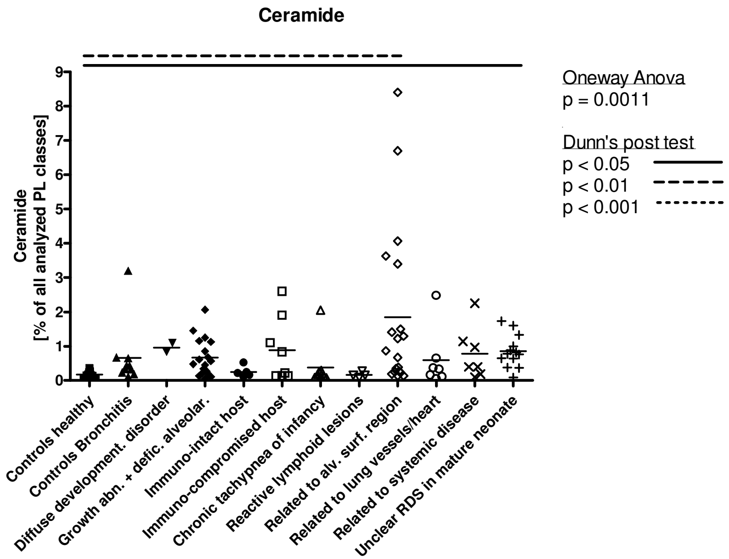

Ceramide
Ceramide [% of all analyzed PL classes]
Oneway Anova
p = 0.0011
Dunn's post test
p < 0.05
p < 0.01
p < 0.001
Controls healthy
Controls Bronchitis
Diffuse development. disorder
Growth abn. + defic. alveolar.
Immuno-intact host
Immuno-compromised host
Chronic tachypnea of infancy
Reactive lymphoid lesions
Related to alv. surf. region
Related to lung vessels/heart
Related to systemic disease
Unclear RDS in mature neonate

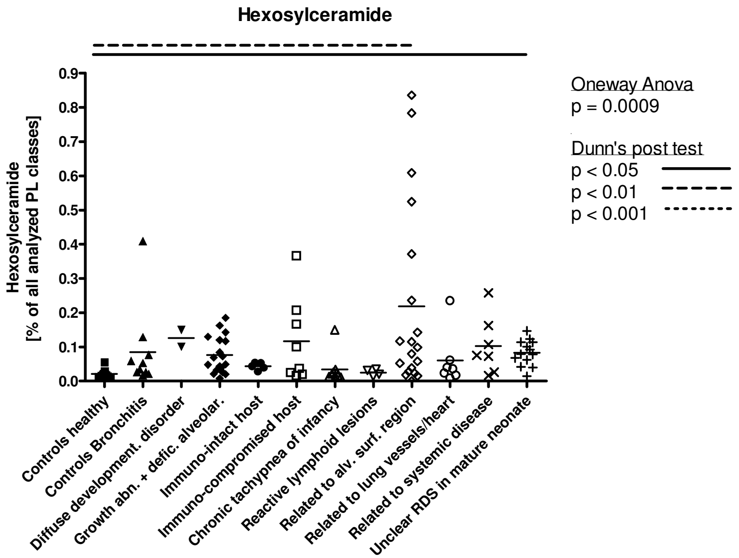

Hexosylceramide
Hexosylceramide [% of all analyzed PL classes]
Oneway Anova
p = 0.0009
Dunn's post test
p < 0.05
p < 0.01
p < 0.001
Controls healthy
Controls Bronchitis
Diffuse development. disorder
Growth abn. + defic. alveolar.
Immuno-intact host
Immuno-compromised host
Chronic tachypnea of infancy
Reactive lymphoid lesions
Related to alv. surf. region
Related to lung vessels/heart
Related to systemic disease
Unclear RDS in mature neonate

Sphingomyelin

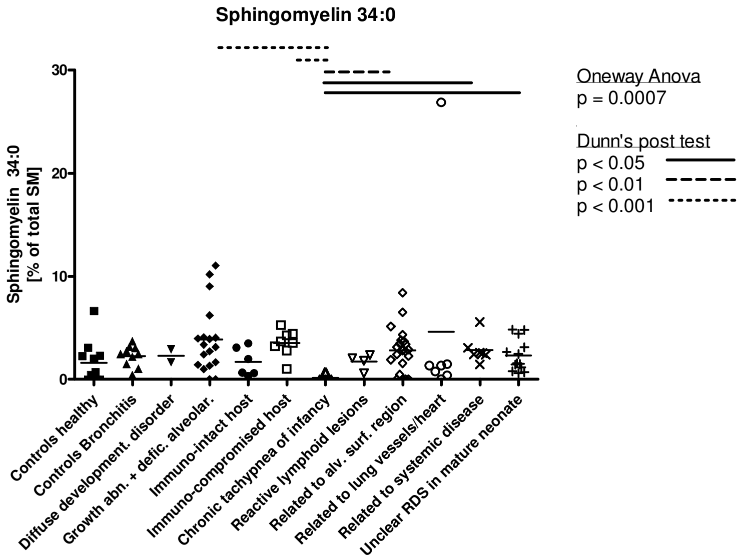

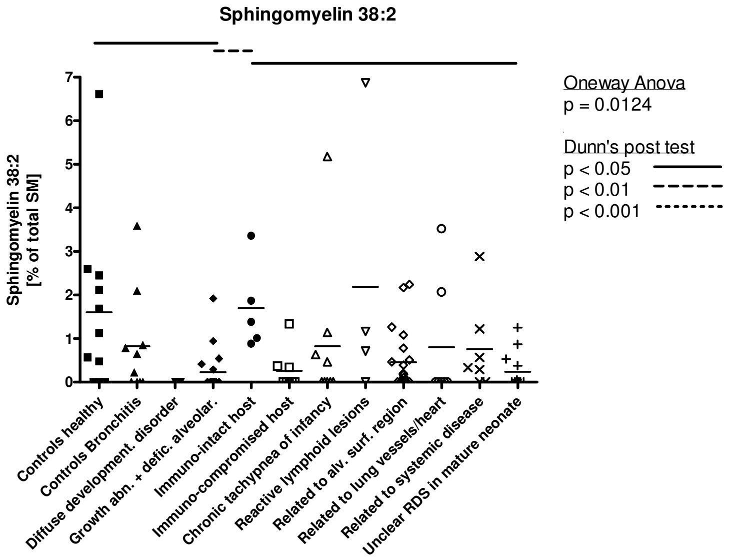

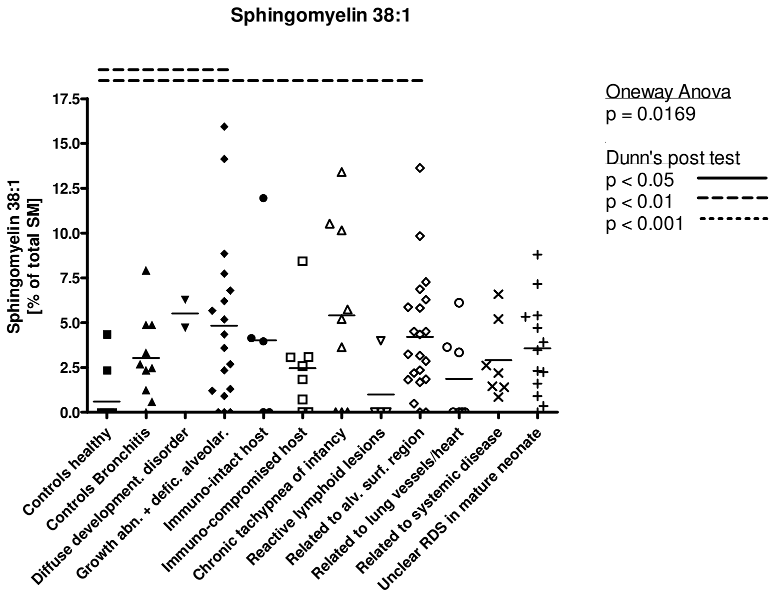

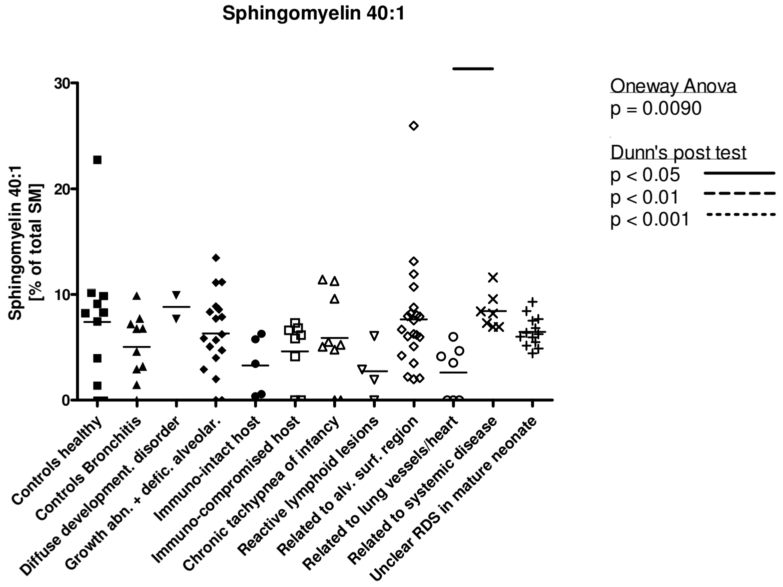

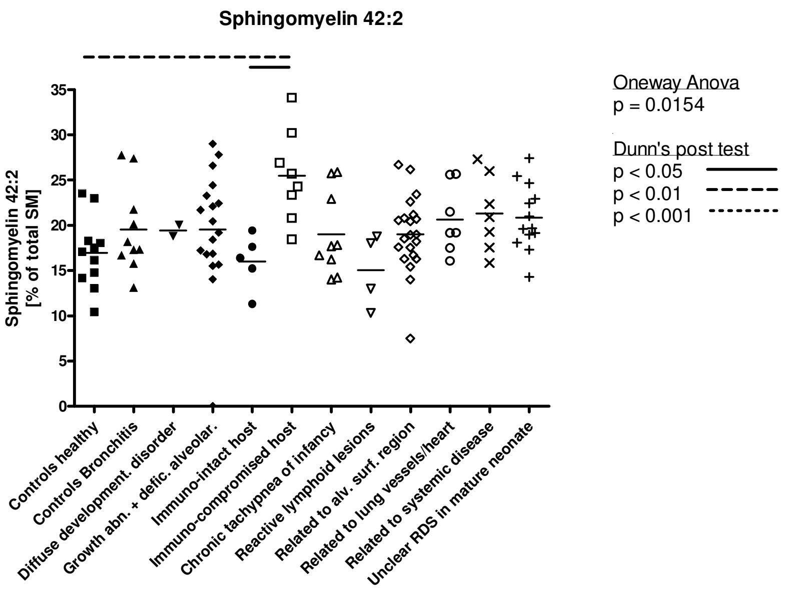

Sphingomyelin 42:2
Sphingomyelin 42:2
[% of total SM]
0
5
10
15
20
25
30
35
Controls healthy
Controls Bronchitis
Diffuse development. disorder
Growth abn. + defic. alveolar.
Immuno-intact host
Immuno-compromised host
Chronic tachypnea of infancy
Reactive lymphoid lesions
Related to alv. surf. region
Related to lung vessels/heart
Related to systemic disease
Unclear RDS in mature neonate
Oneway Anova
p = 0.0154
Dunn's post test
p < 0.05
p < 0.01
p < 0.001

Phosphatidylcholine

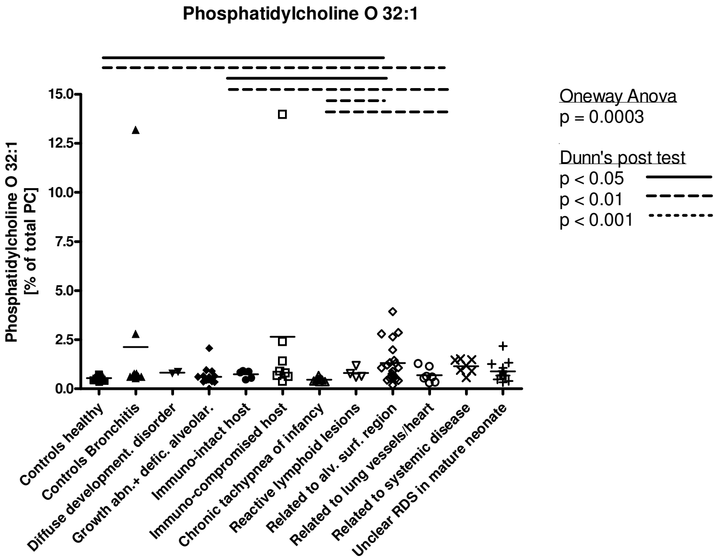

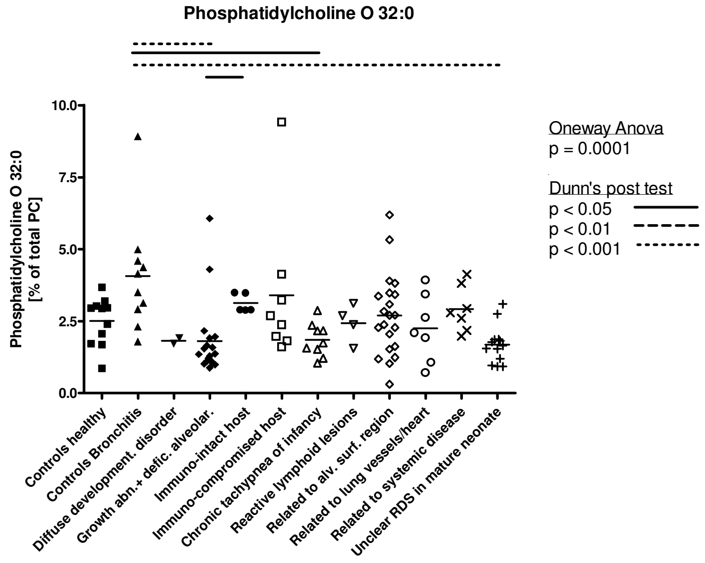

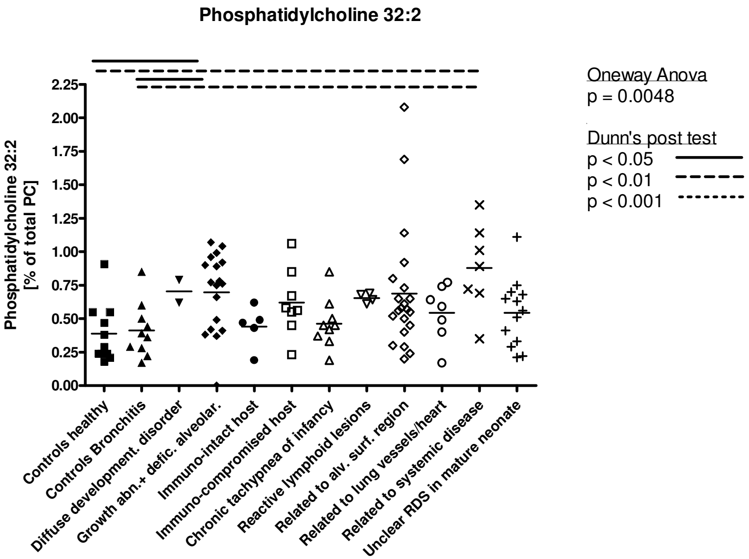

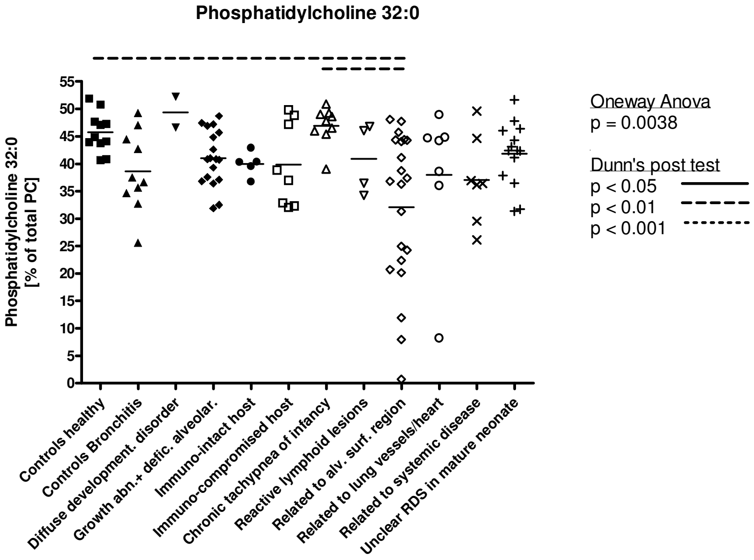

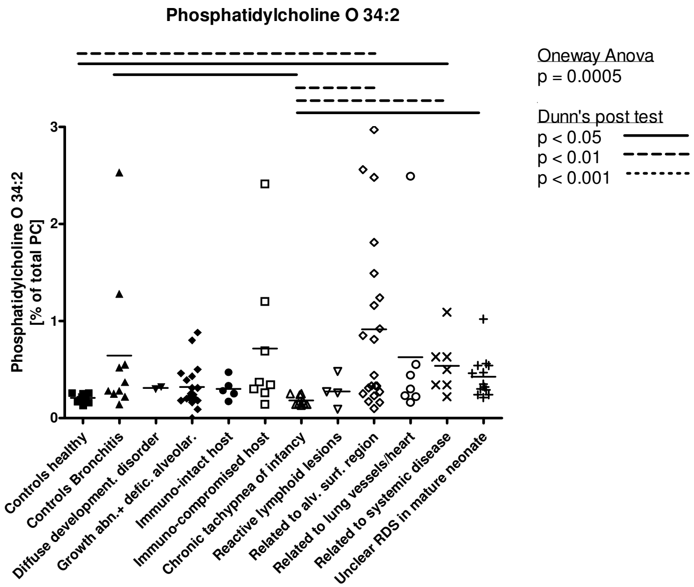
Phosphatidylcholine O 34:2
Phosphatidylcholine O 34:2 [% of total PC]
0
1
2
3
Oneway Anova
p = 0.0005
Dunn's post test
p < 0.05
p < 0.01
p < 0.001
Controls healthy
Controls Bronchitis
Diffuse development. disorder
Growth abn.+ defic. alveolar.
Immuno-intact host
Immuno-compromised host
Chronic tachypnea of infancy
Reactive lymphoid lesions
Related to alv. surf. region
Related to lung vessels/heart
Related to systemic disease
Unclear RDS in mature neonate

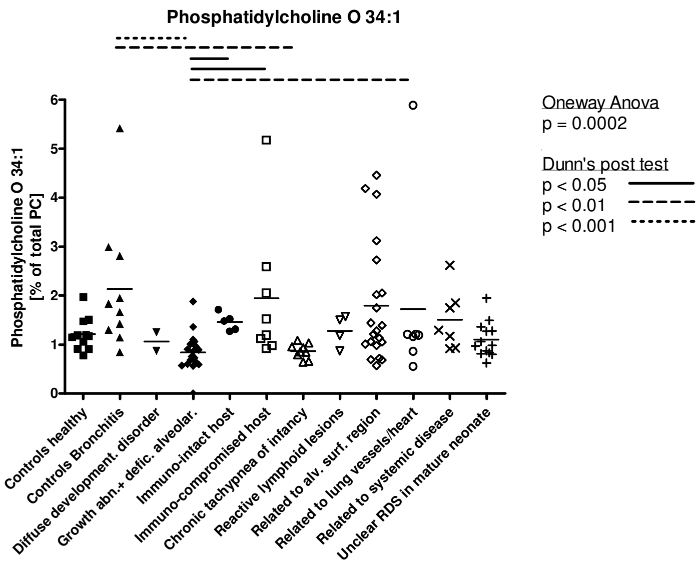
Phosphatidylcholine O 34:1
Phosphatidylcholine O 34:1 [% of total PC]
0
1
2
3
4
5
6
Oneway Anova
p = 0.0002
Dunn's post test
p < 0.05
p < 0.01
p < 0.001
Controls healthy
Controls Bronchitis
Diffuse development. disorder
Growth abn.+ defic. alveolar.
Immuno-intact host
Immuno-compromised host
Chronic tachypnea of infancy
Reactive lymphoid lesions
Related to alv. surf. region
Related to lung vessels/heart
Related to systemic disease
Unclear RDS in mature neonate

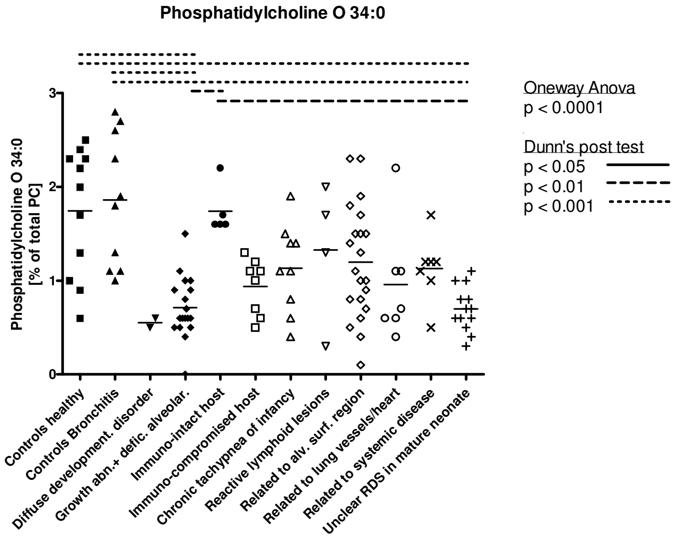
Phosphatidylcholine O 34:0
Phosphatidylcholine O 34:0 [% of total PC]
0
1
2
3
Oneway Anova
p < 0.0001
Dunn's post test
p < 0.05
p < 0.01
p < 0.001
Controls healthy
Controls Bronchitis
Diffuse development. disorder
Growth abn.+ defic. alveolar.
Immuno-intact host
Immuno-compromised host
Chronic tachypnea of infancy
Reactive lymphoid lesions
Related to alv. surf. region
Related to lung vessels/heart
Related to systemic disease
Unclear RDS in mature neonate

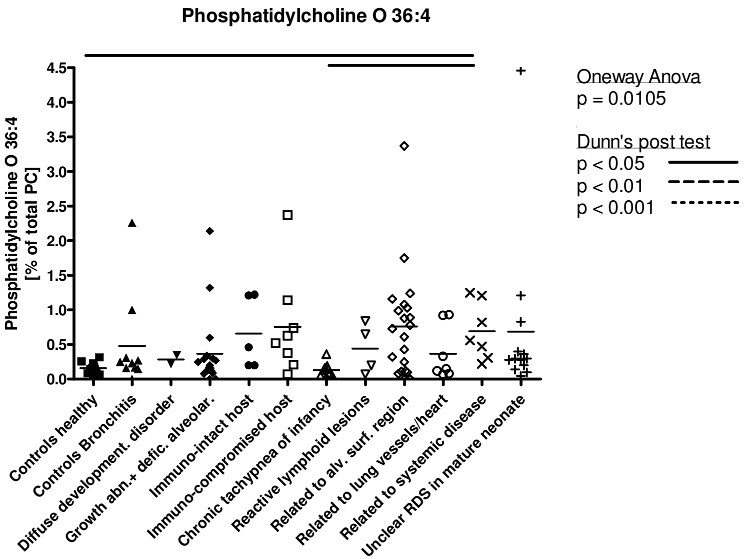
Phosphatidylcholine O 36:4
Phosphatidylcholine O 36:4 [% of total PC]
0.0
0.5
1.0
1.5
2.0
2.5
3.0
3.5
4.0
4.5
Oneway Anova
p = 0.0105
Dunn's post test
p < 0.05
p < 0.01
p < 0.001
Controls healthy
Controls Bronchitis
Diffuse development. disorder
Growth abn.+ defic. alveolar.
Immuno-intact host
Immuno-compromised host
Chronic tachypnea of infancy
Reactive lymphoid lesions
Related to alv. surf. region
Related to lung vessels/heart
Related to systemic disease
Unclear RDS in mature neonate

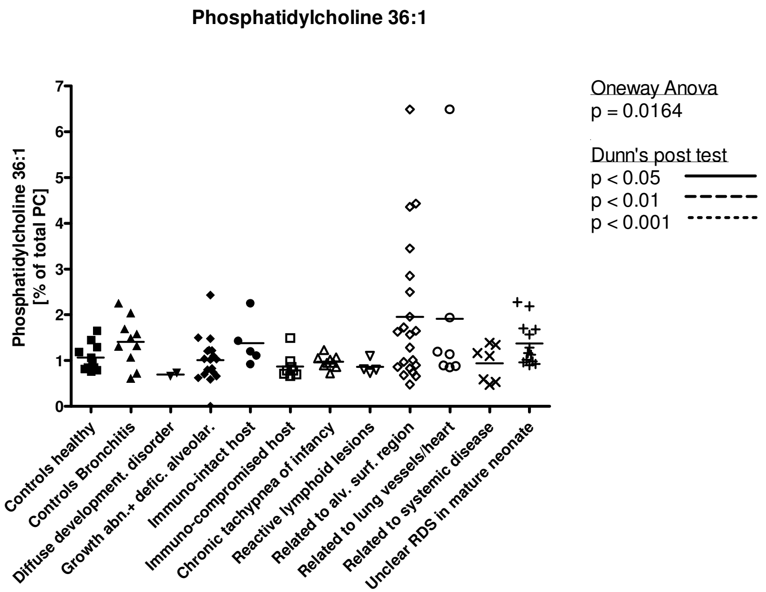
Phosphatidylcholine 36:1
Phosphatidylcholine 36:1 [% of total PC]
0
1
2
3
4
5
6
7
Controls healthy
Controls Bronchitis
Diffuse development. disorder
Growth abn.+ defic. alveolar.
Immuno-intact host
Immuno-compromised host
Chronic tachypnea of infancy
Reactive lymphoid lesions
Related to alv. surf. region
Related to lung vessels/heart
Related to systemic disease
Unclear RDS in mature neonate
Oneway Anova
p = 0.0164
Dunn's post test
p < 0.05
p < 0.01
p < 0.001

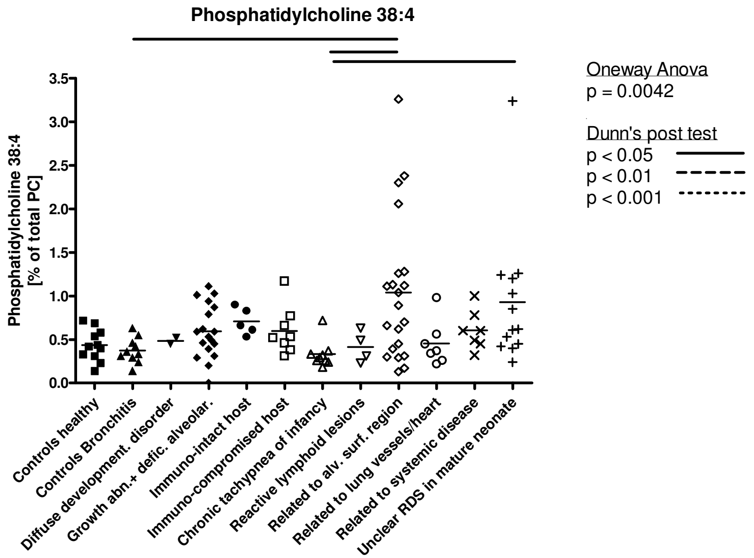
Phosphatidylcholine 38:4
Phosphatidylcholine 38:4 [% of total PC]
0.0
0.5
1.0
1.5
2.0
2.5
3.0
3.5
Controls healthy
Controls Bronchitis
Diffuse development. disorder
Growth abn.+ defic. alveolar.
Immuno-intact host
Immuno-compromised host
Chronic tachypnea of infancy
Reactive lymphoid lesions
Related to alv. surf. region
Related to lung vessels/heart
Related to systemic disease
Unclear RDS in mature neonate
Oneway Anova
p = 0.0042
Dunn's post test
p < 0.05
p < 0.01
p < 0.001

Phosphatidylethanolamine

Phosphatidylethanolamine 34:2

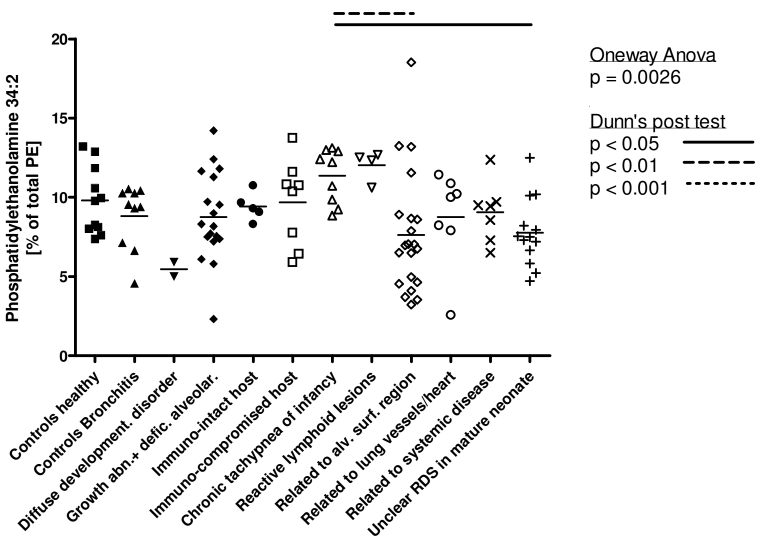

Phosphatidylethanolamine 36:3

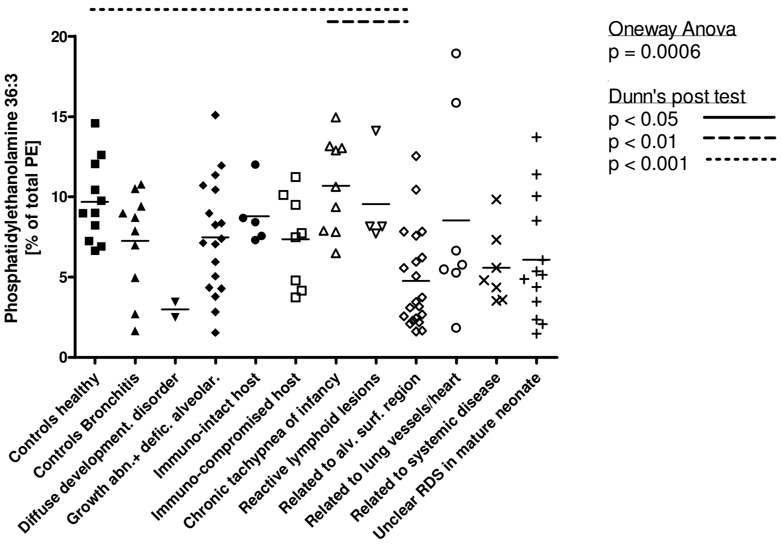

Phosphatidylethanolamine 36:2

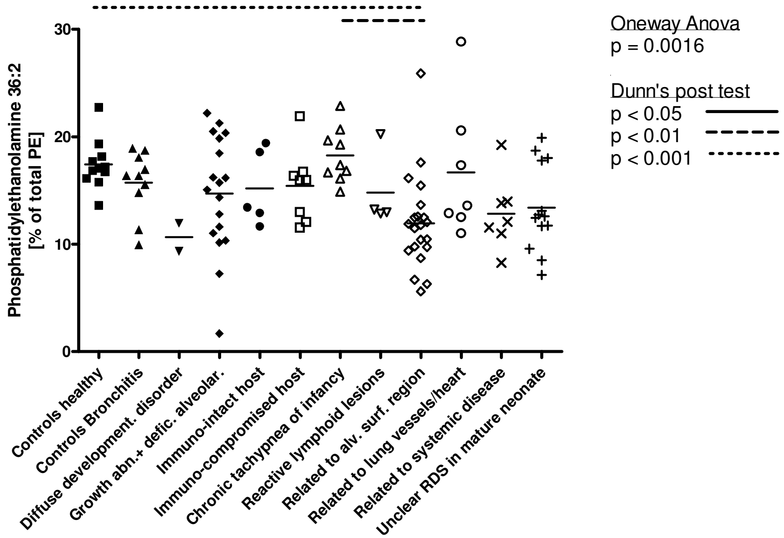

Phosphatidylethanolamine 38:5

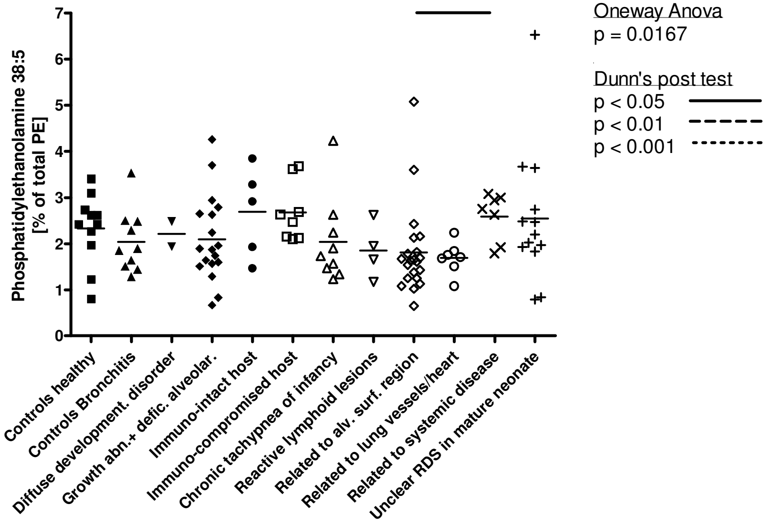

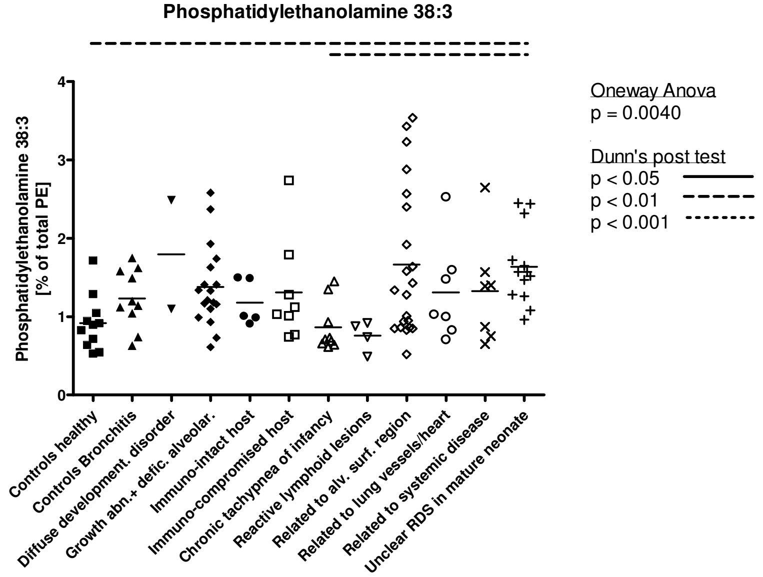
Phosphatidylethanolamine 38:3
Phosphatidylethanolamine 38:3
[% of total PE]
Controls healthy
Controls Bronchitis
Diffuse development. disorder
Growth abn.+ defic. alveolar.
Immuno-intact host
Immuno-compromised host
Chronic tachypnea of infancy
Reactive lymphoid lesions
Related to alv. surf. region
Related to lung vessels/heart
Related to systemic disease
Unclear RDS in mature neonate
Oneway Anova
p = 0.0040
Dunn's post test
p < 0.05
p < 0.01
p < 0.001

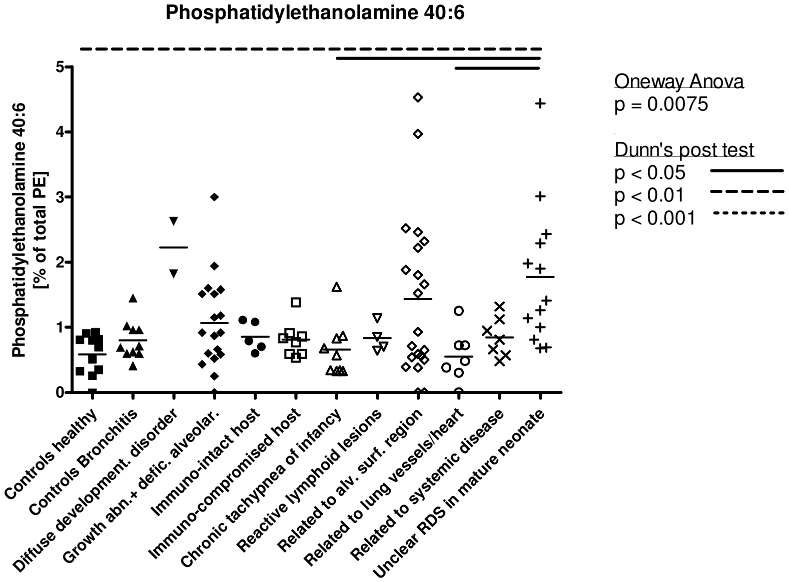
Phosphatidylethanolamine 40:6
Phosphatidylethanolamine 40:6
[% of total PE]
Controls healthy
Controls Bronchitis
Diffuse development. disorder
Growth abn.+ defic. alveolar.
Immuno-intact host
Immuno-compromised host
Chronic tachypnea of infancy
Reactive lymphoid lesions
Related to alv. surf. region
Related to lung vessels/heart
Related to systemic disease
Unclear RDS in mature neonate
Oneway Anova
p = 0.0075
Dunn's post test
p < 0.05
p < 0.01
p < 0.001

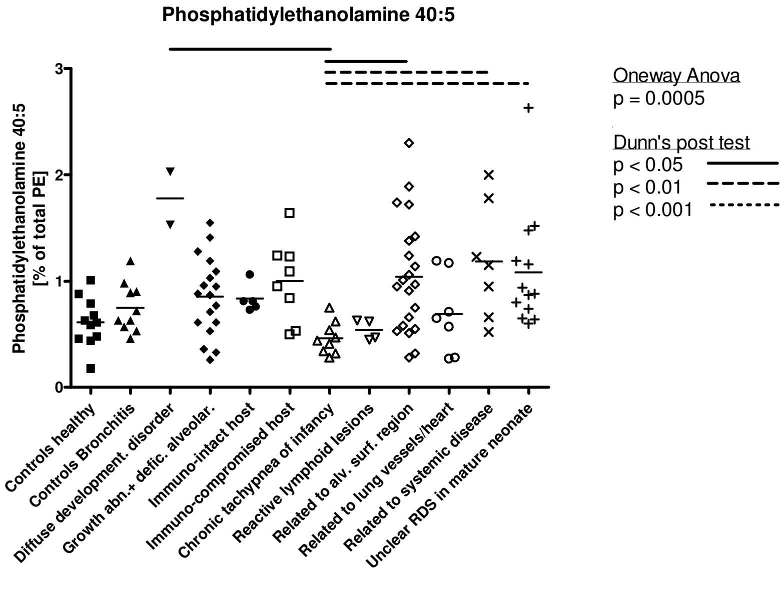
Phosphatidylethanolamine 40:5
Phosphatidylethanolamine 40:5
[% of total PE]
Controls healthy
Controls Bronchitis
Diffuse development. disorder
Growth abn.+ defic. alveolar.
Immuno-intact host
Immuno-compromised host
Chronic tachypnea of infancy
Reactive lymphoid lesions
Related to alv. surf. region
Related to lung vessels/heart
Related to systemic disease
Unclear RDS in mature neonate
Oneway Anova
p = 0.0005
Dunn's post test
p < 0.05
p < 0.01
p < 0.001

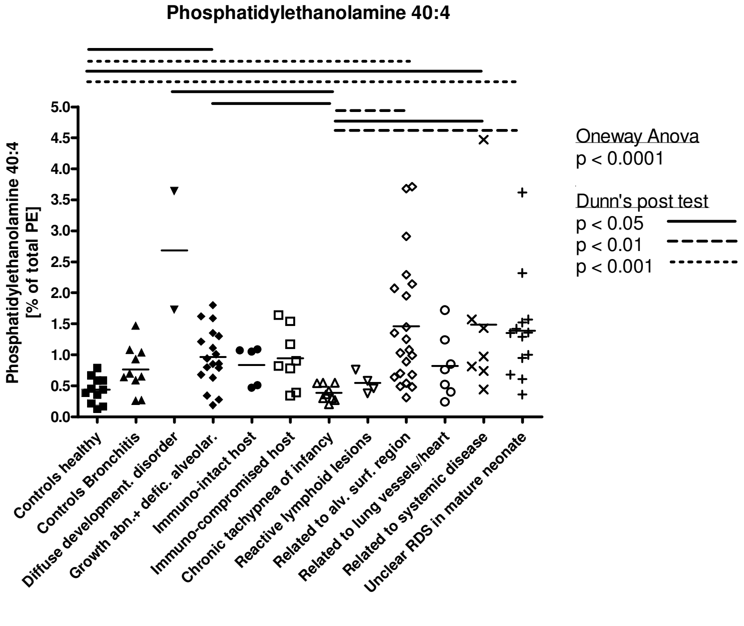
Phosphatidylethanolamine 40:4
Phosphatidylethanolamine 40:4
[% of total PE]
Controls healthy
Controls Bronchitis
Diffuse development. disorder
Growth abn.+ defic. alveolar.
Immuno-intact host
Immuno-compromised host
Chronic tachypnea of infancy
Reactive lymphoid lesions
Related to alv. surf. region
Related to lung vessels/heart
Related to systemic disease
Unclear RDS in mature neonate
Oneway Anova
p < 0.0001
Dunn's post test
p < 0.05
p < 0.01
p < 0.001

Plasmalogens

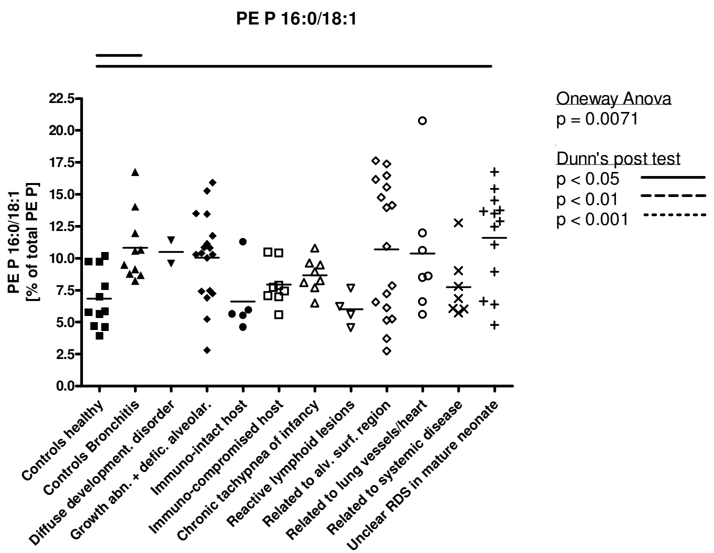

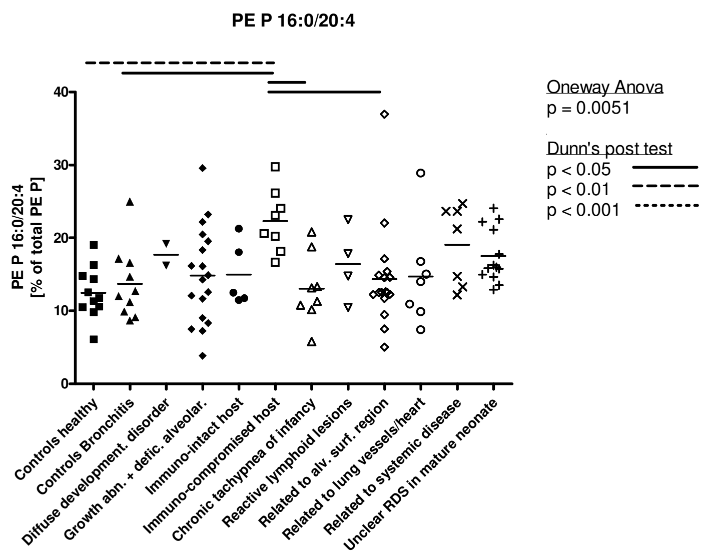

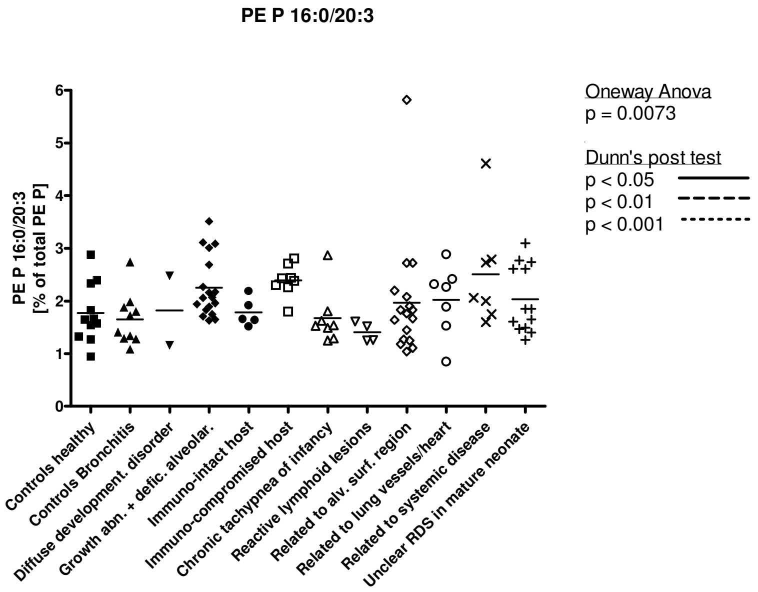

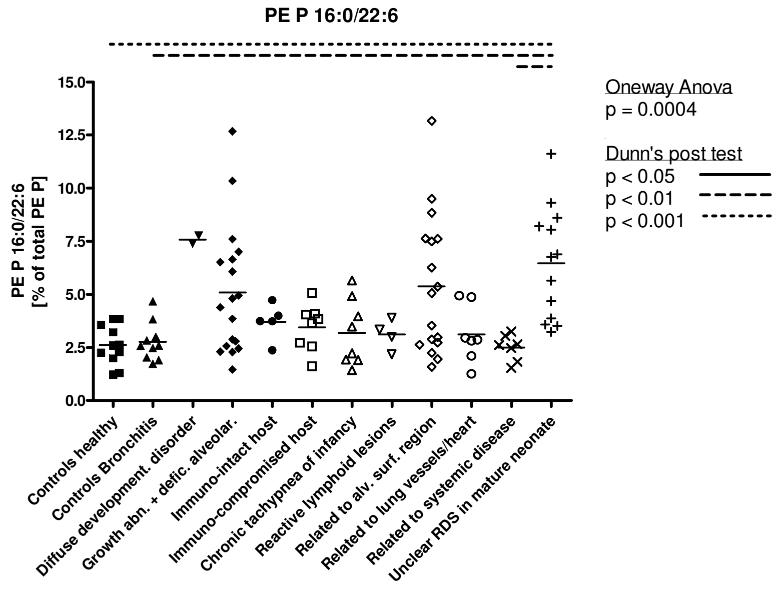

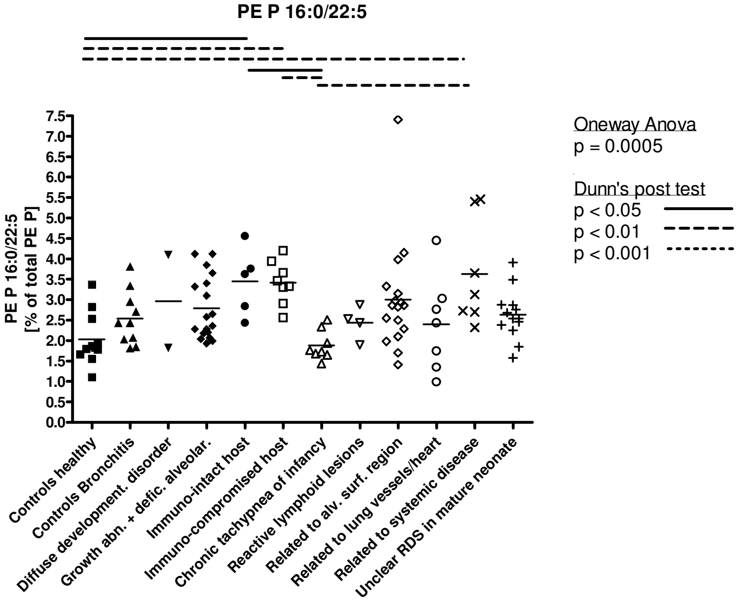
PE P 16:0/22:5
PE P 16:0/22:5
[% of total PE P]
Controls healthy
Controls Bronchitis
Diffuse development. disorder
Growth abn. + defic. alveolar.
Immuno-intact host
Immuno-compromised host
Chronic tachypnea of infancy
Reactive lymphoid lesions
Related to alv. surf. region
Related to lung vessels/heart
Related to systemic disease
Unclear RDS in mature neonate
Oneway Anova
p = 0.0005
Dunn's post test
p < 0.05
p < 0.01
p < 0.001

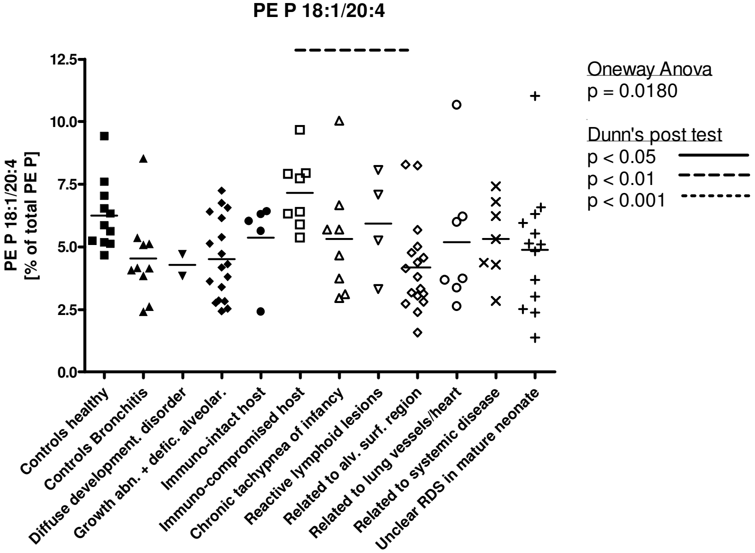
PE P 18:1/20:4
PE P 18:1/20:4
[% of total PE P]
Controls healthy
Controls Bronchitis
Diffuse development. disorder
Growth abn. + defic. alveolar.
Immuno-intact host
Immuno-compromised host
Chronic tachypnea of infancy
Reactive lymphoid lesions
Related to alv. surf. region
Related to lung vessels/heart
Related to systemic disease
Unclear RDS in mature neonate
Oneway Anova
p = 0.0180
Dunn's post test
p < 0.05
p < 0.01
p < 0.001

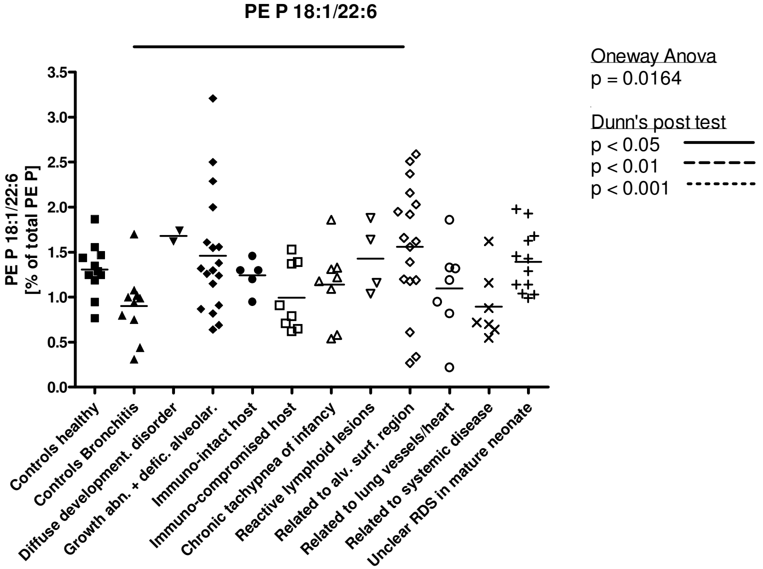
PE P 18:1/22:6
PE P 18:1/22:6
[% of total PE P]
Controls healthy
Controls Bronchitis
Diffuse development. disorder
Growth abn. + defic. alveolar.
Immuno-intact host
Immuno-compromised host
Chronic tachypnea of infancy
Reactive lymphoid lesions
Related to alv. surf. region
Related to lung vessels/heart
Related to systemic disease
Unclear RDS in mature neonate
Oneway Anova
p = 0.0164
Dunn's post test
p < 0.05
p < 0.01
p < 0.001

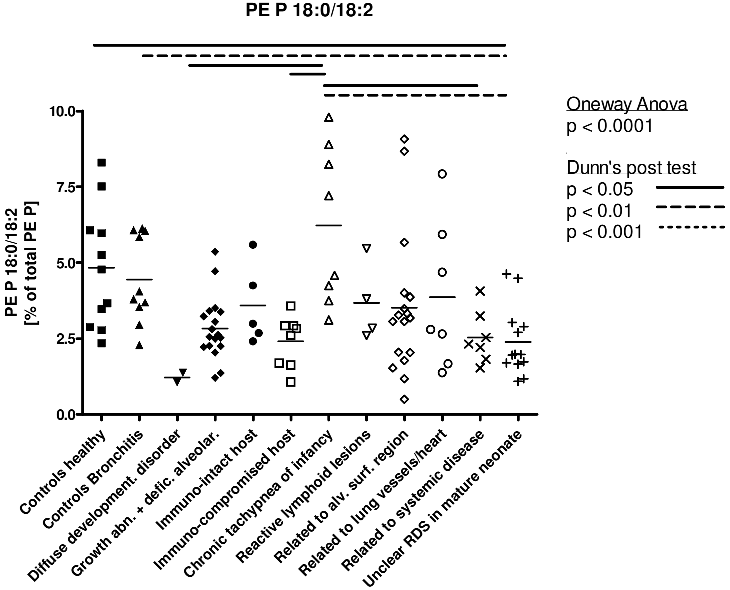
PE P 18:0/18:2
PE P 18:0/18:2
[% of total PE P]
Controls healthy
Controls Bronchitis
Diffuse development. disorder
Growth abn. + defic. alveolar.
Immuno-intact host
Immuno-compromised host
Chronic tachypnea of infancy
Reactive lymphoid lesions
Related to alv. surf. region
Related to lung vessels/heart
Related to systemic disease
Unclear RDS in mature neonate
Oneway Anova
p < 0.0001
Dunn's post test
p < 0.05
p < 0.01
p < 0.001

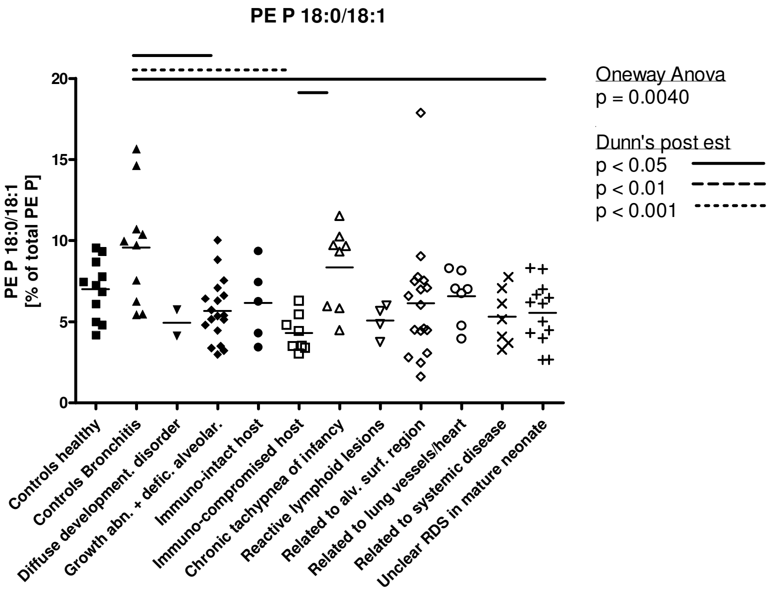

PE P 18:0/18:1
PE P 18:0/18:1
[% of total PE P]
0
5
10
15
20
Controls healthy
Controls Bronchitis
Diffuse development. disorder
Growth abn. + defic. alveolar.
Immuno-intact host
Immuno-compromised host
Chronic tachypnea of infancy
Reactive lymphoid lesions
Related to alv. surf. region
Related to lung vessels/heart
Related to systemic disease
Unclear RDS in mature neonate
Oneway Anova
p = 0.0040
Dunn's post est
p < 0.05
p < 0.01
p < 0.001

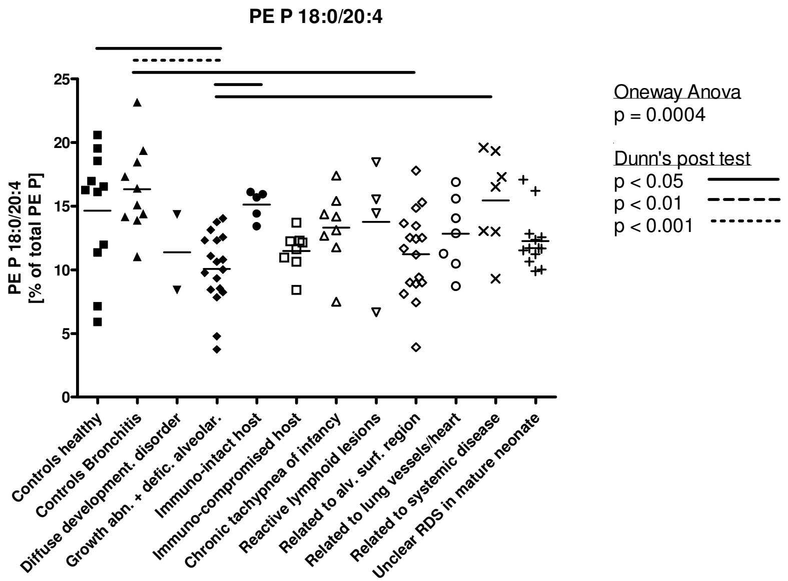

PE P 18:0/20:4
PE P 18:0/20:4
[% of total PE P]
0
5
10
15
20
25
Controls healthy
Controls Bronchitis
Diffuse development. disorder
Growth abn. + defic. alveolar.
Immuno-intact host
Immuno-compromised host
Chronic tachypnea of infancy
Reactive lymphoid lesions
Related to alv. surf. region
Related to lung vessels/heart
Related to systemic disease
Unclear RDS in mature neonate
Oneway Anova
p = 0.0004
Dunn's post test
p < 0.05
p < 0.01
p < 0.001

Phosphatidylserine

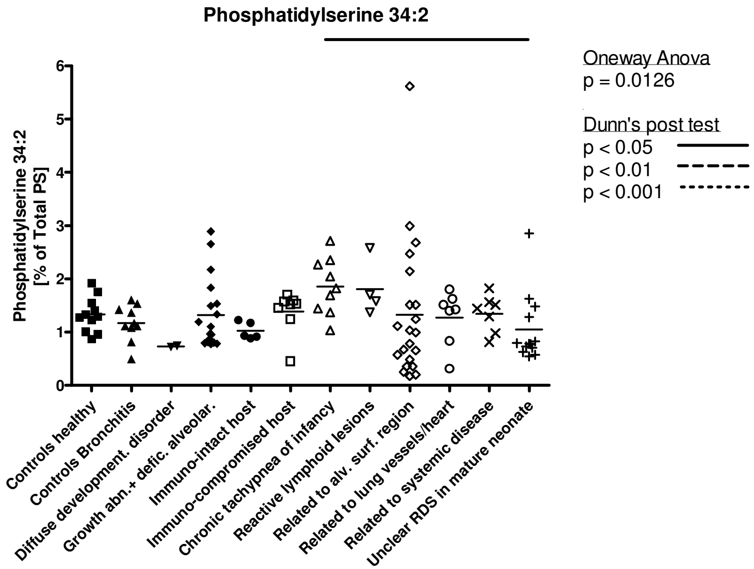

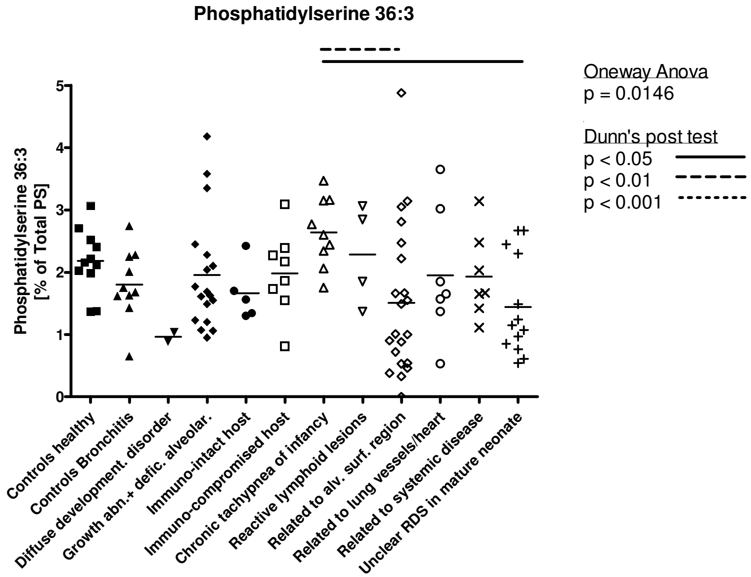

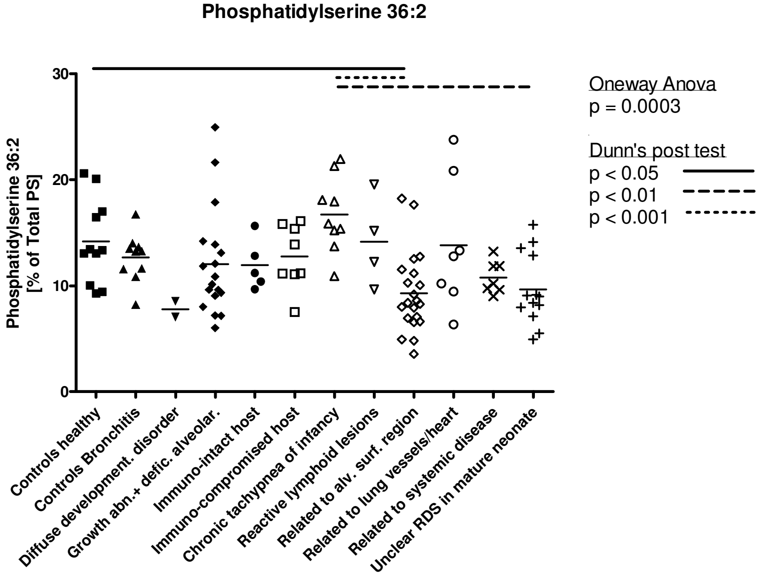

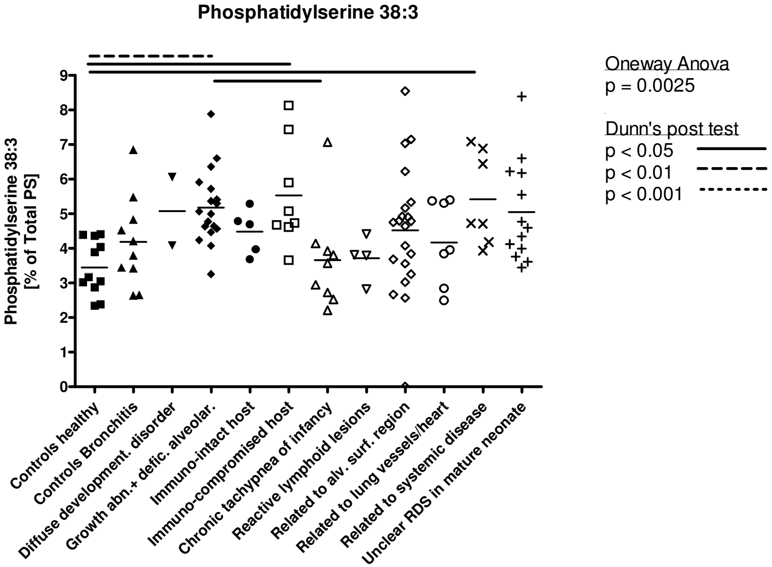

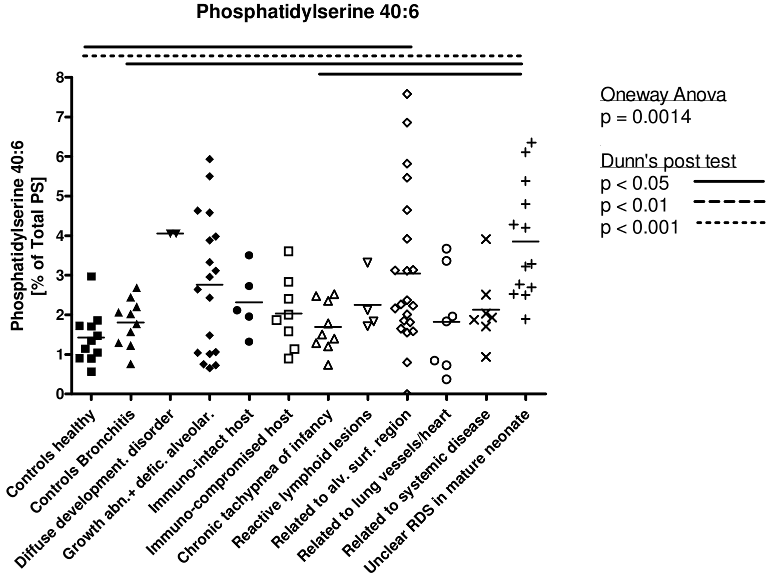
Phosphatidylserine 40:6
Phosphatidylserine 40:6 [% of Total PS]
Oneway Anova
p = 0.0014
Dunn's post test
p < 0.05
p < 0.01
p < 0.001
Controls healthy
Controls Bronchitis
Diffuse development. disorder
Growth abn.+ defic. alveolar.
Immuno-intact host
Immuno-compromised host
Chronic tachypnea of infancy
Reactive lymphoid lesions
Related to alv. surf. region
Related to lung vessels/heart
Related to systemic disease
Unclear RDS in mature neonate

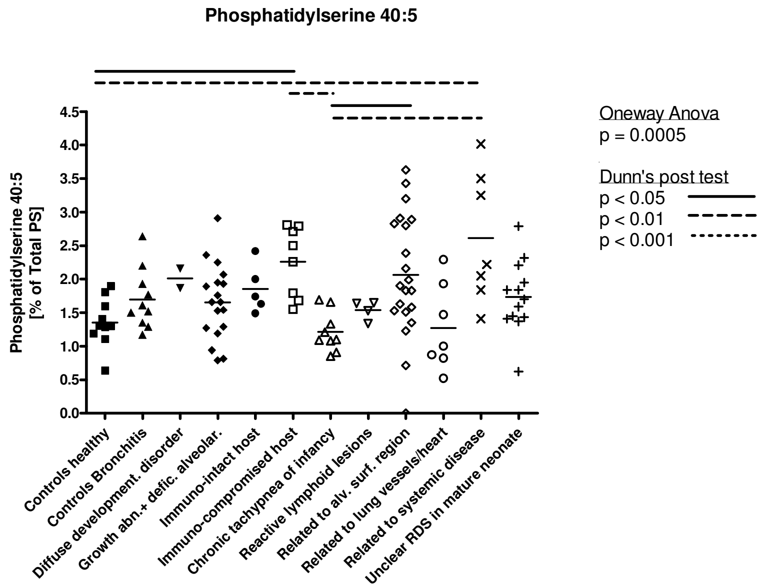
Phosphatidylserine 40:5
Phosphatidylserine 40:5 [% of Total PS]
Oneway Anova
p = 0.0005
Dunn's post test
p < 0.05
p < 0.01
p < 0.001
Controls healthy
Controls Bronchitis
Diffuse development. disorder
Growth abn.+ defic. alveolar.
Immuno-intact host
Immuno-compromised host
Chronic tachypnea of infancy
Reactive lymphoid lesions
Related to alv. surf. region
Related to lung vessels/heart
Related to systemic disease
Unclear RDS in mature neonate

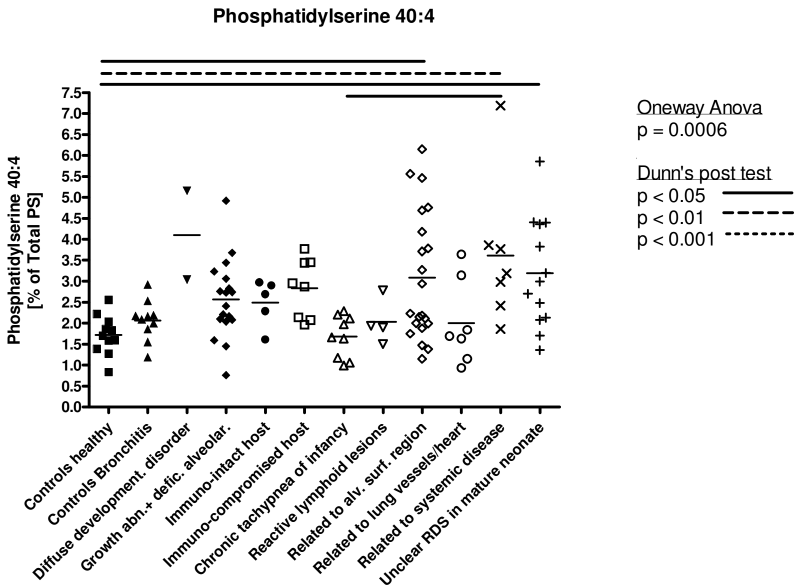
Phosphatidylserine 40:4
Phosphatidylserine 40:4 [% of Total PS]
Oneway Anova
p = 0.0006
Dunn's post test
p < 0.05
p < 0.01
p < 0.001
Controls healthy
Controls Bronchitis
Diffuse development. disorder
Growth abn.+ defic. alveolar.
Immuno-intact host
Immuno-compromised host
Chronic tachypnea of infancy
Reactive lymphoid lesions
Related to alv. surf. region
Related to lung vessels/heart
Related to systemic disease
Unclear RDS in mature neonate

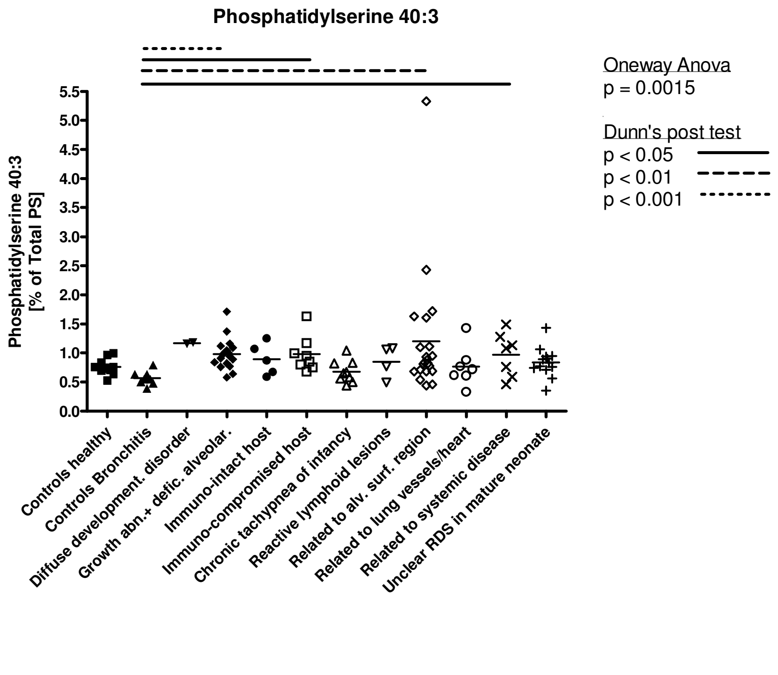
Phosphatidylserine 40:3
Phosphatidylserine 40:3 [% of Total PS]
Oneway Anova
p = 0.0015
Dunn's post test
p < 0.05
p < 0.01
p < 0.001
Controls healthy
Controls Bronchitis
Diffuse development. disorder
Growth abn.+ defic. alveolar.
Immuno-intact host
Immuno-compromised host
Chronic tachypnea of infancy
Reactive lymphoid lesions
Related to alv. surf. region
Related to lung vessels/heart
Related to systemic disease
Unclear RDS in mature neonate

Phosphatidylglycerol

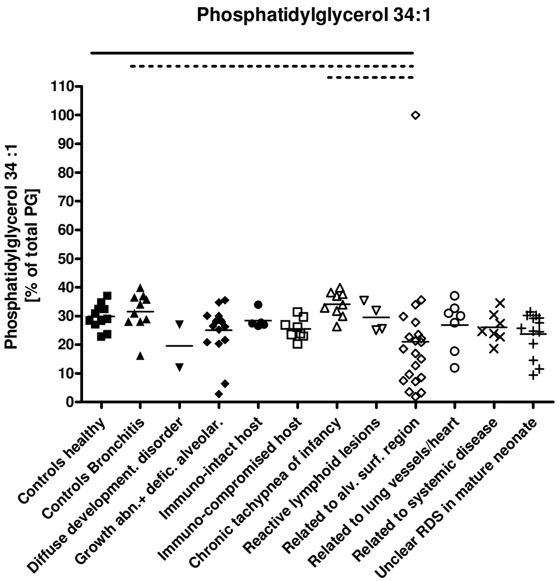

Oneway Anova
$p = 0.0004$

Dunn's post test
$p < 0.05$ ———
$p < 0.01$ – – –
$p < 0.001$ - - - -

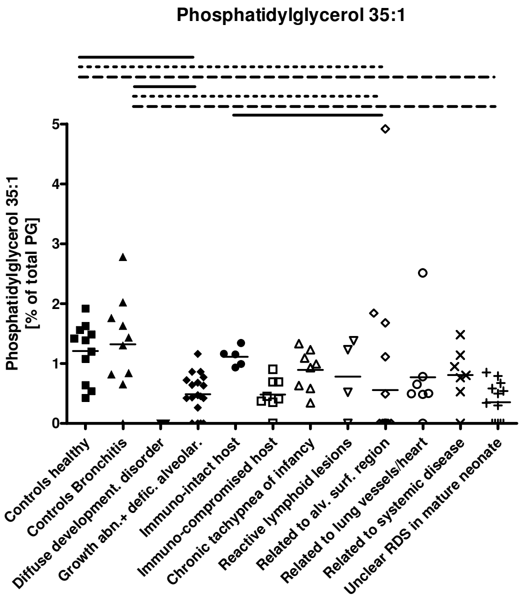

Oneway Anova
$p < 0.0001$

Dunn's post test
$p < 0.05$ ———
$p < 0.01$ – – –
$p < 0.001$ - - - -

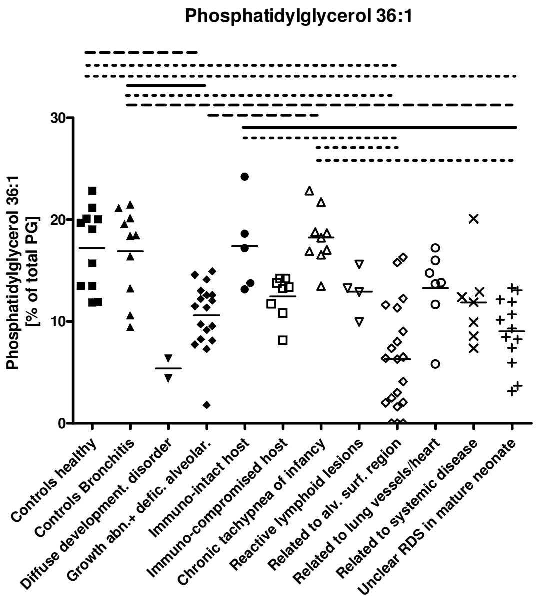

Oneway Anova
$p < 0.0001$

Dunn's post test
$p < 0.05$ ———
$p < 0.01$ – – –
$p < 0.001$ - - - -

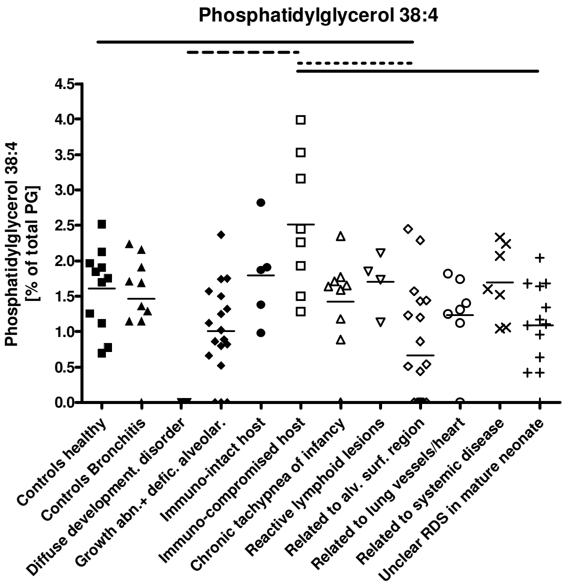

Oneway Anova
$p < 0.0001$

Dunn's post test
$p < 0.05$ ———
$p < 0.01$ – – –
$p < 0.001$ - - - -

Lysophosphadidylcholine

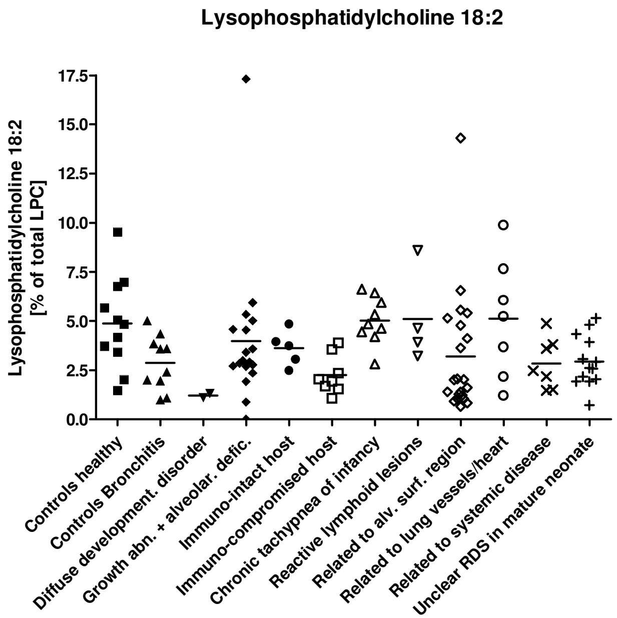

Oneway Anova
$p = 0.0036$

Dunn's post test
$p < 0.05$
$p < 0.01$
$p < 0.001$

Ceramide

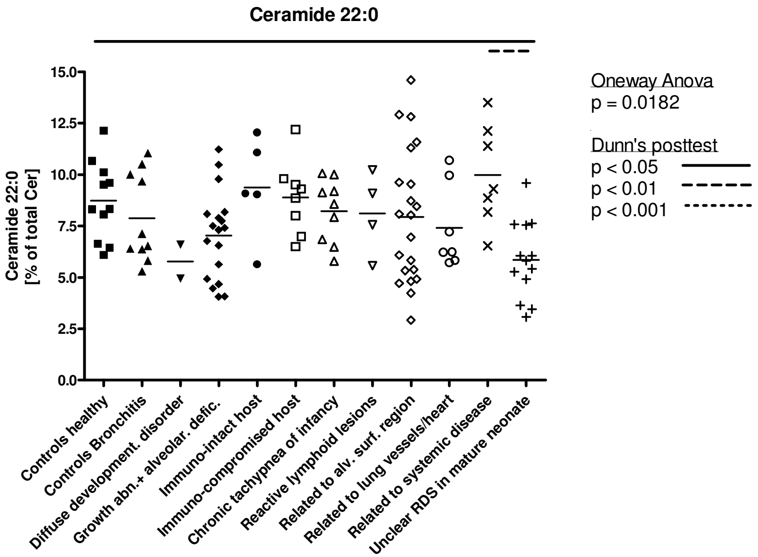
Ceramide 22:0
Ceramide 22:0
[% of total Cer]
15.0
12.5
10.0
7.5
5.0
2.5
0.0
Controls healthy
Controls Bronchitis
Diffuse development. disorder
Growth abn.+ alveolar. defic.
Immuno-intact host
Immuno-compromised host
Chronic tachypnea of infancy
Reactive lymphoid lesions
Related to alv. surf. region
Related to lung vessels/heart
Related to systemic disease
Unclear RDS in mature neonate
Oneway Anova
p = 0.0182
Dunn's posttest
p < 0.05
p < 0.01
p < 0.001

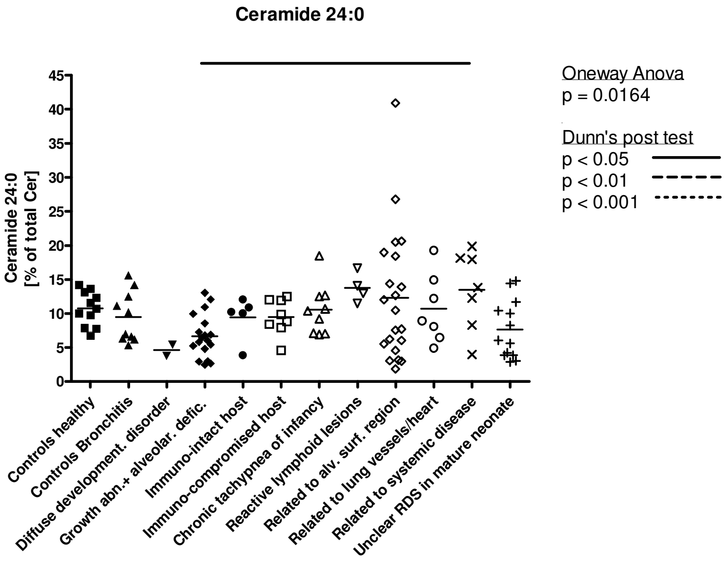
Ceramide 24:0
Ceramide 24:0
[% of total Cer]
45
40
35
30
25
20
15
10
5
0
Controls healthy
Controls Bronchitis
Diffuse development. disorder
Growth abn.+ alveolar. defic.
Immuno-intact host
Immuno-compromised host
Chronic tachypnea of infancy
Reactive lymphoid lesions
Related to alv. surf. region
Related to lung vessels/heart
Related to systemic disease
Unclear RDS in mature neonate
Oneway Anova
p = 0.0164
Dunn's post test
p < 0.05
p < 0.01
p < 0.001

Cholesteryl Ester

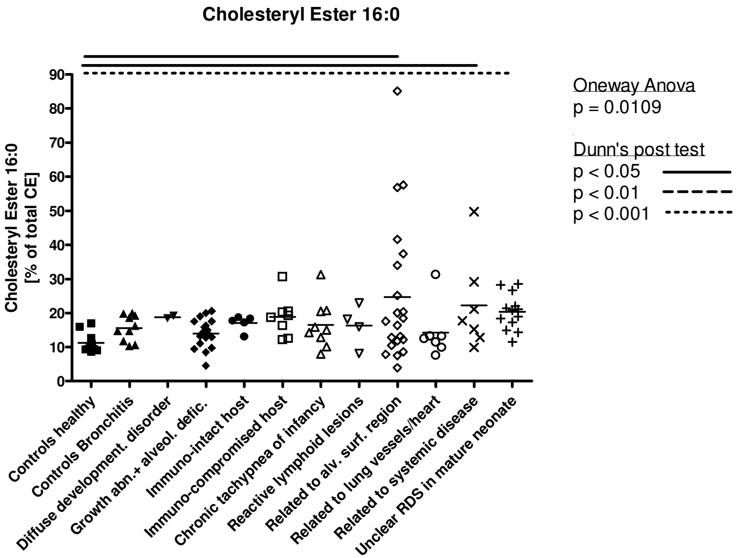

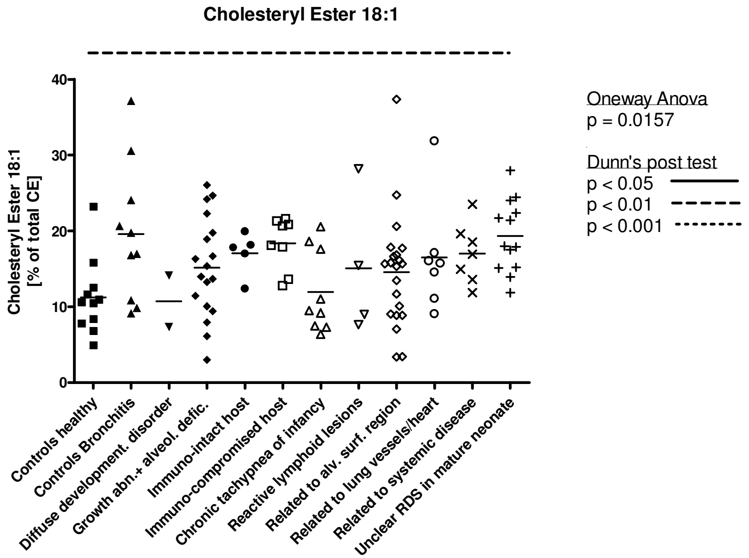

Supplement: S3 Fig — All lipid classes and their species composition are given as individual results of all patients included in the study according the disease category they belong to. Phosphatidylcholine species annotation was based on the assumption of even numbered carbon chains only. Other glycerophospholipid species were annotated based on the assumption that diacyl species are present. SM species annotation is based on the assumption that a sphingoid base with two hydroxyl groups is present. The statistical comparisons were done by ANOVA and Dunn’s post hoc test; all significant results are displayed in each figure. The significance of ANOVA was determined by comparison of the raw P value given with the one calculated to take multiple comparisons into consideration, (P<0.0182 instead of P<0.05). The significant results of Dunn’s post hoc tests are indicated as following: ____ = P < 0.05, _ _ _ = P < 0.01, and …. = P < 0.001. (PDF) [file pone.0117985.s003.pdf]
