# Supplementary figures and images for: Surfactant Lipidomics in Healthy Children and Childhood Interstitial Lung Disease
Source: PLoS One. 2015 Feb 18;10(2):e0117985. doi: 10.1371/journal.pone.0117985 (PMC4333572; doi:10.1371/journal.pone.0117985)

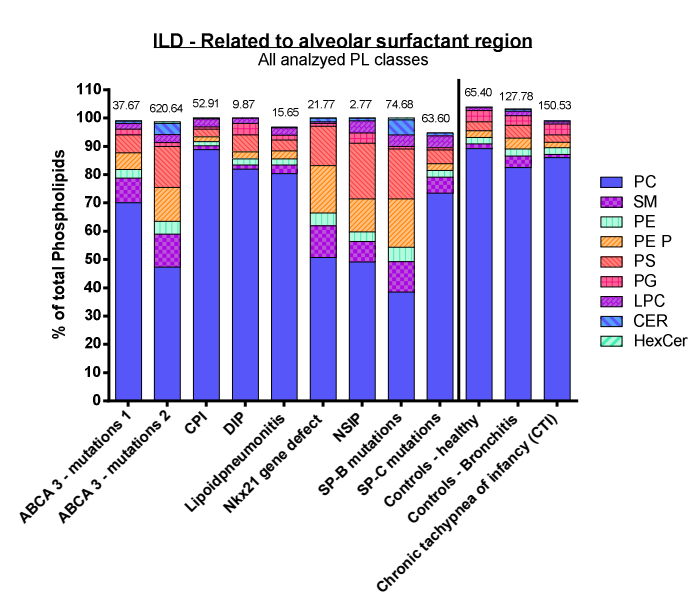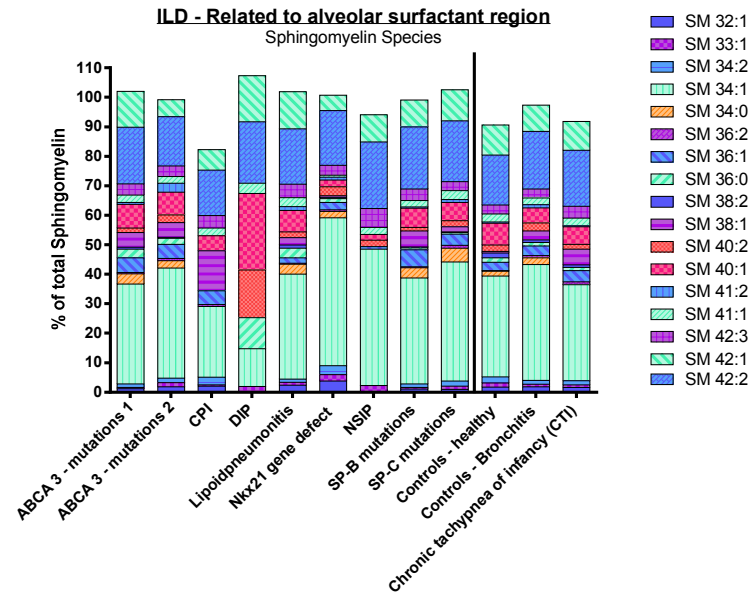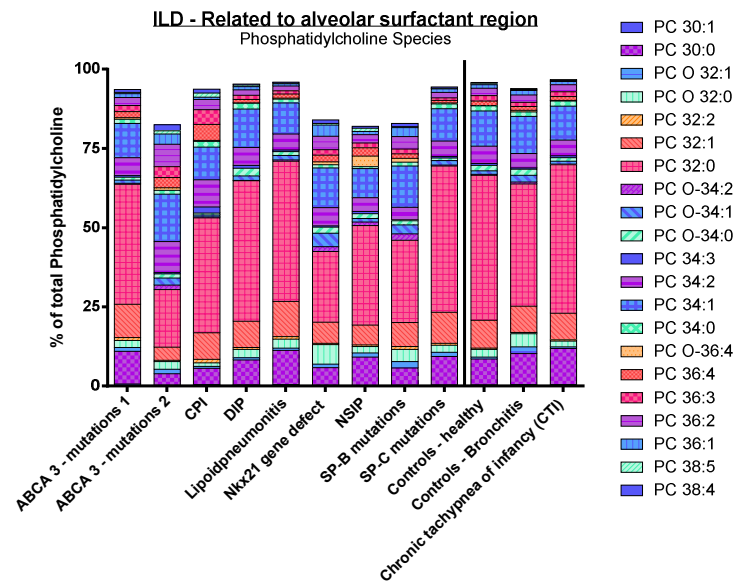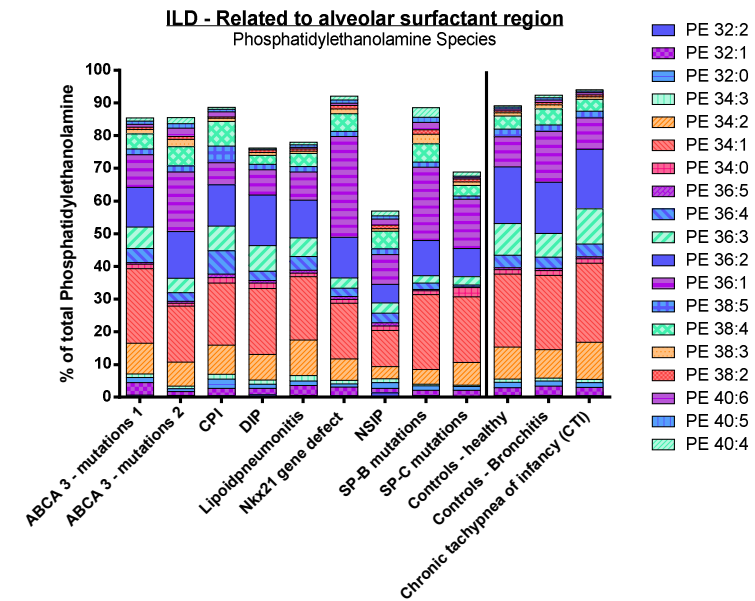

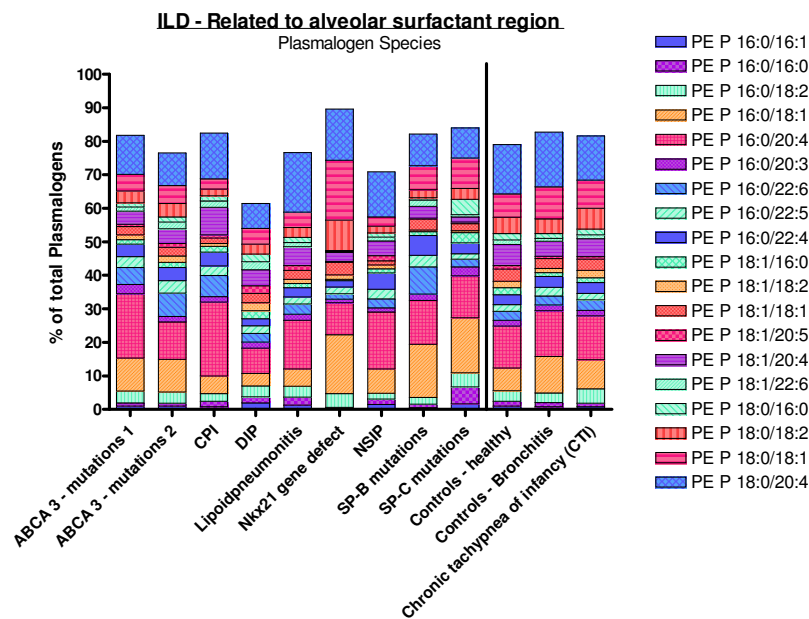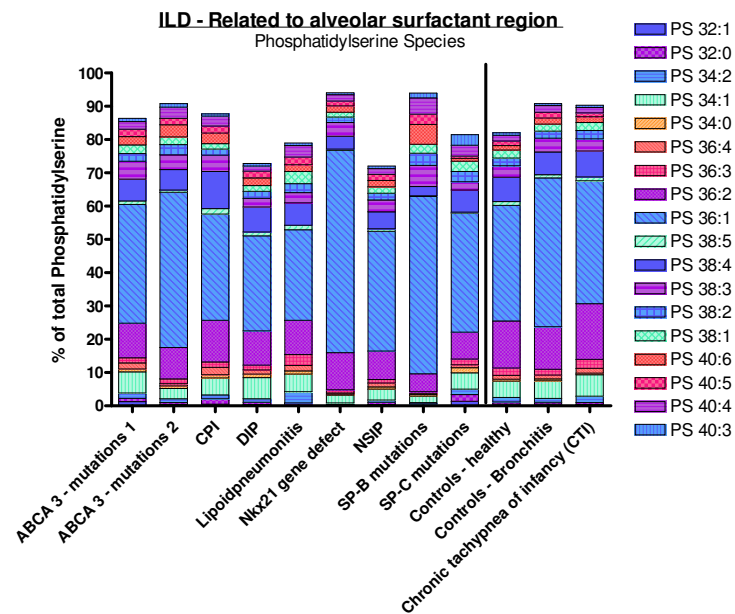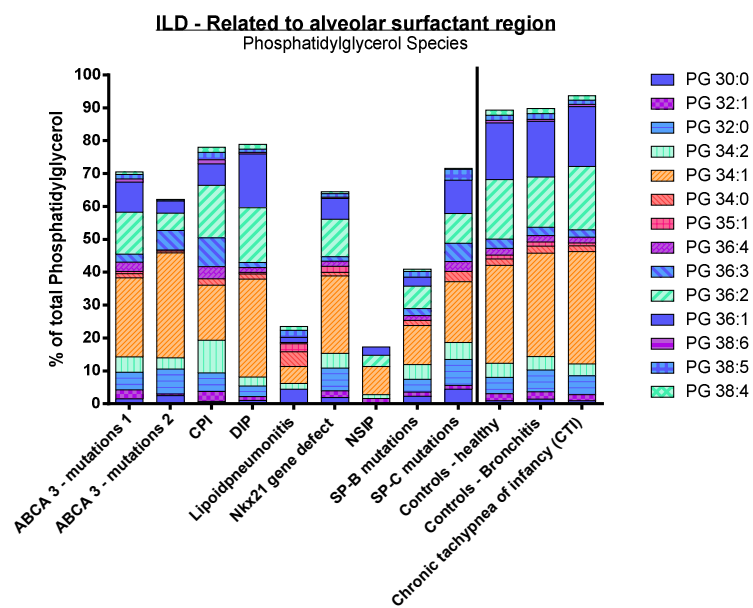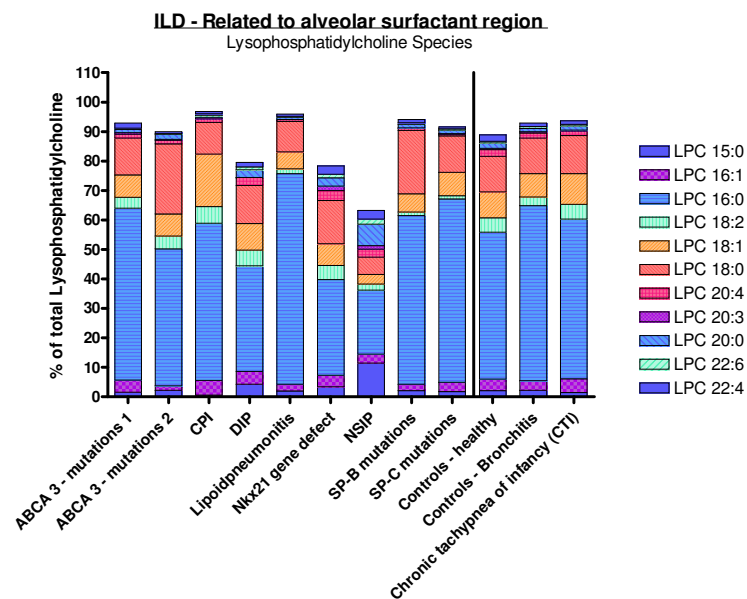

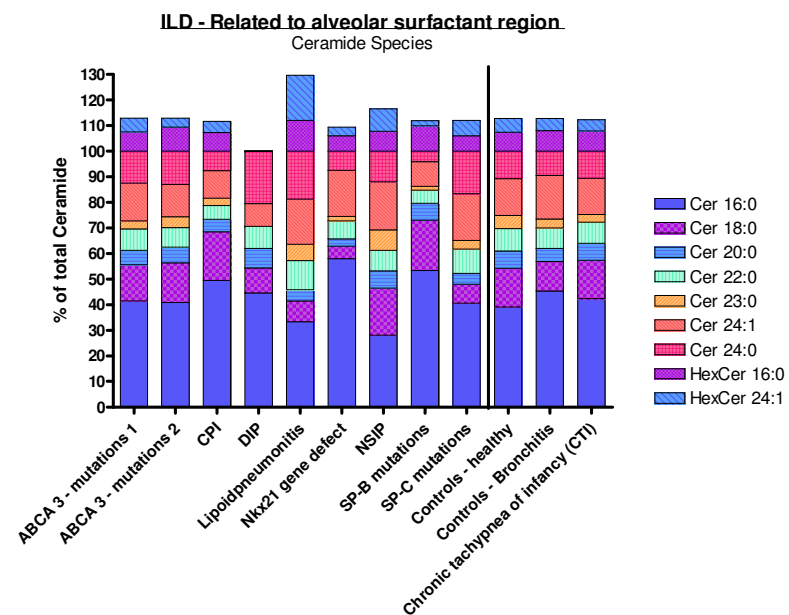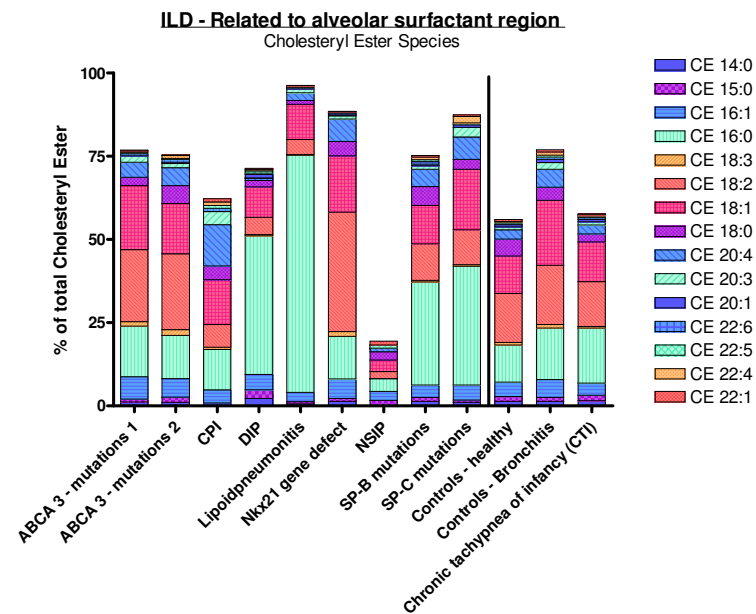

Supplement: S4 Fig — Phospholipid class composition and all species compositions of all classes assessed are indicated as means for each diagnosis. The number of subjects per group is detailed in Table 1. The numbers above the columns indicate the phospholipid concentration analysed (μmol/l). No statistical comparisons were done. Deviations from 100% are the result minor lipid species present at an abundance of < 0.5%; these were included in the calculations, but were not displayed in the graphs. (PDF) [file pone.0117985.s004.pdf]
