# Supplementary material for: Surfactant Lipidomics in Healthy Children and Childhood Interstitial Lung Disease
Source: PLoS One. 2015 Feb 18;10(2):e0117985. doi: 10.1371/journal.pone.0117985 (PMC4333572; doi:10.1371/journal.pone.0117985)
Supplement: S5 Fig — Phospholipid class composition and all species compositions of all classes assessed are indicated as means for each diagnosis. The number of subjects per group is detailed in Table 1. The numbers above the columns indicate the phospholipid concentration analysed (μmol/l). No statistical comparisons were done. Deviations from 100% are the result minor lipid species present at an abundance of < 0.5%; these were included in the calcualtions, but were not displayed in the graphs. (PDF) [file pone.0117985.s005.pdf]

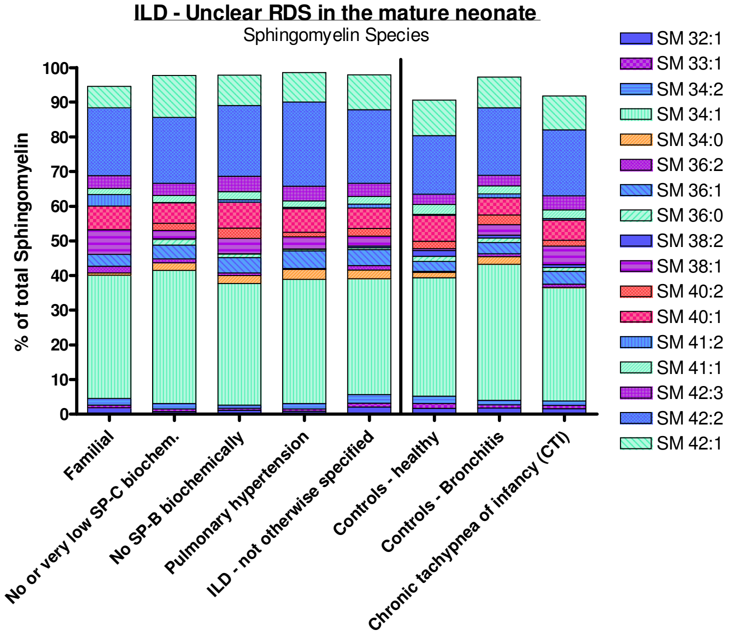
ILD - Unclear RDS in the mature neonate
Sphingomyelin Species
% of total Sphingomyelin
0
10
20
30
40
50
60
70
80
90
100
Familial
No or very low SP-C biochem.
No SP-B biochemically
Pulmonary hypertension
ILD - not otherwise specified
Controls - healthy
Controls - Bronchitis
Chronic tachypnea of infancy (CTI)
SM 32:1
SM 33:1
SM 34:2
SM 34:1
SM 34:0
SM 36:2
SM 36:1
SM 36:0
SM 38:2
SM 38:1
SM 40:2
SM 40:1
SM 41:2
SM 41:1
SM 42:3
SM 42:2
SM 42:1

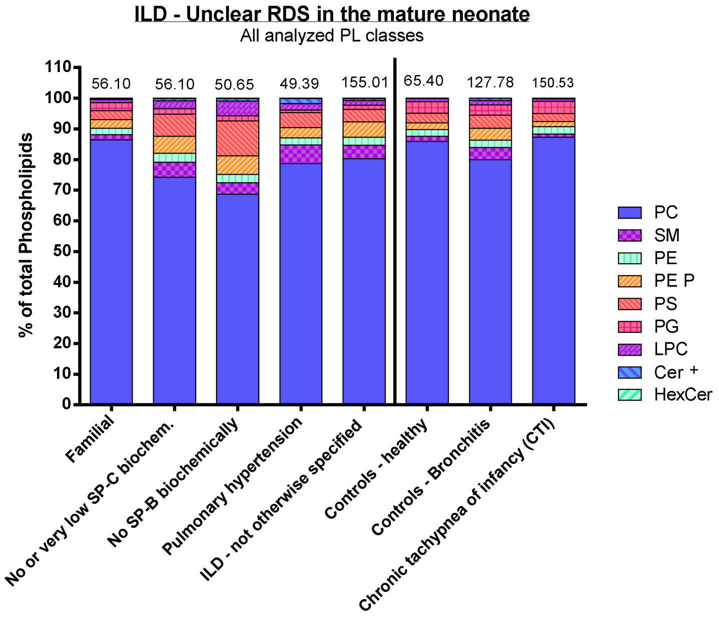
ILD - Unclear RDS in the mature neonate
All analyzed PL classes
% of total Phospholipids
0
10
20
30
40
50
60
70
80
90
100
110
56.10
56.10
50.65
49.39
155.01
65.40
127.78
150.53
Familial
No or very low SP-C biochem.
No SP-B biochemically
Pulmonary hypertension
ILD - not otherwise specified
Controls - healthy
Controls - Bronchitis
Chronic tachypnea of infancy (CTI)
PC
SM
PE
PE P
PS
PG
LPC
Cer +
HexCer

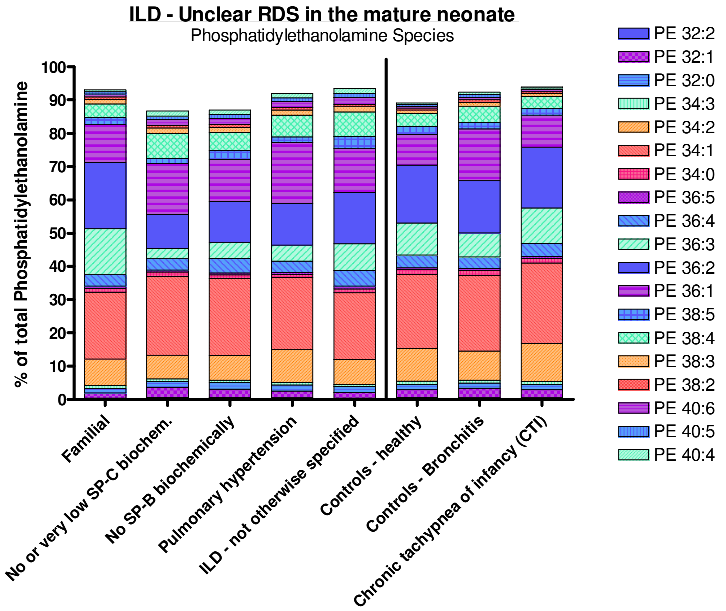
ILD - Unclear RDS in the mature neonate
Phosphatidylethanolamine Species
% of total Phosphatidylethanolamine
0
10
20
30
40
50
60
70
80
90
100
Familial
No or very low SP-C biochem.
No SP-B biochemically
Pulmonary hypertension
ILD - not otherwise specified
Controls - healthy
Controls - Bronchitis
Chronic tachypnea of infancy (CTI)
PE 32:2
PE 32:1
PE 32:0
PE 34:3
PE 34:2
PE 34:1
PE 34:0
PE 36:5
PE 36:4
PE 36:3
PE 36:2
PE 36:1
PE 38:5
PE 38:4
PE 38:3
PE 38:2
PE 40:6
PE 40:5
PE 40:4

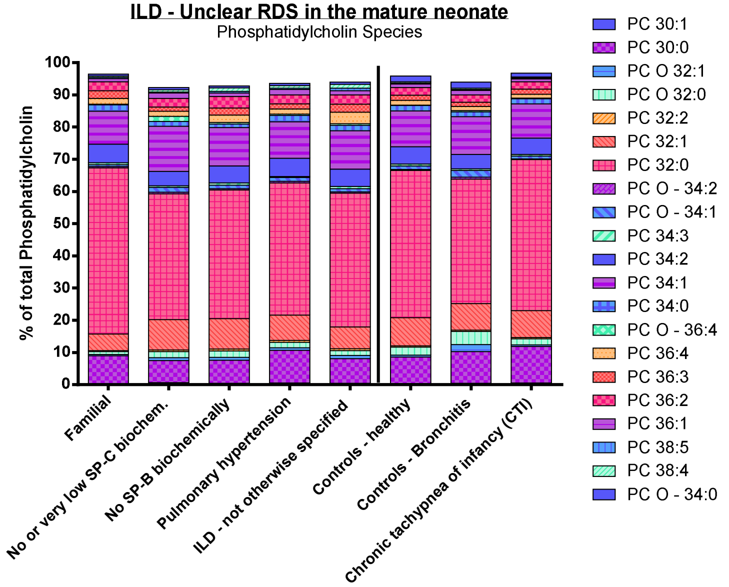
ILD - Unclear RDS in the mature neonate
Phosphatidylcholin Species
% of total Phosphatidylcholin
0
10
20
30
40
50
60
70
80
90
100
Familial
No or very low SP-C biochem.
No SP-B biochemically
Pulmonary hypertension
ILD - not otherwise specified
Controls - healthy
Controls - Bronchitis
Chronic tachypnea of infancy (CTI)
PC 30:1
PC 30:0
PC O 32:1
PC O 32:0
PC 32:2
PC 32:1
PC 32:0
PC O - 34:2
PC O - 34:1
PC 34:3
PC 34:2
PC 34:1
PC 34:0
PC O - 36:4
PC 36:4
PC 36:3
PC 36:2
PC 36:1
PC 38:5
PC 38:4
PC O - 34:0

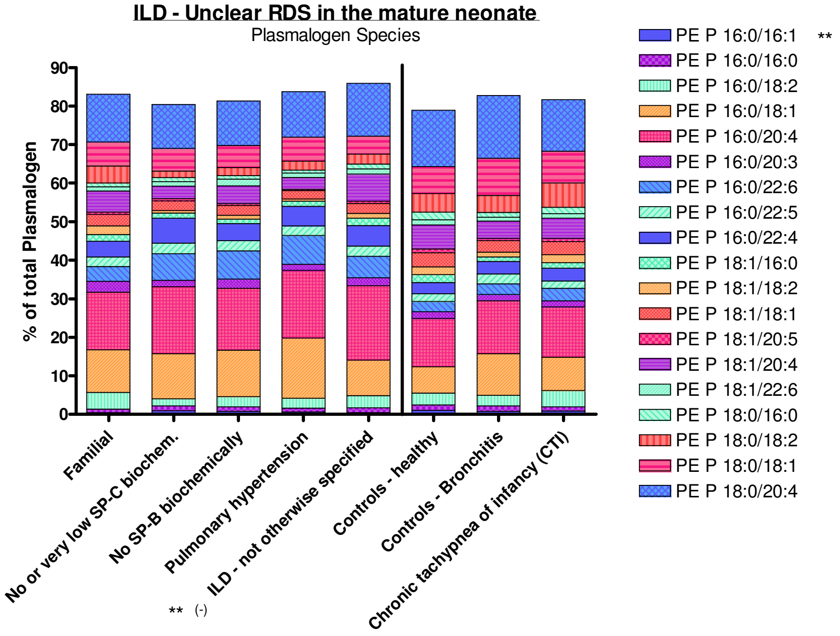
ILD - Unclear RDS in the mature neonate
Plasmalogen Species
% of total Plasmalogen
0
10
20
30
40
50
60
70
80
90
Familial
No or very low SP-C biochem.
No SP-B biochemically
Pulmonary hypertension
ILD - not otherwise specified
Controls - healthy
Controls - Bronchitis
Chronic tachypnea of infancy (CTI)
** (-)
PE P 16:0/16:1 **
PE P 16:0/16:0
PE P 16:0/18:2
PE P 16:0/18:1
PE P 16:0/20:4
PE P 16:0/20:3
PE P 16:0/22:6
PE P 16:0/22:5
PE P 16:0/22:4
PE P 18:1/16:0
PE P 18:1/18:2
PE P 18:1/18:1
PE P 18:1/20:5
PE P 18:1/20:4
PE P 18:1/22:6
PE P 18:0/16:0
PE P 18:0/18:2
PE P 18:0/18:1
PE P 18:0/20:4

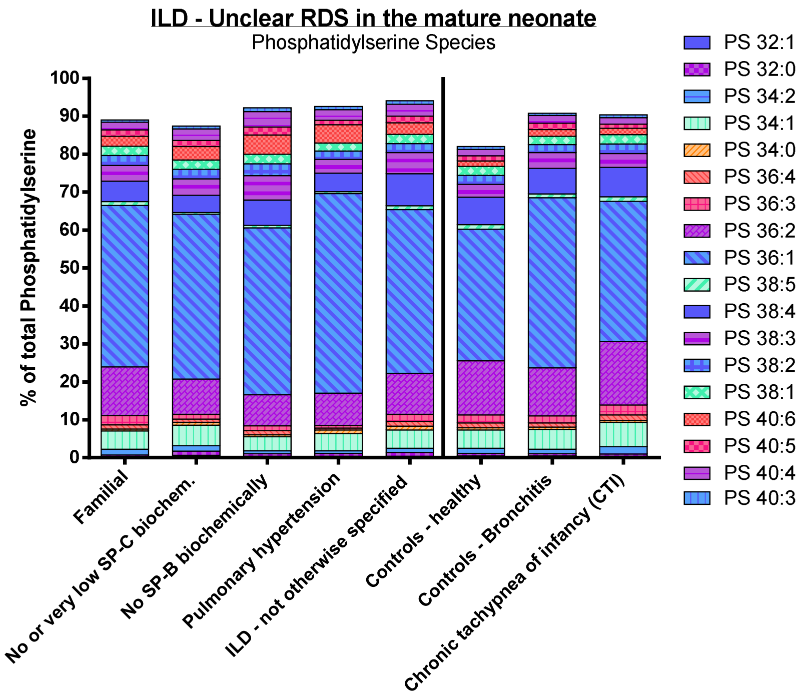
ILD - Unclear RDS in the mature neonate
Phosphatidylserine Species
% of total Phosphatidylserine
0
10
20
30
40
50
60
70
80
90
100
Familial
No or very low SP-C biochem.
No SP-B biochemically
Pulmonary hypertension
ILD - not otherwise specified
Controls - healthy
Controls - Bronchitis
Chronic tachypnea of infancy (CTI)
PS 32:1
PS 32:0
PS 34:2
PS 34:1
PS 34:0
PS 36:4
PS 36:3
PS 36:2
PS 36:1
PS 38:5
PS 38:4
PS 38:3
PS 38:2
PS 38:1
PS 40:6
PS 40:5
PS 40:4
PS 40:3

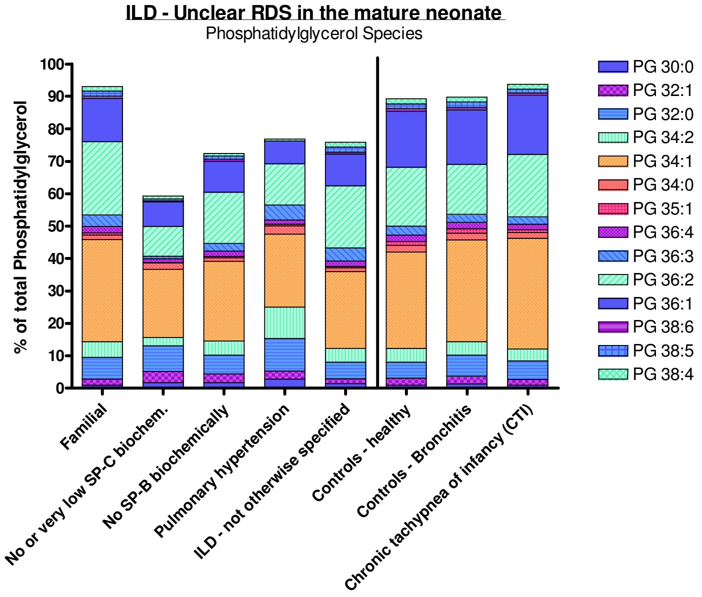
ILD - Unclear RDS in the mature neonate
Phosphatidylglycerol Species
% of total Phosphatidylglycerol
0
10
20
30
40
50
60
70
80
90
100
Familial
No or very low SP-C biochem.
No SP-B biochemically
Pulmonary hypertension
ILD - not otherwise specified
Controls - healthy
Controls - Bronchitis
Chronic tachypnea of infancy (CTI)
PG 30:0
PG 32:1
PG 32:0
PG 34:2
PG 34:1
PG 34:0
PG 35:1
PG 36:4
PG 36:3
PG 36:2
PG 36:1
PG 38:6
PG 38:5
PG 38:4

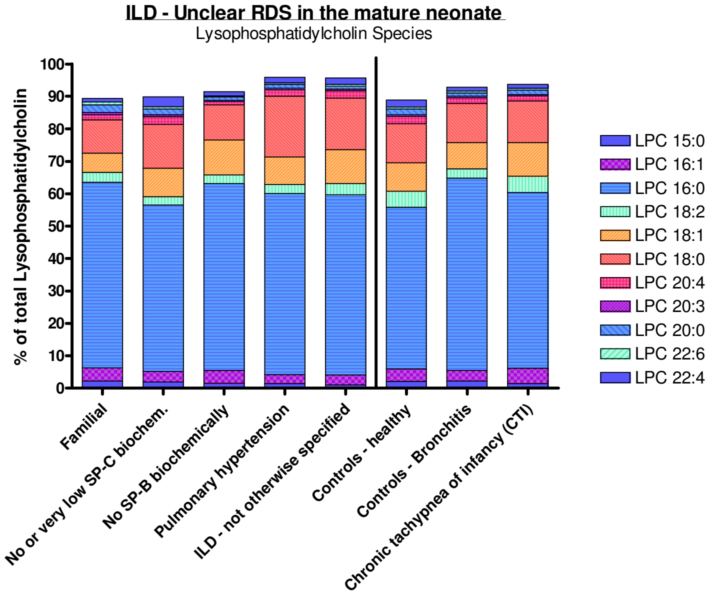
ILD - Unclear RDS in the mature neonate
Lysophosphatidylcholin Species
% of total Lysophosphatidylcholin
0
10
20
30
40
50
60
70
80
90
100
Familial
No or very low SP-C biochem.
No SP-B biochemically
Pulmonary hypertension
ILD - not otherwise specified
Controls - healthy
Controls - Bronchitis
Chronic tachypnea of infancy (CTI)
LPC 15:0
LPC 16:1
LPC 16:0
LPC 18:2
LPC 18:1
LPC 18:0
LPC 20:4
LPC 20:3
LPC 20:0
LPC 22:6
LPC 22:4

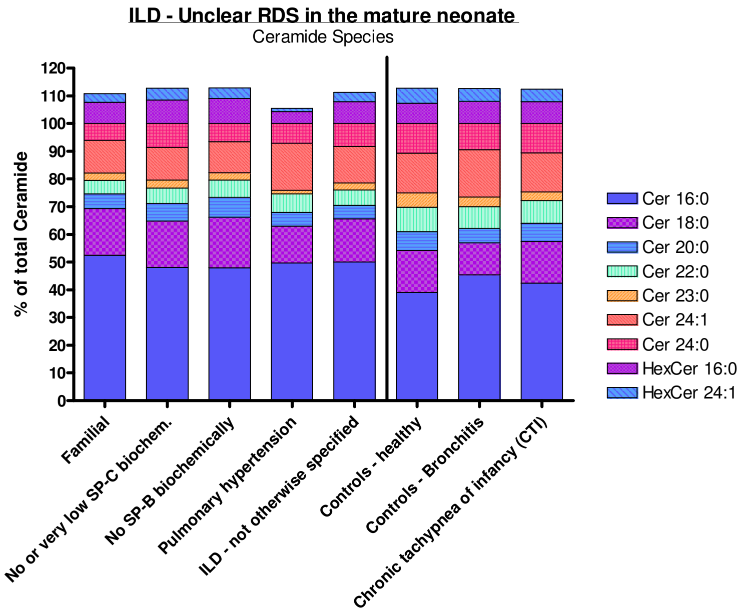
ILD - Unclear RDS in the mature neonate
Ceramide Species
% of total Ceramide
0
10
20
30
40
50
60
70
80
90
100
110
120
Familial
No or very low SP-C biochem.
No SP-B biochemically
Pulmonary hypertension
ILD - not otherwise specified
Controls - healthy
Controls - Bronchitis
Chronic tachypnea of infancy (CTI)
Cer 16:0
Cer 18:0
Cer 20:0
Cer 22:0
Cer 23:0
Cer 24:1
Cer 24:0
HexCer 16:0
HexCer 24:1

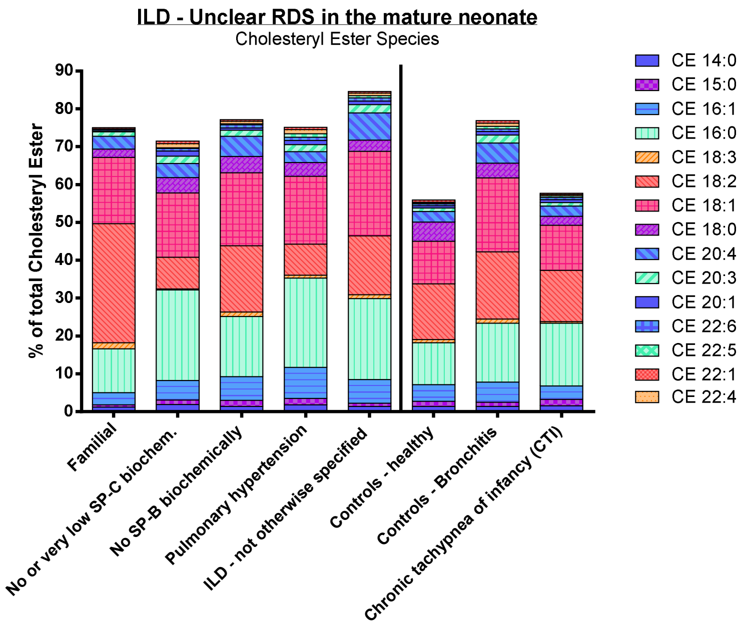
ILD - Unclear RDS in the mature neonate
Cholesteryl Ester Species
% of total Cholesteryl Ester
0
10
20
30
40
50
60
70
80
90
Familial
No or very low SP-C biochem.
No SP-B biochemically
Pulmonary hypertension
ILD - not otherwise specified
Controls - healthy
Controls - Bronchitis
Chronic tachypnea of infancy (CTI)
CE 14:0
CE 15:0
CE 16:1
CE 16:0
CE 18:3
CE 18:2
CE 18:1
CE 18:0
CE 20:4
CE 20:3
CE 20:1
CE 22:6
CE 22:5
CE 22:1
CE 22:4
