# Supplementary material for: Surfactant Lipidomics in Healthy Children and Childhood Interstitial Lung Disease
Source: PLoS One. 2015 Feb 18;10(2):e0117985. doi: 10.1371/journal.pone.0117985 (PMC4333572; doi:10.1371/journal.pone.0117985)
Supplement: S1 Methods — (PDF) [file pone.0117985.s006.pdf]

Online Data Supplement

Surfactant lipidomics in healthy children and childhood interstitial lung disease

Griese M*[1], Kirmeier HG*[1], Liebisch G[2], Rauch D[1], Stückler F[2], Schmitz G[2], Zarbock R[1] and the ILD-BAL working group of the Kids-Lung-Register**

*The two authors contributed equally.

[1]Dr. von Haunersches Kinderspital, University of Munich, Member of the German Center for Lung Research, Lindwurmstr. 4a, D-80337 Munich, Germany

[2]Institute of Computational Biology, Helmholtz Center Munich, Germany

[3]Institute of Clinical Chemistry and Laboratory Medicine, University of Regensburg, Franz-Josef-Strauss-Allee 11, 93053 Regensburg, Germany

Correspondence and reprints:

Prof. Dr. Matthias Griese, Dr. von Haunersches Kinderspital, University of Munich, Lindwurmstraße 4, D-80337 Munich, Germany. Phone: ++49 89 5160 7870, fax: ++49 89 5160 7872;

e-mail: Matthias.griese@med.uni-muenchen.de

METHODS

Patient selection

Between 1997 to 2007 all cases sent to our lab for biochemical and surfactant analysis were carefully diagnosed to have diffuse parenchymal lung disease, i.e. interstitial lung disease, by history, lab findings, radiological (x-ray and CT scan where available) findings, clinical course and sometimes histological and genetic means, as part of the routine work up of these patients. Of these, 111 had chronic (> 4 weeks) diffuse parenchymal lung disease affecting both sides of the lungs by radiology, gave informed consent and had lavage material available for analysis. Children were excluded if they had received therapeutic surfactant application within a 4 week period, as this would interfere with biochemical surfactant analysis. The children were further categorized diagnostically into one of the 10 ILD categories listed in Table 1 and a definite clinical diagnosis was obtained at the highest level possible in each case. Age and duration of their lung disease are indicated in Table 1. These patients were compared to 16 healthy controls (controls-healthy, broncho-alveolar lavage cell differential: 82% macrophages, 5.8% polymorph nuclear leucocytes, 12% lymphocytes, 0.2% eosinophils) and 16 disease controls, i.e. children assessed for chronic cough and exclusion of cystic fibrosis, structural abnormalities, immune deficiencies, allergic airway disease and primary ciliary dyskinesia (controls-bronchitis, broncho-alveolar lavage cell differential: 51.7% macrophages, 28.7% polymorph nuclear leucocytes, 18.8 % lymphocytes, 0.2% eosinophils, 0.3% plasma cells, 0.3% mast cells). The details on diagnosis of each subject are listed in Table S1. Chronic tachypnoea of infancy are children with the typical clinical and computer morphological signs of neuroendocrine cell hyperplasia of infancy, however not all are diagnosed by biopsy. M. Osler denotes hereditary hemorrhagic teleangiectasia. We give a unique patient pseudonym identifier number, so that the subjects may be tracked if used in different publications.

Broncho-alveolar lavages, sample preparation and biochemical analysis

Broncho-alveolar lavages were performed in a standardized manner, using four 1 ml/kg fractions of warmed 0.9% NaCl. The first fraction was kept separate, the other fractions were pooled, centrifuged at 200 g for 10 min, and the supernatant was frozen for lipid analysis. A volume of 150 µl of supernatant was used for samples with a protein concentration below 500 µg/ml, 75 µl between 500 and 1000 µg/ml and 50 µl for samples with protein concentration over 1000 µg/ml. Although tracheal aspirates may be less invasive in ventilated children, these were not considered due to potential differences to standardized lavages. Lavage samples were extracted according to the method of Bligh and Dyer in the presence of not naturally occurring lipid species as internal standards [1]. Crude lipid extracts were quantified by direct flow injection electrospray ionization tandem mass spectrometry (ESI-MS/MS) in positive ion mode using the analytical setup and strategy described previously [2, 3]. A precursor ion of m/z 184 was used for phosphatidylcholine (PC), sphingomyelin (SM)[2] and lysophosphatidylcholine (LPC) [4]. Neutral loss fragments were used for the following lipid classes: Phosphatidylethanolamine (PE) 141, phosphatidylserine (PS) 185, phosphatidylglycerol (PG) 189 [5, 6]. PE-based plasmalogens (PE P) were analyzed according to the principles described by Zemski-Berry (2004)[7]. Sphingosine based ceramides (Cer) and hexosylceramides (HexCer) were analyzed using a fragment ion of m/z 264 [8]. Free cholesterol (FC) and cholesteryl ester (CE) were quantified using a fragment ion of m/z 369 after selective derivatization of FC [3]. Lipid species were annotated according to the recently published proposal for shorthand notation of lipid structures that are derived from mass spectrometry [9]. Glycerophospholipid species annotation was based on the assumption of even numbered carbon chains only. SM species annotation is based on the assumption that a sphingoid base with two hydroxyl groups is present.

Surfactant proteins B and C were determined by Western blotting [10]. The lower limit of detection was 2.5 ng/lane. “No SP-B biochemically” was defined as less than 2.5 ng/lange when 25 mg of lavage protein were applied and “No or very low SP-C biochemically” when less than 5 ng/lane SP-C were detected.

Calculation and expression of lipid results

Results are presented as total lipids, total phospholipids, cholesteryl ester and free cholesterol (all expressed as nmol/ml) (Figure 1). Total lipid concentration was calculated as the sum of all analysed lipid classes including phospholipids, cholesteryl ester and free cholesterol; phospholipid classes were expressed as a percentage by mol of total phospholipids, and the species within a phospholipid species were expressed as the percentage mol of that respective phospholipid class (Figure 1). Species were only taken into consideration for presentation if the species had an abundance of ≥ 0.5 %. To guarantee quality of analysis, diluted bronchoalveolar lavages with a total lipid concentration < 15 µmol/l or a phospholipid concentration < 10 µmol/l, were excluded from calculations, as most lipid species are below or close to the limit of detection. In total 28 of the 143 subjects were excluded from the final analysis, as detailed in Table 1.

References

1. Bligh EG, Dyer WJ: **A rapid method of total lipid extraction and purification**. *Canadian journal of biochemistry and physiology* 1959, **37**(8):911-917.
2. Liebisch G, Lieser B, Rathenberg J, Drobnik W, Schmitz G: **High-throughput quantification of phosphatidylcholine and sphingomyelin by electrospray ionization tandem mass spectrometry coupled with isotope correction algorithm**. *Biochimica et biophysica acta* 2004, **1686**(1-2):108-117.
3. Liebisch G, Binder M, Schifferer R, Langmann T, Schulz B, Schmitz G: **High throughput quantification of cholesterol and cholesteryl ester by electrospray ionization tandem mass spectrometry (ESI-MS/MS)**. *Biochimica et biophysica acta* 2006, **1761**(1):121-128.
4. Liebisch G, Drobnik W, Lieser B, Schmitz G: **High-throughput quantification of lysophosphatidylcholine by electrospray ionization tandem mass spectrometry**. *Clinical chemistry* 2002, **48**(12):2217-2224.
5. Matyash V, Liebisch G, Kurzchalia TV, Shevchenko A, Schwudke D: **Lipid extraction by methyl-tert-butyl ether for high-throughput lipidomics**. *Journal of lipid research* 2008, **49**(5):1137-1146.
6. Brugger B, Erben G, Sandhoff R, Wieland FT, Lehmann WD: **Quantitative analysis of biological membrane lipids at the low picomole level by nano-electrospray ionization tandem mass spectrometry**. *Proceedings of the National Academy of Sciences of the United States of America* 1997, **94**(6):2339-2344.
7. Zemski Berry KA, Murphy RC: **Electrospray ionization tandem mass spectrometry of glycerophosphoethanolamine plasmalogen phospholipids**. *Journal of the American Society for Mass Spectrometry* 2004, **15**(10):1499-1508.
8. Liebisch G, Drobnik W, Reil M, Trumbach B, Arnecke R, Olgemoller B, Roscher A, Schmitz G: **Quantitative measurement of different ceramide species from crude cellular extracts by electrospray ionization tandem mass spectrometry (ESI-MS/MS)**. *Journal of lipid research* 1999, **40**(8):1539-1546.
9. Liebisch G, Vizcaino JA, Kofeler H, Trotzmuller M, Griffiths WJ, Schmitz G, Spener F, Wakelam MJ: **Shorthand notation for lipid structures derived from mass spectrometry**. *Journal of lipid research* 2013, **54**(6):1523-1530.
10. Griese M, Schumacher S, Tredano M, Steinecker M, Braun A, Guttentag S, Beers MF, Bahuau M: **Expression profiles of hydrophobic surfactant proteins in children with diffuse chronic lung disease**. *Respiratory research* 2005, **6**:80.
