# Supplementary material for: Surfactant Lipidomics in Healthy Children and Childhood Interstitial Lung Disease
Source: PLoS One. 2015 Feb 18;10(2):e0117985. doi: 10.1371/journal.pone.0117985 (PMC4333572; doi:10.1371/journal.pone.0117985)
Supplement: S1 Table — All lipid results are indicated. Note that in 7 of the patients PE P and in 2 PG were not measured. Results are indicated in the units above the columns, for the different lipid species, as percent of the lipid class. Phosphatidylcholine species annotation was based on the assumption of even numbered carbon chains only. Other glycerophospholipid species were annotated based on the assumption that diacyl species are present. SM species annotation is based on the assumption that a sphingoid base with two hydroxyl groups is present. (PDF) [file pone.0117985.s007.pdf]

No.	Disease Category	Group No.	Subgroup No.	Disease Subcategory	Age at BAL [years]	Disease Long Term C	ABCA3	SP-B	SP-C
380	Controls healthy	1			6,85	sick-same			
649	Controls healthy	1			11,68	healthy			
566	Controls healthy	1			4,88	sick-worse			
1349	Controls healthy	1			2,07	sick-better			
386	Controls healthy	1			1,08	sick-better			
206	Controls healthy	1			13,23	sick-same			
399	Controls healthy	1			3,93	sick-worse			
725	Controls healthy	1			0,87	sick-better			
560	Controls healthy	1			9,15	sick-better			
436	Controls healthy	1			4,15	sick-same			
435	Controls healthy	1			3,38	sick-better			
357	Controls Bronchitis	2			3,39				
638	Controls Bronchitis	2			0,07	healthy			
727	Controls Bronchitis	2			8,60				
252	Controls Bronchitis	2			0,48	sick-better			
549	Controls Bronchitis	2			10,55				
553	Controls Bronchitis	2			3,75	dead			
1227	Controls Bronchitis	2			1,58				
563	Controls Bronchitis	2			12,70	sick-worse			
378	Controls Bronchitis	2			2,40	sick-same			
344	Controls Bronchitis	2			2,33				
202	ILD-diffuse developmental disorders	3	31	Alveolar capillary dysplasia with misalignment pulmonary vein	0,08	dead			
1178	ILD-diffuse developmental disorders	3	31	Alveolar capillary dysplasia with misalignment pulmonary vein	0,06	dead			
612	ILD-Growth abnormalities deficient alveolarisation	4	41	Intrauterine growth retardation (alcohol)	0,19	sick-same			
550	ILD-Growth abnormalities deficient alveolarisation	4	42	Pulmonary hypoplasia	1,01	sick-better			
639	ILD-Growth abnormalities deficient alveolarisation	4	42	Pulmonary hypoplasia	0,25	sick-better			
818	ILD-Growth abnormalities deficient alveolarisation	4	42	Pulmonary hypoplasia	0,37	sick-better			
495	ILD-Growth abnormalities deficient alveolarisation	4	42	Pulmonary hypoplasia associated with diagphragmatic hernia	0,99	sick-same			
1167	ILD-Growth abnormalities deficient alveolarisation	4	43	Related to chromosomal disorders	0,33	sick-same			
885	ILD-Growth abnormalities deficient alveolarisation	4	43	Related to chromosomal disorders	0,11	sick-better			
256	ILD-Growth abnormalities deficient alveolarisation	4	44	Related to preterm birth (BPD-cLDI)	0,29	dead			
260	ILD-Growth abnormalities deficient alveolarisation	4	44	Related to preterm birth (BPD-cLDI)	0,60	sick-better			
600	ILD-Growth abnormalities deficient alveolarisation	4	44	Related to preterm birth (BPD-cLDI)	0,49	dead			
1299	ILD-Growth abnormalities deficient alveolarisation	4	44	Related to preterm birth (BPD-cLDI)	0,16	sick-better			
653	ILD-Growth abnormalities deficient alveolarisation	4	44	Related to preterm birth (BPD-cLDI)	0,71	sick-same			
313	ILD-Growth abnormalities deficient alveolarisation	4	44	Related to preterm birth (BPD-cLDI)	0,60				
396	ILD-Growth abnormalities deficient alveolarisation	4	44	Related to preterm birth (BPD-cLDI)	0,02	healthy			
1340	ILD-Growth abnormalities deficient alveolarisation	4	44	Related to preterm birth (BPD-cLDI)	0,46	sick-same			
688	ILD-Growth abnormalities deficient alveolarisation	4	44	Related to preterm birth (BPD-cLDI)	0,12	sick-same			
907	ILD-Growth abnormalities deficient alveolarisation	4	44	Related to preterm birth (BPD-cLDI)	0,12	sick-better			
350	ILD-Growth abnormalities deficient alveolarisation	4	44	Related to preterm birth (Wilson Mikity, new BPD)	0,30	sick-better			
1313	ILD-Immune intact host	5	51	Eosinophilic pneumonitis	2,89	sick-better			
1180	ILD-Immune intact host	5	52	Exogen allergic alveolitis/hypersensitivity pneumonitis	9,33	sick-better			
261	ILD-Immune intact host	5	52	Exogen allergic alveolitis/hypersensitivity pneumonitis	14,29	sick-worse			
644	ILD-Immune intact host	5	52	Exogen allergic alveolitis/hypersensitivity pneumonitis	9,50	healthy			
264	ILD-Immune intact host	5	52	Exogen allergic alveolitis/hypersensitivity pneumonitis	10,06	sick-better			
347	ILD-Immuno-compromised host	6	61	Infections–Antibody deficiencies	0,90				
316	ILD-Immuno-compromised host	6	61	Infections–Antibody deficiencies	0,49	healthy			
1323	ILD-Immuno-compromised host	6	62	Infections–Miscellaneous	0,55	sick-better			
235	ILD-Immuno-compromised host	6	63	Infections–Phagocyte defects	7,35	sick-better			
721	ILD-Immuno-compromised host	6	64	Infections–T cell deficiencies	16,26	sick-better			
345	ILD-Immuno-compromised host	6	64	Infections–T cell deficiencies	1,50				
603	ILD-Immuno-compromised host	6	65	Interstitielle Lungenerkrankung	3,45	sick-better			
269	ILD-Immuno-compromised host	6	66	Related to therapeutic intervention	1,62	sick-better			
273	Chronic tachypnoe of infancy (CTI)	7	71	Chronic tachypnoe of infancy (CTI)	1,32	sick-same			
706	Chronic tachypnoe of infancy (CTI)	7	71	Chronic tachypnoe of infancy (CTI)	2,08	sick-better			
674	Chronic tachypnoe of infancy (CTI)	7	71	Chronic tachypnoe of infancy (CTI)	0,41	healthy			
697	Chronic tachypnoe of infancy (CTI)	7	71	Chronic tachypnoe of infancy (CTI)	1,72	healthy			
275	Chronic tachypnoe of infancy (CTI)	7	71	Chronic tachypnoe of infancy (CTI)	0,06	sick-better			
304	Chronic tachypnoe of infancy (CTI)	7	71	Chronic tachypnoe of infancy (CTI)	0,31	sick-same			
544	Chronic tachypnoe of infancy (CTI)	7	71	Chronic tachypnoe of infancy (CTI)	0,52	sick-better			
687	Chronic tachypnoe of infancy (CTI)	7	71	Chronic tachypnoe of infancy (CTI)	0,59	sick-better			
1208	Chronic tachypnoe of infancy (CTI)	7	71	Chronic tachypnoe of infancy (CTI)	0,47	sick-same			
266	ILD-Reactive lymphoid lesions	8	81	Nodular lymphoid hyperplasia of the lung	11,34	sick-same			
487	ILD-Reactive lypmphoid lesions	8	81	Nodular lymphoid hyperplasia of the lung	0,42	sick-same			
548	ILD-Reactive lypmphoid lesions	8	82	Follicular bronchitis/bronchiolits	7,00	sick-worse			
346	ILD-Reactive lypmphoid lesions	8	83	Lymphocytic interstitial pneumonia (LIP)	5,28	sick-worse			
1300	ILD–Related to alveolar surfactant region	9	90	ABCA3 1 Mutation; Related to preterm birth (Wilson Mikity, new BPD)	0,25	sick-better	Arg288Lys	nl	nl
199	ILD–Related to alveolar surfactant region	9	90	ABCA3 mutations 1	0,35	dead	Arg288Lys	nl	nl
719	ILD–Related to alveolar surfactant region	9	90	ABCA3 mutations 1	0,85	healthy	Gly292Val	nl	nl
393	ILD–Related to alveolar surfactant region	9	90	ABCA3 mutations 1	2,90	dead	Glu292Val	nl	nl
541	ILD–Related to alveolar surfactant region	9	90	ABCA3 mutations 1	11,86	dead	Pro770Leu	nl	nl
258	ILD–Related to alveolar surfactant region	9	91	ABCA3 mutations 2	0,19	dead	Arg1561X/Arg1561X	nl	nl
715	ILD–Related to alveolar surfactant region	9	91	ABCA3 mutations 2	0,05	dead	Pro193Ser/Gly1421Arg	nl	nl
253	ILD–Related to alveolar surfactant region	9	91	ABCA3 mutations 2	2,88	dead	Pro32Ser/Gly1314Glu	nl	nl
636	ILD–Related to alveolar surfactant region	9	91	ABCA3 mutations 2	0,03	dead	Phe810fs/Phe810fs	nl	nl
675	ILD–Related to alveolar surfactant region	9	91	ABCA3 mutations 2	0,15	dead	Pro193Arg/Pro193Arg	nl	nl
1326	ILD–Related to alveolar surfactant region	9	92	Chronic pneumonitis of infancy (CPI)	0,04	healthy	nl	nl	nl
68	ILD–Related to alveolar surfactant region	9	93	Desquamative interstitial pneumonia (DIP)	4,34	sick-better	nl	nl	nl
227	ILD–Related to alveolar surfactant region	9	94	Lipoidpneumonitis, Cholesterol pneumonia	8,86	dead	Pro585Pro	nl	nl
594	ILD–Related to alveolar surfactant region	9	94	Lipoidpneumonitis, Cholesterol pneumonia	6,88	sick-better	nl	nl	nl
659	ILD–Related to alveolar surfactant region	9	95	Nkx21 gene defect	19,68		n.d.	nl	nl
507	ILD–Related to alveolar surfactant region	9	96	Nonspecific interstitial pneumonia (NSIP)	10,28	healthy	nl	nl	nl
451	ILD–Related to alveolar surfactant region	9	98	Surfactant protein B mutations	0,33	dead	n.d.	c.1553delT/122delT	n.d.
454	ILD–Related to alveolar surfactant region	9	98	Surfactant protein B mutations	0,11	dead	n.d.	c.121ins2 homocygous	nl
641	ILD–Related to alveolar surfactant region	9	98	Surfactant protein B mutations	0,03	dead	n.d.	c.673-1248del2959	
336	ILD–Related to alveolar surfactant region	9	99	Surfactant protein C mutations	3,00	sick-same	n.d.	nl	Ile73Thr
240	ILD–Related to alveolar surfactant region	9	99	Surfactant protein C mutations	0,71	sick-same	n.d.	nl	Ile73Thr
571	ILD-Related to lung vessels/heart	10	101	Congestive changes related to cardiac dysfunction	0,16	sick-same			
490	ILD-Related to lung vessels/heart	10	101	Congestive changes related to cardiac dysfunction	9,72	sick-same			
429	ILD-Related to lung vessels/heart	10	102	Lymphatic disorders	0,12	dead			
267	ILD-Related to lung vessels/heart	10	103	M. Osler	15,71	sick-same			
306	ILD-Related to lung vessels/heart	10	104	Pulmonary capillary hemangiomatosis	2,01	sick-better			
707	ILD-Related to lung vessels/heart	10	105	Pulmonary hypertension	0,41	dead			
207	ILD-Related to lung vessels/heart	10	105	Pulmonary hypertension	2,19	sick-better			
1318	ILD-Related to systemic diseases	11	111	Alagille Syndrome (arteriohepatic dysplasia)	1,05	dead			
691	ILD-Related to systemic diseases	11	113	Hoyeral Hreidasson Syndrom (Dyskeratosis congenita)	4,04				
494	ILD-Related to systemic diseases	11	114	Idiopathic pulmonary hemosiderosis	8,63	dead			
502	ILD-Related to systemic diseases	11	114	Idiopathic pulmonary hemosiderosis	15,45	healthy			
651	ILD-Related to systemic diseases	11	115	Immune-mediated/collagen vascular disorders	4,49	sick-better			
552	ILD-Related to systemic diseases	11	115	Immune-mediated/collagen vascular disorders	8,34	sick-better			
666	ILD-Related to systemic diseases	11	116	Sarcoidosis	5,67	sick-better			
713	ILD-unclear RDS in the mature neonate	12	121	Interstitial lung disease	0,07	sick-better			
692	ILD-unclear RDS in the mature neonate	12	121	Interstitial lung disease	0,12				
210	ILD-unclear RDS in the mature neonate	12	121	Interstitial lung disease	0,00	sick-better			
650	ILD-unclear RDS in the mature neonate	12	121	Interstitial lung disease	0,08	dead			
460	ILD-unclear RDS in the mature neonate	12	122	Familial	0,12	sick-better			
717	ILD-unclear RDS in the mature neonate	12	123	No or very low SP-C biochemically	0,16	sick-better			
1174	ILD-unclear RDS in the mature neonate	12	123	No or very low SP-C biochemically	0,01	sick-better			
395	ILD-unclear RDS in the mature neonate	12	123	No or very low SP-C biochemically	0,15	dead			
455	ILD-unclear RDS in the mature neonate	12	124	No SP-B biochemically	1,00				
472	ILD-unclear RDS in the mature neonate	12	124	No SP-B biochemically	0,10	healthy			
303	ILD-unclear RDS in the mature neonate	12	124	No SP-B biochemically;Pulmonary hypertension	0,03	sick-better			
197	ILD-unclear RDS in the mature neonate	12	125	Pulmonary Hypertension	0,03	sick-same			
723	ILD-unclear RDS in the mature neonate	12	125	Pulmonary hypertension	0,02	sick-better			

No.	Group No.	Total Lipids [nmol/ml]	Total Phospholipids [nmol/ml]	Free Cholesterol [nmol/ml]	Cholesteryl Ester [nmol/ml]	Total Phospholipids [% of analyzed Lip.]	PC [% of analyzed Lip.]	SM [% of analyzed Lip.]	PE [% of analyzed Lip.]	PE P [% of analyzed Lip.]	PS [% of analyzed Lip.]]	PG [% of analyzed Lip.]	LPC [% of analyzed Lip.]	Cer [% of analyzed Lip.]	HexCer [% of analyzed Lip.]	CE [% of analyzed Lip.]	Free Cholesterol [% of analyzed Lip.]	PC [% of analyzed PL	SM [% of analyzed PL	PE [% of analyzed PL	PE P [% of analyzed PL	PS [% of analyzed PL	PG [% of analyzed PL	LPC [% of analyzed PL	Cer [% of analyzed PL	HexCer [% of analyzed PL]
380	1	30,29	24,02	4,7	1,6	79,32%	64,7%	2,6%	2,0%	3,0%	2,9%	2,5%	1,35%	0,21%	0,02%	5,17%	15,51%	81,61%	3,30%	2,46%	3,76%	3,71%	3,17%	1,70%	0,27%	0,03%
649	1	60,88	54,03	6,1	0,8	88,74%	77,8%	0,6%	2,1%	1,2%	1,9%	4,2%	0,74%	0,07%	0,01%	1,27%	9,98%	87,68%	0,71%	2,41%	1,37%	2,18%	4,71%	0,84%	0,08%	0,01%
566	1	455,65	415,19	38,5	2,0	91,12%	83,0%	0,6%	1,4%	0,7%	1,3%	2,9%	1,17%	0,05%	0,01%	0,43%	8,45%	91,10%	0,61%	1,49%	0,76%	1,47%	3,21%	1,28%	0,06%	0,01%
1349	1	50,25	44,88	4,7	0,7	89,32%	77,7%	0,8%	2,0%	1,3%	2,9%	3,9%	0,64%	0,06%	0,01%	1,30%	9,38%	86,95%	0,92%	2,28%	1,51%	3,20%	4,35%	0,71%	0,07%	0,01%
386	1	17,42	15,00	1,9	0,5	86,11%	72,9%	1,2%	2,1%	2,1%	3,2%	3,8%	0,70%	0,15%	0,02%	3,03%	10,86%	84,70%	1,36%	2,39%	2,48%	3,69%	4,37%	0,81%	0,18%	0,02%
206	1	34,33	28,82	4,9	0,6	83,95%	72,6%	1,4%	1,9%	2,0%	2,8%	2,7%	0,53%	0,12%	0,01%	1,68%	14,37%	86,51%	1,64%	2,21%	2,33%	3,31%	3,21%	0,64%	0,14%	0,02%
399	1	26,47	22,31	3,4	0,8	84,29%	72,1%	1,4%	2,0%	2,1%	2,6%	2,9%	1,04%	0,12%	0,02%	2,97%	12,74%	85,53%	1,61%	2,36%	2,52%	3,11%	3,47%	1,23%	0,15%	0,02%
725	1	16,19	13,46	1,9	0,8	83,17%	68,8%	2,1%	1,7%	2,6%	3,0%	3,3%	1,28%	0,31%	0,05%	4,94%	11,89%	82,70%	2,51%	2,06%	3,18%	3,61%	3,97%	1,54%	0,37%	0,06%
560	1	26,90	22,82	3,6	0,5	84,85%	72,6%	1,5%	1,8%	2,0%	2,9%	3,3%	0,60%	0,13%	0,01%	1,94%	13,21%	85,53%	1,80%	2,16%	2,33%	3,40%	3,90%	0,71%	0,15%	0,02%
436	1	47,79	41,97	4,9	0,9	87,83%	77,1%	0,8%	1,7%	1,6%	2,3%	3,2%	0,89%	0,16%	0,01%	1,88%	10,29%	87,82%	0,94%	1,92%	1,80%	2,65%	3,66%	1,02%	0,18%	0,02%
435	1	42,46	36,85	4,7	0,9	86,78%	73,6%	1,7%	2,2%	2,3%	3,1%	2,8%	0,84%	0,21%	0,02%	2,16%	11,06%	84,86%	1,94%	2,48%	2,65%	3,60%	3,24%	0,96%	0,24%	0,02%
357	2	42,39	36,71	5,1	0,6	86,61%	73,6%	1,1%	2,3%	2,0%	2,7%	3,8%	0,87%	0,16%	0,02%	1,45%	11,94%	84,99%	1,32%	2,61%	2,35%	3,12%	4,38%	1,01%	0,19%	0,02%
638	2	723,58	614,63	98,0	11,0	84,94%	73,5%	0,8%	1,6%	2,1%	2,2%	2,4%	2,20%	0,20%	0,02%	1,52%	13,54%	86,51%	0,95%	1,88%	2,46%	2,56%	2,78%	2,59%	0,23%	0,03%
727	2	26,30	21,86	3,7	0,7	83,13%	69,5%	1,5%	2,1%	2,8%	3,1%	3,0%	0,79%	0,28%	0,03%	2,65%	14,21%	83,57%	1,75%	2,58%	3,40%	3,78%	3,60%	0,95%	0,33%	0,03%
252	2	56,76	42,27	10,6	3,9	74,47%	58,7%	2,3%	2,0%	3,5%	3,5%	3,0%	1,11%	0,32%	0,04%	6,92%	18,61%	78,84%	3,15%	2,64%	4,65%	4,67%	4,08%	1,49%	0,43%	0,06%
549	2	73,71	37,41	28,6	7,7	50,76%	29,7%	6,8%	0,9%	4,1%	4,9%	1,4%	1,05%	1,63%	0,21%	10,49%	38,75%	58,50%	13,33%	1,87%	8,16%	9,71%	2,76%	2,07%	3,20%	0,41%
553	2	74,28	58,81	13,7	1,8	79,17%	60,9%	2,9%	2,5%	4,8%	4,2%	2,5%	0,72%	0,51%	0,06%	2,43%	18,40%	76,98%	3,69%	3,17%	6,08%	5,27%	3,16%	0,91%	0,65%	0,08%
1227	2	135,69	117,31	15,6	2,8	86,45%	73,9%	1,3%	2,4%	2,2%	2,6%	3,1%	0,72%	0,28%	0,02%	2,06%	11,48%	85,52%	1,48%	2,76%	2,51%	3,00%	3,55%	0,83%	0,32%	0,03%
563	2	135,75	112,84	20,9	2,0	83,12%	67,6%	2,1%	2,0%	3,5%	3,2%	3,5%	0,74%	0,36%	0,04%	1,48%	15,40%	81,37%	2,56%	2,46%	4,16%	3,89%	4,19%	0,89%	0,43%	0,05%
378	2	35,43	31,29	3,5	0,6	88,32%	78,0%	1,1%	1,6%	1,4%	2,3%	2,9%	0,93%	0,09%	0,01%	1,67%	10,01%	88,26%	1,23%	1,87%	1,63%	2,56%	3,28%	1,05%	0,11%	0,02%
344	2	364,71	204,70	81,0	79,0	56,13%	41,8%	5,8%	1,0%	1,7%	2,6%	1,0%	1,76%	0,38%	0,07%	21,65%	22,22%	74,46%	10,37%	1,79%	2,96%	4,63%	1,85%	3,13%	0,67%	0,13%
202	3	106,47	80,04	23,8	2,6	75,18%	55,7%	1,8%	1,5%	3,4%	4,3%	0,5%	7,13%	0,82%	0,11%	2,44%	22,38%	74,03%	2,34%	1,99%	4,50%	5,75%	0,67%	9,49%	1,09%	0,15%
1178	3	17,86	13,69	3,7	0,5	76,67%	60,0%	1,8%	2,1%	4,4%	6,0%	0,7%	0,98%	0,64%	0,08%	2,82%	20,51%	78,24%	2,31%	2,68%	5,78%	7,86%	0,93%	1,28%	0,83%	0,10%
612	4	6,07	3,83	1,4	0,9	63,18%	35,2%	3,0%	2,5%	6,1%	10,3%	2,1%	2,66%	1,30%	0,09%	14,22%	22,60%	55,69%	4,75%	3,94%	9,59%	16,24%	3,37%	4,21%	2,06%	0,14%
550	4	84,21	71,19	9,5	3,5	84,54%	69,7%	2,7%	2,4%	3,0%	3,1%	2,4%	0,95%	0,23%	0,03%	4,21%	11,25%	82,49%	3,16%	2,85%	3,58%	3,67%	2,83%	1,12%	0,27%	0,04%
639	4	347,42	295,90	46,0	5,5	85,17%	68,4%	1,5%	2,1%	3,2%	3,2%	0,6%	5,58%	0,58%	0,06%	1,60%	13,23%	80,26%	1,75%	2,49%	3,77%	3,76%	0,67%	6,55%	0,68%	0,07%
818	4	2,07	1,12	0,6	0,4	53,97%	20,3%	0,0%	1,6%	6,5%	18,4%	4,9%	1,71%	0,46%	0,06%	17,63%	28,40%	37,54%	0,00%	2,95%	12,12%	34,13%	9,11%	3,18%	0,85%	0,12%
495	4	85,12	77,41	6,5	1,2	90,94%	78,5%	1,0%	2,3%	1,5%	2,2%	4,6%	0,69%	0,10%	0,02%	1,39%	7,66%	86,36%	1,15%	2,49%	1,67%	2,43%	5,02%	0,76%	0,11%	0,02%
1167	4	56,45	45,24	8,9	2,3	80,14%	63,4%	2,2%	2,6%	4,0%	4,6%	1,4%	0,89%	0,90%	0,10%	4,16%	15,70%	79,11%	2,80%	3,21%	5,02%	5,71%	1,79%	1,11%	1,12%	0,12%
885	4	915,95	847,14	63,9	5,0	92,49%	82,9%	0,4%	1,5%	0,8%	1,1%	4,7%	1,06%	0,05%	0,01%	0,54%	6,97%	89,62%	0,39%	1,65%	0,85%	1,21%	5,07%	1,14%	0,05%	0,01%
256	4	133,87	119,38	12,7	1,8	89,18%	79,3%	1,2%	2,1%	2,1%	2,2%	1,2%	0,90%	0,18%	0,02%	1,32%	9,50%	88,87%	1,38%	2,36%	2,33%	2,51%	1,31%	1,01%	0,20%	0,03%
260	4	21,82	13,49	7,0	1,3	61,82%	28,6%	10,6%	2,9%	7,4%	9,6%	1,1%	0,51%	0,90%	0,11%	5,94%	32,25%	46,30%	17,20%	4,74%	12,01%	15,48%	1,81%	0,83%	1,45%	0,19%
600	4	20,24	16,06	3,4	0,8	79,32%	61,3%	2,0%	2,4%	4,3%	5,3%	1,5%	1,41%	0,91%	0,13%	3,72%	16,97%	77,33%	2,51%	3,08%	5,44%	6,63%	1,91%	1,78%	1,15%	0,16%
1299	4	54,47	46,54	6,9	1,0	85,43%	70,1%	2,3%	2,1%	3,3%	3,5%	1,4%	2,14%	0,52%	0,06%	1,83%	12,74%	82,08%	2,69%	2,41%	3,84%	4,13%	1,66%	2,51%	0,61%	0,07%
653	4	385,75	348,55	35,4	1,8	90,36%	78,3%	1,0%	2,4%	1,9%	2,1%	3,6%	0,98%	0,12%	0,02%	0,46%	9,19%	86,61%	1,10%	2,65%	2,05%	2,38%	3,98%	1,08%	0,13%	0,02%
313	4	24,81	21,07	3,2	0,5	84,94%	71,5%	1,3%	2,0%	2,4%	3,8%	1,8%	1,73%	0,38%	0,04%	1,98%	13,09%	84,15%	1,56%	2,34%	2,88%	4,46%	2,08%	2,04%	0,45%	0,05%
396	4	1799,69	1492,47	276,2	31,0	82,93%	68,4%	1,0%	2,3%	3,5%	2,8%	2,1%	2,41%	0,40%	0,07%	1,72%	15,35%	82,47%	1,16%	2,74%	4,26%	3,36%	2,55%	2,91%	0,48%	0,08%
1340	4	33,38	29,67	2,9	0,8	88,88%	76,0%	1,7%	1,2%	2,0%	3,0%	1,8%	2,91%	0,19%	0,03%	2,36%	8,76%	85,46%	1,88%	1,38%	2,31%	3,37%	2,08%	3,27%	0,21%	0,03%
688	4	383,77	328,78	45,8	9,1	85,67%	72,3%	1,2%	2,0%	3,0%	2,9%	1,0%	2,78%	0,49%	0,04%	2,38%	11,95%	84,42%	1,39%	2,33%	3,47%	3,35%	1,18%	3,25%	0,57%	0,05%
907	4	61,72	49,73	10,2	1,7	80,58%	64,2%	2,3%	2,1%	4,1%	4,4%	1,5%	0,87%	1,01%	0,10%	2,83%	16,60%	79,64%	2,84%	2,66%	5,05%	5,48%	1,86%	1,08%	1,25%	0,13%
350	4	18,04	15,97	1,5	0,6	88,52%	76,9%	1,2%	1,6%	2,1%	3,3%	2,3%	0,71%	0,30%	0,04%	3,21%	8,27%	86,88%	1,40%	1,80%	2,37%	3,78%	2,58%	0,81%	0,34%	0,04%
1313	5	59,65	49,93	7,4	2,4	83,70%	70,0%	1,6%	2,4%	2,5%	3,1%	2,8%	0,81%	0,44%	0,04%	3,98%	12,32%	83,68%	1,85%	2,89%	2,97%	3,71%	3,37%	0,96%	0,52%	0,05%
1180	5	29,08	20,22	6,8	2,1	69,52%	57,3%	2,7%	1,5%	1,6%	2,6%	2,3%	1,42%	0,11%	0,03%	7,16%	23,31%	82,47%	3,84%	2,09%	2,26%	3,76%	3,33%	2,04%	0,16%	0,04%
261	5	18,75	14,82	3,1	0,9	79,02%	62,0%	2,9%	1,8%	2,7%	5,2%	2,5%	1,77%	0,17%	0,03%	4,63%	16,35%	78,42%	3,62%	2,26%	3,41%	6,63%	3,17%	2,24%	0,21%	0,04%
644	5	82,10	66,24	13,0	2,9	80,68%	70,5%	1,7%	1,9%	1,2%	1,9%	2,4%	0,92%	0,09%	0,02%	3,50%	15,82%	87,42%	2,13%	2,32%	1,53%	2,39%	2,93%	1,14%	0,11%	0,03%
264	5	30,44	20,61	6,9	2,9	67,70%	54,1%	3,2%	1,6%	2,6%	3,3%	1,6%	1,12%	0,17%	0,04%	9,47%	22,82%	79,98%	4,70%	2,42%	3,78%	4,81%	2,35%	1,65%	0,25%	0,05%
347	6	243,05	189,63	48,6	4,8	78,02%	65,0%	2,4%	1,8%	3,2%	2,5%	1,8%	0,59%	0,65%	0,08%	1,97%	20,01%	83,27%	3,08%	2,33%	4,15%	3,20%	2,29%	0,75%	0,83%	0,10%
316	6	76,01	65,50	9,2	1,3	86,18%	73,4%	1,5%	2,0%	2,5%	2,6%	3,8%	0,42%	0,11%	0,02%	1,67%	12,15%	85,13%	1,72%	2,27%	2,91%	2,96%	4,37%	0,48%	0,13%	0,03%
1323	6	33,63	21,70	10,0	1,9	64,52%	35,2%	9,2%	3,1%	7,7%	6,6%	1,3%	0,61%	0,71%	0,13%	5,60%	29,88%	54,53%	14,22%	4,77%	11,90%	10,24%	2,08%	0,95%	1,10%	0,21%
235	6	394,90	34,81	148,0	1,3	8,82%	24,9%	5,8%	2,4%	8,0%	6,2%	0,2%	1,22%	1,72%	0,19%	11,94%	37,47%	79,50%	3,99%	2,01%	3,10%	4,06%	4,02%	3,06%	0,21%	0,04%
721	6	111,02	95,14	11,9	4,0	85,70%	74,4%	1,4%	2,0%	1,5%	2,2%	2,9%	1,17%	0,11%	0,02%	3,57%	10,73%	86,76%	1,67%	2,29%	1,78%	2,59%	3,38%	1,37%	0,13%	0,02%
345	6	36,24	22,95	10,8	2,5	63,33%	36,4%	7,1%	3,2%	7,2%	6,2%	0,7%	1,13%	1,21%	0,11%	6,86%	29,81%	57,43%	11,21%	5,09%	11,40%	9,84%	1,17%	1,79%	1,90%	0,17%
603	6	65,40	35,48	26,9	3,0	54,25%	32,7%	7,8%	1,1%	4,2%	4,8%	1,0%	1,03%	1,41%	0,20%	4,62%	41,13%	60,27%	14,30%	2,11%	7,75%	8,92%	1,80%	1,90%	2,60%	0,37%
269	6	156,74	137,31	17,4	2,0	87,60%	78,6%	0,8%	1,9%	1,0%	1,6%	2,8%	0,70%	0,11%	0,01%	1,31%	11,09%	89,67%	0,95%	2,19%	1,17%	1,87%	3,20%	0,80%	0,13%	0,02%
273	7	76,36	62,18	12,35	1,83	81,43%	61,6%	2,0%	1,9%	2,3%	3,0%	2,2%	0,08%	1,67%	0,12%	2,40%	16,17%	75,63%	2,40%	2,34%	2,78%	3,70%	2,75%	0,10%	2,05%	0,15%
706	7	427,39	375,82	48,7	2,9	87,93%	76,9%	0,9%	2,6%	1,0%	1,6%	4,5%	0,42%	0,05%	0,01%	0,68%	11,39%	87,40%	1,01%	2,95%	1,13%	1,87%	5,09%	0,48%	0,06%	0,01%
674	7	75,17	69,32	4,7	1,1	92,22%	81,3%	0,7%	2,2%	1,2%	2,4%	4,0%	0,27%	0,07%	0,01%	1,50%	6,27%	88,21%	0,77%	2,36%	1,35%	2,62%	4,31%	0,30%	0,08%	0,01%
697	7	31,04	27,32	3,1	0,6	88,03%	75,5%	1,1%	2,0%	1,5%	3,2%	3,6%	0,94%	0,23%	0,02%	2,03%	9,93%	85,78%	1,28%	2,22%	1,67%	3,60%	4,10%	1,06%	0,26%	0,03%
275	7	422,64	372,38	46,7	3,6	88,11%	76,6%	0,6%	2,1%	2,4%	2,2%	3,6%	0,44%	0,18%	0,02%	0,85%	11,04%	86,93%	0,73%	2,41%	2,68%	2,47%	4,05%	0,50%	0,21%	0,03%
304	7	102,94	97,29	4,7	1,0	94,52%	83,8%	0,9%	2,0%	1,3%	2,3%	2,9%	1,24%	0,09%	0,01%	0,93%	4,55%	88,67%	0,92%	2,15%	1,35%	2,42%	3,07%	1,31%	0,09%	0,01%
544	7	140,56	122,60	14,0	3,9	87,22%	73,8%	1,8%	2,5%	2,6%	2,3%	3,3%	0,60%	0,25%	0,03%	2,81%	9,97%	84,62%	2,09%	2,84%	2,98%	2,67%	3,79%	0,69%	0,29%	0,03%
687	7	41,46	38,24	2,7	0,5	92,22%	82,0%	0,3%	1,8%	1,1%	2,5%	3,7%	0,62%	0,17%	0,02%	1,29%	6,49%	88,93%	0,34%	1,98%	1,20%	2,67%	4,00%	0,67%	0,18%	0,02%
1208	7	107,05	101,29	4,9	0,8	94,61%	83,2%	1,1%	1,8%	1,6%	1,9%	3,1%	1,63%	0,14%	0,02%	0,79%	4,59%	87,98%	1,20%	1,92%	1,72%	2,03%	3,26%	1,72%	0,15%	0,02%
266	8	38,48	28,50	5,4	4,6	74,06%	61,3%	2,3%	1,9%	1,6%	2,5%	3,2%	1,10%	0,11%	0,02%	12,01%	13,93%	82,78%	3,14%	2,58%	2,15%	3,31%	4,36%	1,49%	0,15%	0,03%
487	8	19,51	16,99	2,0	0,5	87,10%	68,5%	2,8%	2,7%	3,9%	4,7%	3,2%	1,08%	0,23%	0,03%	2,49%	10,42%	78,64%	3,17%	3,07%	4,51%	5,38%	3,70%	1,24%	0,27%	0,04%
548	8	107,10	90,95	13,5	2,6	84,93%	76,3%	1,0%	1,8%	1,5%	2,1%	1,3%	0,92%	0,08%	0,01%	2,47%	12,60%	89,85%	1,12%	2,09%	1,81%	2,46%	1,48%	1,09%	0,09%	0,01%
346	8	18,13	15,84	1,8	0,5	87,40%	72,8%	1,0%	1,5%	1,8%	4,8%	4,0%	1,34%	0,13%	0,02%	2,94%	9,66%	83,34%	1,12%	1,66%	2,06%	5,53%	4,59%	1,53%	0,15%	0,02%
1300	9	29,74	23,98	5,2	0,6	80,65%	64,5%	1,8%	2,6%	3,5%	4,8%	1,4%	0,94%	1,21%	0,09%	1,91%	17,44%	79,94%	2,24%	3,18%	4,28%	5,91%	1,67%	1,17%	1,50%	0,12%
199	9	34,33	34,33	4,9		100,00%	72,6%	1,4%	1,9%	2,0%	2,8%	2,7%	0,53%	0,12%	0,01%	1,68%	14,37%	86,51%	1,64%	2,21%	2,33%	3,31%	3,21%	0,64%	0,14%	0,02%
719	9	114,31	103,77	8,3	2,2	90,78%	78,3%	1,0%	2,3%	1,9%	2,3%	3,7%	1,03%	0,23%	0,02%	1,97%	7,25%	86,27%	1,14%	2,58%	2,06%	2,51%	4,03%	1,13%	0,25%	0,02%
393	9	29,22	16,63	10,3	2,3	56,91%	17,9%	16,2%	2,6%	9,3%	7,6%	0,3%	2,03%	0,80%	0,13%	7,90%	35,19%	31,46%	28,52%	4,63%	16,31%	13,32%	0,55%	3,57%	1,41%	0,24%
541	9	15,82	9,65	3,8	2,4	60,99%	44,1%	4,5%	1,6%	3,3%	5,0%	1,0%	1,05%	0,41%	0,05%	15,11%	23,90%	72,36%	7,34%	2,65%	5,36%	8,15%	1,67%	1,72%	0,67%	0,08%
258	9	84,21	71,19	9,5		84,54%	69,7%	2,7%	2,4%	3,0%	3,1%	2,4%	0,95%	0,23%	0,03%	4,21%	11,25%	82,49%	3,16%	2,85%	3,58%	3,67%	2,83%	1,12%	0,27%	0,04%
715	9	14,38	7,3	5,9	1,3	50,44%	20,4%	6,5%	2,1%	6,6%	9,7%	0,8%	2,30%	1,83%	0,27%	8,73%	40,83%	40,39%	12,94%	4,16%	13,04%	19,20%	1,55%	4,56%	3,63%	0,53%
253	9	5202,01	2973,4	1049,5	1179,2	57,16%	41,9%	9,7%	0,5%	1,2%	0,0%	0,0%	3,67%	0,13%	0,02%	22,67%	20,17%	73,24%	17,03%	0,88%	2,10%	0,07%	0,01%	6,41%	0,23%	0,03%
636	9	21,12	17,4	2,4	1,3	82,20%	57,1%	1,3%	1,9%	2,8%	13,4%	4,3%	1,07%	0,32%	0,04%	6,37%	11,43%	69,48%	1,59%	2,26%	3,46%	16,27%	5,21%	1,30%	0,39%	0,05%
675	9	74,77	34,0	39,4	1,4	45,45%	5,6%	8,8%	3,8%	11,5%	11,4%	0,1%	0,66%	3,04%	0,36%	1,86%	52,69%	12,42%	19,45%	8,45%	25,27%	25,17%	0,32%	1,45%	6,69%	0,78%
1326	9	58,21	52,9	4,3	1,0	90,89%	80,8%	1,1%	1,4%	1,6%	2,4%	0,7%	2,68%	0,18%	0,02%	1,64%	7,47%	88,92%	1,23%	1,51%	1,77%	2,64%	0,77%	2,95%	0,19%	0,02%
68	9	106,47	9,9	23,8	1,0	9,27%	55,7%	1,8%	1,5%	3,4%	4,3%	0,5%	7,13%	0,82%	0,11%	2,44%	22,38%	81,90%	1,59%	2,16%	2,43%	5,97%	4,10%	1,72%	0,14%	0,00%
227	9	481,66	378,42	62,61	40,63	78,57%	61,7%	1,6%	1,7%	1,3%	2,3%	2,2%	2,60%	0,10%	0,04%	13,00%	8,44%	78,47%	2,03%	2,18%	1,64%	2,99%	2,85%	3,31%	0,13%	0,05%
594	9	28,78	15,6	3,5	9,6	54,37%	44,7%	2,3%	1,1%	2,3%	2,5%	0,3%	1,06%	0,19%	0,03%	33,51%	12,13%	82,18%	4,16%	2,09%	4,15%	4,53%	0,54%	1,94%	0,35%	0,06%
659	9	36,95	21,8	12,2	3,0	58,92%	29,9%	6,6%	2,7%	9,9%	8,2%	0,6%	0,34%	0,72%	0,07%	8,19%	32,89%	50,67%	11,18%	4,58%	16,75%	13,96%	0,95%	0,57%	1,22%	0,11%
507	9	4,09	2,8	1,0	0,4	67,91%	33,4%	4,9%	2,2%	8,0%	13,4%	2,5%	2,91%	0,58%	0,10%	8,77%	23,32%	49,14%	7,26%	3,31%	11,72%	19,67%	3,63%	4,28%	0,86%	0,14%
451	9	15,40	7,2	7,5	0,7	46,70%	9,8%	5,2%	3,1%	10,3%	12,1%	0,7%	1,12%	3,92%	0,39%	4,49%	48,81%	21,09%	11,14%	6,56%	22,11%	25,99%	1,46%	2,40%	8,40%	0,84%
454	9	29,54	17,1	11,2	1,3	57,74%	26,1%	5,6%	2,2%	7,9%	8,3%	0,5%	4,43%	2,35%	0,35%	4,39%	37,88%	45,21%	9,70%	3,78%	13,64%	14,38%	0,94%	7,67%	4,06%	0,61%
641	9	394,90	199,8	148,0	47,1	50,59%	24,9%	5,8%	2,4%	8,0%	6,2%	0,2%	1,22%	1,72%	0,19%	11,94%	37,47%	49,24%	11,48%	4,69%	15,86%	12,25%	0,30%	2,41%	3,40%	0,37%
336	9	85,06	60,00	14,40	10,66	70,54%	48,2%	3,7%	2,5%	1,7%	3,8%	0,9%	1,99%	0,34%	0,06%	16,93%	12,53%	68,39%	5,26%	3,49%	2,36%	5,39%	1,27%	2,82%	0,49%	0,08%
240	9	90,27	63,60	23,3	3,4	70,45%	55,4%	4,3%	0,9%	1,6%	3,1%	0,3%	4,00%	0,91%	0,07%	3,75%	25,80%	78,60%	6,05%	1,29%	2,31%	4,33%	0,36%	5,67%	1,29%	0,10%
571	10	71,48	65,32	5,5	0,7	91,38%	80,9%	0,6%	1,6%	1,6%	1,8%	4,0%	0,69%	0,10%	0,02%	0,93%	7,69%	88,50%	0,69%	1,79%	1,72%	1,99%	4,42%	0,75%	0,11%	0,02%
490	10	42,24	35,38	5,4	1,5	83,74%	69,2%	2,2%	1,9%	2,9%	3,2%	2,6%	1,28%	0,31%	0,03%	3,47%	12,79%	82,67%	2,68%	2,26%	3,52%	3,88%	3,05%	1,53%	0,37%	0,04%
429	10	61,37	37,25	22,6	1,5	60,69%	25,5%	7,2%	4,2%	11,1%	9,7%	0,4%	0,95%	1,51%	0,14%	2,42%	36,89%	41,98%	11,83%	6,88%	18,31%	16,03%	0,69%	1,56%	2,48%	0,24%
267	10	15,36	12,88	1,9	0,6	83,87%	68,1%	1,3%	1,8%	1,8%	4,5%	3,4%	2,75%	0,14%	0,02%	3,62%	12,50%	81,20%	1,54%	2,19%	2,19%	5,41%	4,01%	3,28%	0,16%	0,02%
306	10	1576,01	1473,86	96,5	5,6	93,52%	84,4%	0,5%	1,4%	0,9%	1,3%	4,2%	0,74%	0,05%	0,01%	0,36%	6,13%	90,27%	0,53%	1,47%	0,98%	1,41%	4,49%	0,79%	0,05%	0,01%
707	10	532,21	390,08	79,2	63,0	73,29%	60,4%	2,2%	1,9%	2,0%	2,6%	2,7%	1,26%	0,24%	0,03%	11,83%	14,88%	82,36%	3,04%	2,54%	2,73%	3,50%	3,75%	1,71%	0,33%	0,04%
207	10	27,21	20,69	5,0	1,5	76,04%	55,1%	2,6%	2,3%	4,0%	5,1%	2,2%	4,17%	0,49%	0,05%	5,68%	18,28%	72,45%	3,45%	2,98%	5,31%	6,70%	2,92%	5,48%	0,65%	0,06%
1318	11	181,47	126,65	52,4	2,5	69,79%	47,3%	3,2%	1,8%	5,3%	5,6%	0,9%	3,96%	1,57%	0,18%	1,36%	28,85%	67,76%	4,59%	2,65%	7,57%	7,95%	1,30%	5,68%	2,25%	0,26%
691	11	39,03	22,94	11,5	4,5	58,78%	34,7%	7,7%	2,7%	5,9%	5,0%	1,0%	0,99%	0,67%	0,10%	11,65%	29,57%	59,01%	13,08%	4,64%	10,05%	8,55%	1,68%	1,69%	1,14%	0,16%
494	11	54,64	35,30	7,9	11,5	64,60%	52,6%	1,9%	1,7%	2,5%	2,7%	1,6%	1,45%	0,12%	0,02%	21,01%	14,39%	81,49%	2,98%	2,66%	3,79%	4,17%	2,44%	2,25%	0,19%	0,03%
502	11	43,92	25,21	15,8	2,9	57,40%	31,9%	8,8%	3,0%	2,9%	7,1%	1,8%	1,17%	0,56%	0,06%	6,60%	36,01%	55,66%	15,42%	5,21%	5,06%	12,39%	3,17%	2,03%	0,97%	0,11%
651	11	49,45	38,98	7,5	2,9	78,83%	60,1%	3,2%	2,2%	4,9%	4,6%	1,9%	1,52%	0,31%	0,06%	5,95%	15,22%	76,23%	4,08%	2,82%	6,25%	5,82%	2,40%	1,93%	0,40%	0,07%
552	11	102,22	70,75	21,5	10,0	69,22%	54,2%	4,0%	1,4%	2,4%	2,7%	1,1%	3,02%	0,28%	0,05%	9,75%	21,04%	78,28%	5,81%	2,02%	3,49%	3,90%	1,65%	4,36%	0,40%	0,07%
666	11	78,34	64,50	8,1	5,7	82,34%	71,1%	2,0%	1,8%	1,3%	1,8%	3,0%	1,21%	0,08%	0,01%	7,32%	10,34%	86,38%	2,47%	2,19%	1,53%	2,17%	3,68%	1,47%	0,10%	0,02%
713	12	314,32	282,41	25,9	6,1	89,85%	81,5%	0,7%	2,0%	1,8%	1,8%	1,2%	0,71%	0,08%	0,01%	1,93%	8,23%	90,76%	0,81%	2,18%	2,03%	2,04%	1,28%	0,79%	0,09%	0,01%
692	12	49,37	40,17	8,1	1,1	81,37%	65,3%	2,5%	2,1%	3,5%	3,7%	1,9%	1,55%	0,72%	0,07%	2,27%	16,36%	80,20%	3,10%	2,56%	4,29%	4,57%	2,39%	1,91%	0,88%	0,09%
210	12	69,75	43,78	22,9	3,1	62,77%	40,4%	7,3%	2,1%	6,6%	4,3%	0,1%	0,95%	0,84%	0,06%	4,43%	32,80%	64,41%	11,70%	3,33%	10,54%	6,87%	0,20%	1,52%	1,33%	0,10%
650	12	310,75	253,69	46,7	10,4	81,64%	70,2%	1,4%	1,9%	2,8%	2,4%	1,2%	1,51%	0,31%	0,03%	3,35%	15,01%	86,00%	1,72%	2,28%	3,40%	2,90%	1,43%	1,85%	0,38%	0,04%
460	12	65,00	56,10	7,2	1,7	86,31%	74,6%	1,4%	1,9%	2,4%	2,6%	2,3%	0,85%	0,32%	0,03%	2,61%	11,08%	86,45%	1,61%	2,17%	2,78%	2,98%	2,62%	0,98%	0,37%	0,04%
717	12	20,83	17,09	2,7	1,0	82,04%	60,1%	2,0%	3,0%	4,1%	7,4%	3,0%	1,83%	0,64%	0,06%	4,91%	13,06%	73,25%	2,44%	3,64%	4,99%	8,99%	3,60%	2,23%	0,78%	0,08%
1174	12	184,98	142,67	39,9	2,5	77,13%	64,7%	3,8%	0,9%	1,2%	2,6%	0,3%	3,11%	0,49%	0,05%	1,33%	21,54%	83,85%	4,98%	1,22%	1,52%	3,34%	0,35%	4,03%	0,64%	0,07%
395	12	13,51	8,54	3,6	1,3	63,24%	41,5%	4,7%	2,4%	6,4%	5,9%	0,8%	0,98%	0,52%	0,09%	9,82%	26,95%	65,60%	7,43%	3,73%	10,14%	9,35%	1,23%	1,56%	0,82%	0,15%
455	12	10,44	6,29	3,0	1,2	60,30%	30,6%	3,6%	2,4%	5,6%	15,3%	1,3%	1,09%	0,46%	0,07%	11,36%	28,34%	50,70%	5,89%	3,91%	9,26%	25,45%	2,09%	1,81%	0,76%	0,11%
472	12	109,71	91,72	16,6	1,4	83,60%	67,7%	1,6%	1,5%	2,9%	3,0%	1,2%	5,15%	0,54%	0,07%	1,28%	15,11%	80,99%	1,88%	1,80%	3,45%	3,56%	1,44%	6,16%	0,65%	0,08%
303	12	70,96	53,94	12,5	4,5	76,02%	56,5%	2,5%	2,1%	4,1%	3,9%	1,2%	4,83%	0,76%	0,09%	6,38%	17,60%	74,39%	3,32%	2,80%	5,37%	5,09%	1,57%	6,36%	1,00%	0,11%
197	12	52,81	40,07	11,3	1,5	75,88%	60,1%	2,7%	1,5%	2,5%	4,7%	0,9%	1,99%	1,32%	0,09%	2,78%	21,33%	79,23%	3,49%	2,01%	3,33%	6,24%	1,22%	2,63%	1,73%	0,12%
723	12	65,34	58,71	5,5	1,2	89,85%	70,3%	7,7%	2,3%	3,2%	3,2%	0,4%	1,35%	1,44%	0,06%	1,78%	8,37%	78,27%	8,52%	2,52%	3,55%	3,59%	0,40%	1,50%	1,60%	0,06%

No.	Group No.	SM 32:1	SM 33:1	SM 34:2	SM 34:1	SM 34:0	SM 36:2	SM 36:1	SM 36:0	SM 38:2	SM 38:1	SM 40:2	SM 40:1	SM 41:2	SM 41:1	SM 42:3	SM 42:2	SM 42:1
380	1	0,85%	0,96%	2,49%	30,81%	2,27%	0,75%	3,60%	1,19%	0,48%	4,36%	1,49%	7,48%	1,69%	2,88%	4,69%	23,00%	12,55%
649	1	1,75%	2,11%	4,98%	30,35%	6,66%	0,00%	2,00%	0,00%	0,00%	0,00%	0,00%	22,75%	0,00%	0,68%	3,84%	13,07%	8,46%
566	1	4,05%	0,55%	4,78%	48,42%	0,43%	0,00%	1,76%	0,00%	0,57%	0,00%	0,00%	0,00%	0,00%	1,73%	2,73%	17,10%	4,92%
1349	1	1,27%	1,95%	0,00%	24,02%	0,00%	0,00%	5,89%	0,00%	2,46%	0,00%	2,45%	9,89%	1,18%	6,90%	1,79%	16,18%	12,49%
386	1	0,00%	0,00%	0,00%	34,87%	0,00%	0,00%	0,00%	0,00%	0,00%	0,00%	0,00%	10,19%	0,00%	5,14%	2,68%	10,45%	6,14%
206	1	0,00%	1,44%	3,77%	33,39%	0,72%	0,00%	3,74%	0,00%	1,13%	0,00%	3,64%	9,14%	0,00%	2,45%	1,76%	23,56%	10,48%
399	1	2,98%	0,00%	0,00%	30,35%	0,00%	0,00%	6,38%	5,76%	2,12%	0,00%	0,00%	8,31%	0,00%	3,28%	2,31%	14,20%	17,29%
725	1	0,00%	5,10%	1,45%	38,25%	0,00%	0,00%	2,55%	10,13%	0,00%	0,00%	0,00%	4,00%	0,00%	1,08%	6,58%	18,07%	9,16%
560	1	5,18%	0,00%	1,61%	28,20%	2,03%	1,80%	2,79%	0,00%	6,62%	0,00%	2,54%	1,41%	1,58%	1,52%	5,01%	17,55%	7,47%
436	1	2,20%	2,25%	3,45%	44,61%	3,07%	0,00%	0,00%	0,00%	1,69%	0,00%	7,73%	0,00%	0,00%	3,78%	0,00%	14,82%	14,39%
435	1	0,75%	0,77%	1,06%	32,14%	2,31%	0,38%	2,11%	0,00%	2,60%	2,36%	5,16%	8,26%	0,00%	1,36%	1,20%	18,30%	9,21%
357	2	2,49%	0,81%	0,78%	39,00%	1,03%	0,00%	0,00%	0,00%	3,59%	2,69%	5,36%	9,88%	0,00%	3,45%	0,68%	13,12%	10,46%
638	2	3,04%	0,36%	2,99%	35,09%	2,45%	0,14%	4,69%	0,00%	0,00%	4,89%	0,00%	6,79%	0,76%	1,71%	5,51%	16,68%	5,80%
727	2	0,49%	0,00%	0,00%	40,57%	2,72%	4,26%	2,71%	0,00%	0,00%	0,00%	8,34%	1,45%	0,00%	2,38%	4,41%	17,34%	10,02%
252	2	0,92%	1,69%	1,52%	39,12%	0,42%	0,78%	4,39%	0,00%	2,10%	2,35%	0,00%	6,77%	2,05%	2,13%	4,67%	18,16%	9,62%
549	2	1,57%	0,26%	1,27%	32,40%	1,49%	0,28%	5,93%	0,00%	0,65%	4,89%	2,01%	7,72%	0,25%	1,68%	3,06%	27,40%	8,59%
553	2	2,24%	1,16%	2,64%	44,64%	3,16%	0,40%	2,63%	1,62%	0,78%	1,24%	0,00%	7,22%	1,25%	2,57%	2,64%	15,76%	7,66%
1227	2	2,42%	1,71%	1,63%	39,12%	2,19%	1,19%	5,39%	0,00%	0,85%	2,48%	0,00%	4,60%	0,28%	2,40%	1,50%	17,28%	10,27%
563	2	2,24%	1,11%	2,03%	46,54%	3,70%	0,00%	3,62%	0,00%	0,22%	3,33%	1,35%	2,94%	1,32%	0,96%	3,41%	21,76%	5,64%
378	2	1,37%	0,84%	0,00%	27,72%	2,46%	0,00%	2,65%	10,30%	0,00%	7,93%	9,75%	0,00%	4,34%	3,48%	1,47%	20,13%	13,09%
344	2	1,01%	0,97%	1,08%	47,70%	3,03%	0,38%	1,16%	0,55%	0,00%	0,60%	0,42%	3,20%	0,75%	2,12%	2,82%	27,77%	7,56%
202	3	2,27%	0,51%	2,57%	30,80%	2,91%	1,49%	5,62%	0,00%	0,00%	6,29%	1,69%	7,70%	1,24%	3,61%	3,47%	18,81%	7,57%
1178	3	0,00%	0,00%	2,27%	37,59%	1,66%	0,00%	2,49%	0,00%	0,00%	4,73%	3,24%	9,94%	0,00%	1,39%	1,38%	20,03%	8,57%
612	4	0,00%	1,57%	0,00%	29,08%	11,05%	2,12%	0,00%	0,00%	0,00%	15,95%	7,85%	4,72%	0,00%	2,01%	4,51%	17,23%	3,87%
550	4	0,94%	0,89%	2,20%	33,76%	3,36%	0,36%	2,56%	0,00%	0,00%	2,35%	1,18%	5,69%	0,70%	1,57%	7,30%	29,01%	7,41%
639	4	1,45%	1,09%	1,55%	30,61%	2,70%	0,00%	5,74%	0,00%	0,00%	5,67%	1,93%	8,36%	1,60%	2,70%	5,10%	16,80%	8,97%
818	4	0,00%	0,00%	0,00%	0,00%	0,00%	0,00%	0,00%	0,00%	0,00%	0,00%	0,00%	0,00%	0,00%	0,00%	0,00%	0,00%	0,00%
495	4	0,80%	0,38%	2,85%	31,72%	2,40%	0,44%	4,80%	1,19%	1,92%	3,60%	9,25%	13,47%	0,00%	0,92%	1,69%	18,41%	6,19%
1167	4	2,10%	0,78%	1,03%	42,06%	1,66%	0,00%	5,36%	6,67%	0,00%	7,74%	0,55%	11,18%	0,47%	1,44%	2,95%	14,04%	3,27%
885	4	2,47%	0,00%	4,70%	38,56%	1,31%	0,00%	1,20%	0,00%	0,94%	0,00%	0,00%	8,88%	0,00%	2,37%	1,71%	15,65%	12,56%
256	4	2,14%	1,14%	1,06%	30,94%	1,40%	0,00%	2,70%	0,00%	0,00%	0,00%	6,44%	5,08%	1,24%	2,17%	5,22%	23,28%	9,11%
260	4	1,59%	0,56%	0,84%	29,54%	4,02%	0,41%	5,57%	1,06%	0,29%	5,18%	1,63%	11,13%	0,83%	1,21%	3,40%	26,59%	9,14%
600	4	0,00%	0,00%	0,00%	41,99%	10,18%	0,00%	5,14%	5,09%	0,00%	6,80%	0,00%	6,24%	0,00%	1,45%	0,00%	22,10%	13,91%
1299	4	1,45%	0,61%	1,73%	41,23%	1,00%	0,00%	1,50%	0,00%	0,54%	1,31%	0,64%	5,85%	0,56%	2,42%	4,95%	22,43%	9,49%
653	4	1,84%	0,44%	1,86%	33,47%	0,00%	1,27%	2,59%	0,00%	0,00%	1,21%	2,05%	2,01%	0,68%	2,41%	4,06%	24,43%	11,37%
313	4	0,70%	0,00%	3,07%	26,73%	3,92%	0,00%	12,65%	22,39%	0,00%	8,86%	0,00%	7,72%	3,03%	3,86%	3,70%	15,52%	5,38%
396	4	3,38%	0,93%	2,50%	36,85%	3,13%	0,23%	5,47%	0,18%	0,00%	0,91%	0,99%	4,01%	1,60%	1,40%	5,55%	21,68%	8,84%
1340	4	2,86%	0,00%	1,04%	28,36%	6,20%	1,63%	0,99%	2,74%	0,00%	14,14%	4,26%	2,91%	0,00%	0,70%	3,53%	16,87%	7,85%
688	4	1,87%	0,52%	1,56%	31,33%	4,09%	0,60%	6,88%	0,00%	0,00%	6,21%	0,00%	7,88%	0,77%	2,54%	2,80%	20,45%	7,51%
907	4	2,58%	1,43%	1,93%	39,14%	3,96%	0,70%	4,87%	0,00%	0,41%	4,35%	0,50%	8,58%	0,47%	1,96%	1,45%	19,19%	4,99%
350	4	0,00%	0,00%	0,00%	51,75%	9,02%	0,00%	11,63%	20,88%	0,00%	2,69%	0,00%	0,00%	0,00%	0,00%	0,00%	27,81%	3,96%
1313	5	0,7%	0,9%	2,1%	39,4%	3,5%	1,8%	5,3%	0,0%	1,4%	4,1%	3,1%	0,6%	0,0%	1,4%	3,8%	15,2%	8,0%
1180	5	1,9%	2,2%	3,8%	50,1%	0,6%	0,0%	4,6%	2,0%	0,9%	0,0%	0,0%	3,5%	1,8%	2,0%	11,1%	11,3%	2,6%
261	5	0,4%	2,1%	1,5%	30,6%	0,6%	1,2%	2,3%	0,0%	3,4%	12,0%	1,8%	6,3%	0,0%	2,3%	4,4%	19,4%	6,9%
644	5	2,8%	2,0%	3,3%	44,8%	0,3%	1,2%	1,0%	0,0%	1,9%	4,0%	0,7%	0,4%	0,0%	1,4%	4,0%	16,4%	6,4%
264	5	0,9%	1,2%	2,7%	38,1%	2,0%	0,5%	2,8%	0,0%	1,0%	0,0%	1,2%	5,8%	0,7%	1,4%	5,8%	17,6%	5,7%
347	6	1,84%	0,96%	1,36%	50,88%	4,26%	0,31%	2,08%	0,00%	0,34%	2,58%	0,00%	4,13%	0,90%	1,18%	2,21%	18,46%	7,97%
316	6	3,52%	1,64%	2,39%	46,82%	5,26%	0,30%	0,68%	0,00%	0,00%	0,00%	0,00%	0,00%	0,00%	1,95%	4,05%	24,28%	7,19%
1323	6	2,29%	0,60%	0,71%	35,36%	3,21%	0,00%	3,57%	0,00%	0,00%	1,83%	1,13%	6,16%	0,58%	1,53%	2,91%	30,21%	10,66%
235	6	1,65%	1,29%	3,42%	30,71%	4,40%	0,00%	5,03%	0,00%	0,00%	8,43%	0,89%	6,80%	0,46%	1,28%	4,24%	20,80%	11,29%
721	6	2,45%	1,04%	2,94%	38,19%	2,78%	1,57%	3,13%	0,00%	0,00%	0,00%	0,00%	0,00%	0,69%	1,78%	7,45%	23,35%	6,08%
345	6	0,63%	0,51%	1,13%	29,07%	3,53%	0,54%	4,92%	0,00%	1,34%	3,07%	1,25%	6,61%	1,01%	1,36%	3,48%	34,11%	8,16%
603	6	1,71%	0,34%	1,48%	32,89%	3,66%	0,62%	5,46%	1,28%	0,37%	3,08%	2,49%	7,32%	0,95%	2,00%	2,81%	26,91%	8,55%
269	6	2,16%	0,39%	0,90%	31,79%	0,99%	0,00%	2,20%	0,00%	0,00%	0,72%	0,00%	5,83%	0,73%	2,28%	3,67%	25,70%	10,57%
273	7	0,38%	0,35%	1,41%	39,51%	0,00%	1,62%	0,00%	0,00%	0,00%	5,20%	0,00%	5,23%	0,30%	4,54%	2,90%	25,87%	8,32%
706	7	2,01%	1,17%	4,22%	36,02%	0,67%	0,16%	2,53%	0,00%	0,46%	10,16%	0,65%	5,50%	0,85%	2,51%	0,56%	16,23%	9,70%
674	7	2,25%	2,62%	1,45%	29,84%	0,00%	0,78%	5,60%	7,87%	0,00%	0,00%	5,93%	0,00%	0,00%	0,99%	9,82%	17,83%	8,86%
697	7	1,05%	0,00%	1,63%	27,14%	0,00%	0,00%	5,87%	0,00%	0,00%	13,42%	0,00%	9,60%	0,00%	5,67%	2,46%	14,01%	10,60%
275	7	2,27%	1,70%	1,12%	41,48%	0,00%	0,58%	2,51%	0,00%	0,63%	5,74%	0,00%	5,06%	0,00%	1,12%	0,54%	17,70%	7,53%
304	7	1,72%	1,50%	0,91%	27,16%	0,00%	0,91%	2,51%	0,00%	0,00%	10,53%	3,66%	11,42%	0,00%	2,05%	1,32%	16,68%	9,51%
544	7	1,85%	0,78%	1,58%	35,05%	0,72%	0,41%	3,03%	0,00%	1,14%	3,63%	4,45%	4,79%	1,04%	1,95%	4,50%	22,91%	9,73%
687	7	2,18%	0,00%	0,00%	22,20%	0,00%	3,18%	2,95%	0,00%	5,18%	0,00%	0,00%	0,00%	0,00%	2,07%	11,61%	14,22%	14,53%
1208	7	0,55%	0,00%	0,00%	34,85%	0,00%	0,00%	9,03%	1,83%	0,00%	0,00%	0,00%	11,29%	1,41%	2,16%	2,47%	25,74%	9,04%
266	8	4,20%	1,96%	3,59%	32,07%	0,60%	1,45%	3,89%	0,00%	0,71%	4,00%	7,75%	6,08%	1,06%	2,12%	8,75%	10,33%	5,22%
487	8	0,00%	2,34%	0,00%	56,22%	1,81%	0,00%	0,00%	0,00%	0,00%	0,00%	0,00%	2,92%	0,00%	3,95%	1,12%	18,04%	10,91%
548	8	1,55%	1,66%	3,04%	46,87%	2,06%	0,00%	2,51%	0,00%	1,16%	0,00%	1,41%	1,94%	1,18%	3,32%	4,02%	18,77%	6,95%
346	8	0,00%	0,00%	0,00%	42,78%	2,36%	0,00%	0,00%	0,00%	6,87%	0,00%	0,00%	0,00%	0,00%	0,00%	13,90%	13,00%	8,02%
1300	9	1,67%	0,00%	0,00%	35,32%	2,40%	0,00%	8,21%	3,83%	0,00%	9,83%	1,53%	2,09%	0,00%	3,74%	3,33%	16,71%	7,80%
199	9	2,07%	0,32%	1,96%	34,76%	8,41%	0,63%	3,79%	0,00%	0,04%	1,85%	2,39%	8,06%	0,55%	1,91%	3,56%	23,43%	11,50%
719	9	0,33%	1,47%	0,87%	29,87%	0,45%	1,33%	4,45%	10,27%	2,24%	5,88%	0,00%	13,14%	2,10%	1,53%	3,88%	19,01%	8,93%
393	9	0,31%	0,00%	1,27%	27,75%	1,90%	0,26%	3,63%	0,47%	1,08%	3,24%	1,56%	10,73%	0,57%	3,07%	3,25%	18,95%	22,67%
541	9	1,20%	1,01%	1,53%	41,45%	4,32%	0,00%	4,76%	0,00%	0,00%	4,52%	2,03%	6,04%	0,00%	2,30%	5,10%	17,60%	9,96%
258	9	2,52%	0,83%	2,55%	49,76%	5,14%	0,81%	4,68%	0,77%	0,00%	2,36%	0,65%	3,50%	0,48%	0,96%	4,29%	17,54%	4,63%
715	9	1,16%	0,00%	1,13%	33,19%	2,88%	0,51%	6,15%	0,00%	0,39%	6,29%	4,80%	6,27%	1,60%	2,68%	4,75%	18,21%	10,18%
253	9	3,85%	1,93%	3,82%	28,49%	1,56%	2,49%	5,93%	0,31%	1,26%	4,35%	6,26%	7,95%	2,88%	3,49%	5,03%	14,00%	4,65%
636	9	0,00%	4,01%	0,00%	49,84%	0,00%	0,00%	0,00%	8,64%	0,00%	4,52%	0,00%	11,94%	9,28%	2,41%	0,00%	7,49%	0,00%
675	9	1,32%	0,45%	0,61%	25,08%	2,70%	0,43%	6,70%	0,55%	0,46%	6,87%	1,74%	8,77%	0,93%	1,90%	3,64%	26,69%	9,28%
1326	9	2,01%	0,49%	2,59%	23,93%	0,00%	0,64%	4,68%	0,00%	0,00%	13,64%	0,00%	5,09%	0,00%	2,55%	4,25%	15,43%	6,87%
68	9	0,00%	1,93%	0,00%	12,86%	0,00%	0,00%	0,00%	10,48%	0,00%	0,00%	16,16%	25,96%	0,00%	3,44%	0,00%	20,80%	15,74%
227	9	2,45%	1,91%	2,34%	37,26%	3,13%	0,80%	0,00%	6,64%	0,11%	2,19%	0,00%	6,69%	1,03%	3,76%	4,86%	21,19%	11,23%
594	9	2,27%	0,00%	0,00%	33,69%	3,69%	0,00%	3,43%	0,00%	2,17%	2,86%	3,80%	7,91%	1,47%	2,45%	4,18%	16,27%	13,97%
659	9	3,83%	2,16%	3,02%	50,07%	2,26%	0,50%	2,46%	1,30%	0,51%	0,49%	3,09%	2,22%	0,99%	0,63%	3,41%	18,52%	5,26%
507	9	0,00%	2,28%	0,00%	46,28%	0,00%	0,00%	0,83%	0,00%	0,00%	0,00%	2,11%	2,00%	0,00%	2,32%	6,42%	22,61%	9,21%
451	9	0,98%	0,00%	1,76%	38,14%	3,45%	0,00%	7,61%	0,00%	0,00%	7,28%	0,68%	6,17%	0,00%	2,61%	3,90%	16,30%	8,21%
454	9	0,93%	1,29%	0,00%	30,56%	3,80%	0,22%	5,12%	1,60%	0,20%	5,82%	2,06%	7,55%	0,62%	1,54%	2,45%	26,18%	9,69%
641	9	1,20%	0,58%	1,82%	38,90%	3,39%	0,46%	4,50%	0,39%	0,78%	3,16%	0,75%	5,97%	0,90%	2,03%	5,57%	20,55%	9,56%
336	9	0,51%	1,46%	1,92%	38,67%	2,79%	1,47%	3,63%	0,00%	0,17%	1,82%	1,81%	4,21%	1,38%	3,80%	3,77%	20,43%	12,09%
240	9	1,35%	0,75%	1,64%	42,07%	6,51%	0,52%	3,90%	1,18%	0,18%	1,68%	2,22%	8,22%	0,52%	2,40%	2,37%	20,69%	8,92%
571	10	0,00%	0,84%	1,45%	43,27%	0,00%	7,84%	2,37%	12,07%	0,00%	0,00%	0,00%	0,00%	1,87%	0,00%	0,00%	16,05%	10,37%
490	10	1,21%	0,00%	2,31%	45,86%	0,76%	0,52%	2,30%	0,47%	2,07%	3,34%	4,65%	0,00%	0,39%	2,58%	2,16%	19,16%	7,24%
429	10	0,65%	0,74%	0,34%	46,41%	26,88%	0,30%	2,06%	2,51%	0,00%	6,12%	0,19%	4,67%	0,48%	2,71%	2,17%	17,48%	10,82%
267	10	0,00%	0,00%	0,00%	40,80%	1,47%	0,00%	0,00%	0,00%	3,52%	0,00%	0,00%	3,54%	2,57%	0,00%	0,00%	25,68%	15,71%
306	10	1,72%	0,34%	3,56%	33,76%	0,39%	1,69%	1,59%	0,00%	0,00%	0,00%	0,00%	0,00%	0,80%	1,57%	3,85%	25,59%	7,46%
707	10	1,28%	0,74%	1,36%	49,62%	1,32%	0,14%	1,10%	0,00%	0,00%	0,00%	1,21%	4,13%	0,76%	2,33%	2,67%	21,48%	10,92%
207	10	0,84%	0,00%	3,35%	32,64%	1,32%	0,47%	7,43%	0,00%	0,00%	3,64%	4,68%	6,01%	1,33%	2,39%	1,95%	19,15%	13,29%
1318	11	0,58%	0,52%	0,95%	36,13%	3,06%	0,66%	5,07%	0,00%	0,00%	5,21%	0,00%	8,22%	0,60%	2,95%	5,74%	17,53%	10,02%
691	11	1,35%	0,84%	1,80%	39,26%	5,55%	0,31%	4,41%	2,60%	0,57%	2,19%	0,57%	6,94%	0,52%	1,46%	3,42%	25,98%	6,65%
494	11	2,67%	1,13%	3,18%	29,63%	2,38%	0,50%	1,23%	0,00%	0,33%	0,86%	3,78%	8,41%	1,46%	2,15%	4,99%	22,34%	12,22%
502	11	2,12%	2,11%	3,22%	27,09%	2,42%	1,40%	2,85%	0,41%	0,29%	1,39%	3,00%	9,57%	1,42%	1,55%	3,01%	20,89%	14,57%
651	11	0,83%	0,93%	0,96%	40,09%	1,44%	0,32%	5,64%	0,00%	0,00%	1,45%	0,00%	11,62%	1,08%	3,30%	2,27%	15,86%	9,61%
552	11	1,14%	0,58%	1,54%	33,14%	2,55%	0,38%	2,97%	1,73%	1,22%	2,62%	2,28%	7,32%	0,30%	3,33%	2,22%	27,29%	10,10%
666	11	1,04%	1,48%	1,96%	26,03%	2,60%	1,93%	4,51%	0,00%	2,88%	6,59%	5,75%	6,95%	1,62%	1,70%	4,97%	19,28%	7,44%
713	12	2,10%	1,39%	3,01%	33,08%	1,19%	1,57%	4,34%	0,00%	0,00%	3,91%	0,00%	5,17%	1,86%	2,53%	0,77%	22,45%	11,90%
692	12	1,43%	0,49%	1,67%	33,32%	2,45%	0,45%	3,48%	0,00%	1,25%	5,41%	1,87%	6,38%	0,73%	2,41%	4,42%	25,43%	7,57%
210	12	1,17%	0,61%	3,05%	31,12%	4,84%	2,42%	6,56%	2,03%	0,87%	2,31%	2,74%	6,25%	0,84%	1,93%	6,02%	18,08%	11,50%
650	12	3,73%	1,64%	2,33%	36,18%	1,56%	0,94%	3,86%	0,00%	0,00%	0,00%	3,92%	6,03%	1,01%	2,15%	3,71%	18,97%	9,32%
460	12	1,84%	0,73%	1,96%	35,59%	0,69%	1,87%	3,40%	0,00%	0,00%	7,16%	0,00%	6,82%	3,32%	1,78%	3,61%	19,60%	6,18%
717	12	0,00%	0,00%	0,00%	42,37%	1,49%	1,31%	4,75%	2,09%	0,00%	1,61%	3,31%	4,45%	0,00%	2,01%	2,95%	19,67%	8,08%
1174	12	1,55%	0,60%	3,18%	31,28%	4,43%	2,01%	5,82%	2,93%	0,53%	5,34%	2,61%	8,44%	0,18%	1,40%	4,33%	14,29%	12,37%
395	12	0,54%	1,58%	1,90%	41,49%	0,61%	0,00%	1,44%	0,00%	0,00%	0,36%	0,00%	4,89%	0,00%	2,65%	3,45%	22,93%	15,87%
455	12	1,05%	0,00%	0,00%	38,11%	0,78%	0,00%	3,45%	0,00%	0,00%	0,91%	3,60%	5,51%	0,00%	1,63%	4,50%	24,65%	11,03%
472	12	0,91%	0,53%	0,61%	33,12%	1,43%	0,80%	6,08%	3,19%	0,38%	8,81%	1,57%	9,30%	1,60%	2,96%	4,74%	17,29%	7,63%
303	12	1,62%	0,93%	2,05%	34,23%	4,79%	1,28%	3,64%	0,00%	0,00%	3,47%	3,81%	7,52%	0,87%	2,20%	4,17%	19,14%	7,60%
197	12	1,24%	0,71%	2,07%	41,04%	2,66%	0,62%	4,95%	0,00%	0,00%	2,26%	0,70%	5,94%	0,00%	2,38%	3,18%	21,00%	8,46%
723	12	0,52%	0,51%	0,99%	30,67%	3,12%	0,00%	5,17%	1,00%	0,05%	4,71%	2,02%	7,69%	0,73%	1,28%	5,42%	27,41%	8,59%

No.	Group No.	PC 30:1	PC 30:0	PC O-32:1	PC O-32:0	PC 32:2	PC 32:1	PC 32:0	PC O-34:2	PC O-34:1	PC O-34:0	PC 34:3	PC 34:2	PC 34:1	PC 34:0	PC O-36:4	PC 36:4	PC 36:3	PC 36:2	PC 36:1	PC 38:5	PC 38:4
380	1	0,22%	5,11%	0,54%	2,08%	0,24%	10,31%	40,89%	0,19%	1,20%	1,3%	0,39%	5,58%	16,15%	1,43%	0,26%	1,81%	1,86%	2,83%	1,65%	0,45%	0,72%
649	1	0,45%	8,29%	0,57%	2,97%	0,55%	13,86%	43,80%	0,19%	1,48%	2,2%	0,62%	4,33%	10,74%	1,02%	0,09%	1,06%	1,13%	1,75%	0,86%	0,28%	0,23%
566	1	0,58%	5,14%	0,37%	0,87%	0,91%	12,51%	40,72%	0,26%	1,19%	0,6%	0,95%	6,78%	13,37%	1,42%	0,07%	0,74%	2,81%	3,87%	1,07%	0,14%	0,14%
1349	1	0,22%	8,41%	0,48%	2,96%	0,29%	6,30%	47,71%	0,13%	1,17%	2,3%	0,35%	6,01%	10,46%	2,15%	0,05%	1,41%	1,33%	2,58%	1,19%	0,29%	0,42%
386	1	0,30%	8,07%	0,59%	1,73%	0,18%	6,55%	51,97%	0,26%	0,91%	1,0%	0,57%	5,98%	10,72%	1,39%	0,32%	1,27%	1,47%	2,24%	0,79%	0,13%	0,40%
206	1	0,34%	9,52%	0,58%	2,95%	0,24%	8,06%	50,82%	0,21%	1,51%	2,3%	0,31%	3,58%	8,90%	1,55%	0,23%	1,27%	0,89%	1,56%	0,76%	0,24%	0,31%
399	1	0,20%	8,37%	0,53%	3,04%	0,21%	6,77%	43,94%	0,22%	1,15%	2,0%	0,36%	4,69%	13,52%	1,74%	0,18%	1,92%	1,80%	2,17%	1,45%	0,44%	0,69%
725	1	0,43%	9,95%	0,41%	1,69%	0,47%	9,04%	47,12%	0,16%	0,78%	0,9%	0,85%	6,00%	9,76%	0,84%	0,15%	1,42%	2,09%	2,47%	0,96%	0,30%	0,54%
560	1	0,24%	7,76%	0,75%	3,21%	0,24%	6,91%	47,29%	0,17%	1,06%	2,5%	0,31%	4,59%	11,03%	2,06%	0,15%	1,50%	1,51%	2,02%	1,30%	0,28%	0,44%
436	1	0,35%	9,08%	0,45%	2,41%	0,55%	7,08%	44,09%	0,24%	0,92%	1,7%	0,80%	7,75%	8,26%	2,74%	0,11%	2,04%	2,16%	3,59%	0,82%	0,40%	0,58%
435	1	0,43%	10,77%	0,64%	3,68%	0,38%	9,35%	44,91%	0,25%	1,97%	2,4%	0,40%	4,19%	9,61%	1,37%	0,16%	1,17%	1,08%	1,44%	0,86%	0,20%	0,33%
357	2	0,51%	12,38%	0,60%	4,37%	0,44%	10,69%	36,60%	0,28%	1,95%	2,7%	0,35%	4,31%	13,56%	1,09%	0,25%	1,12%	0,73%	1,56%	1,49%	0,27%	0,36%
638	2	0,67%	11,48%	0,67%	1,79%	0,85%	9,29%	42,68%	0,25%	0,84%	1,1%	0,80%	5,58%	9,36%	2,10%	0,16%	2,05%	2,48%	2,36%	0,72%	0,47%	0,55%
727	2	0,44%	11,17%	0,60%	3,13%	0,29%	8,05%	44,48%	0,28%	1,14%	1,9%	0,61%	5,02%	9,81%	1,49%	0,17%	1,70%	1,39%	2,15%	1,07%	0,32%	0,33%
252	2	0,34%	6,77%	0,67%	2,31%	0,17%	6,55%	49,23%	0,37%	1,30%	1,8%	0,38%	5,49%	11,52%	1,45%	0,27%	1,11%	1,45%	2,12%	1,31%	0,29%	0,31%
549	2	0,34%	5,95%	13,19%	8,92%	0,28%	4,09%	32,73%	2,53%	5,42%	1,1%	0,22%	1,64%	7,30%	1,33%	1,00%	0,71%	0,75%	1,08%	0,61%	0,30%	0,14%
553	2	0,36%	11,84%	0,78%	4,99%	0,36%	9,23%	34,62%	0,52%	2,81%	2,8%	0,40%	3,65%	12,30%	1,21%	0,31%	0,99%	0,89%	2,12%	2,25%	0,35%	0,42%
1227	2	0,50%	11,95%	0,62%	4,59%	0,39%	9,75%	35,62%	0,22%	1,83%	2,3%	0,43%	4,50%	14,05%	1,31%	0,15%	1,25%	1,40%	2,22%	1,58%	0,38%	0,46%
563	2	0,43%	11,27%	0,77%	2,92%	0,50%	9,35%	37,51%	0,55%	1,66%	1,3%	0,74%	5,45%	12,38%	1,36%	0,23%	1,23%	1,67%	2,77%	1,69%	0,26%	0,29%
378	2	0,16%	9,91%	0,53%	3,50%	0,22%	7,57%	47,01%	0,14%	1,42%	2,6%	0,32%	4,15%	11,76%	1,66%	0,00%	0,95%	0,81%	1,73%	1,32%	0,16%	0,24%
344	2	0,69%	5,98%	2,79%	4,15%	0,60%	8,17%	25,57%	1,28%	2,99%	1,0%	0,37%	4,59%	15,41%	1,39%	2,26%	1,61%	1,58%	4,46%	2,04%	0,28%	0,63%
202	3	0,53%	9,12%	0,78%	1,73%	0,79%	9,82%	46,59%	0,32%	0,87%	0,6%	0,68%	4,98%	10,44%	1,09%	0,22%	1,98%	1,58%	1,80%	0,73%	0,41%	0,45%
1178	3	0,14%	7,14%	0,86%	1,92%	0,62%	11,20%	52,21%	0,30%	1,25%	0,5%	0,19%	2,53%	8,86%	0,93%	0,35%	2,36%	0,70%	1,10%	0,66%	0,34%	0,52%
612	4	0,51%	6,74%	0,34%	0,88%	0,38%	6,96%	31,88%	0,88%	0,56%	0,9%	1,08%	7,99%	9,70%	2,56%	0,00%	2,43%	4,21%	6,12%	2,43%	0,74%	1,03%
550	4	0,66%	6,42%	0,64%	1,37%	0,96%	12,18%	40,82%	0,31%	0,96%	0,5%	0,99%	7,03%	12,94%	1,00%	0,29%	1,94%	2,40%	2,99%	0,70%	0,45%	0,59%
639	4	0,54%	9,57%	0,60%	1,66%	0,76%	11,86%	47,44%	0,20%	0,75%	0,6%	0,71%	5,31%	8,84%	1,06%	0,20%	1,65%	1,53%	1,98%	0,62%	0,29%	0,45%
818	4	0,00%	3,09%	0,00%	6,07%	0,00%	1,57%	32,50%	0,00%	0,00%	0,0%	0,00%	0,92%	5,06%	0,00%	2,14%	0,00%	1,58%	0,82%	0,00%	0,00%	0,00%
495	4	0,29%	7,43%	0,43%	0,99%	0,37%	7,66%	48,70%	0,25%	0,86%	0,5%	0,59%	6,73%	13,21%	1,16%	0,17%	1,18%	2,26%	2,72%	0,83%	0,23%	0,20%
1167	4	0,41%	9,82%	0,47%	1,21%	0,49%	5,23%	45,69%	0,18%	0,68%	0,6%	0,87%	7,11%	10,75%	1,71%	0,12%	1,90%	2,42%	3,33%	1,23%	0,48%	0,87%
885	4	0,43%	7,55%	0,36%	1,29%	0,75%	7,36%	42,62%	0,20%	0,59%	0,8%	1,18%	7,31%	11,09%	2,43%	0,09%	1,87%	3,05%	3,51%	1,10%	0,38%	0,59%
256	4	0,70%	9,96%	0,47%	1,35%	0,99%	10,35%	47,18%	0,18%	0,73%	0,6%	0,85%	5,11%	8,66%	1,32%	0,14%	1,84%	2,05%	2,10%	0,66%	0,39%	0,46%
260	4	1,60%	10,52%	2,06%	4,30%	0,42%	7,03%	36,38%	0,80%	1,88%	1,5%	0,34%	2,74%	9,08%	1,66%	1,32%	1,40%	0,92%	1,44%	0,59%	0,23%	0,62%
600	4	0,80%	10,77%	0,89%	2,16%	1,04%	9,90%	36,78%	0,46%	1,10%	0,9%	1,17%	5,37%	10,51%	1,68%	0,34%	2,47%	1,88%	2,79%	1,22%	0,62%	0,94%
1299	4	1,25%	12,07%	0,95%	1,55%	1,07%	11,93%	37,12%	0,50%	1,36%	0,6%	0,43%	3,98%	11,83%	1,40%	0,60%	1,41%	1,31%	2,33%	1,03%	0,30%	0,49%
653	4	1,17%	10,88%	0,47%	1,05%	0,89%	10,65%	41,01%	0,21%	0,64%	0,4%	1,33%	7,01%	11,55%	0,91%	0,12%	1,49%	2,66%	2,99%	0,74%	0,32%	0,31%
313	4	0,84%	8,19%	0,59%	1,11%	0,90%	12,15%	40,71%	0,43%	0,60%	0,5%	1,22%	6,61%	11,34%	1,40%	0,16%	1,27%	3,00%	3,03%	1,02%	0,42%	0,29%
396	4	0,71%	10,87%	0,65%	1,96%	0,78%	8,97%	39,28%	0,24%	1,01%	1,0%	0,70%	4,93%	10,92%	2,14%	0,25%	2,95%	2,34%	2,99%	1,04%	0,81%	1,11%
1340	4	0,82%	11,19%	0,52%	1,59%	0,92%	7,80%	40,83%	0,31%	0,90%	1,0%	1,06%	6,02%	9,35%	1,88%	0,27%	2,47%	2,73%	2,78%	0,80%	0,56%	0,79%
688	4	0,37%	10,03%	0,38%	1,02%	0,41%	6,40%	46,90%	0,16%	0,57%	0,6%	0,54%	5,74%	11,27%	2,45%	0,08%	1,64%	2,55%	3,37%	1,21%	0,31%	0,53%
907	4	0,76%	10,03%	0,54%	1,91%	0,77%	7,12%	37,58%	0,39%	1,06%	1,1%	0,73%	5,95%	11,86%	2,21%	0,30%	2,12%	2,57%	3,52%	1,50%	0,62%	1,01%
350	4	0,68%	10,60%	0,47%	1,12%	0,66%	9,37%	44,83%	0,09%	0,89%	0,7%	0,58%	4,32%	11,84%	1,62%	0,00%	1,07%	1,56%	2,20%	1,48%	0,31%	0,39%
1313	5	0,35%	9,03%	0,46%	2,89%	0,19%	8,07%	40,33%	0,17%	1,31%	2,2%	0,30%	4,84%	14,67%	1,80%	0,20%	1,11%	1,58%	2,86%	2,25%	0,49%	0,61%
1180	5	0,59%	12,68%	0,91%	3,48%	0,47%	8,16%	36,75%	0,25%	1,71%	1,6%	0,52%	4,42%	10,67%	1,29%	1,22%	2,28%	1,78%	2,12%	1,11%	0,59%	0,83%
261	5	0,45%	10,31%	0,55%	2,90%	0,62%	8,97%	42,91%	0,47%	1,52%	1,7%	0,50%	4,63%	9,93%	1,96%	0,20%	1,07%	1,75%	2,47%	1,20%	0,57%	0,53%
644	5	0,48%	11,87%	0,83%	2,90%	0,49%	8,35%	40,37%	0,28%	1,48%	1,6%	0,59%	4,90%	10,16%	2,04%	0,46%	1,63%	1,83%	2,73%	1,43%	0,38%	0,66%
264	5	0,48%	10,43%	0,88%	3,49%	0,43%	9,48%	39,56%	0,33%	1,27%	1,6%	0,52%	5,23%	9,80%	1,57%	1,21%	1,74%	1,42%	2,22%	0,92%	0,33%	0,90%
347	6	0,31%	10,52%	0,81%	2,38%	0,45%	7,15%	48,84%	0,34%	0,92%	0,7%	0,48%	6,19%	8,38%	1,27%	0,74%	1,50%	1,60%	2,31%	0,78%	0,24%	0,52%
316	6	0,50%	12,18%	0,68%	1,82%	0,55%	9,23%	47,18%	0,30%	1,19%	0,5%	0,42%	5,00%	9,96%	1,14%	0,38%	1,01%	1,06%	1,95%	0,71%	0,26%	0,46%
1323	6	0,51%	8,42%	2,41%	4,13%	0,56%	7,61%	32,27%	1,20%	2,59%	0,6%	0,63%	5,31%	10,56%	0,67%	2,37%	1,71%	1,68%	2,65%	0,98%	0,48%	0,77%
235	6	1,05%	12,75%	0,90%	3,23%	1,06%	9,80%	38,83%	0,37%	1,52%	1,3%	1,19%	4,86%	8,88%	1,41%	0,52%	1,89%	1,86%	2,27%	0,70%	0,42%	0,66%
721	6	0,64%	8,78%	0,63%	1,97%	0,85%	9,57%	36,98%	0,26%	1,12%	1,0%	1,21%	7,34%	12,42%	1,45%	0,21%	2,10%	3,47%	3,23%	0,78%	0,58%	0,53%
345	6	1,12%	7,10%	1,41%	2,69%	0,67%	9,12%	32,86%	0,69%	2,05%	1,2%	0,60%	5,05%	13,14%	1,00%	0,63%	2,01%	2,45%	3,97%	1,49%	0,65%	1,17%
603	6	0,31%	5,69%	13,98%	9,42%	0,23%	4,50%	32,02%	2,41%	5,18%	1,1%	0,27%	1,86%	7,26%	1,21%	1,14%	0,59%	0,33%	1,22%	0,66%	0,15%	0,31%
269	6	0,32%	6,91%	0,38%	1,61%	0,58%	10,40%	49,82%	0,14%	0,98%	1,1%	0,45%	3,54%	12,00%	1,43%	0,07%	1,40%	1,53%	2,69%	0,86%	0,56%	0,38%
273	7	0,50%	9,83%	0,49%	1,52%	0,61%	10,10%	45,99%	0,24%	0,85%	0,6%	0,71%	4,48%	11,87%	1,32%	0,19%	1,33%	1,76%	2,34%	1,05%	0,19%	0,37%
706	7	0,38%	9,91%	0,49%	2,36%	0,33%	8,90%	48,98%	0,15%	0,95%	1,4%	0,47%	4,90%	11,02%	1,55%	0,13%	0,97%	1,19%	1,98%	0,95%	0,20%	0,26%
674	7	0,46%	12,29%	0,36%	1,74%	0,45%	7,58%	45,38%	0,15%	0,84%	1,1%	0,57%	6,02%	11,01%	1,83%	0,05%	1,33%	1,80%	2,67%	1,07%	0,29%	0,29%
697	7	0,36%	10,33%	0,36%	2,87%	0,19%	5,40%	50,87%	0,13%	1,03%	1,9%	0,35%	3,67%	10,65%	2,36%	0,05%	0,73%	0,94%	1,92%	1,23%	0,21%	0,26%
275	7	0,96%	13,89%	0,67%	2,16%	0,85%	9,09%	38,99%	0,25%	1,08%	1,4%	0,71%	4,84%	9,46%	1,70%	0,15%	2,23%	2,28%	2,54%	0,95%	0,63%	0,72%
304	7	0,57%	12,88%	0,49%	2,17%	0,45%	10,09%	47,76%	0,14%	0,93%	1,5%	0,37%	4,30%	8,88%	1,73%	0,09%	1,11%	0,90%	1,58%	0,86%	0,21%	0,32%
544	7	0,34%	9,38%	0,46%	1,05%	0,37%	8,44%	46,47%	0,25%	0,64%	0,4%	0,67%	7,36%	12,31%	1,10%	0,36%	1,30%	1,76%	2,66%	0,89%	0,25%	0,33%
687	7	0,54%	11,09%	0,47%	1,57%	0,42%	7,89%	48,58%	0,14%	0,79%	1,1%	0,62%	4,56%	10,81%	1,70%	0,03%	0,81%	1,67%	2,58%	1,02%	0,23%	0,18%
1208	7	0,60%	12,59%	0,39%	1,21%	0,50%	7,44%	49,21%	0,18%	0,66%	0,8%	0,77%	5,10%	9,85%	1,44%	0,16%	1,26%	1,82%	2,05%	0,72%	0,21%	0,24%
266	8	0,78%	11,79%	0,77%	3,12%	0,68%	11,33%	36,46%	0,27%	1,57%	2,0%	0,58%	5,28%	10,78%	1,20%	0,65%	2,04%	1,40%	1,86%	0,81%	0,44%	0,49%
487	8	0,72%	9,91%	1,18%	2,38%	0,64%	11,62%	34,25%	0,48%	1,50%	1,3%	0,85%	7,04%	12,04%	1,00%	0,84%	1,52%	1,63%	2,55%	1,10%	0,29%	0,63%
548	8	0,46%	9,84%	0,58%	2,69%	0,61%	7,77%	46,06%	0,26%	1,18%	1,7%	0,91%	6,38%	8,34%	1,92%	0,20%	1,22%	2,08%	2,46%	0,80%	0,29%	0,31%
346	8	0,40%	8,63%	0,63%	1,56%	0,69%	10,23%	46,81%	0,09%	0,87%	0,3%	0,63%	5,11%	11,45%	1,51%	0,07%	1,30%	2,39%	2,93%	0,73%	0,41%	0,23%
1300	9	0,43%	11,17%	0,42%	1,19%	0,56%	6,06%	41,14%	0,23%	0,72%	1,0%	0,50%	7,30%	9,76%	2,40%	0,00%	1,91%	2,78%	4,03%	1,57%	0,40%	0,62%
199	9	1,12%	13,08%	0,54%	1,53%	2,08%	16,19%	38,73%	0,16%	0,69%	0,4%	0,68%	3,52%	12,11%	0,60%	0,12%	1,41%	1,24%	1,24%	0,74%	0,33%	0,45%
719	9	0,49%	10,13%	0,48%	2,28%	0,45%	9,87%	47,73%	0,17%	1,01%	1,5%	0,50%	5,34%	9,73%	1,27%	0,08%	1,01%	1,42%	1,98%	0,86%	0,21%	0,30%
393	9	1,12%	8,00%	3,93%	3,37%	0,80%	13,44%	24,26%	0,85%	1,06%	0,7%	0,21%	6,47%	12,07%	0,41%	1,16%	2,99%	2,29%	1,90%	1,28%	0,51%	2,06%
541	9	0,28%	8,96%	0,76%	2,71%	0,30%	7,32%	37,37%	0,33%	1,39%	0,9%	0,57%	6,03%	10,31%	1,62%	1,03%	2,22%	2,12%	3,25%	1,96%	0,54%	1,04%
258	9	0,14%	1,29%	1,20%	3,48%	0,24%	1,60%	7,97%	2,56%	4,46%	0,8%	0,27%	5,86%	19,71%	1,42%	1,24%	2,23%	4,63%	10,83%	6,49%	0,90%	2,30%
715	9	0,00%	2,81%	2,79%	3,90%	0,29%	3,37%	24,93%	1,24%	2,73%	1,4%	0,00%	5,61%	13,50%	1,17%	0,73%	3,50%	1,72%	3,61%	4,36%	0,59%	1,26%
253	9	0,14%	0,33%	0,19%	0,31%	0,58%	1,07%	0,70%	0,81%	0,68%	0,1%	1,50%	29,99%	11,57%	0,20%	0,88%	7,75%	7,51%	13,95%	1,65%	2,59%	3,26%
636	9	0,48%	10,08%	0,44%	1,24%	0,57%	9,34%	45,72%	0,27%	1,21%	2,3%	0,25%	3,14%	10,61%	1,83%	0,00%	0,35%	0,93%	2,05%	0,83%	0,47%	0,13%
675	9	0,45%	3,67%	2,64%	3,09%	0,65%	5,00%	11,92%	1,81%	2,05%	1,8%	0,19%	4,28%	19,22%	1,60%	1,08%	2,38%	2,43%	5,18%	2,85%	0,61%	2,38%
1326	9	0,48%	5,16%	0,64%	1,03%	1,14%	8,40%	36,26%	0,31%	0,57%	0,6%	1,90%	8,64%	10,27%	1,82%	0,32%	5,05%	4,62%	3,22%	0,68%	1,34%	1,28%
68	9	0,34%	7,93%	0,63%	2,70%	0,55%	8,36%	44,33%	0,10%	1,44%	2,3%	0,94%	5,61%	12,21%	1,77%	0,10%	1,08%	1,33%	1,70%	1,01%	0,52%	0,31%
227	9	0,57%	10,14%	0,68%	2,28%	0,92%	11,88%	44,42%	0,25%	0,98%	0,8%	0,64%	6,03%	10,94%	1,31%	0,43%	1,27%	1,07%	1,24%	0,48%	0,24%	0,29%
594	9	0,44%	11,31%	0,89%	3,42%	0,61%	10,25%	44,26%	0,33%	2,02%	1,7%	0,30%	4,07%	8,67%	1,04%	0,25%	1,29%	0,86%	1,85%	0,90%	0,21%	0,39%
659	9	0,30%	5,56%	1,07%	6,19%	0,40%	6,63%	22,38%	1,49%	4,19%	1,9%	0,39%	5,82%	12,48%	1,06%	0,89%	2,11%	1,71%	4,27%	3,45%	0,53%	1,11%
507	9	0,00%	9,13%	1,30%	2,05%	0,50%	6,33%	31,31%	1,16%	1,13%	1,5%	0,66%	4,38%	9,20%	0,57%	3,37%	2,61%	1,52%	2,64%	0,97%	1,05%	0,66%
451	9	0,00%	1,70%	1,98%	3,82%	0,20%	4,12%	20,12%	2,48%	3,12%	1,5%	0,00%	3,90%	16,59%	1,27%	0,99%	0,85%	1,52%	5,25%	4,43%	0,00%	1,13%
454	9	0,50%	9,67%	1,33%	2,38%	1,69%	12,31%	36,81%	0,92%	1,28%	1,0%	0,52%	3,35%	10,72%	0,87%	0,78%	1,45%	0,96%	2,22%	1,63%	0,33%	0,89%
641	9	0,27%	4,99%	2,86%	5,33%	0,73%	6,39%	20,73%	2,97%	4,07%	1,3%	0,45%	4,75%	11,74%	1,39%	1,75%	2,02%	2,09%	4,34%	2,50%	0,53%	1,12%
336	9	0,31%	8,36%	1,06%	1,62%	0,65%	9,62%	48,09%	0,33%	1,05%	0,5%	0,60%	6,55%	8,71%	1,16%	0,61%	1,29%	0,96%	1,54%	0,66%	0,23%	0,70%
240	9	0,34%	9,80%	1,44%	2,84%	0,52%	10,11%	44,04%	0,44%	1,75%	1,1%	0,17%	3,27%	11,79%	1,76%	0,11%	0,29%	0,58%	1,83%	1,72%	0,10%	0,17%
571	10	0,43%	9,19%	0,34%	1,07%	0,59%	7,43%	44,22%	0,16%	0,55%	0,4%	0,69%	7,80%	11,31%	2,02%	0,12%	1,83%	2,99%	3,64%	1,19%	0,29%	0,45%
490	10	0,62%	11,64%	0,63%	2,63%	0,64%	9,84%	44,71%	0,44%	1,21%	1,1%	0,61%	4,69%	8,55%	1,24%	0,33%	1,30%	1,32%	2,32%	0,89%	0,33%	0,37%
429	10	0,00%	1,41%	1,28%	3,93%	0,17%	3,12%	8,25%	2,49%	5,89%	0,6%	0,29%	3,23%	21,75%	0,98%	0,93%	1,00%	3,11%	13,24%	6,49%	0,75%	0,98%
267	10	0,53%	11,61%	0,60%	3,44%	0,40%	9,80%	48,95%	0,30%	1,16%	2,2%	0,35%	2,79%	8,35%	1,52%	0,07%	0,49%	0,62%	1,36%	0,88%	0,23%	0,22%
306	10	0,37%	4,32%	0,30%	0,71%	0,77%	8,78%	36,04%	0,22%	0,86%	0,6%	1,59%	9,57%	14,09%	2,04%	0,08%	2,31%	6,04%	6,03%	1,14%	0,37%	0,34%
707	10	0,84%	12,79%	1,15%	1,93%	0,74%	10,20%	44,86%	0,55%	1,19%	0,7%	0,57%	3,74%	8,54%	1,17%	0,92%	0,77%	1,06%	1,64%	0,85%	0,14%	0,26%
207	10	0,46%	6,71%	0,54%	2,10%	0,49%	11,85%	38,62%	0,23%	1,21%	1,1%	0,58%	5,78%	15,14%	1,29%	0,15%	1,83%	1,29%	2,91%	1,94%	0,44%	0,56%
1318	11	0,34%	9,77%	0,90%	1,98%	0,69%	7,49%	49,56%	0,50%	0,93%	0,5%	1,08%	7,31%	7,02%	0,82%	0,31%	1,27%	2,32%	1,78%	0,53%	0,24%	0,32%
691	11	0,48%	5,78%	1,48%	3,82%	0,72%	7,49%	29,55%	0,63%	2,62%	1,1%	0,53%	5,69%	15,19%	1,12%	1,25%	2,04%	2,25%	3,82%	1,34%	0,78%	1,00%
494	11	0,98%	14,86%	0,95%	2,60%	1,14%	12,81%	36,19%	0,34%	0,91%	1,0%	1,03%	5,70%	8,52%	0,67%	0,47%	1,95%	1,71%	1,76%	0,59%	0,33%	0,45%
502	11	0,31%	6,95%	1,52%	2,94%	1,01%	10,13%	26,09%	1,09%	1,75%	1,2%	0,95%	8,00%	15,13%	0,96%	0,82%	1,51%	2,22%	3,87%	1,16%	0,53%	0,60%
651	11	1,43%	13,72%	1,17%	4,13%	1,35%	11,32%	36,40%	0,34%	1,29%	1,2%	1,03%	3,74%	7,47%	0,74%	1,21%	2,20%	1,56%	1,46%	0,47%	0,38%	0,60%
552	11	0,58%	6,65%	1,46%	2,79%	0,89%	12,86%	36,91%	0,63%	1,85%	1,2%	0,55%	4,80%	13,73%	1,38%	0,56%	1,73%	1,18%	2,23%	1,10%	0,39%	0,49%
666	11	0,29%	6,60%	0,56%	2,18%	0,35%	8,97%	44,62%	0,22%	1,17%	1,7%	0,42%	5,48%	12,06%	1,93%	0,22%	1,84%	1,64%	2,94%	1,39%	0,43%	0,78%
713	12	0,47%	7,55%	0,67%	1,82%	0,75%	7,84%	42,44%	0,24%	1,06%	0,6%	0,85%	5,16%	11,97%	1,95%	0,30%	2,77%	3,02%	2,97%	0,90%	0,81%	0,85%
692	12	0,67%	8,95%	0,47%	0,93%	0,65%	7,55%	46,03%	0,21%	0,80%	1,1%	0,69%	5,07%	11,43%	1,45%	0,28%	1,77%	2,57%	3,03%	1,13%	0,44%	0,45%
210	12	0,13%	3,44%	2,17%	1,87%	0,22%	5,09%	31,33%	0,54%	1,36%	0,4%	0,32%	4,97%	15,47%	1,61%	1,21%	7,69%	1,97%	2,65%	2,28%	1,22%	3,24%
650	12	0,51%	10,39%	0,68%	1,54%	0,63%	6,79%	46,40%	0,35%	0,81%	0,6%	0,87%	6,47%	8,91%	1,66%	0,28%	1,90%	2,56%	2,82%	0,93%	0,34%	0,53%
460	12	0,19%	8,75%	0,40%	0,93%	0,29%	5,15%	51,68%	0,29%	0,62%	0,5%	0,62%	5,76%	10,25%	2,04%	0,14%	1,74%	2,46%	2,77%	1,02%	0,33%	0,40%
717	12	0,57%	7,10%	0,33%	0,96%	0,56%	11,03%	42,35%	0,24%	0,98%	1,0%	0,45%	5,12%	13,72%	0,89%	0,10%	1,59%	1,56%	3,48%	2,19%	0,47%	0,62%
1174	12	0,68%	6,79%	1,27%	1,65%	0,51%	11,76%	43,14%	0,30%	1,49%	0,3%	0,13%	3,06%	15,51%	2,24%	0,05%	0,68%	0,47%	1,61%	1,70%	0,09%	0,24%
395	12	0,59%	6,77%	1,06%	3,10%	0,33%	5,55%	31,71%	1,02%	1,95%	0,8%	0,86%	5,39%	12,66%	1,50%	4,46%	2,68%	1,76%	3,06%	1,16%	0,42%	1,03%
455	12	0,34%	7,24%	1,32%	2,75%	0,21%	7,15%	42,39%	0,56%	0,87%	0,7%	0,49%	5,53%	8,97%	0,42%	0,83%	2,13%	1,45%	4,16%	0,98%	0,39%	1,26%
472	12	0,42%	5,83%	0,86%	1,20%	0,70%	10,37%	41,10%	0,32%	0,83%	0,8%	0,69%	5,30%	15,24%	1,50%	0,20%	1,98%	2,48%	3,42%	0,96%	0,57%	0,61%
303	12	0,56%	8,23%	0,67%	1,78%	1,11%	10,74%	36,47%	0,47%	1,28%	0,8%	0,92%	4,96%	11,71%	1,37%	0,36%	2,90%	2,61%	3,07%	1,28%	0,77%	1,24%
197	12	0,70%	11,57%	0,89%	1,86%	0,68%	6,97%	44,31%	0,54%	0,97%	0,7%	0,41%	4,92%	9,40%	1,92%	0,30%	1,06%	1,32%	2,45%	1,68%	0,20%	0,42%
723	12	0,27%	8,78%	0,67%	1,55%	0,41%	8,85%	37,83%	0,46%	1,27%	0,6%	0,40%	6,22%	13,31%	2,09%	0,40%	2,23%	1,76%	3,32%	1,57%	0,52%	1,20%

No.	Group No.	PE 32:2	PE 32:1	PE 32:0	PE 34:3	PE 34:2	PE 34:1	PE 34:0	PE 36:5	PE 36:4	PE 36:3	PE 36:2	PE 36:1	PE 38:5	PE 38:4	PE 38:3	PE 38:2	PE 40:6	PE 40:5	PE 40:4
380	1	0,39%	1,85%	0,85%	0,84%	8,28%	22,12%	1,20%	0,52%	3,13%	6,92%	19,37%	11,99%	2,43%	4,93%	0,95%	0,65%	0,80%	0,68%	0,67%
649	1	0,45%	4,09%	1,74%	1,33%	12,91%	26,22%	1,12%	1,03%	4,54%	8,99%	16,77%	7,15%	2,42%	2,84%	0,53%	0,33%	0,51%	0,44%	0,17%
566	1	0,38%	3,48%	3,58%	1,07%	10,60%	27,82%	2,54%	0,32%	1,89%	10,46%	22,76%	8,91%	0,81%	1,16%	0,55%	0,93%	0,26%	0,18%	0,13%
1349	1	0,52%	1,92%	1,01%	0,84%	13,23%	20,00%	1,29%	0,54%	4,57%	12,06%	18,17%	8,14%	2,62%	3,92%	0,64%	0,62%	0,91%	0,59%	0,22%
386	1	0,62%	1,44%	1,28%	0,98%	9,80%	19,30%	0,72%	0,45%	3,39%	12,63%	16,88%	7,68%	1,97%	3,71%	0,83%	0,60%	0,33%	0,46%	0,36%
206	1	0,53%	2,06%	1,62%	0,93%	8,04%	21,13%	1,20%	0,68%	4,01%	9,78%	17,74%	10,13%	3,10%	5,08%	0,72%	0,47%	0,70%	0,79%	0,46%
399	1	0,54%	2,07%	1,04%	0,73%	7,40%	21,20%	1,85%	0,71%	4,34%	7,27%	17,10%	9,55%	3,41%	6,07%	0,92%	0,45%	0,81%	0,88%	0,44%
725	1	0,77%	1,91%	2,51%	1,55%	8,15%	24,42%	1,01%	0,59%	2,42%	6,66%	13,64%	8,48%	1,23%	3,22%	1,72%	0,47%	0,00%	1,01%	0,79%
560	1	0,58%	1,74%	1,38%	1,18%	7,64%	23,65%	1,35%	0,52%	4,09%	9,02%	16,16%	9,06%	2,62%	4,81%	0,90%	0,50%	0,35%	0,61%	0,59%
436	1	0,57%	1,63%	1,60%	0,95%	11,86%	16,18%	1,48%	0,41%	4,56%	14,59%	17,23%	9,30%	2,27%	4,55%	1,29%	0,54%	0,93%	0,63%	0,39%
435	1	0,62%	3,48%	1,45%	0,84%	9,99%	23,31%	1,01%	0,72%	4,78%	8,23%	15,80%	10,33%	2,74%	4,81%	1,05%	0,65%	0,82%	0,48%	0,59%
357	2	0,61%	4,20%	1,13%	1,02%	10,28%	25,91%	0,99%	0,74%	4,00%	6,98%	16,96%	10,59%	2,29%	3,96%	0,74%	0,42%	0,62%	0,46%	0,27%
638	2	0,36%	2,34%	2,41%	0,75%	9,41%	22,22%	2,10%	0,53%	4,70%	10,77%	15,56%	11,12%	3,53%	5,35%	1,58%	0,57%	1,45%	0,72%	0,64%
727	2	0,51%	2,53%	1,28%	0,91%	9,32%	19,46%	1,60%	0,78%	4,21%	9,41%	16,38%	10,31%	2,50%	4,61%	1,04%	0,74%	0,96%	0,57%	0,65%
252	2	0,34%	1,86%	1,31%	0,80%	10,26%	21,57%	1,09%	0,41%	3,52%	10,50%	16,37%	14,95%	1,89%	4,16%	1,14%	0,72%	0,60%	0,89%	0,93%
549	2	0,72%	3,64%	3,11%	0,73%	6,64%	24,10%	2,08%	0,66%	3,44%	2,71%	11,37%	11,75%	1,85%	4,01%	1,49%	0,84%	1,02%	0,63%	1,08%
553	2	0,59%	4,01%	1,14%	0,74%	7,14%	22,08%	1,62%	0,54%	2,85%	4,96%	14,81%	23,65%	1,44%	4,57%	1,75%	0,83%	0,60%	0,98%	1,04%
1227	2	0,46%	3,27%	1,02%	0,76%	9,54%	25,95%	1,19%	0,74%	3,65%	7,87%	18,72%	11,76%	2,48%	4,42%	1,20%	0,50%	0,96%	0,90%	0,59%
563	2	0,35%	2,68%	1,11%	1,24%	10,43%	21,34%	1,12%	0,49%	2,76%	8,98%	18,06%	18,59%	1,51%	3,21%	1,12%	0,72%	0,69%	0,63%	0,70%
378	2	0,53%	3,26%	0,78%	1,12%	10,53%	23,42%	1,06%	0,43%	2,90%	8,71%	18,92%	10,87%	1,64%	2,73%	0,63%	0,79%	0,41%	0,53%	0,26%
344	2	0,29%	1,65%	1,85%	0,47%	4,58%	20,86%	1,74%	0,82%	2,65%	1,64%	9,95%	31,25%	1,28%	11,38%	1,62%	0,74%	0,71%	1,19%	1,47%
202	3	0,34%	1,73%	1,71%	0,44%	5,92%	23,75%	1,19%	0,40%	2,77%	3,46%	11,95%	17,24%	1,94%	4,66%	2,49%	1,02%	2,63%	2,03%	3,64%
1178	3	0,67%	2,40%	1,49%	0,84%	5,01%	23,56%	1,43%	0,84%	4,25%	2,50%	9,36%	13,01%	2,48%	5,46%	1,10%	1,00%	1,82%	1,53%	1,73%
612	4	1,16%	2,67%	3,05%	1,21%	5,81%	16,15%	1,18%	0,47%	1,86%	4,28%	7,25%	11,42%	0,83%	3,21%	1,63%	0,63%	0,66%	1,41%	1,62%
550	4	0,39%	3,07%	1,48%	1,06%	12,40%	24,98%	0,90%	0,71%	4,93%	8,35%	16,18%	8,80%	2,94%	4,66%	0,99%	0,66%	0,87%	0,87%	0,94%
639	4	0,35%	2,77%	1,10%	0,75%	11,28%	25,60%	0,73%	0,56%	5,55%	8,25%	16,22%	9,84%	2,65%	4,98%	1,18%	0,50%	1,15%	1,03%	1,59%
818	4	2,31%	1,81%	2,07%	1,52%	2,32%	4,85%	1,61%	1,08%	1,30%	1,54%	1,69%	2,23%	0,67%	1,20%	0,73%	0,77%	0,00%	1,09%	0,85%
495	4	0,35%	2,07%	2,33%	1,12%	11,81%	24,84%	1,60%	0,32%	3,21%	15,09%	20,50%	7,43%	1,29%	2,21%	0,61%	0,41%	0,25%	0,33%	0,28%
1167	4	0,32%	1,11%	1,36%	0,54%	9,53%	22,15%	0,84%	0,58%	3,33%	7,40%	15,07%	13,42%	1,96%	5,35%	2,37%	0,85%	3,00%	1,19%	1,35%
885	4	0,36%	2,17%	1,63%	1,12%	14,21%	24,50%	1,78%	0,74%	5,06%	10,43%	18,46%	9,40%	1,74%	3,93%	1,41%	0,38%	0,52%	0,26%	0,19%
256	4	0,34%	1,93%	1,40%	0,91%	9,73%	22,51%	0,60%	0,60%	4,75%	11,95%	20,37%	8,44%	3,70%	4,84%	1,17%	0,78%	0,58%	0,61%	0,63%
260	4	0,52%	3,37%	1,91%	0,64%	7,72%	25,05%	1,42%	0,74%	6,24%	3,78%	10,35%	11,55%	2,79%	5,96%	1,16%	0,69%	0,92%	1,28%	1,21%
600	4	1,01%	3,31%	1,73%	0,90%	7,52%	19,19%	1,33%	0,63%	3,64%	4,34%	11,64%	15,23%	2,24%	5,16%	2,58%	1,20%	1,18%	1,55%	1,80%
1299	4	0,50%	3,70%	2,30%	0,80%	6,10%	23,17%	2,49%	0,91%	4,81%	2,83%	11,03%	16,48%	1,87%	8,39%	1,34%	0,79%	1,60%	0,88%	1,30%
653	4	0,40%	3,31%	1,99%	1,27%	11,65%	27,23%	1,47%	0,54%	2,73%	10,71%	21,26%	8,57%	1,64%	2,43%	0,93%	0,60%	0,43%	0,36%	0,34%
313	4	0,64%	2,82%	1,60%	1,03%	9,01%	20,75%	1,16%	0,49%	1,86%	8,97%	19,84%	10,23%	1,60%	2,91%	1,21%	0,68%	0,60%	0,95%	0,79%
396	4	0,31%	2,42%	1,62%	0,61%	7,38%	21,29%	1,48%	0,63%	6,34%	7,13%	15,73%	13,39%	4,26%	8,08%	1,93%	0,72%	1,51%	0,77%	0,86%
1340	4	0,78%	3,46%	3,49%	0,77%	8,18%	23,06%	3,57%	0,81%	3,68%	5,05%	10,16%	10,57%	1,51%	3,59%	1,10%	0,53%	1,58%	0,61%	1,01%
688	4	0,19%	1,31%	1,21%	0,57%	7,22%	22,69%	0,69%	0,31%	2,85%	11,36%	22,20%	13,01%	1,89%	4,25%	1,41%	0,92%	1,51%	0,71%	0,80%
907	4	0,37%	1,75%	1,59%	0,79%	7,55%	21,01%	1,14%	0,55%	3,64%	7,05%	14,37%	17,79%	2,63%	5,28%	1,74%	0,95%	1,94%	0,96%	1,11%
350	4	0,95%	2,22%	1,85%	1,47%	8,32%	19,67%	2,11%	0,65%	3,03%	5,94%	12,80%	12,53%	1,57%	3,54%	1,33%	0,74%	0,92%	0,53%	0,68%
1313	5	0,31%	2,22%	0,94%	0,54%	9,31%	26,11%	1,31%	0,35%	2,64%	7,55%	19,42%	13,09%	1,47%	4,44%	1,50%	0,64%	0,79%	0,76%	1,09%
1180	5	0,70%	2,67%	1,62%	1,37%	9,68%	17,89%	1,55%	1,08%	6,08%	8,69%	12,92%	7,33%	3,85%	5,98%	1,01%	0,57%	0,70%	0,81%	0,51%
261	5	0,71%	3,27%	1,42%	0,97%	8,33%	18,98%	2,11%	0,64%	3,30%	8,42%	13,42%	8,58%	1,93%	4,70%	0,99%	0,59%	1,08%	0,81%	1,07%
644	5	0,48%	2,84%	1,42%	0,88%	9,08%	21,00%	2,09%	0,50%	4,68%	12,00%	18,57%	9,44%	2,92%	5,27%	0,91%	0,54%	0,60%	0,73%	0,47%
264	5	0,54%	2,34%	2,03%	0,94%	10,76%	17,58%	1,92%	0,66%	5,14%	7,30%	11,66%	8,35%	3,28%	8,31%	1,49%	0,54%	1,11%	1,06%	1,05%
347	6	0,33%	1,86%	1,84%	0,65%	13,75%	20,19%	2,11%	0,42%	4,38%	10,12%	16,75%	10,28%	2,10%	6,28%	1,79%	0,66%	0,86%	1,23%	1,17%
316	6	0,44%	2,77%	2,37%	0,97%	10,76%	23,35%	2,60%	0,66%	4,86%	9,50%	15,98%	8,60%	2,69%	4,94%	1,03%	0,52%	0,53%	0,95%	0,78%
1323	6	0,46%	2,45%	1,33%	0,89%	10,39%	24,48%	1,08%	0,61%	5,01%	4,78%	12,08%	13,76%	2,12%	6,74%	1,12%	0,83%	0,91%	1,24%	1,54%
235	6	0,84%	3,39%	1,74%	1,30%	10,83%	21,03%	2,28%	1,05%	5,78%	7,73%	12,98%	7,15%	3,68%	5,49%	1,01%	0,46%	0,59%	1,09%	0,90%
721	6	0,60%	3,36%	1,92%	1,28%	11,62%	23,72%	1,13%	0,94%	4,52%	11,23%	16,36%	6,02%	2,47%	3,50%	0,74%	0,42%	0,83%	0,53%	0,39%
345	6	0,51%	2,05%	1,81%	0,50%	5,92%	24,14%	1,14%	0,54%	2,41%	4,14%	15,96%	15,37%	2,63%	6,23%	2,74%	1,18%	1,38%	1,64%	1,64%
603	6	0,74%	3,93%	3,19%	0,84%	6,44%	28,29%	2,44%	0,61%	4,03%	3,73%	11,55%	12,73%	2,15%	4,16%	1,28%	0,90%	0,59%	0,84%	0,82%
269	6	0,29%	2,97%	1,55%	0,73%	7,78%	26,98%	1,87%	0,39%	4,50%	7,48%	21,91%	9,29%	3,62%	4,23%	0,77%	0,63%	0,77%	0,50%	0,34%
273	7	0,42%	2,28%	1,30%	0,75%	8,86%	22,81%	1,94%	0,54%	3,53%	7,88%	16,82%	11,80%	2,63%	4,66%	1,35%	0,65%	0,33%	0,62%	0,55%
706	7	0,35%	2,49%	0,95%	0,93%	12,22%	27,19%	0,95%	0,35%	3,69%	13,15%	20,69%	9,17%	1,90%	2,56%	0,61%	0,39%	0,34%	0,32%	0,20%
674	7	0,53%	2,05%	1,71%	1,30%	12,41%	21,44%	1,45%	0,43%	3,94%	14,95%	19,27%	7,59%	2,24%	2,93%	0,71%	0,40%	0,57%	0,28%	0,36%
697	7	0,48%	2,20%	1,62%	0,81%	9,86%	22,57%	1,25%	0,37%	4,02%	9,35%	17,38%	10,31%	1,73%	3,86%	0,93%	0,66%	0,68%	0,54%	0,55%
275	7	0,41%	2,56%	1,44%	0,86%	9,25%	21,83%	1,06%	0,86%	5,61%	10,62%	16,69%	8,80%	4,23%	6,23%	1,45%	0,76%	1,62%	0,75%	0,54%
304	7	0,45%	3,19%	1,49%	1,06%	13,12%	27,36%	1,33%	0,65%	5,15%	7,81%	16,13%	9,04%	1,57%	4,57%	0,66%	0,44%	0,83%	0,44%	0,30%
544	7	0,33%	2,42%	1,30%	0,94%	12,99%	24,05%	1,39%	0,35%	3,02%	13,04%	19,67%	10,42%	1,47%	3,19%	0,64%	0,60%	0,34%	0,47%	0,42%
687	7	0,56%	1,63%	1,20%	1,08%	10,72%	21,11%	0,96%	0,47%	2,97%	12,89%	22,86%	9,14%	1,23%	1,81%	0,73%	0,69%	0,33%	0,34%	0,27%
1208	7	0,62%	3,43%	2,62%	1,19%	12,91%	29,28%	1,98%	0,74%	3,95%	6,49%	14,90%	9,77%	1,34%	2,82%	0,69%	0,43%	0,87%	0,41%	0,30%
266	8	0,95%	4,58%	1,59%	1,46%	12,69%	24,13%	0,71%	1,09%	5,90%	8,17%	13,23%	6,27%	2,62%	4,35%	0,49%	0,32%	0,85%	0,63%	0,38%
487	8	0,79%	3,48%	1,57%	1,42%	12,52%	20,54%	1,75%	0,85%	3,70%	8,16%	12,86%	9,59%	1,66%	4,97%	0,92%	0,50%	0,64%	0,47%	0,76%
548	8	0,41%	2,08%	1,17%	1,16%	12,34%	19,35%	1,56%	0,49%	3,22%	14,11%	20,24%	10,26%	1,95%	3,69%	0,88%	0,55%	0,70%	0,45%	0,46%
346	8	1,08%	3,24%	2,61%	1,02%	10,61%	19,25%	3,50%	0,65%	3,26%	7,71%	12,97%	6,03%	1,17%	2,86%	0,74%	0,46%	1,14%	0,62%	0,58%
1300	9	0,56%	1,00%	1,64%	0,49%	8,68%	20,87%	0,75%	0,43%	2,72%	7,84%	16,16%	15,24%	1,43%	4,30%	1,92%	1,31%	1,88%	0,97%	1,03%
199	9	0,55%	6,91%	2,41%	1,01%	7,04%	21,98%	2,68%	0,23%	4,37%	2,66%	8,71%	6,57%	1,65%	3,66%	0,95%	0,38%	0,39%	0,53%	0,89%
719	9	0,37%	3,28%	1,26%	1,32%	13,20%	25,84%	0,96%	0,48%	3,63%	12,54%	17,60%	8,31%	1,82%	2,87%	0,94%	0,39%	0,65%	0,58%	0,48%
393	9	0,56%	5,17%	0,67%	1,07%	11,56%	29,05%	0,41%	0,72%	7,82%	3,74%	6,30%	8,76%	2,43%	6,27%	1,64%	0,44%	0,59%	1,14%	1,25%
541	9	1,14%	2,64%	2,17%	1,26%	6,76%	15,97%	2,25%	0,59%	2,76%	6,22%	11,91%	10,82%	1,69%	5,84%	1,58%	0,67%	0,93%	1,42%	1,35%
258	9	0,10%	0,47%	0,59%	0,17%	3,23%	15,28%	0,78%	0,18%	1,11%	2,29%	12,49%	39,21%	1,13%	6,84%	3,43%	1,21%	3,97%	1,74%	2,14%
715	9	0,62%	1,33%	1,12%	0,58%	4,98%	19,46%	0,71%	0,47%	1,47%	2,43%	9,74%	22,32%	1,02%	4,27%	2,57%	0,83%	2,46%	1,24%	2,29%
253	9	0,32%	0,50%	0,27%	1,61%	18,52%	6,44%	0,31%	1,10%	6,82%	10,44%	25,90%	5,06%	3,60%	9,81%	1,28%	0,39%	1,52%	1,38%	0,54%
636	9	1,10%	1,94%	2,11%	1,26%	6,52%	16,98%	2,43%	0,55%	1,67%	5,06%	11,78%	7,45%	1,08%	1,59%	0,83%	0,51%	0,00%	0,55%	0,68%
675	9	0,28%	1,62%	0,58%	0,23%	3,54%	27,56%	0,35%	0,28%	2,36%	1,66%	11,48%	17,18%	2,13%	7,11%	3,23%	1,28%	4,53%	2,30%	3,68%
1326	9	0,39%	2,30%	2,85%	1,45%	8,92%	18,93%	1,75%	1,08%	7,09%	7,58%	12,58%	6,85%	5,08%	7,44%	1,01%	0,37%	1,66%	0,75%	0,64%
68	9	0,71%	1,97%	1,30%	1,21%	7,88%	20,19%	1,63%	0,83%	2,77%	7,84%	15,46%	7,74%	1,67%	2,78%	0,85%	0,85%	0,00%	0,28%	0,31%
227	9	0,36%	3,36%	1,22%	2,23%	13,25%	18,16%	0,00%	0,90%	4,80%	5,56%	9,41%	7,10%	1,25%	3,35%	0,52%	0,51%	0,54%	0,66%	0,49%
594	9	0,68%	2,71%	1,48%	1,15%	8,60%	20,39%	2,40%	0,71%	3,59%	5,94%	13,66%	10,02%	2,16%	4,68%	0,85%	0,77%	0,56%	0,91%	0,99%
659	9	0,42%	2,69%	0,99%	1,07%	6,50%	17,12%	1,12%	0,89%	2,56%	3,10%	12,49%	30,80%	1,61%	5,35%	1,43%	1,08%	0,71%	1,01%	1,08%
507	9	1,30%	1,39%	1,76%	1,20%	3,71%	11,07%	1,34%	0,97%	2,97%	3,17%	5,61%	9,08%	1,78%	5,40%	0,86%	1,02%	1,80%	1,06%	1,45%
451	9	0,48%	1,17%	1,54%	0,51%	4,55%	21,82%	1,00%	0,33%	1,35%	2,56%	10,48%	19,31%	1,38%	4,82%	3,54%	1,67%	2,22%	1,89%	3,71%
454	9	0,47%	2,34%	1,49%	0,50%	4,65%	25,57%	1,38%	0,50%	2,35%	2,09%	9,78%	18,36%	1,54%	5,60%	2,88%	1,23%	2,52%	0,95%	2,07%
641	9	0,28%	1,47%	1,39%	0,33%	4,11%	21,57%	1,06%	0,24%	2,04%	2,19%	12,06%	29,77%	1,63%	6,29%	2,40%	1,18%	2,32%	1,72%	2,91%
336	9	0,00%	1,21%	0,00%	0,00%	7,06%	11,10%	1,20%	0,06%	0,00%	3,45%	6,70%	6,21%	1,25%	4,00%	0,86%	0,60%	0,38%	0,51%	1,95%
240	9	0,37%	2,67%	2,35%	0,66%	6,97%	29,14%	4,40%	0,33%	1,21%	1,61%	10,43%	24,02%	0,65%	2,52%	1,34%	1,05%	0,50%	0,32%	0,70%
571	10	0,37%	1,54%	1,38%	0,86%	10,22%	19,81%	1,68%	0,46%	4,06%	15,84%	20,59%	8,49%	2,24%	3,15%	0,83%	0,56%	0,38%	0,27%	0,40%
490	10	0,55%	2,67%	2,05%	1,13%	10,88%	23,59%	0,90%	0,59%	4,40%	6,64%	12,89%	13,94%	1,51%	4,71%	1,03%	0,71%	0,72%	0,71%	0,85%
429	10	0,27%	1,13%	1,08%	0,29%	2,59%	26,17%	0,81%	0,29%	1,39%	1,83%	12,53%	30,39%	1,08%	4,53%	2,53%	1,42%	1,25%	1,17%	1,72%
267	10	1,04%	3,62%	2,42%	1,09%	8,24%	22,83%	1,69%	1,00%	4,34%	5,28%	11,02%	7,17%	1,68%	3,46%	1,00%	0,46%	0,00%	0,65%	0,52%
306	10	0,21%	1,41%	2,02%	0,86%	7,91%	18,18%	1,51%	0,31%	3,17%	18,93%	28,85%	9,02%	1,76%	1,76%	0,71%	0,79%	0,30%	0,28%	0,24%
707	10	0,48%	3,41%	2,04%	1,13%	11,44%	24,52%	1,39%	1,13%	5,06%	5,77%	13,61%	14,81%	1,71%	7,55%	1,48%	0,52%	0,48%	0,57%	0,76%
207	10	0,31%	2,26%	1,06%	0,72%	10,01%	22,45%	0,79%	0,47%	2,80%	5,48%	17,37%	13,76%	1,84%	4,61%	1,60%	0,70%	0,72%	1,19%	1,24%
1318	11	0,57%	2,01%	2,81%	0,84%	9,45%	23,76%	1,70%	0,59%	2,76%	4,80%	11,56%	15,48%	1,79%	4,63%	2,65%	0,99%	1,12%	2,00%	4,47%
691	11	0,32%	1,98%	1,79%	0,53%	6,51%	25,73%	1,28%	0,52%	4,02%	3,59%	13,96%	16,24%	2,75%	5,68%	1,39%	1,14%	1,32%	1,78%	1,57%
494	11	1,26%	5,49%	1,59%	1,67%	12,38%	22,96%	1,42%	1,10%	5,37%	7,32%	12,09%	7,49%	2,63%	5,51%	1,40%	0,39%	0,66%	0,66%	0,74%
502	11	0,74%	1,79%	2,10%	1,04%	9,51%	28,10%	0,51%	0,93%	6,54%	4,36%	8,28%	8,24%	3,00%	5,68%	0,65%	0,44%	0,95%	1,15%	0,97%
651	11	0,69%	2,81%	1,81%	0,92%	7,30%	18,80%	2,41%	0,52%	5,98%	5,58%	10,99%	10,27%	3,08%	10,73%	1,57%	0,62%	0,48%	1,23%	1,43%
552	11	0,50%	4,64%	2,24%	0,64%	8,57%	28,80%	1,77%	0,60%	4,30%	3,51%	13,82%	13,94%	1,92%	4,47%	0,87%	0,48%	0,81%	0,95%	0,81%
666	11	0,30%	2,68%	1,00%	0,97%	9,73%	24,58%	0,81%	0,61%	5,22%	9,84%	19,26%	8,48%	2,94%	4,75%	0,75%	0,43%	0,57%	0,52%	0,44%
713	12	0,33%	2,18%	2,15%	0,74%	7,50%	20,79%	1,38%	0,86%	6,74%	10,04%	18,02%	7,28%	6,53%	6,76%	1,08%	0,47%	1,41%	0,60%	0,36%
692	12	0,39%	1,40%	1,42%	0,59%	7,55%	21,57%	0,63%	0,50%	2,77%	8,52%	18,72%	14,47%	2,02%	4,98%	1,65%	0,77%	1,14%	0,87%	1,35%
210	12	0,23%	1,49%	1,75%	0,39%	4,73%	18,35%	1,15%	0,88%	5,27%	2,07%	7,16%	16,20%	3,64%	13,41%	2,45%	0,60%	4,44%	2,63%	3,62%
650	12	0,42%	1,89%	1,97%	0,99%	10,12%	19,60%	1,49%	0,61%	4,30%	11,40%	17,79%	14,66%	2,48%	4,72%	1,57%	0,72%	0,69%	0,74%	0,68%
460	12	0,39%	1,51%	1,35%	0,91%	7,95%	20,17%	1,16%	0,56%	3,61%	13,72%	19,92%	11,35%	2,20%	4,06%	1,28%	0,83%	0,81%	0,65%	0,61%
717	12	0,59%	3,17%	1,19%	1,13%	10,19%	23,44%	1,10%	0,63%	3,54%	4,88%	12,59%	11,90%	1,83%	4,94%	1,52%	0,82%	2,43%	0,80%	1,36%
1174	12	0,62%	4,59%	2,73%	0,51%	5,83%	32,49%	1,44%	0,57%	3,09%	1,48%	8,50%	17,08%	0,84%	4,41%	1,57%	0,68%	1,98%	0,64%	0,95%
395	12	0,51%	1,61%	1,37%	0,50%	5,24%	15,12%	1,69%	0,43%	3,92%	2,35%	9,59%	17,28%	1,97%	12,73%	2,32%	0,52%	1,00%	1,52%	2,32%
455	12	0,78%	1,81%	1,81%	0,80%	8,21%	18,75%	1,14%	0,63%	3,78%	5,15%	11,70%	9,35%	2,74%	5,47%	0,96%	0,99%	0,68%	1,48%	1,00%
472	12	0,27%	2,87%	2,49%	0,59%	7,20%	27,85%	1,25%	0,48%	3,79%	4,39%	13,13%	13,71%	1,93%	4,59%	1,26%	0,63%	3,01%	0,88%	1,57%
303	12	0,61%	3,03%	1,44%	0,91%	6,66%	23,02%	0,82%	0,70%	5,26%	5,35%	11,75%	14,84%	3,67%	6,15%	2,44%	0,87%	1,90%	1,16%	1,42%
197	12	0,58%	1,88%	2,21%	0,72%	7,30%	23,01%	1,28%	0,42%	2,05%	3,48%	12,46%	22,05%	0,79%	4,80%	1,72%	0,92%	2,29%	0,94%	1,29%
723	12	0,45%	2,14%	1,45%	0,74%	12,50%	20,36%	0,56%	0,71%	4,79%	6,06%	12,75%	14,67%	2,47%	8,28%	1,47%	0,56%	1,26%	1,19%	1,52%

No.	Group No.	PE P 16:0/16:1	PE P 16:0/16:0	PE P 16:0/18:2	PE P 16:0/18:1	PE P 16:0/20:4	PE P 16:0/20:3	PE P 16:0/22:6	PE P 16:0/22:5	PE P 16:0/22:4	PE P 18:1/16:0	PE P 18:1/18:2	PE P 18:1/18:1	PE P 18:1/20:5	PE P 18:1/20:4	PE P 18:1/22:6	PE P 18:0/16:0	PE P 18:0/18:2	PE P 18:0/18:1	PE P 18:0/20:4
380	1	1,09%	0,84%	2,02%	7,83%	14,83%	1,28%	2,61%	2,54%	3,00%	1,26%	1,16%	3,63%	0,75%	7,62%	1,19%	1,15%	2,78%	6,86%	18,58%
649	1	1,18%	1,41%	2,83%	7,01%	11,79%	2,40%	2,26%	1,80%	1,56%	1,31%	2,51%	4,69%	1,26%	7,05%	1,25%	1,93%	5,26%	8,71%	12,00%
566	1	1,13%	4,01%	5,69%	9,76%	6,13%	0,95%	1,23%	1,11%	1,02%	9,62%	4,61%	8,04%	0,88%	5,14%	0,77%	5,00%	5,98%	9,35%	5,93%
1349	1	0,83%	1,02%	3,93%	5,81%	9,84%	1,33%	3,57%	1,56%	2,58%	1,14%	1,68%	2,89%	1,12%	5,64%	1,87%	1,60%	8,31%	7,48%	16,58%
386	1	1,46%	0,91%	3,77%	5,66%	19,07%	1,65%	1,31%	1,87%	4,53%	1,56%	1,82%	3,14%	1,60%	6,55%	1,35%	1,60%	3,67%	4,20%	11,37%
206	1	0,96%	1,07%	2,15%	4,64%	16,29%	1,67%	2,63%	1,79%	4,39%	1,88%	1,51%	2,74%	1,19%	9,44%	1,25%	1,48%	2,36%	5,00%	16,98%
399	1	1,10%	1,36%	1,47%	3,95%	14,35%	1,55%	3,85%	1,67%	3,21%	0,90%	0,92%	2,19%	1,08%	4,68%	1,56%	1,95%	2,89%	7,80%	19,57%
725	1	1,38%	1,64%	3,53%	10,20%	10,60%	2,89%	3,23%	3,38%	3,87%	1,73%	1,44%	3,50%	1,40%	5,26%	1,47%	1,79%	3,48%	4,82%	7,16%
560	1	0,92%	1,00%	2,56%	4,71%	11,41%	2,34%	2,01%	1,93%	2,92%	1,27%	1,62%	2,82%	1,15%	6,35%	1,29%	2,29%	4,79%	6,11%	20,61%
436	1	0,69%	0,87%	3,43%	5,87%	10,54%	1,58%	3,85%	2,83%	2,67%	1,22%	2,35%	2,61%	0,71%	5,88%	1,44%	1,46%	7,52%	7,27%	16,30%
435	1	0,72%	0,51%	3,19%	9,75%	12,57%	1,83%	2,30%	1,83%	2,85%	1,04%	2,07%	3,46%	0,64%	5,19%	0,95%	1,40%	6,08%	9,58%	16,12%
357	2	0,87%	0,69%	1,65%	8,24%	11,19%	1,08%	2,47%	2,03%	1,92%	0,65%	1,27%	2,92%	0,89%	4,16%	1,02%	1,27%	3,80%	9,98%	23,16%
638	2	0,48%	1,43%	3,54%	9,48%	16,60%	2,74%	4,68%	1,81%	2,64%	1,77%	1,90%	3,06%	0,44%	8,53%	1,70%	1,58%	3,69%	5,44%	14,16%
727	2	0,70%	1,37%	2,52%	8,78%	14,69%	1,29%	3,00%	2,95%	3,04%	1,03%	1,42%	2,42%	0,99%	5,36%	1,08%	1,47%	3,54%	6,25%	16,40%
252	2	0,58%	0,76%	4,05%	11,97%	12,69%	1,72%	2,84%	2,71%	5,36%	0,81%	1,40%	3,12%	0,34%	4,07%	0,80%	0,90%	6,07%	10,71%	15,09%
549	2	1,49%	4,53%	3,18%	10,57%	17,16%	1,98%	2,59%	3,34%	3,57%	2,38%	0,84%	3,01%	0,71%	5,11%	0,94%	3,00%	2,96%	5,48%	11,03%
553	2	0,30%	0,47%	2,61%	14,03%	8,66%	1,80%	1,74%	2,43%	2,67%	0,49%	1,05%	3,26%	0,21%	2,62%	0,44%	0,70%	6,13%	15,65%	17,33%
1227	2	0,52%	0,59%	2,05%	9,13%	12,01%	1,34%	3,83%	3,81%	2,97%	0,79%	0,82%	2,40%	0,60%	4,14%	1,00%	0,95%	4,05%	10,39%	18,45%
563	2	0,42%	0,53%	3,02%	16,75%	9,90%	1,41%	2,58%	2,07%	2,91%	0,80%	1,34%	5,04%	0,25%	3,85%	0,75%	0,79%	5,84%	14,64%	14,39%
378	2	1,50%	1,62%	3,94%	8,66%	9,09%	1,88%	1,91%	1,85%	2,56%	1,41%	1,94%	2,82%	1,08%	5,08%	0,99%	1,95%	6,05%	9,72%	13,88%
344	2	0,60%	1,54%	1,69%	10,66%	24,98%	1,28%	2,03%	2,43%	5,39%	1,18%	0,46%	1,91%	0,28%	2,41%	0,31%	0,97%	2,29%	7,55%	19,35%
202	3	0,68%	1,26%	1,74%	11,42%	16,20%	2,48%	7,76%	4,10%	10,53%	1,52%	0,78%	2,73%	0,31%	3,85%	1,62%	0,72%	1,06%	4,13%	8,42%
1178	3	0,68%	1,05%	1,17%	9,59%	19,19%	1,16%	7,40%	1,82%	3,91%	1,41%	0,51%	1,92%	0,77%	4,71%	1,74%	0,93%	1,38%	5,75%	14,35%
612	4	1,31%	1,13%	3,29%	13,45%	8,33%	1,65%	6,07%	2,04%	2,84%	1,61%	1,36%	2,04%	1,31%	2,54%	1,26%	1,10%	4,72%	5,36%	9,34%
550	4	0,87%	1,79%	3,90%	7,24%	23,19%	2,17%	2,30%	3,85%	5,29%	2,77%	1,25%	2,40%	0,72%	7,25%	0,69%	1,10%	2,26%	3,51%	12,32%
639	4	0,57%	0,88%	3,15%	11,75%	19,54%	2,07%	6,65%	3,10%	5,74%	1,04%	0,91%	2,50%	0,29%	4,73%	1,15%	0,61%	2,04%	5,41%	12,31%
818	4	2,84%	2,88%	2,53%	2,80%	3,84%	1,75%	2,28%	2,36%	2,26%	2,04%	1,88%	2,26%	3,33%	2,83%	3,21%	2,35%	2,56%	3,39%	3,75%
495	4	0,75%	1,76%	5,11%	10,02%	11,66%	2,27%	1,46%	1,93%	4,19%	2,74%	3,48%	5,36%	0,75%	6,41%	0,64%	1,66%	5,36%	8,84%	10,02%
1167	4	0,35%	0,75%	3,11%	15,28%	14,87%	1,71%	12,68%	4,12%	3,34%	1,16%	0,92%	2,00%	0,47%	2,43%	1,56%	0,88%	2,22%	6,31%	9,78%
885	4	0,62%	1,63%	7,62%	8,71%	15,74%	4,13%	2,21%	1,22%	1,62%	1,87%	2,48%	2,87%	0,49%	3,84%	0,44%	2,34%	7,67%	7,56%	10,91%
256	4	0,64%	1,46%	4,17%	6,30%	20,00%	2,23%	2,28%	1,99%	4,50%	2,60%	2,09%	3,27%	0,82%	10,12%	0,89%	1,44%	3,31%	4,34%	13,54%
260	4	0,93%	1,57%	1,69%	7,42%	29,55%	2,06%	3,85%	3,40%	4,23%	1,11%	0,48%	1,91%	0,53%	6,15%	0,87%	1,09%	1,37%	2,99%	13,74%
600	4	0,77%	1,54%	2,28%	11,13%	16,11%	1,89%	4,94%	3,32%	5,47%	1,15%	0,68%	2,52%	0,71%	3,80%	1,30%	1,19%	2,25%	6,62%	10,63%
1299	4	0,87%	1,66%	1,37%	10,28%	22,18%	1,94%	7,01%	2,58%	4,62%	1,62%	0,48%	2,67%	0,49%	5,14%	1,38%	1,11%	1,21%	4,80%	11,08%
653	4	1,01%	2,74%	5,27%	10,81%	16,16%	3,11%	2,45%	2,20%	3,68%	3,75%	2,67%	4,29%	0,92%	6,57%	0,82%	1,95%	3,38%	5,14%	8,45%
313	4	1,47%	1,68%	3,70%	10,39%	12,56%	3,09%	4,39%	2,65%	5,20%	2,20%	1,64%	3,39%	1,00%	5,39%	0,91%	1,47%	3,23%	5,16%	7,84%
396	4	0,50%	0,93%	2,55%	10,84%	20,47%	3,01%	4,81%	2,18%	4,09%	1,16%	0,91%	2,67%	0,39%	6,75%	1,24%	1,02%	2,62%	5,73%	14,05%
1340	4	0,92%	1,35%	2,02%	7,46%	18,32%	1,96%	7,60%	2,28%	3,57%	1,10%	1,14%	1,56%	1,18%	4,31%	1,61%	1,51%	2,50%	4,46%	13,14%
688	4	0,30%	0,62%	3,27%	13,50%	14,28%	1,83%	10,34%	1,99%	3,43%	1,22%	1,73%	3,96%	0,36%	4,18%	2,29%	0,84%	3,50%	6,43%	10,81%
907	4	0,38%	0,73%	2,66%	15,92%	12,07%	1,63%	6,52%	2,28%	2,99%	1,26%	0,85%	3,18%	0,38%	3,40%	1,55%	0,95%	3,05%	10,04%	12,57%
350	4	1,94%	1,94%	3,20%	10,29%	9,04%	3,51%	2,57%	2,07%	3,29%	1,85%	1,55%	2,70%	1,52%	2,76%	1,32%	2,03%	3,41%	7,55%	8,54%
1313	5	0,57%	0,56%	2,82%	11,30%	12,49%	1,66%	4,72%	3,76%	5,02%	0,78%	1,00%	2,46%	0,41%	2,42%	0,95%	0,83%	2,99%	9,37%	15,94%
1180	5	1,44%	1,71%	2,41%	4,62%	18,04%	1,64%	3,99%	3,63%	2,39%	1,21%	1,00%	2,86%	1,11%	6,31%	1,46%	1,22%	2,68%	3,44%	14,42%
261	5	1,19%	1,39%	1,95%	5,95%	11,72%	1,52%	3,72%	2,84%	3,71%	1,96%	1,87%	2,47%	1,16%	5,64%	1,30%	1,44%	4,25%	6,26%	13,43%
644	5	0,88%	1,45%	3,07%	5,52%	11,47%	1,92%	2,36%	2,43%	3,44%	1,63%	1,89%	2,96%	0,81%	6,43%	1,30%	2,46%	5,59%	7,45%	16,10%
264	5	1,02%	1,18%	2,00%	5,64%	21,24%	2,19%	3,74%	4,56%	4,55%	0,86%	1,04%	1,26%	0,83%	6,04%	1,20%	1,37%	2,41%	4,30%	15,69%
347	6	0,52%	1,65%	5,53%	7,43%	26,15%	2,81%	4,04%	3,94%	4,91%	1,23%	1,97%	1,90%	0,26%	5,90%	0,62%	1,03%	3,57%	3,40%	11,60%
316	6	1,30%	2,56%	4,38%	7,07%	23,08%	2,43%	1,61%	3,66%	4,80%	2,43%	2,07%	2,46%	0,85%	7,74%	0,65%	1,80%	2,92%	3,51%	10,96%
1323	6	0,66%	1,32%	4,17%	7,87%	29,72%	1,80%	4,09%	4,20%	6,10%	1,32%	0,98%	1,83%	0,69%	6,33%	0,91%	0,56%	1,63%	3,04%	10,63%
235	6	0,97%	1,29%	3,16%	5,58%	24,05%	2,71%	3,82%	3,33%	4,74%	1,05%	1,04%	1,38%	0,51%	6,40%	1,37%	1,15%	2,83%	3,52%	13,69%
721	6	1,03%	2,29%	4,47%	7,73%	16,64%	2,38%	3,63%	2,91%	3,34%	2,51%	2,57%	2,54%	1,46%	7,92%	1,53%	1,22%	2,92%	4,44%	8,42%
345	6	0,57%	1,37%	1,37%	10,42%	20,58%	2,44%	5,06%	3,46%	4,45%	2,36%	0,70%	2,84%	0,55%	7,95%	1,39%	0,99%	1,07%	4,80%	12,23%
603	6	1,52%	3,85%	3,21%	10,47%	20,18%	2,30%	2,72%	3,30%	3,44%	2,62%	0,94%	2,61%	0,42%	5,37%	0,71%	2,68%	2,60%	5,46%	12,15%
269	6	1,25%	2,19%	1,92%	6,97%	18,13%	2,26%	2,54%	2,56%	2,78%	3,35%	1,57%	4,08%	0,70%	9,67%	0,79%	1,82%	1,69%	6,31%	12,30%
706	7	0,46%	0,81%	5,52%	9,48%	11,29%	1,25%	1,43%	1,65%	3,06%	1,22%	3,34%	4,98%	0,44%	5,69%	0,54%	1,75%	9,79%	11,52%	13,11%
674	7	1,10%	1,00%	5,66%	7,70%	10,79%	1,81%	1,93%	1,44%	3,43%	1,46%	2,46%	3,06%	0,94%	4,66%	1,31%	2,20%	8,24%	9,33%	12,67%
697	7	1,12%	1,19%	2,16%	8,26%	10,17%	1,49%	3,48%	2,51%	4,00%	1,62%	1,33%	2,56%	1,20%	3,11%	1,09%	1,74%	4,58%	10,25%	11,76%
275	7	0,46%	0,95%	3,10%	6,50%	18,76%	2,87%	5,66%	1,73%	2,10%	1,47%	1,75%	2,53%	0,93%	10,04%	1,86%	1,20%	3,11%	4,48%	14,36%
304	7	0,72%	1,14%	3,28%	8,10%	13,32%	1,29%	3,97%	1,94%	2,25%	1,28%	1,34%	2,68%	0,55%	3,74%	1,22%	2,17%	7,21%	9,72%	15,41%
544	7	0,64%	0,80%	4,72%	10,80%	20,81%	1,54%	1,91%	2,34%	6,20%	0,95%	2,00%	3,51%	0,36%	6,67%	0,58%	0,72%	3,75%	5,84%	14,18%
687	7	1,21%	1,35%	5,22%	9,63%	5,79%	1,53%	2,23%	1,76%	2,50%	2,17%	3,05%	5,90%	1,11%	2,96%	1,18%	2,14%	8,90%	9,68%	7,51%
1208	7	0,78%	1,51%	4,42%	8,96%	13,13%	1,62%	4,92%	1,68%	3,16%	1,17%	1,15%	2,09%	0,90%	5,69%	1,33%	1,78%	4,25%	5,97%	17,41%
266	8	1,22%	1,58%	2,18%	5,61%	17,85%	1,52%	3,34%	2,88%	4,34%	1,47%	1,70%	2,40%	0,92%	8,07%	1,64%	0,97%	3,81%	4,86%	14,44%
487	8	0,91%	0,95%	2,66%	6,23%	22,48%	1,26%	2,18%	2,53%	4,44%	0,82%	1,14%	1,48%	0,75%	5,26%	1,04%	0,82%	2,60%	3,76%	18,45%
548	8	0,76%	1,48%	3,75%	4,58%	14,77%	1,25%	3,00%	2,43%	2,84%	2,20%	2,75%	3,00%	1,07%	7,09%	1,16%	2,31%	5,47%	6,02%	15,54%
346	8	2,12%	2,06%	3,36%	7,67%	10,46%	1,61%	3,90%	1,90%	3,89%	2,53%	2,06%	2,69%	1,76%	3,32%	1,88%	2,66%	2,84%	5,68%	6,66%
1300	9	0,42%	0,78%	3,22%	13,96%	12,26%	1,45%	8,84%	2,93%	4,36%	1,74%	1,77%	3,22%	0,79%	3,05%	2,03%	1,24%	3,87%	6,05%	9,42%
719	9	0,68%	0,85%	3,90%	10,93%	12,52%	1,25%	5,06%	2,98%	4,30%	1,16%	2,08%	3,89%	0,37%	4,57%	1,18%	1,53%	5,67%	7,55%	12,43%
393	9	1,71%	1,15%	3,93%	6,57%	36,94%	5,82%	2,89%	3,98%	3,41%	0,40%	0,36%	0,54%	0,26%	2,81%	0,34%	0,34%	1,53%	1,63%	12,53%
541	9	1,00%	1,22%	3,10%	7,85%	14,87%	2,72%	3,54%	2,82%	3,14%	1,80%	1,76%	2,10%	1,57%	5,01%	1,62%	1,46%	3,32%	4,44%	12,51%
715	9	0,64%	1,05%	3,18%	16,17%	12,63%	1,76%	7,63%	2,50%	4,02%	0,73%	0,57%	2,56%	0,50%	2,39%	1,39%	0,62%	3,18%	7,51%	11,19%
253	9	0,13%	0,12%	6,65%	2,74%	11,70%	1,04%	5,36%	7,41%	1,37%	0,80%	4,80%	2,28%	1,42%	8,25%	2,16%	0,20%	8,67%	2,47%	14,86%
636	9	2,47%	1,97%	2,46%	6,13%	5,02%	1,90%	1,96%	1,41%	2,51%	3,33%	2,16%	2,44%	2,46%	3,17%	1,95%	5,15%	3,49%	7,00%	3,93%
675	9	0,49%	0,48%	1,03%	14,15%	15,38%	1,64%	13,17%	3,33%	7,98%	0,90%	0,30%	2,98%	0,11%	3,80%	2,59%	0,27%	0,50%	4,48%	8,99%
1326	9	0,77%	1,70%	2,26%	5,25%	22,03%	1,67%	6,26%	2,86%	4,13%	1,70%	0,82%	1,74%	0,85%	8,29%	1,92%	1,46%	2,05%	3,08%	13,65%
68	9	1,91%	1,81%	3,29%	3,71%	7,50%	1,83%	2,63%	2,29%	2,09%	2,42%	2,32%	2,77%	2,36%	4,77%	2,37%	2,26%	3,06%	4,60%	7,45%
594	9	1,26%	2,42%	3,21%	5,15%	14,58%	1,83%	2,98%	2,10%	2,82%	1,27%	1,19%	2,74%	1,34%	5,68%	1,20%	1,52%	3,07%	4,51%	17,79%
659	9	0,33%	0,30%	4,04%	17,64%	9,47%	1,11%	1,59%	1,98%	1,97%	0,33%	1,39%	3,73%	0,24%	2,73%	0,27%	0,27%	9,07%	17,89%	15,31%
507	9	1,49%	1,57%	1,71%	7,22%	17,12%	1,18%	2,73%	2,86%	4,79%	1,36%	1,05%	1,29%	1,50%	4,38%	1,19%	1,19%	2,04%	2,81%	13,45%
451	9	0,39%	0,98%	1,87%	14,76%	12,22%	2,08%	7,49%	4,15%	7,30%	1,00%	0,40%	2,70%	0,36%	3,13%	1,66%	0,59%	1,78%	7,10%	8,91%
454	9	0,62%	1,27%	1,32%	17,38%	12,54%	2,20%	9,50%	3,15%	4,76%	1,12%	0,53%	4,29%	0,33%	4,14%	2,51%	0,61%	1,17%	6,59%	8,11%
641	9	0,49%	0,94%	2,80%	15,56%	14,54%	1,27%	7,61%	2,55%	6,03%	1,08%	0,77%	2,84%	0,08%	3,33%	1,56%	0,71%	4,01%	7,77%	11,66%
240	9	1,61%	5,08%	4,18%	16,48%	12,50%	2,72%	2,24%	1,70%	3,19%	3,04%	0,57%	2,14%	0,40%	1,58%	0,61%	4,64%	3,28%	9,05%	8,99%
571	10	0,79%	1,31%	7,80%	8,60%	10,92%	2,32%	2,86%	0,99%	2,07%	2,01%	4,29%	4,07%	0,73%	6,22%	1,32%	1,79%	7,93%	6,77%	11,27%
490	10	0,61%	1,34%	3,63%	10,62%	16,76%	1,89%	2,95%	1,75%	2,77%	1,38%	1,56%	2,91%	0,71%	6,00%	0,95%	1,25%	4,68%	7,05%	15,59%
429	10	0,59%	0,70%	1,47%	20,75%	15,03%	2,42%	4,93%	2,77%	4,46%	1,27%	0,48%	3,99%	0,26%	3,75%	1,19%	0,73%	1,38%	8,16%	10,48%
267	10	1,71%	1,55%	2,01%	5,60%	9,88%	2,27%	2,81%	2,43%	3,50%	1,67%	1,54%	1,99%	1,89%	3,68%	1,86%	2,34%	2,65%	4,77%	14,05%
306	10	0,37%	1,85%	3,49%	6,60%	7,40%	0,85%	1,25%	1,35%	2,05%	8,12%	6,36%	9,17%	1,23%	10,68%	0,82%	3,52%	5,93%	8,31%	8,71%
707	10	0,59%	1,56%	2,51%	8,50%	28,88%	2,89%	2,09%	3,03%	6,48%	1,42%	0,40%	1,55%	0,29%	2,64%	0,22%	1,13%	1,67%	3,97%	16,88%
207	10	0,69%	1,16%	2,49%	11,98%	13,98%	1,53%	4,86%	4,45%	5,17%	1,18%	0,94%	2,66%	0,54%	3,37%	1,33%	0,95%	2,80%	7,03%	12,86%
1318	11	0,52%	1,50%	2,85%	12,77%	14,73%	2,79%	3,25%	5,46%	11,51%	1,08%	0,75%	2,41%	0,25%	2,84%	0,64%	0,78%	1,82%	6,13%	9,31%
691	11	0,51%	1,16%	2,40%	6,84%	23,62%	2,06%	3,05%	5,40%	5,90%	1,74%	1,23%	2,27%	0,49%	6,80%	0,88%	0,86%	2,32%	4,09%	13,06%
494	11	1,65%	1,41%	3,50%	5,72%	23,63%	4,61%	1,82%	3,65%	5,23%	0,94%	1,28%	1,52%	0,76%	5,32%	0,72%	0,94%	3,24%	3,29%	12,99%
502	11	1,30%	1,22%	2,22%	7,80%	13,28%	1,60%	2,61%	2,70%	4,52%	2,30%	0,76%	2,90%	1,25%	4,29%	1,16%	1,29%	1,53%	5,16%	16,52%
651	11	0,63%	1,14%	1,85%	6,03%	24,71%	2,73%	1,55%	2,73%	5,92%	0,83%	0,76%	1,38%	0,56%	6,23%	0,70%	0,94%	2,21%	3,69%	19,59%
552	11	0,99%	1,28%	2,55%	9,03%	21,22%	1,75%	2,66%	3,13%	4,28%	1,16%	0,77%	2,48%	0,42%	4,37%	0,55%	1,24%	2,54%	7,08%	17,31%
666	11	0,82%	0,81%	2,69%	6,07%	12,19%	2,00%	2,48%	2,32%	2,72%	1,09%	1,56%	2,44%	0,87%	7,42%	1,62%	1,76%	4,07%	7,77%	19,34%
713	12	0,57%	1,66%	2,77%	4,77%	22,56%	2,74%	3,58%	1,58%	2,56%	2,40%	1,37%	2,38%	0,79%	11,03%	1,68%	1,60%	2,71%	4,00%	16,19%
692	12	0,51%	1,15%	3,16%	12,46%	17,75%	1,85%	6,89%	2,38%	5,32%	2,07%	1,34%	3,67%	0,61%	6,31%	1,43%	0,77%	1,96%	5,02%	9,91%
210	12	0,24%	0,95%	1,19%	6,64%	21,12%	1,26%	8,60%	3,91%	9,27%	1,35%	0,45%	1,27%	0,18%	5,96%	1,46%	1,05%	1,09%	2,67%	17,08%
650	12	0,40%	1,21%	5,64%	12,89%	15,76%	2,61%	3,24%	2,76%	4,10%	1,86%	1,96%	3,00%	0,43%	5,13%	0,76%	1,47%	4,63%	7,02%	11,54%
460	12	0,44%	0,93%	4,30%	11,07%	14,99%	2,77%	3,88%	2,46%	4,13%	1,76%	2,22%	2,90%	0,63%	5,53%	1,03%	0,95%	4,48%	6,21%	12,37%
717	12	0,98%	0,98%	2,09%	13,50%	14,65%	1,65%	8,04%	2,88%	3,64%	0,89%	1,02%	2,91%	0,77%	3,02%	1,63%	0,86%	1,98%	6,69%	11,72%
1174	12	1,15%	2,10%	1,88%	15,44%	13,53%	1,61%	8,21%	1,85%	4,28%	2,30%	0,56%	2,58%	0,68%	1,37%	1,04%	1,74%	1,99%	8,25%	10,01%
395	12	0,71%	0,74%	1,25%	6,39%	24,06%	1,47%	4,69%	3,49%	11,30%	0,87%	0,51%	1,73%	0,61%	5,10%	0,99%	0,56%	1,18%	2,66%	12,56%
455	12	1,18%	1,34%	3,93%	8,95%	15,97%	3,10%	3,52%	2,54%	3,87%	1,29%	1,93%	2,12%	0,86%	6,58%	1,29%	1,24%	3,03%	4,48%	11,24%
472	12	0,58%	1,10%	1,87%	13,67%	15,82%	1,49%	11,61%	2,54%	4,99%	1,06%	0,52%	2,55%	0,27%	2,51%	1,93%	0,78%	1,74%	6,13%	11,69%
303	12	0,63%	0,95%	2,00%	13,76%	16,28%	2,61%	6,77%	2,87%	4,65%	1,13%	0,54%	2,99%	0,27%	4,83%	1,98%	0,63%	1,66%	6,48%	11,66%
197	12	0,51%	1,32%	2,48%	16,76%	12,90%	1,85%	9,31%	2,25%	4,15%	1,62%	0,67%	2,38%	0,42%	2,37%	1,14%	0,96%	2,90%	8,32%	10,65%
723	12	0,70%	0,78%	2,41%	14,54%	22,20%	1,40%	5,65%	2,68%	6,11%	0,92%	0,57%	1,94%	0,33%	3,69%	1,14%	0,65%	1,70%	4,32%	12,83%

No.	Group No.	PS 32:1	PS 32:0	PS 34:2	PS 34:1	PS 34:0	PS 36:4	PS 36:3	PS 36:2	PS 36:1	PS 38:5	PS 38:4	PS 38:3	PS 38:2	PS 38:1	PS 40:6	PS 40:5	PS 40:4	PS 40:3
380	1	0,35%	0,36%	0,87%	4,67%	0,49%	0,88%	1,38%	10,04%	40,55%	0,74%	6,40%	2,38%	2,69%	2,47%	1,86%	1,60%	2,04%	0,97%
649	1	0,74%	0,54%	1,76%	6,45%	0,36%	1,98%	3,07%	16,48%	33,60%	1,58%	6,91%	3,05%	2,36%	2,14%	0,90%	1,30%	1,28%	0,76%
566	1	0,87%	2,50%	1,92%	8,67%	1,87%	0,56%	2,71%	20,09%	41,16%	0,50%	2,89%	2,88%	3,86%	3,58%	0,57%	0,64%	0,84%	0,84%
1349	1	0,26%	0,41%	1,28%	3,43%	0,59%	1,16%	2,41%	17,03%	34,55%	1,21%	8,53%	4,36%	2,59%	2,37%	2,97%	1,41%	2,56%	1,00%
386	1	0,39%	0,47%	1,33%	4,10%	0,58%	1,18%	2,03%	13,45%	31,72%	1,01%	5,85%	2,34%	1,86%	1,63%	1,05%	1,19%	1,39%	0,75%
206	1	0,55%	0,56%	0,96%	4,63%	0,76%	1,20%	2,12%	13,07%	35,91%	1,20%	9,47%	3,02%	2,27%	2,81%	1,15%	1,11%	1,60%	0,53%
399	1	0,39%	0,34%	1,01%	4,16%	0,30%	1,65%	1,99%	9,44%	30,03%	1,86%	9,96%	4,39%	1,95%	2,35%	1,35%	1,90%	1,84%	0,70%
725	1	0,59%	0,63%	1,23%	4,34%	0,42%	1,12%	1,37%	9,29%	31,85%	0,94%	4,00%	3,89%	1,54%	2,35%	0,91%	1,29%	1,70%	0,64%
560	1	0,38%	0,34%	1,41%	4,57%	0,60%	1,34%	2,22%	13,36%	31,15%	1,21%	9,06%	3,17%	1,99%	2,73%	1,71%	1,31%	1,87%	0,71%
436	1	0,30%	0,50%	1,55%	3,19%	0,41%	1,10%	2,52%	20,62%	33,57%	1,03%	7,30%	4,41%	2,22%	1,73%	1,47%	1,31%	1,59%	0,76%
435	1	0,36%	0,76%	1,29%	5,10%	0,24%	1,14%	2,16%	13,07%	38,05%	1,30%	9,35%	4,04%	2,23%	2,14%	1,73%	1,81%	2,22%	0,73%
357	2	0,53%	0,28%	1,53%	6,40%	0,24%	1,75%	2,28%	13,25%	36,93%	1,63%	8,99%	2,65%	1,79%	1,99%	1,22%	1,17%	1,55%	0,58%
638	2	0,50%	1,18%	1,60%	5,82%	0,82%	1,98%	2,74%	16,73%	35,00%	1,68%	12,78%	6,85%	2,13%	1,81%	2,20%	1,52%	1,86%	0,79%
727	2	0,47%	0,50%	1,11%	4,18%	0,36%	0,80%	1,75%	11,57%	37,32%	0,96%	6,38%	3,79%	2,17%	2,36%	2,44%	1,50%	2,00%	0,55%
252	2	0,23%	0,18%	1,08%	3,20%	0,40%	1,11%	1,63%	14,00%	46,22%	0,83%	6,37%	4,52%	2,18%	2,26%	2,02%	1,93%	2,92%	0,48%
549	2	1,22%	2,03%	1,42%	10,50%	1,32%	1,26%	1,68%	11,61%	36,30%	0,85%	6,95%	4,21%	2,18%	2,14%	2,06%	1,77%	2,19%	0,63%
553	2	0,26%	0,26%	0,81%	3,16%	0,46%	0,65%	1,43%	10,84%	55,68%	0,87%	4,91%	5,47%	1,70%	1,71%	1,57%	2,20%	2,09%	0,39%
1227	2	0,31%	0,21%	1,16%	5,05%	0,27%	1,46%	2,25%	13,51%	42,44%	1,79%	7,45%	4,83%	2,43%	2,71%	2,68%	2,64%	2,17%	0,57%
563	2	0,31%	0,24%	1,11%	4,22%	0,31%	0,88%	2,01%	13,24%	55,99%	0,55%	4,03%	3,44%	1,88%	1,66%	1,77%	1,61%	2,16%	0,50%
378	2	0,31%	0,33%	1,36%	4,68%	0,27%	1,19%	1,62%	13,63%	38,37%	0,78%	5,67%	2,63%	2,59%	3,00%	1,29%	1,29%	1,19%	0,63%
344	2	0,30%	0,80%	0,49%	5,19%	1,64%	0,41%	0,65%	8,21%	63,61%	0,29%	4,23%	3,42%	1,54%	2,00%	0,76%	1,35%	2,53%	0,55%
202	3	0,36%	1,27%	0,72%	4,77%	0,78%	0,69%	1,04%	8,53%	44,34%	0,53%	4,96%	6,06%	3,57%	2,62%	4,05%	2,16%	5,16%	1,16%
1178	3	0,51%	0,79%	0,74%	4,68%	0,57%	1,27%	0,89%	7,04%	40,77%	0,74%	5,92%	4,08%	2,77%	2,47%	4,05%	1,87%	3,04%	1,18%
612	4	0,48%	1,03%	0,86%	2,79%	0,79%	0,88%	0,95%	7,16%	36,32%	0,41%	2,90%	5,06%	2,50%	2,80%	3,98%	2,36%	2,21%	0,94%
550	4	0,71%	1,60%	1,83%	7,29%	1,16%	1,23%	2,45%	14,20%	35,25%	1,20%	8,44%	4,99%	3,16%	2,47%	1,48%	1,89%	2,83%	0,84%
639	4	0,36%	0,42%	1,19%	4,53%	0,33%	1,76%	1,49%	10,12%	43,63%	0,93%	7,78%	4,77%	2,89%	2,73%	4,63%	2,91%	4,92%	0,91%
818	4	0,49%	0,65%	0,81%	2,42%	0,49%	0,97%	1,55%	6,01%	27,87%	0,66%	3,03%	4,07%	1,79%	2,55%	2,64%	1,19%	2,04%	1,16%
495	4	0,36%	0,72%	2,89%	7,10%	0,53%	1,21%	4,18%	21,62%	35,74%	0,79%	4,56%	3,25%	2,52%	1,50%	1,04%	0,94%	1,45%	0,76%
1167	4	0,25%	0,53%	0,78%	3,01%	0,49%	0,92%	1,07%	9,61%	48,24%	0,58%	5,17%	5,72%	2,44%	2,70%	5,93%	1,95%	2,74%	0,82%
885	4	0,49%	1,02%	2,65%	7,09%	0,60%	1,98%	3,58%	24,95%	32,65%	0,97%	9,31%	6,36%	1,60%	1,66%	0,65%	0,81%	0,76%	0,58%
256	4	0,55%	1,20%	1,49%	6,17%	0,89%	1,28%	2,28%	13,89%	39,04%	1,01%	7,03%	5,41%	3,45%	3,02%	1,01%	1,66%	2,76%	1,09%
260	4	0,68%	1,06%	1,10%	5,97%	0,94%	1,25%	1,63%	9,33%	42,64%	0,94%	6,76%	5,36%	2,50%	2,54%	2,43%	2,25%	3,44%	0,64%
600	4	0,45%	0,80%	0,96%	3,63%	1,04%	0,93%	1,23%	7,19%	39,12%	0,77%	4,44%	6,60%	3,10%	3,91%	3,11%	2,07%	3,68%	1,71%
1299	4	0,43%	1,29%	0,80%	5,31%	1,32%	1,09%	1,06%	8,02%	43,63%	0,81%	7,28%	5,29%	2,37%	2,10%	3,88%	1,76%	3,23%	1,04%
653	4	0,79%	1,92%	2,17%	7,89%	1,21%	1,42%	3,35%	17,88%	38,07%	1,27%	6,96%	4,64%	2,92%	2,66%	0,75%	0,79%	1,59%	1,12%
313	4	0,48%	1,10%	1,33%	5,40%	0,56%	0,79%	2,10%	12,07%	35,81%	0,89%	4,46%	5,91%	3,20%	2,82%	1,06%	1,54%	2,41%	1,37%
396	4	0,33%	0,80%	0,77%	3,83%	0,55%	1,36%	1,69%	10,85%	42,62%	1,33%	12,41%	7,88%	2,88%	2,08%	2,96%	1,93%	3,06%	1,02%
1340	4	0,48%	0,82%	1,53%	5,18%	0,64%	1,22%	2,04%	13,09%	32,56%	1,63%	8,42%	4,56%	1,76%	2,08%	3,33%	1,27%	2,16%	0,99%
688	4	0,14%	0,50%	0,81%	3,31%	0,42%	0,68%	1,61%	11,84%	50,20%	0,74%	5,67%	4,24%	3,55%	2,56%	5,50%	1,53%	2,08%	0,89%
907	4	0,39%	0,55%	0,79%	2,82%	0,75%	0,62%	1,20%	9,04%	51,73%	0,59%	5,34%	4,63%	2,62%	2,44%	4,58%	1,66%	2,73%	0,77%
350	4	0,55%	0,76%	0,95%	4,58%	0,87%	0,83%	1,77%	9,62%	33,59%	0,48%	4,71%	4,46%	1,80%	2,09%	0,72%	1,29%	2,10%	1,00%
1313	5	0,4%	0,4%	1,2%	4,4%	0,4%	1,1%	1,6%	12,8%	43,3%	0,9%	5,8%	4,7%	3,4%	3,1%	2,1%	2,0%	2,9%	0,7%
1180	5	0,4%	0,6%	0,9%	4,0%	0,7%	1,4%	1,7%	11,2%	29,4%	1,2%	8,7%	4,8%	2,0%	2,3%	2,7%	1,6%	2,3%	1,3%
261	5	0,5%	0,7%	0,9%	5,7%	1,3%	1,0%	1,3%	9,7%	37,9%	0,8%	7,6%	3,7%	2,3%	2,1%	3,5%	1,7%	2,7%	0,9%
644	5	0,5%	0,6%	1,2%	5,8%	0,7%	1,4%	2,4%	15,6%	39,2%	1,0%	7,3%	4,0%	1,9%	2,1%	1,3%	1,5%	1,6%	1,1%
264	5	0,5%	0,4%	0,9%	4,7%	0,6%	1,4%	1,3%	10,4%	34,3%	1,3%	9,1%	5,3%	2,5%	2,5%	1,9%	2,4%	3,0%	0,6%
347	6	0,26%	0,60%	1,59%	4,99%	0,64%	0,92%	1,86%	15,38%	41,06%	0,56%	7,48%	7,43%	2,60%	2,25%	2,40%	2,81%	3,45%	0,68%
316	6	0,52%	0,94%	1,70%	5,92%	0,79%	1,89%	2,39%	15,82%	34,47%	1,11%	8,83%	5,07%	2,58%	1,92%	0,89%	2,26%	2,87%	0,99%
1323	6	0,62%	0,86%	1,45%	6,78%	0,96%	0,88%	1,73%	11,16%	42,37%	0,78%	6,15%	4,73%	3,21%	2,41%	1,86%	2,50%	2,95%	0,95%
235	6	0,53%	0,57%	1,52%	5,06%	0,65%	1,72%	2,27%	11,12%	32,08%	1,68%	9,62%	5,89%	2,10%	2,43%	2,83%	2,79%	3,44%	1,17%
721	6	0,62%	0,74%	1,53%	5,59%	0,82%	1,36%	3,09%	16,08%	33,94%	1,03%	6,81%	4,67%	2,32%	1,82%	2,00%	1,79%	2,07%	0,75%
345	6	0,34%	0,85%	0,45%	3,55%	0,88%	0,62%	0,81%	7,51%	45,95%	0,47%	3,98%	8,13%	3,85%	3,06%	3,60%	2,71%	3,77%	1,63%
603	6	0,92%	2,34%	1,57%	10,24%	1,58%	1,47%	1,55%	11,07%	40,41%	0,73%	6,95%	4,61%	2,22%	2,41%	1,58%	1,68%	2,14%	0,85%
269	6	0,51%	1,11%	1,24%	8,18%	0,34%	1,79%	2,17%	13,87%	39,84%	1,49%	7,25%	3,65%	2,82%	2,83%	1,13%	1,55%	1,96%	0,80%
273	7	0,53%	0,62%	1,69%	6,44%	0,46%	1,13%	2,06%	10,89%	39,38%	0,89%	7,88%	3,80%	2,82%	2,12%	1,28%	1,66%	2,20%	0,65%
706	7	0,26%	0,26%	2,35%	7,42%	0,11%	1,50%	3,47%	21,95%	38,96%	1,18%	8,62%	2,52%	2,39%	2,98%	0,73%	0,91%	1,06%	0,44%
674	7	0,38%	0,53%	2,27%	7,52%	0,17%	1,67%	3,16%	21,30%	30,60%	1,22%	8,35%	3,57%	1,91%	1,81%	2,47%	1,21%	0,99%	0,50%
697	7	0,41%	0,64%	1,44%	4,98%	0,49%	1,25%	1,75%	13,72%	34,90%	1,13%	6,57%	3,92%	2,77%	2,49%	2,35%	1,33%	1,97%	0,83%
275	7	0,43%	1,16%	1,03%	5,11%	0,83%	1,24%	2,44%	15,20%	36,79%	1,76%	10,50%	7,07%	3,37%	2,76%	2,52%	1,69%	2,11%	1,04%
304	7	0,43%	0,62%	2,04%	6,72%	0,39%	2,06%	2,34%	18,08%	33,22%	1,62%	11,74%	4,14%	2,02%	2,03%	1,50%	1,10%	1,66%	0,58%
544	7	0,23%	0,38%	1,82%	6,40%	0,36%	0,90%	2,60%	15,85%	48,43%	0,69%	4,35%	2,21%	2,53%	2,43%	1,20%	1,09%	2,29%	0,56%
687	7	0,40%	0,57%	1,37%	5,82%	0,51%	0,87%	2,77%	15,38%	35,25%	0,78%	5,45%	2,94%	2,57%	2,78%	1,40%	0,85%	1,63%	0,82%
1208	7	0,51%	0,65%	2,71%	8,22%	0,49%	2,40%	3,15%	17,96%	35,40%	1,19%	6,58%	2,72%	1,96%	2,32%	1,78%	1,08%	1,17%	0,67%
266	8	0,71%	0,52%	2,58%	6,93%	0,42%	2,10%	3,06%	15,17%	26,01%	1,95%	9,62%	3,79%	1,64%	1,55%	1,83%	1,65%	1,50%	0,77%
487	8	0,53%	0,33%	1,58%	4,69%	0,29%	1,47%	1,85%	12,23%	42,54%	0,99%	5,28%	2,82%	2,03%	1,37%	1,71%	1,34%	1,90%	1,06%
548	8	0,29%	0,54%	1,70%	4,69%	0,61%	1,10%	2,85%	19,56%	36,50%	1,17%	7,27%	4,41%	2,68%	2,24%	2,12%	1,53%	1,94%	1,08%
346	8	0,63%	0,68%	1,37%	4,69%	0,89%	1,04%	1,37%	9,65%	37,38%	0,82%	4,23%	3,81%	2,16%	2,04%	3,32%	1,64%	2,78%	0,49%
1300	9	0,29%	0,48%	0,78%	2,27%	0,53%	0,64%	0,88%	8,37%	50,13%	0,52%	3,24%	4,78%	3,40%	2,82%	4,65%	1,63%	2,15%	1,10%
199	9	3,63%	2,09%	2,14%	10,85%	2,22%	3,51%	0,00%	6,93%	24,50%	1,67%	7,16%	4,79%	1,10%	2,07%	1,58%	2,39%	1,38%	1,63%
719	9	0,49%	0,33%	2,47%	6,47%	0,29%	1,87%	3,14%	18,22%	37,67%	1,27%	6,39%	3,68%	2,58%	2,30%	1,81%	1,58%	2,11%	0,69%
393	9	1,48%	0,97%	1,51%	8,26%	0,70%	1,95%	2,22%	7,90%	31,17%	0,92%	10,25%	8,54%	2,19%	3,16%	2,26%	3,63%	3,71%	0,93%
541	9	0,35%	0,94%	1,11%	4,11%	0,55%	1,09%	1,67%	10,04%	35,29%	0,96%	5,81%	5,16%	2,23%	2,51%	2,16%	1,90%	1,99%	0,95%
258	9	0,04%	0,15%	0,17%	1,41%	0,61%	0,10%	0,38%	6,54%	71,25%	0,18%	2,82%	4,64%	2,46%	1,31%	3,13%	1,23%	2,23%	0,46%
715	9	0,33%	0,58%	0,67%	3,02%	0,66%	0,50%	0,72%	7,05%	50,01%	0,43%	3,58%	5,33%	3,00%	2,81%	3,92%	1,83%	3,27%	0,88%
253	9	0,76%	1,08%	2,99%	4,19%	0,85%	1,71%	2,81%	12,75%	24,56%	1,98%	16,35%	3,25%	1,75%	1,41%	3,11%	2,91%	4,18%	0,81%
636	9	0,43%	1,25%	1,51%	4,53%	1,26%	1,04%	3,05%	17,64%	32,76%	0,83%	5,44%	3,02%	2,95%	2,75%	0,80%	0,71%	1,15%	0,44%
675	9	0,12%	0,19%	0,20%	2,23%	0,22%	0,15%	0,33%	3,55%	54,22%	0,18%	2,30%	6,22%	5,16%	2,98%	7,58%	2,89%	6,15%	2,43%
1326	9	0,47%	1,55%	1,24%	5,01%	1,00%	2,25%	1,66%	12,52%	31,92%	1,66%	11,08%	4,92%	1,90%	1,59%	3,12%	2,16%	2,94%	0,68%
68	9	0,41%	0,69%	0,99%	6,32%	1,11%	1,21%	1,49%	10,26%	28,59%	1,12%	7,51%	2,66%	2,04%	1,73%	2,37%	1,99%	1,47%	0,78%
227	9	0,55%	0,00%	5,62%	7,34%	1,15%	2,25%	4,88%	11,52%	18,79%	1,81%	6,51%	2,56%	3,39%	4,17%	1,65%	3,20%	4,76%	0,68%
594	9	0,60%	0,51%	1,03%	3,29%	0,91%	0,97%	1,55%	9,17%	35,74%	0,93%	6,71%	3,84%	1,84%	3,25%	2,23%	1,53%	2,18%	0,84%
659	9	0,15%	0,21%	0,48%	2,25%	0,40%	0,33%	0,90%	11,17%	60,78%	0,42%	3,94%	4,07%	1,65%	1,49%	1,85%	1,51%	1,88%	0,54%
507	9	0,42%	0,68%	0,65%	3,19%	0,75%	1,13%	1,01%	8,59%	36,01%	0,74%	5,12%	3,55%	2,03%	1,83%	2,01%	1,83%	1,75%	0,71%
451	9	0,16%	0,28%	0,35%	1,66%	0,46%	0,21%	0,53%	4,92%	50,12%	0,29%	1,85%	7,03%	4,06%	3,34%	5,82%	3,43%	5,46%	1,61%
454	9	0,30%	0,55%	0,35%	2,08%	0,36%	0,46%	0,54%	4,80%	52,02%	0,32%	3,23%	7,15%	4,00%	2,58%	5,46%	2,83%	3,78%	1,72%
641	9	0,11%	0,34%	0,25%	2,09%	0,72%	0,24%	0,46%	6,62%	57,60%	0,22%	3,13%	4,75%	3,09%	1,88%	6,86%	2,80%	5,56%	1,11%
336	9	2,22%	2,77%	2,68%	4,76%	1,82%	1,27%	2,47%	8,27%	18,16%	0,34%	8,95%	0,00%	3,16%	1,87%	0,00%	0,00%	4,69%	5,33%
240	9	0,52%	1,13%	0,57%	5,20%	1,27%	0,41%	1,00%	7,98%	53,28%	0,25%	4,26%	4,90%	3,23%	4,30%	1,55%	1,35%	2,00%	0,82%
571	10	0,40%	0,58%	1,80%	4,83%	0,66%	1,47%	3,02%	20,84%	33,20%	1,11%	8,28%	2,84%	2,21%	2,09%	0,84%	0,82%	1,15%	0,72%
490	10	0,38%	0,65%	1,42%	4,43%	0,57%	0,88%	1,57%	13,32%	45,45%	0,78%	5,03%	3,84%	2,01%	1,97%	1,96%	1,47%	1,69%	0,61%
429	10	0,20%	0,38%	0,31%	2,62%	0,69%	0,21%	0,53%	6,33%	56,89%	0,32%	2,59%	5,30%	3,91%	3,19%	3,67%	2,29%	3,14%	1,43%
267	10	0,71%	0,70%	1,40%	4,31%	1,26%	1,45%	1,65%	10,19%	33,07%	1,11%	7,54%	3,95%	2,25%	1,88%	1,83%	1,00%	1,84%	0,88%
306	10	0,31%	1,48%	1,62%	5,35%	1,66%	0,89%	3,65%	23,77%	41,52%	0,96%	5,03%	2,49%	3,26%	2,82%	0,37%	0,52%	0,93%	0,77%
707	10	0,69%	1,05%	1,51%	6,92%	0,95%	1,08%	1,85%	12,78%	52,34%	0,51%	5,49%	5,37%	1,55%	2,55%	0,72%	0,87%	1,63%	0,62%
207	10	0,32%	0,38%	0,83%	3,65%	0,49%	0,97%	1,37%	9,42%	43,36%	0,60%	7,09%	5,39%	2,41%	2,07%	3,36%	1,93%	3,64%	0,33%
1318	11	0,33%	1,23%	1,29%	3,69%	0,70%	0,67%	1,42%	8,99%	46,15%	0,48%	2,94%	7,08%	3,73%	2,96%	2,03%	3,50%	7,19%	1,49%
691	11	0,36%	0,88%	0,81%	5,94%	1,04%	0,55%	1,11%	9,74%	43,10%	0,82%	4,90%	4,71%	3,00%	2,36%	1,93%	4,02%	3,77%	1,08%
494	11	1,10%	1,16%	1,82%	6,00%	0,97%	1,83%	3,14%	11,82%	30,47%	1,28%	10,08%	6,88%	1,68%	2,28%	1,70%	1,84%	2,42%	1,28%
502	11	0,34%	0,81%	0,98%	3,92%	1,16%	0,84%	1,67%	9,61%	25,39%	1,62%	18,97%	4,18%	1,76%	1,97%	3,91%	3,25%	3,19%	0,46%
651	11	0,58%	0,44%	1,44%	4,52%	0,37%	1,88%	2,48%	10,21%	32,80%	1,13%	10,83%	6,44%	1,93%	1,88%	0,94%	2,05%	3,86%	1,13%
552	11	0,72%	0,81%	1,56%	7,25%	0,91%	1,38%	1,65%	11,86%	40,30%	1,35%	8,02%	4,72%	2,36%	2,37%	1,88%	2,22%	2,98%	0,76%
666	11	0,51%	0,35%	1,51%	6,04%	0,26%	1,62%	2,03%	13,22%	32,88%	1,78%	11,78%	3,93%	2,04%	2,75%	2,51%	1,41%	1,86%	0,59%
713	12	0,55%	1,38%	1,28%	6,06%	1,27%	1,79%	2,67%	13,54%	34,86%	1,90%	13,53%	6,17%	1,93%	2,14%	2,69%	1,43%	1,36%	0,83%
692	12	0,24%	0,68%	0,82%	4,30%	0,79%	0,76%	1,15%	8,16%	49,81%	0,66%	4,74%	3,99%	3,17%	2,32%	3,22%	1,59%	2,99%	0,95%
210	12	0,28%	1,17%	0,70%	4,56%	1,09%	1,24%	0,97%	5,50%	45,12%	0,48%	7,95%	5,55%	2,22%	3,00%	4,28%	2,79%	5,86%	0,76%
650	12	0,33%	0,98%	1,62%	4,35%	0,73%	1,45%	2,67%	15,75%	42,69%	0,98%	7,65%	6,60%	2,21%	2,22%	1,89%	1,45%	2,48%	0,89%
460	12	0,24%	0,50%	1,48%	4,86%	0,45%	1,12%	2,45%	12,85%	42,56%	1,01%	5,42%	4,12%	2,58%	2,49%	2,52%	1,70%	2,08%	0,56%
717	12	0,85%	1,16%	2,85%	6,09%	0,39%	1,18%	2,30%	14,13%	39,10%	0,65%	4,18%	3,61%	2,96%	2,49%	3,29%	1,41%	2,13%	1,06%
1174	12	0,59%	1,71%	0,62%	5,89%	1,14%	0,62%	0,61%	4,91%	51,35%	0,25%	3,86%	4,59%	2,81%	3,51%	4,80%	1,37%	2,70%	0,74%
395	12	0,51%	0,37%	0,77%	4,18%	0,90%	0,69%	0,76%	8,98%	39,82%	0,42%	5,66%	4,77%	1,67%	1,54%	2,50%	1,95%	4,36%	0,35%
455	12	0,24%	0,78%	0,79%	3,88%	0,59%	1,35%	1,49%	9,13%	40,77%	0,89%	9,73%	6,22%	2,93%	2,26%	2,77%	1,84%	3,20%	0,90%
472	12	0,45%	0,77%	0,78%	4,25%	0,65%	0,99%	1,07%	8,37%	46,41%	0,70%	5,23%	4,34%	3,31%	3,00%	6,35%	2,21%	4,41%	0,77%
303	12	0,43%	0,57%	0,57%	3,04%	0,44%	0,85%	1,24%	7,11%	44,65%	0,73%	5,03%	8,39%	3,39%	2,17%	6,11%	2,32%	4,40%	1,43%
197	12	0,24%	0,84%	0,54%	4,88%	0,87%	0,55%	0,54%	9,07%	53,67%	0,31%	5,09%	3,44%	1,96%	2,59%	5,38%	0,62%	1,70%	0,71%
723	12	0,42%	0,83%	0,73%	4,25%	1,02%	0,53%	0,85%	7,97%	51,57%	0,59%	4,81%	3,76%	2,41%	1,60%	4,20%	1,84%	3,83%	0,94%

No.	Group No.	PG 30:0	PG 32:1	PG 32:0	PG 34:2	PG 34:1	PG 34:0	PG 35:1	PG 36:4	PG 36:3	PG 36:2	PG 36:1	PG 38:6	PG 38:5	PG 38:4
380	1	0,65%	2,90%	4,01%	2,57%	28,55%	2,81%	1,08%	2,39%	0,80%	16,32%	19,72%	0,89%	1,45%	1,12%
649	1	0,62%	3,04%	5,89%	4,82%	32,58%	1,25%	1,42%	2,18%	2,05%	17,42%	15,75%	0,89%	2,07%	1,85%
566	1	0,42%	2,23%	5,69%	5,83%	23,75%	1,24%	0,43%	1,39%	6,74%	33,71%	11,96%	0,42%	0,99%	0,70%
1349	1	0,43%	1,51%	4,72%	3,15%	30,95%	2,93%	1,49%	2,50%	2,57%	14,87%	22,85%	1,36%	1,21%	1,91%
386	1	1,47%	1,40%	4,31%	5,26%	29,02%	1,24%	0,64%	1,74%	2,61%	22,45%	13,50%	0,21%	1,63%	0,78%
206	1	1,18%	1,91%	4,19%	3,72%	28,45%	2,17%	1,63%	1,57%	1,52%	18,94%	20,14%	0,81%	1,47%	1,76%
399	1	0,94%	1,95%	3,86%	2,42%	37,18%	1,42%	1,56%	1,77%	1,11%	10,99%	21,20%	1,25%	1,20%	1,26%
725	1	1,65%	2,13%	6,37%	4,60%	27,03%	1,41%	0,54%	1,69%	3,29%	19,75%	11,88%	0,48%	1,57%	1,70%
560	1	0,66%	1,51%	5,07%	3,44%	32,48%	2,96%	1,92%	2,25%	1,86%	11,99%	20,08%	0,60%	2,00%	2,13%
436	1	0,54%	1,35%	4,85%	6,17%	22,88%	2,08%	1,20%	1,97%	6,28%	19,84%	19,08%	0,94%	1,64%	1,97%
435	1	1,12%	3,84%	5,74%	5,07%	34,80%	1,77%	1,39%	3,14%	1,99%	12,71%	13,50%	1,15%	1,46%	2,52%
357	2	1,18%	3,05%	4,72%	3,05%	36,88%	1,74%	1,63%	1,91%	1,27%	11,40%	19,57%	1,03%	1,06%	1,15%
638	2	0,81%	2,22%	6,31%	8,64%	27,96%	1,17%	0,82%	2,53%	7,16%	22,42%	10,64%	1,39%	2,25%	1,29%
727	2	1,21%	1,88%	4,61%	3,84%	28,88%	2,72%	1,30%	1,86%	2,75%	20,66%	13,26%	0,61%	1,35%	1,36%
252	2	0,63%	1,90%	7,69%	3,55%	35,66%	1,95%	1,43%	2,56%	1,45%	13,02%	18,43%	0,45%	1,29%	2,16%
549	2	5,27%	1,45%	16,37%	2,81%	16,09%	3,29%	0,00%	2,05%	2,27%	7,83%	9,44%	0,00%	6,62%	0,00%
553	2	1,30%	3,03%	5,84%	3,40%	34,04%	2,10%	1,76%	1,67%	1,27%	12,04%	21,15%	0,56%	1,00%	1,91%
1227	2	1,16%	2,57%	4,50%	3,73%	36,39%	1,72%	2,02%	1,99%	1,94%	13,48%	20,16%	0,85%	1,36%	1,69%
563	2	0,62%	2,41%	4,05%	4,33%	30,92%	1,46%	0,84%	1,70%	2,37%	20,42%	21,50%	0,61%	1,26%	1,71%
378	2	0,83%	1,67%	5,02%	2,79%	39,73%	2,78%	2,78%	1,42%	0,98%	9,95%	18,48%	0,38%	0,85%	1,15%
344	2	0,65%	2,44%	6,58%	5,22%	27,99%	1,81%	0,65%	1,87%	3,71%	22,18%	16,39%	0,16%	0,76%	2,24%
202	3	1,50%	1,95%	8,07%	5,83%	27,02%	0,00%	0,00%	0,00%	3,70%	13,77%	6,36%	2,69%	1,14%	0,00%
1178	3	3,93%	2,05%	9,94%	2,13%	12,10%	1,80%	0,00%	1,10%	2,64%	4,24%	4,42%	0,00%	0,00%	0,00%
612	4	1,99%	0,00%	2,40%	2,88%	6,34%	0,78%	0,00%	0,00%	2,36%	8,45%	8,27%	0,00%	0,00%	0,00%
550	4	0,55%	3,71%	7,64%	8,39%	30,05%	0,99%	0,26%	2,87%	5,53%	19,17%	9,71%	0,46%	2,22%	1,74%
639	4	1,12%	3,28%	7,38%	7,41%	35,47%	2,36%	0,77%	3,01%	3,95%	14,29%	7,30%	1,11%	2,73%	0,86%
818	4	3,43%	0,00%	2,70%	0,00%	2,74%	0,00%	0,00%	0,00%	0,00%	2,30%	1,81%	0,00%	0,00%	0,00%
495	4	0,61%	1,59%	6,23%	4,05%	34,76%	0,78%	0,47%	1,67%	4,18%	26,87%	12,22%	0,28%	1,47%	1,12%
1167	4	1,20%	1,96%	6,25%	6,10%	28,73%	1,93%	0,42%	2,32%	4,15%	18,08%	12,58%	1,23%	1,46%	1,50%
885	4	0,36%	1,61%	4,90%	6,65%	27,86%	1,47%	0,64%	2,71%	7,26%	25,39%	14,13%	0,85%	1,47%	1,57%
256	4	0,73%	2,27%	5,55%	5,92%	25,24%	1,42%	0,63%	2,25%	7,12%	25,27%	11,39%	0,95%	1,98%	1,25%
260	4	3,45%	2,43%	9,71%	2,68%	21,57%	1,95%	1,16%	1,74%	2,80%	5,21%	7,75%	0,00%	1,62%	0,00%
600	4	3,50%	4,91%	5,54%	5,86%	20,83%	1,74%	0,00%	1,79%	2,94%	11,09%	8,13%	1,04%	0,91%	2,37%
1299	4	1,39%	6,33%	7,08%	6,71%	28,61%	1,50%	0,72%	1,77%	2,50%	18,27%	9,17%	0,98%	0,93%	1,02%
653	4	0,57%	2,77%	4,94%	5,92%	28,17%	1,55%	0,42%	1,78%	5,53%	29,17%	11,50%	0,65%	1,99%	1,32%
313	4	0,93%	2,77%	3,81%	6,17%	20,28%	2,04%	0,00%	1,83%	4,14%	27,08%	9,55%	0,81%	1,87%	0,66%
396	4	0,82%	2,92%	7,33%	5,64%	30,01%	2,25%	0,86%	2,33%	3,67%	21,03%	13,03%	1,38%	2,34%	1,75%
1340	4	2,89%	2,09%	13,88%	3,69%	26,19%	3,49%	0,86%	0,44%	2,12%	10,55%	14,60%	1,14%	0,86%	0,89%
688	4	0,57%	1,59%	4,88%	4,90%	28,69%	1,94%	0,45%	1,96%	4,01%	25,04%	14,93%	1,01%	1,51%	0,82%
907	4	1,11%	1,95%	6,17%	6,89%	26,40%	0,93%	0,68%	1,94%	5,00%	22,03%	12,62%	0,72%	1,71%	0,80%
350	4	1,30%	2,59%	5,99%	3,26%	28,63%	2,56%	0,43%	1,32%	2,95%	18,48%	12,04%	0,00%	0,68%	0,52%
1313	5	0,9%	2,0%	4,4%	3,4%	33,9%	2,8%	1,2%	1,4%	1,6%	13,2%	24,2%	0,3%	0,9%	1,9%
1180	5	1,8%	2,0%	6,7%	3,7%	26,9%	1,5%	0,9%	2,9%	2,5%	14,3%	17,2%	1,0%	2,3%	2,8%
261	5	1,3%	1,3%	4,9%	3,2%	27,3%	1,2%	1,0%	1,1%	2,7%	19,1%	13,8%	0,0%	1,9%	1,0%
644	5	0,8%	2,0%	5,5%	3,9%	26,4%	2,5%	1,3%	2,0%	3,5%	21,8%	18,6%	0,5%	1,7%	1,9%
264	5	1,5%	3,5%	7,5%	5,0%	27,5%	0,5%	1,1%	1,8%	2,5%	16,1%	13,2%	0,4%	1,5%	1,4%
347	6	1,16%	2,25%	9,86%	7,60%	31,34%	1,39%	0,45%	3,71%	3,89%	15,28%	13,23%	0,70%	2,44%	2,45%
316	6	1,07%	2,83%	7,99%	5,51%	25,98%	1,77%	0,35%	3,60%	4,58%	19,57%	14,21%	0,54%	3,02%	3,99%
1323	6	1,50%	2,56%	7,43%	5,75%	22,94%	2,72%	0,00%	3,48%	4,57%	14,99%	8,14%	0,95%	3,38%	3,16%
235	6	1,07%	2,73%	7,12%	6,19%	23,52%	1,58%	0,69%	4,47%	5,10%	18,15%	11,73%	1,11%	2,64%	3,53%
721	6	0,80%	2,36%	5,74%	6,83%	26,89%	1,44%	0,69%	1,68%	6,28%	23,52%	10,84%	0,71%	2,21%	1,50%
345	6	1,36%	2,04%	6,22%	3,14%	20,19%	1,48%	0,37%	0,42%	3,33%	14,74%	13,37%	0,00%	0,00%	1,93%
603	6	1,85%	2,47%	15,89%	4,64%	23,87%	4,14%	0,41%	1,84%	2,87%	7,93%	13,80%	0,20%	1,22%	2,26%
269	6	0,45%	2,15%	4,07%	4,08%	29,62%	1,35%	0,90%	1,60%	2,43%	31,28%	14,22%	0,46%	1,96%	1,28%
273	7		0,00%	0,00%	0,00%	33,89%	0,00%		0,00%	0,00%	37,18%	16,90%		0,00%	0,00%
706	7	0,50%	2,07%	5,22%	4,72%	37,61%	1,57%	1,09%	2,27%	2,15%	16,84%	18,69%	0,49%	1,54%	1,59%
674	7	0,73%	2,12%	6,58%	3,76%	32,83%	2,61%	0,63%	2,10%	2,81%	17,45%	18,22%	0,77%	2,00%	2,35%
697	7	0,87%	1,74%	4,84%	3,96%	31,78%	1,48%	1,23%	1,24%	1,77%	15,37%	22,85%	0,44%	0,67%	1,18%
275	7	0,88%	2,56%	5,16%	5,81%	26,22%	1,73%	0,99%	2,19%	4,82%	24,86%	13,48%	1,58%	2,55%	1,64%
304	7	1,00%	2,67%	8,57%	3,85%	39,76%	2,72%	1,33%	1,97%	0,95%	9,13%	18,78%	0,91%	1,02%	1,71%
544	7	0,82%	2,04%	7,22%	4,73%	36,94%	1,35%	0,34%	2,42%	2,49%	17,96%	16,56%	0,37%	1,34%	1,65%
687	7	0,71%	1,68%	3,81%	3,38%	29,90%	1,20%	0,93%	1,97%	3,79%	25,65%	17,05%	0,28%	1,33%	0,89%
1208	7	1,31%	1,89%	10,07%	2,92%	38,06%	3,70%	0,58%	1,11%	1,17%	9,10%	21,74%	0,56%	0,70%	1,77%
266	8	1,04%	3,73%	7,11%	5,40%	35,29%	1,60%	1,38%	2,97%	2,04%	11,59%	13,27%	0,97%	2,24%	1,85%
487	8	1,24%	3,24%	7,55%	5,14%	31,89%	2,14%	0,52%	2,24%	1,29%	11,60%	12,90%	1,01%	1,47%	1,73%
548	8	0,59%	1,82%	5,12%	6,33%	25,58%	1,85%	1,23%	1,99%	5,66%	21,35%	15,61%	0,95%	1,36%	1,13%
346	8	0,72%	2,64%	5,17%	5,13%	25,01%	0,65%	0,00%	1,78%	4,63%	24,60%	9,96%	0,46%	2,01%	2,11%
1300	9	0,91%	1,12%	4,56%	5,18%	22,61%	1,14%	1,68%	2,09%	5,28%	20,13%	11,63%	0,58%	0,98%	0,44%
199	9		7,14%	8,44%	5,64%	33,93%	1,03%		4,09%	3,68%	15,83%	8,02%		4,45%	2,45%
719	9	0,60%	3,15%	5,99%	5,31%	35,54%	1,13%	1,11%	2,09%	3,50%	17,99%	15,80%	0,68%	1,23%	1,23%
393	9	1,88%	0,00%	3,71%	2,77%	9,59%	0,00%	0,00%	2,58%	0,00%	2,83%	3,03%	2,52%	0,00%	0,00%
541	9	2,34%	2,41%	3,96%	4,57%	18,49%	3,20%	0,00%	3,08%	0,00%	6,98%	7,38%	0,00%	0,00%	0,00%
258	9		0,00%	0,00%	0,00%	100,00%	0,00%		0,00%	0,00%	0,00%	0,00%		0,00%	0,00%
715	9	4,75%	0,88%	1,97%	0,00%	7,41%	0,00%	0,00%	0,00%	0,00%	4,76%	4,11%	0,00%	0,00%	0,00%
253	9	0,00%	0,00%	31,99%	14,74%	27,77%	0,00%	0,00%	0,00%	25,50%	0,00%	0,00%	0,00%	0,00%	0,00%
636	9	1,62%	1,37%	4,16%	2,66%	20,84%	2,95%	0,00%	1,55%	3,90%	21,96%	12,27%	0,00%	1,41%	1,20%
675	9	3,32%	0,00%	0,00%	0,00%	3,44%	0,00%	0,00%	0,00%	0,00%	0,00%	2,08%	0,00%	0,00%	0,00%
1326	9	0,65%	3,05%	5,69%	9,86%	16,84%	1,89%	0,00%	3,72%	8,72%	16,00%	6,50%	1,27%	2,30%	1,57%
68	9	0,99%	1,15%	3,24%	2,73%	29,80%	1,48%	0,49%	1,49%	1,55%	16,72%	16,32%	0,51%	0,94%	1,43%
227	9				3,51%	7,06%	8,83%	4,92%	0,78%					4,20%	2,29%
594	9	4,43%	0,00%	0,00%	0,00%	3,19%	0,00%	0,00%	0,00%	0,00%	0,00%	1,63%	0,00%	0,00%	0,00%
659	9	1,88%	2,01%	6,96%	4,46%	23,51%	1,07%	1,84%	1,63%	1,37%	11,38%	6,31%	0,45%	1,05%	0,54%
507	9	0,00%	1,52%	0,00%	1,18%	8,57%	0,00%	0,00%	0,00%	0,00%	3,46%	2,50%	0,00%	0,00%	0,00%
451	9	3,10%	0,00%	0,00%	0,00%	1,78%	0,00%	0,00%	0,00%	0,00%	1,91%	2,03%	0,00%	0,00%	0,00%
454	9	1,90%	2,69%	4,20%	3,81%	21,28%	1,00%	0,00%	2,21%	0,00%	8,64%	0,00%	0,00%	1,17%	0,86%
641	9	1,53%	1,21%	7,63%	9,67%	12,63%	3,41%	0,00%	2,24%	6,43%	9,89%	6,39%	0,00%	3,86%	1,44%
336	9		0,00%	4,10%	8,50%	15,04%	0,00%		6,03%	11,05%	15,20%	9,05%		6,70%	0,00%
240	9	4,44%	2,21%	11,68%	1,76%	22,12%	6,16%	0,00%	0,00%	0,00%	2,87%	11,35%	0,00%	0,00%	0,51%
571	10	0,64%	1,72%	5,73%	5,13%	30,92%	1,07%	0,48%	3,01%	5,15%	22,20%	14,76%	0,82%	2,20%	1,40%
490	10	0,88%	2,65%	5,53%	6,55%	30,04%	1,44%	0,78%	2,56%	3,73%	18,44%	13,71%	0,73%	0,98%	1,82%
429	10	4,03%	0,00%	1,94%	2,17%	11,93%	1,16%	0,00%	0,00%	1,09%	4,44%	5,82%	0,00%	0,00%	0,00%
267	10	0,95%	3,12%	6,60%	3,89%	27,57%	1,78%	2,51%	1,20%	1,60%	14,96%	16,00%	0,37%	0,58%	1,12%
306	10	0,13%	0,96%	2,50%	4,41%	17,68%	0,72%	0,50%	2,21%	10,20%	40,43%	13,83%	0,38%	1,97%	1,25%
707	10	1,47%	3,79%	9,91%	6,21%	32,65%	2,34%	0,49%	1,82%	3,71%	19,54%	11,68%	0,45%	0,85%	1,31%
207	10	1,04%	2,33%	4,62%	3,31%	36,95%	1,35%	0,65%	1,51%	2,01%	11,59%	17,22%	0,18%	1,40%	1,74%
1318	11	1,91%	3,46%	16,95%	12,36%	18,60%	3,35%	0,53%	2,59%	5,91%	12,34%	7,37%	0,65%	0,92%	1,04%
691	11	2,34%	2,31%	7,56%	3,55%	24,65%	3,05%	1,48%	2,58%	2,63%	13,73%	11,91%	0,00%	1,88%	2,33%
494	11	4,24%	5,60%	10,15%	7,88%	27,38%	1,81%	0,80%	2,17%	3,74%	11,00%	9,95%	0,46%	1,10%	1,06%
502	11	5,07%	1,82%	4,12%	8,50%	22,71%	0,70%	0,00%	0,00%	2,77%	12,96%	8,58%	0,00%	0,41%	2,07%
651	11	3,03%	3,05%	6,15%	6,03%	23,82%	1,32%	0,95%	4,34%	2,24%	10,46%	12,39%	0,48%	1,85%	1,52%
552	11	0,88%	4,64%	9,49%	6,28%	30,43%	2,37%	0,76%	2,03%	2,39%	12,71%	12,94%	0,99%	1,96%	2,24%
666	11	0,58%	2,29%	4,32%	4,07%	34,42%	0,98%	1,14%	1,56%	2,06%	17,98%	20,10%	1,12%	0,96%	1,60%
713	12	0,47%	2,04%	6,45%	4,83%	25,79%	1,68%	0,50%	2,00%	6,42%	26,72%	13,06%	1,17%	2,44%	1,68%
692	12	1,02%	1,94%	5,17%	4,11%	27,56%	0,80%	0,30%	1,96%	4,64%	28,47%	10,16%	0,61%	1,55%	1,09%
210	12	3,33%	0,00%	1,36%	1,99%	11,53%	0,00%	0,00%	0,00%	0,00%	1,10%	3,70%	0,00%	0,00%	1,68%
650	12	0,90%	2,03%	7,53%	5,96%	30,20%	2,10%	0,58%	2,99%	5,04%	20,56%	11,92%	0,61%	2,07%	2,04%
460	12	0,94%	1,82%	6,73%	4,89%	31,43%	1,43%	0,67%	1,93%	3,59%	22,63%	13,31%	0,63%	1,74%	1,34%
717	12	0,79%	3,44%	7,27%	4,81%	29,17%	2,45%	0,34%	2,91%	1,54%	16,08%	12,18%	0,92%	1,49%	1,17%
1174	12	1,99%	6,96%	14,45%	3,03%	24,36%	3,56%	0,00%	0,55%	0,85%	6,78%	7,43%	0,00%	0,00%	0,42%
395	12	2,52%	0,00%	1,89%	0,00%	9,36%	0,00%	0,00%	0,00%	0,00%	4,75%	3,17%	0,00%	0,00%	1,11%
455	12	3,68%	0,00%	1,61%	0,00%	14,48%	0,00%	0,00%	0,00%	0,00%	8,50%	8,48%	0,00%	0,00%	0,00%
472	12	0,70%	3,18%	8,25%	5,61%	29,27%	1,96%	0,79%	2,37%	3,60%	21,53%	10,69%	1,10%	1,36%	0,96%
303	12	0,99%	4,52%	7,74%	7,24%	30,24%	1,10%	0,54%	2,51%	3,54%	17,38%	9,32%	0,90%	1,56%	1,64%
197	12	1,06%	1,73%	10,89%	9,68%	24,69%	3,74%	0,00%	1,07%	4,88%	18,02%	8,26%	0,00%	0,00%	0,64%
723	12	4,64%	3,09%	9,28%	9,77%	20,36%	1,44%	0,85%	1,52%	4,31%	7,40%	5,97%	0,00%	0,00%	0,42%

No.	Group No.	LPC 15:0	LPC 16:1	LPC 16:0	LPC 18:2	LPC 18:1	LPC 18:0	LPC 20:4	LPC 20:3	LPC 20:0	LPC 22:6	LPC 22:4
380	1	1,64%	4,14%	49,63%	6,77%	13,26%	9,46%	3,98%	0,52%	1,24%	0,66%	1,31%
649	1	2,13%	4,65%	60,23%	3,73%	7,75%	9,77%	1,32%	0,00%	1,02%	0,45%	1,07%
566	1	0,54%	5,23%	67,63%	1,47%	14,66%	7,00%	0,16%	0,10%	0,88%	0,14%	0,10%
1349	1	1,98%	3,40%	49,02%	5,06%	7,85%	16,43%	1,45%	0,00%	1,57%	0,95%	0,62%
386	1	2,91%	3,04%	38,22%	5,67%	7,98%	16,16%	3,79%	0,00%	3,23%	0,00%	4,78%
206	1	2,04%	3,91%	45,70%	4,17%	8,37%	13,74%	3,52%	0,91%	1,56%	1,14%	3,92%
399	1	3,54%	3,79%	45,77%	2,02%	5,61%	9,95%	1,39%	0,64%	2,63%	0,80%	1,11%
725	1	3,32%	4,40%	40,50%	4,84%	7,27%	9,53%	1,48%	1,01%	2,68%	0,65%	3,54%
560	1	2,08%	2,72%	44,11%	3,42%	5,94%	16,33%	3,80%	0,85%	3,31%	0,90%	3,98%
436	1	1,73%	4,05%	49,01%	9,53%	10,81%	13,85%	1,68%	0,53%	1,02%	0,56%	1,29%
435	1	1,55%	2,69%	59,42%	6,98%	6,85%	10,84%	1,81%	0,50%	1,05%	0,41%	1,77%
357	2	2,88%	3,95%	51,84%	2,01%	6,36%	11,06%	1,85%	0,63%	2,35%	1,02%	2,17%
638	2	0,49%	1,79%	78,74%	1,09%	4,39%	12,26%	0,27%	0,11%	0,17%	0,13%	0,08%
727	2	2,93%	3,02%	47,78%	5,01%	7,97%	12,71%	1,94%	1,01%	1,91%	0,70%	2,64%
252	2	1,43%	3,19%	63,87%	3,58%	6,46%	11,02%	0,95%	0,45%	0,97%	0,70%	1,09%
549	2	2,03%	2,23%	62,66%	2,40%	5,94%	9,97%	2,85%	0,57%	1,00%	0,69%	1,20%
553	2	1,96%	3,52%	57,20%	1,96%	8,76%	15,86%	1,15%	0,39%	1,25%	0,41%	1,35%
1227	2	1,73%	3,47%	59,98%	3,59%	12,23%	11,82%	1,50%	0,46%	0,76%	0,38%	0,73%
563	2	1,49%	3,52%	63,74%	3,84%	9,09%	11,73%	1,30%	0,36%	0,65%	0,36%	0,80%
378	2	2,95%	4,72%	50,53%	4,34%	6,28%	9,65%	2,46%	0,56%	1,67%	0,85%	1,11%
344	2	4,86%	2,13%	58,31%	0,99%	12,46%	14,78%	2,41%	0,36%	0,49%	0,13%	1,08%
202	3	0,70%	3,38%	76,75%	1,10%	8,28%	7,91%	0,29%	0,19%	0,13%	0,16%	0,30%
1178	3	2,75%	1,99%	46,34%	1,31%	3,61%	15,36%	2,09%	0,00%	3,17%	1,27%	3,15%
612	4	3,03%	3,73%	24,90%	17,31%	12,81%	6,38%	3,26%	1,49%	3,10%	1,54%	1,89%
550	4	1,31%	5,40%	57,48%	2,91%	9,26%	12,56%	1,09%	0,51%	1,20%	0,47%	1,01%
639	4	0,45%	3,33%	80,97%	0,88%	6,02%	7,16%	0,29%	0,10%	0,10%	0,15%	0,08%
818	4	6,32%	0,00%	12,86%	0,00%	4,50%	7,82%	0,00%	0,00%	9,46%	0,00%	4,55%
495	4	1,10%	3,55%	58,15%	5,02%	11,03%	10,92%	1,68%	0,38%	1,34%	0,47%	1,63%
1167	4	1,69%	3,15%	47,75%	4,54%	8,38%	18,48%	1,45%	0,61%	2,25%	0,72%	1,06%
885	4	0,59%	3,21%	55,16%	5,34%	17,99%	12,07%	0,77%	0,44%	1,32%	0,25%	0,14%
256	4	0,79%	4,50%	61,61%	2,77%	10,73%	12,30%	1,20%	0,43%	0,86%	0,44%	0,84%
260	4	3,77%	2,17%	26,87%	2,36%	4,68%	21,18%	3,45%	0,00%	3,97%	1,46%	6,15%
600	4	2,47%	4,78%	48,56%	2,71%	7,50%	13,53%	2,10%	0,65%	1,95%	0,70%	2,64%
1299	4	1,84%	5,22%	56,27%	1,92%	12,90%	12,17%	1,49%	0,42%	1,07%	0,44%	1,69%
653	4	0,62%	5,57%	61,55%	3,40%	14,99%	9,16%	0,79%	0,36%	0,54%	0,51%	0,37%
313	4	1,23%	6,11%	61,97%	2,99%	7,83%	8,61%	1,12%	0,51%	0,90%	0,55%	0,90%
396	4	0,54%	4,36%	57,48%	3,58%	17,00%	13,76%	1,11%	0,58%	0,16%	0,68%	0,14%
1340	4	0,82%	6,52%	54,33%	4,57%	14,22%	12,11%	1,29%	0,48%	0,54%	0,53%	1,28%
688	4	0,33%	3,38%	53,52%	5,93%	20,10%	13,32%	0,86%	0,25%	0,26%	0,82%	0,12%
907	4	2,05%	3,18%	51,62%	2,69%	8,03%	16,90%	1,35%	0,54%	2,23%	0,73%	1,25%
350	4	3,68%	3,60%	38,55%	2,85%	5,85%	10,95%	1,16%	1,00%	3,98%	1,35%	1,89%
1313	5	1,4%	3,5%	52,4%	4,8%	9,8%	14,9%	1,5%	0,8%	1,0%	0,7%	1,6%
1180	5	2,6%	3,1%	55,6%	2,5%	6,0%	13,2%	2,8%	0,6%	1,3%	0,5%	2,7%
261	5	1,7%	3,7%	50,0%	3,7%	8,2%	11,0%	1,7%	0,6%	1,4%	0,6%	2,5%
644	5	1,2%	2,8%	57,1%	4,0%	8,9%	16,5%	1,6%	0,4%	0,8%	0,4%	1,3%
264	5	2,4%	3,1%	54,5%	3,1%	6,1%	13,3%	2,9%	0,5%	1,1%	0,5%	3,9%
347	6	2,10%	2,50%	65,07%	1,69%	4,22%	15,27%	1,15%	0,34%	1,22%	0,44%	2,04%
316	6	2,30%	3,91%	50,02%	2,35%	5,55%	16,51%	2,24%	0,66%	1,48%	0,75%	3,35%
1323	6	5,53%	1,65%	38,34%	1,93%	5,86%	18,27%	3,52%	0,98%	2,17%	0,88%	6,91%
235	6	1,23%	5,81%	63,02%	3,88%	11,67%	7,87%	1,18%	0,54%	0,36%	0,42%	0,67%
721	6	1,42%	3,98%	60,64%	3,55%	10,46%	10,00%	1,09%	0,34%	1,17%	0,57%	1,01%
345	6	2,13%	3,59%	57,55%	1,53%	11,06%	12,46%	1,64%	0,63%	1,38%	0,43%	2,12%
603	6	2,30%	1,64%	41,73%	1,07%	4,88%	38,08%	0,99%	0,45%	1,13%	0,39%	2,36%
269	6	0,84%	6,27%	57,40%	2,03%	17,26%	7,78%	1,55%	0,57%	0,69%	0,59%	0,62%
273	7	0,99%	2,60%	60,78%	4,20%	12,64%	12,20%	2,28%	0,00%	0,00%	0,00%	0,92%
706	7	1,01%	5,21%	60,25%	4,45%	10,07%	12,13%	1,22%	0,29%	1,00%	0,43%	0,59%
674	7	2,17%	4,05%	46,03%	5,33%	7,20%	17,30%	2,01%	0,00%	2,85%	1,00%	1,94%
697	7	1,80%	3,22%	54,15%	2,81%	8,00%	15,64%	1,06%	0,62%	1,97%	0,53%	1,05%
275	7	1,23%	4,46%	58,19%	4,62%	9,33%	14,04%	1,73%	0,66%	0,82%	0,66%	0,95%
304	7	0,80%	5,86%	59,37%	5,94%	9,43%	11,81%	1,12%	0,33%	0,75%	0,72%	0,43%
544	7	1,76%	4,47%	48,24%	6,43%	9,95%	12,94%	2,41%	0,50%	1,64%	0,43%	3,22%
687	7	2,75%	4,59%	45,94%	4,84%	11,07%	11,78%	0,99%	0,79%	2,52%	0,71%	0,84%
1208	7	0,55%	7,83%	54,87%	6,62%	15,97%	7,71%	1,28%	0,33%	0,60%	0,64%	0,86%
266	8	1,70%	4,68%	51,08%	3,91%	6,76%	14,25%	3,11%	0,66%	1,42%	0,73%	2,53%
487	8	4,04%	3,49%	37,80%	4,63%	6,90%	12,84%	4,63%	0,66%	3,16%	1,04%	3,59%
548	8	1,49%	3,92%	62,78%	3,22%	6,25%	12,37%	1,01%	0,35%	1,12%	0,42%	1,26%
346	8	2,38%	6,68%	37,34%	8,59%	11,11%	10,32%	2,83%	0,99%	1,59%	1,11%	2,04%
1300	9	2,41%	2,47%	45,38%	6,55%	9,50%	16,50%	1,43%	0,00%	2,07%	0,86%	1,70%
199	9	0,85%	7,90%	70,38%	1,40%	9,32%	7,16%	0,89%	0,83%	0,09%	0,18%	0,11%
719	9	1,18%	4,80%	63,64%	4,11%	8,92%	11,59%	0,73%	0,32%	0,63%	0,31%	0,42%
393	9	1,26%	2,79%	69,38%	1,04%	5,03%	14,55%	0,65%	0,40%	0,54%	0,00%	1,09%
541	9	2,15%	2,56%	42,75%	5,15%	5,74%	12,63%	2,71%	1,07%	2,25%	1,02%	4,84%
258	9	2,47%	0,54%	42,37%	2,02%	12,45%	37,03%	0,75%	0,56%	0,59%	0,51%	0,38%
715	9	1,72%	1,11%	51,72%	1,22%	5,52%	25,77%	0,97%	0,43%	1,48%	0,47%	1,35%
253	9	0,74%	1,07%	55,10%	14,31%	7,28%	17,39%	1,70%	0,87%	0,13%	0,23%	0,07%
636	9	4,20%	3,79%	29,75%	3,63%	6,20%	11,90%	1,71%	0,00%	5,10%	0,00%	0,00%
675	9	1,70%	1,05%	53,66%	0,83%	6,17%	26,67%	0,61%	0,00%	1,03%	0,41%	1,08%
1326	9	0,55%	5,01%	53,37%	5,55%	17,87%	10,71%	1,26%	0,54%	0,52%	0,60%	0,79%
68	9	4,22%	4,39%	35,82%	5,41%	8,92%	13,03%	2,60%	0,00%	2,61%	1,08%	1,43%
227	9	1,32%	1,69%	80,94%	1,04%	5,93%	7,91%	0,22%	0,10%	0,19%	0,08%	0,09%
594	9	2,65%	2,93%	62,04%	2,01%	5,56%	12,64%	1,22%	0,00%	1,33%	0,56%	1,35%
659	9	3,33%	3,95%	32,50%	4,78%	7,38%	14,72%	3,33%	1,50%	2,85%	1,17%	2,91%
507	9	11,49%	3,06%	21,67%	2,06%	3,25%	5,90%	2,75%	1,13%	7,22%	1,75%	3,00%
451	9	3,42%	1,15%	41,86%	1,62%	4,92%	25,69%	1,48%	0,00%	2,48%	1,12%	2,22%
454	9	0,63%	3,92%	71,95%	0,64%	5,87%	14,22%	0,22%	0,18%	0,48%	0,24%	0,32%
641	9	2,09%	1,69%	57,69%	1,40%	7,85%	24,57%	0,60%	0,24%	0,61%	0,19%	0,97%
336	9	2,77%	2,80%	55,04%	1,27%	5,02%	12,44%	0,83%	0,36%	2,07%	0,75%	1,11%
240	9	0,72%	3,62%	69,44%	0,92%	10,75%	12,35%	0,18%	0,14%	0,39%	0,09%	0,11%
571	10	1,90%	3,81%	50,29%	5,24%	8,75%	11,55%	1,21%	0,41%	2,65%	0,92%	1,09%
490	10	1,29%	3,90%	56,11%	3,68%	10,01%	12,86%	2,03%	0,40%	1,06%	0,57%	2,05%
429	10	1,80%	1,29%	41,86%	1,21%	11,58%	24,69%	1,60%	0,82%	1,91%	0,65%	3,01%
267	10	2,58%	15,26%	34,77%	9,88%	17,27%	5,07%	3,87%	0,79%	1,24%	1,09%	0,84%
306	10	0,32%	5,34%	53,77%	6,06%	19,74%	10,93%	0,70%	0,20%	0,59%	0,20%	0,12%
707	10	2,79%	3,56%	61,99%	2,17%	9,93%	11,16%	2,27%	0,50%	0,82%	0,25%	1,14%
207	10	1,42%	9,05%	50,16%	7,66%	17,09%	6,45%	2,09%	0,75%	0,61%	0,47%	0,41%
1318	11	0,59%	1,55%	79,47%	1,47%	5,26%	9,68%	0,20%	0,25%	0,27%	0,08%	0,27%
691	11	2,10%	1,97%	54,25%	2,47%	10,53%	13,66%	1,40%	0,73%	1,15%	0,41%	2,66%
494	11	1,92%	3,83%	62,59%	3,59%	6,80%	10,65%	1,52%	0,76%	0,76%	0,25%	2,13%
502	11	3,54%	2,65%	45,15%	3,80%	9,06%	15,73%	1,58%	0,73%	1,98%	1,11%	2,69%
651	11	2,00%	3,98%	59,96%	2,17%	7,34%	9,15%	2,08%	0,99%	0,78%	0,38%	3,23%
552	11	1,02%	6,41%	64,30%	1,51%	14,85%	8,47%	0,67%	0,32%	0,35%	0,24%	0,58%
666	11	0,86%	2,57%	51,01%	4,88%	9,59%	22,43%	1,39%	0,66%	0,79%	0,74%	0,76%
713	12	1,06%	4,48%	53,06%	5,15%	13,79%	12,75%	1,81%	0,60%	1,06%	0,73%	1,01%
692	12	1,28%	4,51%	51,32%	4,81%	14,85%	10,96%	2,30%	0,50%	1,10%	0,62%	1,76%
210	12	0,95%	1,65%	47,33%	2,03%	6,56%	24,23%	4,68%	0,78%	0,90%	0,78%	4,75%
650	12	0,58%	1,93%	70,42%	1,93%	6,68%	15,40%	0,55%	0,23%	0,41%	0,23%	0,31%
460	12	2,18%	4,04%	57,33%	3,07%	5,86%	10,35%	1,47%	0,81%	2,35%	0,82%	1,08%
717	12	1,58%	3,55%	50,92%	4,34%	11,10%	13,09%	1,90%	0,59%	1,23%	0,87%	1,25%
1174	12	0,74%	4,50%	67,70%	0,73%	9,65%	12,74%	0,32%	0,16%	0,55%	0,14%	0,13%
395	12	3,34%	1,78%	35,43%	2,61%	5,63%	14,58%	5,10%	1,19%	3,39%	1,02%	7,80%
455	12	3,31%	2,37%	41,13%	3,92%	6,31%	11,86%	2,07%	0,00%	2,76%	0,00%	3,25%
472	12	0,50%	5,09%	63,83%	1,92%	16,68%	8,89%	0,52%	0,28%	0,26%	0,56%	0,14%
303	12	0,70%	4,40%	67,99%	2,17%	9,34%	11,87%	0,62%	0,53%	0,30%	0,50%	0,28%
197	12	1,77%	2,51%	54,94%	2,58%	9,74%	20,32%	1,51%	0,39%	1,27%	0,43%	0,86%
723	12	1,11%	2,89%	56,92%	2,92%	7,32%	17,24%	2,32%	0,49%	1,29%	0,42%	2,69%

No.	Group No.	Cer 16:0	Cer 18:0	Cer 20:0	Cer 22:0	Cer 23:0	Cer 24:1	Cer 24:0	HexCer 16:0	HexCer 24:1
380	1	33,73%	14,56%	8,34%	9,53%	6,06%	13,57%	14,20%	6,59%	4,08%
649	1	38,75%	15,03%	6,16%	10,14%	7,14%	12,99%	9,79%	8,18%	5,91%
566	1	41,60%	5,86%	4,89%	8,33%	3,32%	25,24%	10,76%	9,12%	11,28%
1349	1	31,11%	20,65%	9,15%	10,68%	5,50%	10,55%	12,34%	8,22%	7,06%
386	1	35,27%	11,66%	7,06%	8,08%	6,86%	17,38%	13,68%	5,86%	5,35%
206	1	39,97%	14,06%	5,29%	6,66%	5,54%	18,44%	10,05%	6,41%	4,39%
399	1	31,57%	16,62%	9,32%	12,16%	5,99%	11,18%	13,16%	6,95%	6,22%
725	1	42,45%	20,61%	8,51%	6,47%	3,92%	10,25%	7,79%	11,07%	3,79%
560	1	41,32%	13,32%	4,39%	9,61%	5,44%	14,33%	11,59%	7,07%	4,44%
436	1	48,12%	16,01%	6,66%	8,35%	3,06%	9,89%	7,90%	5,45%	3,47%
435	1	45,84%	17,69%	5,85%	6,11%	3,52%	14,18%	6,80%	5,93%	3,29%
357	2	40,07%	14,07%	5,80%	10,01%	4,55%	15,33%	10,16%	7,37%	4,04%
638	2	51,71%	16,06%	4,42%	5,29%	1,50%	14,41%	6,61%	6,82%	4,67%
727	2	39,55%	14,30%	9,51%	9,67%	4,16%	11,71%	11,11%	6,18%	3,96%
252	2	52,14%	11,82%	6,15%	6,53%	3,04%	13,37%	6,96%	9,16%	4,47%
549	2	35,39%	8,38%	4,23%	11,04%	2,63%	24,14%	14,19%	8,59%	4,17%
553	2	56,78%	8,11%	4,01%	6,40%	1,68%	16,69%	6,33%	8,88%	3,00%
1227	2	49,33%	16,22%	6,17%	7,12%	2,39%	13,43%	5,34%	6,18%	1,83%
563	2	57,26%	8,49%	4,39%	6,37%	1,67%	15,63%	6,19%	8,48%	3,77%
378	2	31,97%	15,60%	5,90%	10,51%	9,90%	13,64%	12,48%	9,88%	6,17%
344	2	39,72%	2,10%	1,04%	5,82%	4,35%	31,37%	15,60%	8,25%	10,96%
202	3	48,22%	22,75%	7,62%	4,95%	1,38%	11,31%	3,77%	10,72%	3,02%
1178	3	46,18%	21,63%	9,35%	6,60%	2,03%	8,78%	5,44%	9,92%	2,32%
612	4	55,15%	17,03%	6,57%	6,56%	3,33%	6,58%	4,79%	5,74%	1,14%
550	4	32,47%	8,27%	3,32%	9,79%	3,06%	31,00%	12,07%	6,35%	8,27%
639	4	55,69%	22,30%	7,64%	4,93%	1,05%	5,93%	2,46%	8,62%	1,54%
818	4	24,37%	19,12%	15,09%	7,90%	11,66%	16,45%	5,41%	13,74%	0,00%
495	4	44,28%	12,87%	4,63%	8,18%	4,27%	19,00%	6,77%	11,30%	6,71%
1167	4	58,56%	17,02%	5,95%	4,08%	1,21%	10,19%	2,99%	9,30%	1,38%
885	4	38,62%	13,49%	7,45%	11,23%	4,58%	11,59%	13,04%	8,51%	6,77%
256	4	44,38%	18,41%	8,25%	6,77%	2,00%	13,29%	6,90%	9,84%	3,52%
260	4	40,88%	11,53%	4,98%	10,48%	2,85%	19,33%	9,95%	8,34%	4,43%
600	4	52,01%	15,49%	8,05%	7,41%	1,60%	9,63%	5,81%	11,53%	2,56%
1299	4	50,61%	17,14%	5,59%	4,67%	2,27%	12,46%	7,26%	8,25%	3,67%
653	4	42,80%	11,05%	5,38%	7,75%	2,34%	19,74%	10,94%	8,73%	6,98%
313	4	47,76%	15,95%	7,93%	7,31%	3,29%	11,36%	6,41%	7,99%	3,02%
396	4	49,33%	18,19%	6,47%	5,63%	1,14%	14,01%	5,23%	13,02%	4,63%
1340	4	48,55%	15,65%	4,19%	7,50%	3,21%	12,34%	8,57%	9,40%	5,34%
688	4	51,59%	23,44%	7,52%	4,47%	1,16%	9,17%	2,66%	6,53%	1,82%
907	4	54,66%	20,11%	6,36%	4,06%	1,06%	10,80%	2,95%	8,31%	2,09%
350	4	45,40%	16,59%	7,72%	8,09%	3,11%	13,10%	5,99%	7,34%	5,05%
1313	5	52,53%	21,41%	7,50%	5,64%	1,63%	7,41%	3,87%	8,46%	1,47%
1180	5	39,27%	12,05%	7,99%	12,06%	0,00%	16,56%	12,06%	14,48%	9,28%
261	5	41,19%	17,09%	4,03%	9,09%	0,00%	18,36%	10,24%	14,97%	5,37%
644	5	45,63%	13,00%	4,95%	9,04%	3,13%	14,21%	10,04%	14,69%	11,21%
264	5	37,82%	12,43%	6,11%	11,08%	5,02%	16,66%	10,89%	11,94%	9,93%
347	6	49,00%	7,73%	3,51%	6,50%	2,11%	22,35%	8,80%	7,86%	4,22%
316	6	41,34%	11,07%	4,24%	8,86%	3,43%	21,24%	9,82%	9,73%	9,50%
1323	6	38,31%	10,50%	5,25%	9,80%	2,52%	21,68%	11,93%	10,24%	8,61%
235	6	35,30%	12,64%	6,20%	12,20%	4,57%	16,59%	12,50%	8,81%	8,66%
721	6	44,18%	13,11%	4,53%	9,31%	2,95%	18,00%	7,92%	8,17%	6,62%
345	6	50,00%	13,48%	6,97%	6,99%	1,83%	16,14%	4,58%	6,27%	2,43%
603	6	41,63%	8,48%	3,91%	9,52%	2,18%	22,25%	12,03%	10,18%	3,90%
269	6	47,50%	8,47%	5,92%	8,01%	2,50%	19,16%	8,43%	7,26%	5,64%
273	7	44,17%	7,50%	4,49%	9,13%	1,93%	20,11%	12,67%	4,40%	2,70%
706	7	44,91%	12,25%	6,57%	10,06%	2,54%	13,00%	10,68%	13,40%	8,45%
674	7	41,87%	17,32%	5,77%	10,01%	1,56%	10,95%	12,51%	8,18%	4,68%
697	7	48,84%	19,69%	7,80%	6,85%	2,61%	7,17%	7,03%	7,52%	2,51%
275	7	45,30%	14,76%	5,63%	5,79%	2,17%	19,25%	7,11%	8,50%	3,78%
304	7	40,31%	20,02%	8,71%	9,20%	3,98%	10,85%	6,93%	6,85%	4,93%
544	7	41,76%	11,32%	4,70%	6,48%	2,96%	23,58%	9,20%	7,88%	3,92%
687	7	35,65%	15,19%	6,93%	8,57%	5,04%	10,15%	18,48%	6,19%	3,23%
1208	7	38,36%	17,60%	8,89%	7,93%	4,05%	12,80%	10,37%	8,12%	6,00%
266	8	35,12%	15,69%	6,74%	9,09%	6,25%	14,14%	12,97%	11,57%	8,76%
487	8	39,83%	10,56%	3,90%	7,56%	6,21%	17,88%	14,06%	7,15%	5,86%
548	8	34,53%	7,18%	3,65%	10,23%	5,88%	21,87%	16,66%	7,38%	7,34%
346	8	39,22%	20,98%	5,28%	5,57%	0,00%	17,47%	11,48%	8,40%	5,19%
1300	9	47,11%	29,22%	8,01%	2,92%	1,41%	9,49%	1,84%	6,73%	1,07%
199	9	36,20%	3,61%	3,19%	12,82%	3,12%	26,68%	14,38%	8,80%	11,99%
719	9	54,22%	18,16%	6,34%	4,82%	1,81%	9,11%	5,54%	5,67%	1,56%
393	9	30,25%	7,30%	4,98%	12,92%	4,22%	13,54%	26,79%	9,55%	7,25%
541	9	39,64%	12,09%	5,99%	8,45%	4,68%	15,23%	13,91%	6,55%	5,32%
258	9	56,38%	15,96%	4,25%	4,24%	1,05%	13,56%	4,57%	8,93%	3,91%
715	9	49,69%	18,73%	8,63%	6,09%	1,89%	8,70%	6,26%	11,98%	2,49%
253	9	10,66%	2,66%	2,04%	14,61%	13,87%	15,21%	40,95%	6,52%	5,54%
636	9	38,41%	17,82%	7,17%	8,03%	3,13%	14,98%	10,46%	9,94%	3,65%
675	9	49,06%	22,78%	8,37%	5,33%	1,22%	10,17%	3,06%	9,48%	2,24%
1326	9	49,50%	19,02%	4,81%	5,38%	2,92%	10,67%	7,71%	7,22%	4,46%
68	9	44,62%	9,80%	7,52%	8,73%	0,00%	8,82%	20,51%	0,00%	0,00%
227	9	35,53%	5,23%	3,02%	11,31%	6,13%	20,35%	18,42%	15,86%	27,19%
594	9	31,31%	10,97%	5,56%	11,58%	6,62%	14,98%	18,98%	8,34%	7,96%
659	9	57,96%	4,92%	2,85%	6,96%	1,83%	17,94%	7,53%	6,05%	3,36%
507	9	28,14%	18,37%	6,72%	8,08%	7,95%	18,73%	12,01%	7,76%	8,80%
451	9	52,28%	21,37%	8,15%	5,83%	1,27%	7,94%	3,16%	8,71%	1,25%
454	9	54,45%	20,73%	6,63%	4,71%	1,35%	9,12%	3,00%	12,22%	2,76%
641	9	53,31%	16,71%	5,40%	4,92%	1,53%	12,07%	6,05%	8,70%	2,22%
336	9	35,61%	7,63%	3,80%	9,54%	4,59%	18,19%	20,63%	7,40%	9,00%
240	9	45,68%	6,99%	4,64%	9,63%	2,21%	18,20%	12,65%	4,70%	2,96%
571	10	39,10%	21,07%	6,58%	10,69%	3,43%	11,06%	8,08%	9,23%	5,89%
490	10	52,28%	10,05%	4,01%	6,23%	2,11%	16,40%	8,92%	7,62%	3,31%
429	10	58,97%	8,49%	5,14%	5,83%	1,69%	13,41%	6,46%	6,22%	3,28%
267	10	37,52%	16,78%	4,70%	6,24%	4,13%	15,71%	14,92%	10,20%	4,19%
306	10	40,39%	12,73%	4,95%	9,97%	2,43%	17,31%	12,20%	9,70%	6,89%
707	10	37,69%	5,22%	2,25%	5,72%	5,15%	24,69%	19,29%	5,72%	5,11%
207	10	46,69%	19,52%	8,66%	7,22%	1,89%	11,11%	4,91%	6,80%	2,56%
1318	11	54,87%	17,34%	7,23%	6,54%	1,42%	8,60%	4,00%	9,52%	1,94%
691	11	47,22%	8,31%	3,31%	8,18%	2,39%	22,30%	8,30%	7,73%	6,50%
494	11	33,99%	7,38%	4,76%	11,39%	5,38%	19,11%	17,98%	8,78%	5,04%
502	11	21,07%	8,50%	4,33%	9,31%	3,83%	33,12%	19,85%	6,92%	4,07%
651	11	36,71%	10,09%	5,46%	13,51%	5,59%	14,80%	13,84%	10,14%	8,57%
552	11	41,01%	7,23%	3,13%	8,86%	3,69%	23,79%	12,29%	10,66%	7,44%
666	11	29,22%	13,00%	6,64%	12,13%	5,31%	15,55%	18,14%	9,60%	6,79%
713	12	39,34%	12,53%	4,25%	7,58%	4,73%	17,11%	14,46%	9,22%	6,81%
692	12	52,15%	23,81%	5,97%	3,46%	1,20%	10,52%	2,89%	8,98%	1,42%
210	12	55,47%	12,65%	3,89%	5,81%	2,05%	10,12%	10,02%	5,55%	2,01%
650	12	52,79%	13,91%	5,05%	5,27%	2,35%	15,01%	5,62%	7,78%	3,34%
460	12	52,53%	16,78%	5,27%	4,91%	2,59%	11,86%	6,07%	7,66%	3,15%
717	12	58,28%	18,69%	6,91%	3,08%	1,58%	8,41%	3,05%	7,99%	1,96%
1174	12	50,50%	19,86%	5,99%	6,05%	1,63%	7,72%	8,25%	8,45%	2,17%
395	12	35,34%	11,64%	6,10%	7,63%	5,71%	18,82%	14,77%	9,03%	8,85%
455	12	35,78%	16,65%	7,16%	7,55%	4,63%	16,53%	11,69%	8,24%	6,88%
472	12	54,19%	18,02%	7,53%	6,05%	1,67%	8,67%	3,88%	9,85%	2,27%
303	12	53,87%	19,98%	6,80%	5,42%	1,22%	8,49%	4,23%	8,82%	2,48%
197	12	58,48%	18,02%	4,58%	3,64%	1,04%	10,35%	3,88%	5,93%	1,18%
723	12	40,87%	8,60%	5,26%	9,60%	1,49%	23,77%	10,41%	2,75%	1,10%

No.	Group No.	CE 14:0	CE 15:0	CE 16:1	CE 16:0	CE 18:3	CE 18:2	CE 18:1	CE 18:0	CE 20:4	CE 20:3	CE 20:1	CE 22:6	CE 22:5	CE 22:4	CE 22:1
380	1	1,27%	1,22%	5,24%	12,74%	0,79%	30,32%	15,84%	3,06%	6,48%	0,54%	0,49%	0,48%	0,34%	0,00%	0,32%
649	1	1,22%	1,77%	4,09%	10,95%	0,00%	13,74%	11,68%	2,57%	2,89%	0,65%	0,84%	0,55%	0,62%	0,00%	0,55%
566	1	1,14%	0,65%	4,51%	16,09%	1,03%	8,47%	23,25%	6,48%	1,98%	1,04%	1,49%	0,00%	0,38%	0,55%	0,64%
1349	1	1,18%	1,26%	1,63%	9,42%	0,62%	8,40%	4,99%	7,91%	1,23%	0,53%	0,00%	0,60%	0,59%	0,00%	0,57%
386	1	1,00%	1,19%	4,02%	9,62%	1,06%	9,67%	10,98%	3,54%	1,68%	0,71%	0,61%	0,00%	0,00%	0,00%	0,78%
206	1	1,96%	2,29%	7,56%	10,39%	0,61%	2,38%	6,88%	10,17%	1,14%	0,63%	0,72%	0,72%	0,00%	0,00%	0,69%
399	1	1,42%	1,13%	3,09%	9,37%	0,98%	21,36%	10,52%	4,24%	3,39%	0,97%	0,00%	0,56%	0,42%	0,00%	0,61%
725	1	2,12%	1,51%	7,01%	17,03%	0,82%	11,05%	10,65%	2,85%	2,13%	1,19%	1,40%	0,48%	0,44%	0,44%	0,54%
560	1	1,53%	1,21%	3,25%	9,50%	0,74%	7,50%	8,42%	7,23%	1,17%	0,88%	0,89%	0,00%	0,62%	0,00%	0,86%
436	1	1,08%	1,98%	2,62%	8,73%	0,86%	27,81%	7,83%	4,06%	5,24%	1,00%	0,47%	1,11%	0,00%	0,00%	0,70%
435	1	1,01%	0,91%	4,57%	9,15%	1,15%	21,20%	12,55%	4,19%	3,66%	1,16%	0,00%	0,46%	0,40%	0,38%	0,46%
357	2	1,28%	1,82%	4,94%	10,58%	0,74%	8,01%	9,86%	6,13%	1,45%	1,10%	0,58%	0,69%	0,79%	0,00%	0,55%
638	2	1,13%	0,53%	7,09%	19,74%	0,84%	19,00%	24,07%	2,22%	8,79%	3,59%	1,15%	1,05%	0,52%	0,83%	0,96%
727	2	1,70%	1,58%	4,41%	10,27%	0,91%	18,49%	10,85%	3,67%	3,98%	0,97%	0,68%	0,55%	0,60%	0,00%	0,74%
252	2	0,87%	0,92%	7,43%	19,16%	0,47%	12,87%	30,59%	4,43%	5,13%	2,37%	1,53%	0,65%	0,86%	2,27%	0,47%
549	2	1,17%	0,56%	4,09%	18,62%	1,98%	35,04%	16,80%	1,85%	5,63%	1,43%	0,65%	0,34%	0,57%	0,71%	0,49%
553	2	1,66%	1,35%	4,98%	19,97%	1,16%	17,12%	16,96%	7,75%	3,50%	1,65%	0,88%	0,55%	0,82%	0,48%	0,57%
1227	2	1,69%	1,08%	5,71%	11,75%	1,64%	33,53%	19,74%	2,46%	4,62%	1,23%	0,33%	0,81%	0,26%	0,27%	0,61%
563	2	1,30%	0,82%	6,90%	14,70%	1,36%	19,48%	20,65%	3,59%	5,25%	1,68%	0,79%	0,60%	0,39%	0,63%	0,52%
378	2	1,60%	2,30%	3,33%	16,23%	0,62%	7,54%	9,13%	4,59%	1,08%	0,00%	1,53%	0,77%	0,60%	0,00%	0,50%
344	2	0,86%	0,43%	4,63%	14,76%	0,40%	6,75%	37,13%	1,92%	14,36%	6,62%	1,96%	0,41%	1,08%	3,83%	1,05%
202	3	2,02%	1,58%	7,62%	19,17%	1,31%	11,67%	14,15%	3,86%	5,45%	1,78%	1,26%	0,63%	0,68%	0,55%	0,29%
1178	3	2,40%	2,76%	3,12%	18,44%	0,76%	2,75%	7,33%	4,13%	2,42%	0,00%	1,25%	0,00%	1,16%	0,00%	0,00%
612	4	1,68%	1,31%	4,13%	12,81%	1,34%	20,59%	11,44%	1,73%	3,24%	0,71%	0,53%	0,84%	0,00%	0,57%	0,52%
550	4	0,65%	0,39%	4,60%	14,99%	2,07%	31,65%	22,30%	1,56%	5,53%	1,27%	0,57%	0,59%	0,31%	0,68%	0,20%
639	4	1,11%	0,49%	6,56%	14,31%	3,84%	18,63%	19,76%	5,42%	8,42%	1,59%	0,44%	0,80%	0,35%	0,28%	0,26%
818	4	1,40%	1,71%	1,55%	4,52%	0,00%	1,69%	2,99%	2,66%	0,00%	0,00%	0,00%	0,00%	0,00%	0,00%	0,00%
495	4	1,43%	1,08%	3,76%	13,20%	0,64%	17,38%	13,25%	2,19%	2,90%	0,75%	1,10%	0,00%	0,70%	0,00%	0,00%
1167	4	1,99%	1,09%	3,76%	20,00%	1,50%	29,51%	16,67%	2,44%	3,37%	0,72%	0,87%	0,85%	0,74%	0,19%	0,24%
885	4	1,36%	0,45%	4,95%	20,58%	0,93%	7,62%	26,04%	2,10%	6,33%	5,42%	1,23%	0,90%	0,40%	0,73%	0,68%
256	4	1,31%	0,91%	4,00%	13,80%	1,89%	22,46%	13,68%	2,09%	4,25%	1,28%	0,54%	0,72%	0,29%	0,60%	0,37%
260	4	1,95%	1,95%	4,32%	19,01%	1,02%	14,25%	16,31%	3,94%	5,44%	1,89%	0,76%	1,44%	0,64%	1,49%	0,66%
600	4	2,19%	3,11%	2,81%	16,31%	0,70%	10,05%	9,43%	4,03%	3,20%	1,31%	1,01%	0,61%	0,66%	0,63%	0,67%
1299	4	2,24%	1,53%	5,28%	17,49%	0,59%	5,71%	15,37%	4,04%	4,49%	2,94%	1,01%	0,68%	0,57%	1,47%	0,63%
653	4	1,28%	1,10%	5,47%	11,10%	1,64%	12,60%	13,98%	1,98%	3,54%	2,49%	0,60%	0,79%	0,60%	0,00%	0,63%
313	4	2,10%	1,80%	4,44%	9,45%	0,90%	4,65%	6,13%	4,28%	1,27%	0,93%	0,00%	0,00%	0,00%	0,83%	1,18%
396	4	1,73%	0,43%	5,76%	15,85%	0,98%	26,76%	24,19%	2,78%	10,37%	3,19%	0,41%	0,99%	0,34%	0,63%	0,32%
1340	4	1,36%	1,51%	2,72%	9,74%	1,11%	12,16%	10,06%	2,13%	4,96%	1,86%	0,78%	0,50%	0,57%	0,43%	0,54%
688	4	1,03%	0,47%	4,69%	12,91%	1,56%	36,90%	24,69%	1,68%	7,31%	0,78%	0,16%	0,78%	0,17%	0,18%	0,27%
907	4	1,40%	1,03%	5,51%	17,56%	0,65%	17,63%	18,89%	2,94%	4,51%	1,24%	1,11%	0,79%	0,57%	0,78%	0,35%
350	4	1,86%	2,11%	4,72%	8,45%	1,10%	8,28%	7,92%	2,10%	1,43%	0,00%	0,74%	1,17%	0,00%	0,00%	0,70%
1313	5	1,68%	0,86%	5,02%	13,11%	0,95%	33,36%	18,18%	1,67%	4,53%	1,07%	0,23%	0,43%	0,30%	0,23%	0,26%
1180	5	2,90%	1,15%	6,90%	18,35%	1,80%	23,09%	17,04%	2,19%	7,50%	1,11%	0,44%	0,46%	0,48%	0,52%	0,22%
261	5	1,54%	0,98%	4,36%	17,74%	0,98%	17,49%	12,42%	1,29%	4,97%	0,69%	0,49%	0,68%	0,00%	0,51%	0,64%
644	5	1,83%	1,01%	5,98%	17,23%	1,13%	26,85%	17,84%	2,02%	6,23%	1,78%	0,61%	0,29%	0,45%	0,39%	0,29%
264	5	1,45%	0,97%	6,94%	18,80%	0,71%	21,90%	19,96%	2,40%	7,34%	2,54%	0,64%	0,47%	0,57%	0,96%	0,34%
347	6	1,16%	0,78%	4,50%	20,49%	1,13%	26,87%	20,66%	2,32%	6,68%	3,68%	0,61%	0,52%	0,64%	1,39%	0,23%
316	6	0,93%	1,10%	3,60%	18,75%	0,66%	12,86%	13,62%	2,70%	3,27%	1,12%	0,66%	0,34%	0,57%	0,50%	0,49%
1323	6	1,99%	0,78%	5,66%	20,33%	0,97%	13,43%	21,31%	2,48%	6,85%	3,05%	1,01%	0,62%	0,80%	1,40%	0,42%
235	6	1,27%	0,62%	3,76%	12,23%	1,08%	31,35%	12,79%	3,72%	6,79%	0,89%	0,14%	0,48%	0,22%	0,21%	0,22%
721	6	1,79%	0,92%	7,70%	16,37%	1,51%	21,00%	17,91%	1,85%	5,17%	1,03%	0,54%	0,43%	0,37%	0,23%	0,25%
345	6	0,44%	0,51%	3,26%	12,58%	0,84%	37,05%	18,12%	1,62%	6,62%	1,81%	0,42%	0,52%	0,46%	0,50%	0,21%
603	6	1,26%	0,71%	5,32%	30,65%	0,63%	7,34%	21,61%	4,18%	6,91%	2,34%	1,03%	0,50%	0,68%	1,18%	0,40%
269	6	1,34%	0,75%	6,30%	19,25%	1,09%	8,32%	20,84%	3,26%	4,64%	1,50%	1,03%	0,42%	0,42%	0,91%	0,66%
273	7	0,91%	1,17%	2,30%	12,82%	0,14%	5,81%	9,17%	2,80%	1,70%	1,36%	0,54%	0,23%	0,27%	0,61%	0,42%
706	7	1,14%	0,93%	3,75%	15,00%	0,74%	21,52%	20,58%	1,97%	4,35%	1,93%	0,98%	0,47%	0,48%	0,68%	0,49%
674	7	2,98%	2,12%	4,77%	31,25%	0,39%	6,47%	11,02%	2,54%	0,71%	0,25%	1,90%	0,43%	1,11%	0,26%	0,37%
697	7	1,21%	2,15%	2,48%	10,10%	0,00%	11,32%	6,37%	3,02%	1,59%	0,00%	0,00%	0,73%	0,78%	0,83%	0,00%
275	7	1,58%	1,02%	5,18%	20,74%	0,69%	12,48%	18,61%	1,93%	8,69%	3,86%	1,07%	1,06%	0,83%	0,87%	0,45%
304	7	1,47%	1,67%	3,62%	7,92%	0,00%	11,21%	7,49%	1,80%	1,94%	0,00%	0,00%	1,08%	0,00%	0,00%	0,81%
544	7	1,00%	0,95%	3,29%	15,85%	1,66%	41,54%	17,62%	1,31%	4,16%	0,37%	0,57%	0,42%	0,27%	0,00%	0,14%
687	7	1,90%	2,88%	3,61%	20,48%	0,00%	2,86%	7,31%	4,20%	0,00%	0,00%	0,91%	0,00%	0,00%	0,00%	0,79%
1208	7	1,30%	2,16%	3,76%	14,22%	0,65%	8,31%	9,51%	1,79%	1,40%	0,62%	0,66%	0,80%	0,00%	0,00%	0,00%
266	8	3,22%	0,86%	9,68%	18,11%	2,33%	32,40%	15,46%	1,39%	7,21%	1,04%	0,13%	0,48%	0,22%	0,21%	0,10%
487	8	1,23%	2,18%	3,60%	8,11%	0,00%	5,47%	7,63%	5,07%	1,29%	1,04%	0,70%	0,00%	1,30%	0,00%	0,00%
548	8	1,23%	1,06%	9,52%	22,92%	0,45%	5,38%	28,22%	2,42%	3,51%	2,94%	1,47%	0,36%	0,63%	1,00%	0,63%
346	8	1,35%	1,94%	3,83%	15,92%	0,00%	3,94%	8,98%	2,24%	1,48%	1,17%	1,07%	0,00%	0,00%	0,00%	0,96%
1300	9	1,31%	1,55%	3,01%	8,60%	0,00%	9,25%	8,89%	4,41%	1,52%	0,00%	0,00%	0,62%	0,00%	0,65%	0,66%
199	9	1,18%	0,15%	15,43%	10,45%	2,39%	15,83%	37,36%	0,76%	6,41%	5,21%	0,38%	0,29%	0,19%	0,17%	0,13%
719	9	0,80%	0,73%	4,08%	11,78%	1,23%	35,46%	15,32%	1,43%	2,50%	0,43%	0,43%	0,59%	0,31%	0,32%	0,25%
393	9	1,45%	0,70%	6,16%	20,05%	1,97%	20,62%	16,64%	4,43%	8,20%	3,33%	0,29%	0,67%	0,53%	0,63%	0,25%
541	9	1,01%	0,72%	5,42%	25,15%	0,93%	27,30%	17,83%	1,30%	3,93%	0,85%	0,24%	0,44%	0,26%	0,32%	0,15%
258	9	1,16%	0,45%	9,04%	13,14%	2,11%	26,28%	24,72%	1,95%	10,33%	2,95%	0,28%	1,53%	0,46%	0,87%	0,10%
715	9	2,47%	2,37%	8,24%	16,37%	2,42%	13,45%	16,15%	3,21%	6,46%	1,05%	0,90%	0,63%	0,37%	0,31%	0,45%
253	9	0,63%	0,22%	3,77%	7,49%	3,02%	61,10%	15,68%	0,42%	5,09%	0,84%	0,13%	0,19%	0,13%	0,08%	0,02%
636	9	0,00%	3,33%	2,89%	7,84%	0,00%	4,63%	7,07%	3,38%	0,00%	0,00%	0,00%	0,00%	0,00%	1,71%	0,00%
675	9	1,19%	0,93%	3,79%	20,35%	0,83%	8,71%	11,68%	17,80%	5,57%	1,39%	0,61%	1,94%	0,63%	1,25%	0,55%
1326	9	0,82%	0,00%	3,97%	12,15%	0,67%	6,82%	13,47%	4,10%	12,44%	3,97%	0,00%	0,95%	0,84%	1,05%	1,00%
68	9	2,12%	2,61%	4,65%	41,64%	0,40%	5,26%	9,04%	1,96%	0,78%	0,00%	1,05%	0,42%	0,69%	0,33%	0,39%
227	9	0,85%	0,63%	3,86%	57,52%	0,25%	5,86%	17,74%	1,38%	3,97%	2,00%	0,50%	0,07%	0,29%	0,73%	0,17%
594	9	0,39%	0,63%	1,66%	85,11%	0,10%	3,16%	3,39%	1,02%	0,58%	0,30%	0,08%	0,05%	0,07%	0,09%	0,06%
659	9	1,34%	0,88%	5,79%	12,85%	1,41%	35,96%	16,87%	4,37%	6,76%	0,86%	0,34%	0,57%	0,27%	0,00%	0,22%
507	9	0,00%	1,69%	2,59%	3,92%	0,00%	2,10%	3,43%	2,53%	1,03%	0,00%	0,00%	0,00%	0,92%	0,00%	1,13%
451	9	1,79%	2,21%	3,60%	17,55%	0,00%	7,36%	8,87%	5,40%	2,20%	0,00%	0,93%	1,08%	0,81%	0,58%	0,66%
454	9	1,33%	0,83%	4,74%	18,47%	0,82%	14,19%	15,80%	9,67%	5,48%	1,44%	0,30%	0,61%	0,50%	0,54%	0,38%
641	9	0,82%	0,46%	3,01%	56,80%	0,58%	11,36%	10,12%	1,93%	7,86%	1,66%	0,35%	0,55%	0,46%	1,33%	0,20%
336	9	0,70%	0,53%	4,98%	37,40%	0,26%	9,56%	15,65%	1,64%	11,32%	4,25%	0,38%	0,00%	0,53%	2,19%	0,22%
240	9	1,32%	0,85%	4,18%	33,94%	0,56%	11,48%	20,62%	4,43%	2,18%	1,40%	1,30%	0,25%	0,24%	1,73%	0,73%
571	10	0,92%	1,35%	3,19%	9,92%	0,60%	7,24%	9,11%	5,48%	1,52%	1,26%	0,76%	0,00%	0,00%	0,00%	0,63%
490	10	1,50%	1,58%	4,81%	13,27%	1,71%	28,23%	16,10%	1,68%	3,44%	0,91%	0,51%	0,49%	0,36%	0,34%	0,34%
429	10	1,13%	2,14%	3,97%	11,52%	1,26%	3,62%	15,78%	7,42%	2,99%	2,74%	1,24%	0,95%	1,72%	1,02%	1,54%
267	10	1,91%	2,82%	5,62%	13,22%	1,51%	9,41%	11,12%	1,83%	1,96%	0,95%	1,94%	0,79%	0,79%	0,00%	1,15%
306	10	0,00%	1,20%	3,09%	7,67%	1,13%	29,01%	14,60%	1,86%	4,74%	1,01%	0,00%	0,00%	0,00%	0,00%	0,92%
707	10	1,13%	0,27%	6,50%	31,33%	0,38%	5,05%	31,89%	2,01%	9,37%	4,69%	1,02%	0,25%	0,65%	1,54%	0,65%
207	10	1,13%	1,19%	4,13%	12,56%	1,10%	33,14%	17,15%	1,86%	4,33%	0,55%	0,53%	0,37%	0,36%	0,00%	0,00%
1318	11	1,81%	1,58%	5,13%	17,76%	1,18%	15,42%	11,89%	5,35%	3,62%	1,68%	1,19%	0,54%	0,75%	0,69%	0,44%
691	11	0,91%	0,55%	4,63%	15,22%	2,09%	28,54%	23,53%	1,75%	8,10%	1,88%	0,69%	0,56%	0,88%	1,22%	0,39%
494	11	0,90%	0,39%	3,63%	29,14%	2,10%	38,43%	13,61%	0,75%	4,05%	1,10%	0,26%	0,20%	0,15%	0,22%	0,18%
502	11	1,30%	0,90%	6,18%	12,86%	0,69%	14,44%	14,97%	3,20%	3,69%	1,55%	0,80%	0,48%	0,42%	0,71%	0,59%
651	11	2,36%	1,22%	5,15%	21,06%	0,55%	6,89%	18,54%	3,34%	7,68%	6,08%	0,79%	0,31%	0,56%	1,66%	0,69%
552	11	1,21%	0,59%	6,32%	49,72%	0,21%	5,16%	19,61%	1,98%	5,39%	1,76%	0,59%	0,36%	0,61%	1,25%	0,21%
666	11	0,43%	0,37%	3,22%	9,95%	1,31%	49,95%	16,93%	1,55%	6,80%	1,03%	0,11%	0,68%	0,12%	0,06%	0,08%
713	12	1,12%	0,59%	5,39%	21,09%	0,86%	13,16%	27,96%	1,74%	11,05%	3,26%	0,78%	1,04%	0,61%	1,38%	0,35%
692	12	1,71%	1,55%	5,13%	26,68%	0,69%	9,28%	15,22%	3,28%	1,59%	1,16%	1,57%	0,89%	1,19%	0,40%	0,45%
210	12	1,42%	1,23%	9,86%	20,81%	0,78%	9,50%	24,44%	5,98%	7,19%	0,90%	0,91%	0,81%	0,37%	0,42%	0,77%
650	12	1,15%	0,17%	4,55%	17,25%	1,44%	30,35%	21,71%	1,05%	8,85%	3,37%	0,51%	0,51%	0,41%	0,55%	0,18%
460	12	1,15%	0,66%	3,24%	11,51%	1,67%	31,43%	17,50%	2,21%	3,41%	1,12%	0,25%	0,32%	0,25%	0,00%	0,32%
717	12	1,46%	0,00%	4,11%	22,02%	0,00%	14,80%	11,86%	1,67%	4,32%	0,00%	0,89%	0,92%	0,00%	0,00%	0,00%
1174	12	2,42%	2,88%	7,51%	28,50%	0,00%	3,24%	15,11%	6,00%	1,35%	0,95%	1,72%	0,76%	0,00%	1,66%	1,32%
395	12	1,68%	0,71%	3,89%	21,40%	0,53%	7,19%	24,03%	4,38%	5,59%	4,83%	1,43%	0,40%	0,50%	1,56%	0,77%
455	12	1,94%	3,36%	5,90%	18,35%	0,71%	14,14%	13,93%	5,63%	3,64%	1,42%	0,79%	0,65%	0,00%	1,09%	0,73%
472	12	0,98%	0,75%	4,73%	14,34%	0,99%	10,47%	21,41%	5,01%	5,09%	1,28%	0,52%	1,05%	0,41%	0,66%	0,47%
303	12	1,21%	0,60%	8,22%	14,95%	1,75%	28,19%	22,40%	2,17%	7,48%	2,17%	0,32%	0,80%	0,25%	0,32%	0,22%
197	12	1,97%	1,51%	5,82%	28,24%	0,63%	9,55%	18,00%	4,67%	2,44%	1,52%	1,25%	0,72%	0,49%	0,60%	0,50%
723	12	1,59%	1,80%	10,72%	18,95%	0,76%	6,90%	17,88%	2,61%	3,36%	2,15%	1,08%	0,98%	1,28%	1,53%	0,83%
