# Supplementary material for: Surfactant Lipidomics in Healthy Children and Childhood Interstitial Lung Disease
Source: PLoS One. 2015 Feb 18;10(2):e0117985. doi: 10.1371/journal.pone.0117985 (PMC4333572; doi:10.1371/journal.pone.0117985)
Supplement: S2 Table — The individual values of the other phospholipid classes and their species are depicted in the S1 Table. (PDF) [file pone.0117985.s008.pdf]

Phosphatidylcholine and phosphatidylglycerol and their species composition in BAL from healthy children (n = 11) in comparison to children with two disease causing ABCA3 mutations (n = 5). The individual values of the other phospholipid classes and their species are depicted in Table S1.

	Controls healthy	ABCA3 2 Mutations	P (corrected for multiple comparisons, significant if < 0.0135)*
Phosphatidylcholine, PC [% of total PL#]	85.9 ± 2.6	55.6 ± 28.8	0.003*
PC-Species [% of total PC]:			
PC 30:0	8.2 ± 1.8	3.6 ± 3.8	0.054
PC 30:1	0.3 ± 0.1	0.2 ± 0.2	0.395
PC 32:0	45.8 ± 3.6	18.3 ± 17.7	0.017
PC 32:1	8.8 ± 2.5	4.1 ± 3.3	0.023
PC 32:2	0.4 ± 0.2	0.5 ± 0.2	0.212
PC 34:0	1.6 ± 0.5	1.2 ± 0.6	0.533
PC 34:1	11.1 ± 2.3	14.9 ± 4.3	0.069
PC 34:2	5.4 ± 1.2	9.8 ± 11.4	0.821
PC 34:3	0.5 ± 0.2	0.4 ± 0.6	0.070
PC 36:1	1.1 ± 0.3	3.2 ± 2.2	0.036
PC 36:2	2.4 ± 0.8	7.1 ± 5.1	0.031
PC 36:3	1.6 ± 0.6	3.4 ± 2.7	0.213
PC 36:4	1.4 ± 0.4	3.2 ± 2.8	0.070
PC 38:4	0.4 ± 0.2	1.9 ± 1.2	0.070
PC 38:5	0.3 ± 0.1	1.0 ± 0.9	0.002*
PC O-32:0	2.5 ± 0.8	2.4 ± 1.6	0.734
PC O-32:1	0.5 ± 0.1	1.5 ± 1.2	0.428
PC O-34:0	1.7 ± 0.7	1.3 ± 0.9	0.308
PC O-34:1	1.2 ± 0.3	2.2 ± 1.5	0.141
PC O-34:2	0.2 ± 0.0	1.3 ± 0.9	0.002*

PC O-36:4	0.2 ± 0.1	0.8 ± 0.5	0.070
Phosphatidylglycerol [% of total PL]	3.8 ± 0.5	2.0 ±2.1	0.070
Species [% of total PG]:			
PG 30:0	0.9 ±0.4	2.4 ±2.1	0.215
PG 32:0	5.0 ±0.8	7.6 ±13.7	0.113
PG 32:1	2.2 ±0.8	0.5 ±0.6	0.003*
PG 34:0	1.9 ±0.7	0.6 ±1.3	0.054
PG 34:1	29.8 ±4.4	31.9 ±39.3	0.141
PG 34:2	4.3 ±1.3	3.5 ±6.4	0.113
PG 35:1	1.2 ±0.5	0.0 ±0.0	0.001*
PG 36:1	17.2 ±4.0	3.7 ±5.1	0.005*
PG 36:2	18.1 ± 6.3	5.3 ±9.5	0.042
PG 36:3	2.8 ±2.0	5.9 ±11.1	0.428
PG 36:4	2.1 ±0.5	0.3 ±0.7	0.003*
PG 38:4	1.6 ±0.6	0.2 ±0.5	0.007*
PG 38:5	1.5 ±0.3	0.3 ±0.6	0.007*
PG 38:6	0.8 ±0.4	0.0 ±0.0	0.001*

*For comparison of the two groups Mann-Whitney test was used. The P values given are uncorrected and were calculated for the initial $P < 0.05$. To account for the multiple comparisons made, a corrected P values of 0.0129 was calculated by false discovery rate method.

#Total phospholipid was 65.4 ± 117 nmol/ml in controls and 621 ± 1315 nmol/ml in ABCA3 patients. All data are means ± standard deviation.
