# Supplementary material for: Surfactant Lipidomics in Healthy Children and Childhood Interstitial Lung Disease
Source: PLoS One. 2015 Feb 18;10(2):e0117985. doi: 10.1371/journal.pone.0117985 (PMC4333572; doi:10.1371/journal.pone.0117985)
Supplement: S3 Table — (PDF) [file pone.0117985.s009.pdf]

Columns Statistics

All Phospholipids analyzed

	Controls healthy	Controls Bronchitis	Diffuse development. disorder	Growth abn. + defic. alveolar.	Immuno-intact host	Immuno-compromised host	Chronic tachypnea of infancy	Reactive lymphoid lesions	Related to alv. surf. region	Related to lung vessels/heart	Related to systemic disease	Unclear RDS in mature neonate
**Number of values**	11	10	2	18	5	8	9	4	21	7	7	13
**Minimum**	13,46	21,86	13,69	1,12	14,82	21,7	27,32	15,84	2,8	12,88	22,94	6,29
**25% Percentile**	22,31	34		16,02	17,52	28,88	50,22	16,42	12,75	20,69	25,21	28,58
**Median**	28,82	50,54	46,87	48,14	20,61	50,49	97,29	22,75	23,98	37,25	38,98	53,94
**75% Percentile**	44,88	161		312,3	58,09	116,2	247,5	59,73	67,4	390,1	70,75	117,2
**Maximum**	415,2	614,6	80,04	1492	66,24	189,6	375,8	90,95	2973	1474	126,7	282,4
**Mean**	65,4	127,8	46,87	212,4	34,36	75,32	140,7	38,07	196,2	290,8	54,9	84,24
**Std. Deviation**	116,7	180,2	46,92	382,3	22,53	61,23	135,7	35,71	642,1	538,5	36,59	89,29
**Std. Error**	35,19	56,99	33,18	90,1	10,07	21,65	45,24	17,86	140,1	203,5	13,83	24,76
**Lower 95% CI of mean**	-13,01	-1,144	-374,7	22,32	6,395	24,12	36,4	-18,76	-96,07	-207,3	21,07	30,29
**Upper 95% CI of mean**	143,8	256,7	468,4	402,5	62,33	126,5	245	94,9	488,5	788,8	88,74	138,2
**Sum**	719,4	1278	93,73	3824	171,8	602,5	1266	152,3	4121	2035	384,3	1095

Phosphatidylcholine

	Controls healthy	Controls Bronchitis	Diffuse development. disorder	Growth abn. + defic. alveolar.	Immuno-intact host	Immuno-compromised host	Chronic tachypnea of infancy	Reactive lymphoid lesions	Related to alv. surf. region	Related to lung vessels/heart	Related to systemic disease	Unclear RDS in mature neonate
**Number of values**	11	10	2	18	5	8	9	4	19	7	7	13
**Minimum**	81,6	58,5	74	37,5	78,4	54,5	75,63	78,6	12,4	42	55,7	50,7
**25% Percentile**	84,7	75,75		78,2	79,2	58,85	85,2	80,7	45,2	72,5	59	69,4
**Median**	85,5	82,5	76,1	82,5	82,5	81,4	87,4	83,05	72,4	82,4	76,2	79,2
**75% Percentile**	87,7	86		86,5	85,55	85,95	88,45	86,6	82,2	88,5	81,5	84,95
**Maximum**	91,1	88,3	78,2	89,6	87,4	89,7	88,9	89,9	88,9	90,3	86,4	90,8
**Mean**	85,9	79,91	76,1	77,52	82,4	74,58	86,01	83,65	62,18	77,09	72,13	76,47
**Std. Deviation**	2,583	8,719	2,97	14,98	3,481	14,6	4,136	4,669	23,91	16,5	11,6	10,95
**Std. Error**	0,7789	2,757	2,1	3,532	1,557	5,16	1,379	2,335	5,486	6,237	4,384	3,036
**Lower 95% CI of mean**	84,16	73,67	49,42	70,07	78,08	62,37	82,84	76,22	50,66	61,83	61,4	69,85
**Upper 95% CI of mean**	87,64	86,15	102,8	84,97	86,72	86,78	89,19	91,08	73,71	92,35	82,86	83,08
**Sum**	944,9	799,1	152,2	1395	412	596,6	774,1	334,6	1182	539,6	504,9	994,1

Sphingomyelin

	Controls healthy	Controls Bronchitis	Diffuse development. disorder	Growth abn. + defic. alveolar.	Immuno-intact host	Immuno-compromised host	Chronic tachypnea of infancy	Reactive lymphoid lesions	Related to alv. surf. region	Related to lung vessels/heart	Related to systemic disease	Unclear RDS in mature neonate
**Number of values**	11	10	2	18	5	8	9	4	19	7	7	13
**Minimum**	0,6	1	2,3	0	1,9	1	0,3	1,1	1,1	0,5	2,5	0,8
**25% Percentile**	0,9	1,25		1,15	2	1,7	0,75	1,1	1,6	0,7	3	1,8
**Median**	1,6	2,2	2,3	1,7	3,6	3,55	1	2,1	7,3	2,7	4,6	3,3
**75% Percentile**	1,9	7,05		2,8	4,25	12,7	1,7	3,15	11,5	3,4	13,1	6,65
**Maximum**	3,3	13,3	2,3	17,2	4,7	14,3	2,4	3,2	28,5	11,8	15,4	11,7
**Mean**	1,564	4	2,3	2,739	3,22	6,4	1,189	2,125	8,353	3,371	6,929	4,369
**Std. Deviation**	0,8152	4,286	0	3,778	1,19	5,81	0,6716	1,184	7,39	3,884	5,157	3,211
**Std. Error**	0,2458	1,355	0	0,8904	0,5324	2,054	0,2239	0,5921	1,695	1,468	1,949	0,8906
**Lower 95% CI of mean**	1,016	0,9337	2,3	0,8602	1,742	1,543	0,6726	0,2405	4,791	-0,2207	2,159	2,429
**Upper 95% CI of mean**	2,111	7,066	2,3	4,618	4,698	11,26	1,705	4,009	11,91	6,964	11,7	6,31
**Sum**	17,2	40	4,6	49,3	16,1	51,2	10,7	8,5	158,7	23,6	48,5	56,8

Phosphatidylethanolamine

	Controls healthy	Controls Bronchitis	Diffuse development. disorder	Growth abn. + defic. alveolar.	Immuno-intact host	Immuno-compromised host	Chronic tachypnea of infancy	Reactive lymphoid lesions	Related to alv. surf. region	Related to lung vessels/heart	Related to systemic disease	Unclear RDS in mature neonate
**Number of values**	11	10	2	18	5	8	9	4	19	7	7	13
**Minimum**	1,5	1,8	2	1,4	2,1	2	1,9	1,7	0,9	1,5	2	1,2
**25% Percentile**	2,1	1,9		2,3	2,2	2,15	2,1	1,9	2,2	1,8	2,2	2,1
**Median**	2,3	2,55	2,35	2,6	2,3	2,3	2,34	2,35	2,8	2,3	2,7	2,5
**75% Percentile**	2,4	2,7		3,05	2,65	3,55	2,6	2,85	4,6	3	4,6	3,45
**Maximum**	2,5	3,2	2,7	4,7	2,9	5,1	2,9	3,1	8,4	6,9	5,2	3,9
**Mean**	2,218	2,38	2,35	2,672	2,4	2,888	2,349	2,375	3,368	2,886	3,157	2,623
**Std. Deviation**	0,2994	0,4756	0,495	0,7676	0,3	1,28	0,3317	0,6076	1,859	1,834	1,235	0,8064
**Std. Error**	0,09027	0,1504	0,35	0,1809	0,1342	0,4525	0,1106	0,3038	0,4264	0,6933	0,4669	0,2237
**Lower 95% CI of mean**	2,017	2,04	-2,097	2,291	2,027	1,817	2,094	1,408	2,473	1,189	2,015	2,136
**Upper 95% CI of mean**	2,419	2,72	6,797	3,054	2,773	3,958	2,604	3,342	4,264	4,582	4,3	3,11
**Sum**	24,4	23,8	4,7	48,1	12	23,1	21,14	9,5	64	20,2	22,1	34,1

Plasmalogen

	Controls healthy	Controls Bronchitis	Diffuse development. disorder	Growth abn. + defic. alveolar.	Immuno-intact host	Immuno-compromised host	Chronic tachypnea of infancy	Reactive lymphoid lesions	Related to alv. surf. region	Related to lung vessels/heart	Related to systemic disease	Unclear RDS in mature neonate

**Number of values**	11	10	2	18	5	8	9	4	19	7	7	13
**Minimum**	0,8	1,6	4,5	0,9	1,5	1,2	1,1	1,8	1,8	1	1,5	1,5
**25% Percentile**	1,5	2,45		2,3	1,9	2,35	1,25	1,95	2,3	1,7	3,5	3,05
**Median**	2,3	3,2	5,15	3,7	3	3,65	1,7	2,15	4,3	2,7	5,1	3,5
**75% Percentile**	2,6	5,35		5,25	3,6	9,55	2,74	3,35	15,9	5,3	7,6	7,35
**Maximum**	3,8	8,2	5,8	12,1	3,8	11,9	3	4,5	25,3	18,3	10,1	10,5
**Mean**	2,245	3,85	5,15	4,6	2,8	5,525	1,876	2,65	8,874	4,957	5,414	4,962
**Std. Deviation**	0,8454	2,011	0,9192	3,319	0,9138	4,259	0,7449	1,245	7,599	6,045	2,864	3,051
**Std. Error**	0,2549	0,636	0,65	0,7824	0,4087	1,506	0,2483	0,6225	1,743	2,285	1,082	0,8463
**Lower 95% CI of mean**	1,678	2,411	-3,109	2,949	1,665	1,964	1,303	0,6689	5,211	-0,6334	2,766	3,118
**Upper 95% CI of mean**	2,813	5,289	13,41	6,251	3,935	9,086	2,448	4,631	12,54	10,55	8,063	6,805
**Sum**	24,7	38,5	10,3	82,8	14	44,2	16,88	10,6	168,6	34,7	37,9	64,5

Phosphatidylserine

	**Controls healthy**	**Controls Bronchitis**	**Diffuse development. disorder**	**Growth abn. + defic. alveolar.**	**Immuno-intact host**	**Immuno-compromised host**	**Chronic tachypnea of infancy**	**Reactive lymphoid lesions**	**Related to alv. surf. region**	**Related to lung vessels/heart**	**Related to systemic disease**	**Unclear RDS in mature neonate**
**Number of values**	11	10	2	18	5	8	9	4	19	7	7	13
**Minimum**	1,5	2,6	5,7	1,2	2,4	1,9	1,9	2,5	0,1	1,4	2,2	2
**25% Percentile**	2,6	2,8		2,9	3,05	2,8	2,2	2,9	3,7	2	3,9	3,15
**Median**	3,3	3,85	6,8	3,8	3,8	3,65	2,6	4,35	8,1	3,9	5,8	4,6
**75% Percentile**	3,6	5		6,15	5,7	9,35	3,15	5,45	16,3	6,7	8,6	7,95
**Maximum**	3,7	9,7	7,9	34,1	6,6	10,2	3,7	5,5	26	16	12,4	25,5
**Mean**	3,082	4,33	6,8	6,783	4,26	5,463	2,678	4,175	10,59	5,557	6,443	6,538
**Std. Deviation**	0,7054	2,101	1,556	7,957	1,561	3,525	0,62	1,509	7,967	4,955	3,476	6,147
**Std. Error**	0,2127	0,6643	1,1	1,876	0,6983	1,246	0,2067	0,7543	1,828	1,873	1,314	1,705
**Lower 95% CI of mean**	2,608	2,827	-7,177	2,826	2,321	2,515	2,201	1,774	6,755	0,9747	3,228	2,824
**Upper 95% CI of mean**	3,556	5,833	20,78	10,74	6,199	8,41	3,154	6,576	14,43	10,14	9,657	10,25
**Sum**	33,9	43,3	13,6	122,1	21,3	43,7	24,1	16,7	201,3	38,9	45,1	85

Phosphatidylglycerol

	**Controls healthy**	**Controls Bronchitis**	**Diffuse development. disorder**	**Growth abn. + defic. alveolar.**	**Immuno-intact host**	**Immuno-compromised host**	**Chronic tachypnea of infancy**	**Reactive lymphoid lesions**	**Related to alv. surf. region**	**Related to lung vessels/heart**	**Related to systemic disease**	**Unclear RDS in mature neonate**
**Number of values**	11	10	2	18	5	8	9	4	19	7	7	13
**Minimum**	3,2	1,9	0,7	0,7	2,4	1,2	2,8	1,5	0	0,7	1,3	0,2
**25% Percentile**	3,2	2,8		1,75	2,65	1,95	3,2	2,6	0,5	2,9	1,7	0,8
**Median**	3,7	3,45	0,8	2,1	3,2	2,75	4	4,05	1,5	3,7	2,4	1,4
**75% Percentile**	4,4	4,15		3,7	3,35	3,7	4,2	4,5	3,2	4,4	3,2	2,25
**Maximum**	4,7	4,4	0,9	9,1	3,4	4,4	5,1	4,6	5,2	4,5	3,7	3,6
**Mean**	3,764	3,39	0,8	2,833	3,04	2,8	3,844	3,55	1,795	3,329	2,343	1,515
**Std. Deviation**	0,5573	0,7622	0,1414	1,982	0,4037	1,122	0,6966	1,42	1,552	1,307	0,8658	0,9737
**Std. Error**	0,168	0,241	0,1	0,4671	0,1806	0,3969	0,2322	0,71	0,3561	0,4941	0,3272	0,2701
**Lower 95% CI of mean**	3,389	2,845	-0,4706	1,848	2,539	1,862	3,309	1,29	1,047	2,12	1,542	0,927
**Upper 95% CI of mean**	4,138	3,935	2,071	3,819	3,541	3,738	4,38	5,81	2,543	4,538	3,144	2,104
**Sum**	41,4	33,9	1,6	51	15,2	22,4	34,6	14,2	34,1	23,3	16,4	19,7

Lysophosphatidylcholine

	**Controls healthy**	**Controls Bronchitis**	**Diffuse development. disorder**	**Growth abn. + defic. alveolar.**	**Immuno-intact host**	**Immuno-compromised host**	**Chronic tachypnea of infancy**	**Reactive lymphoid lesions**	**Related to alv. surf. region**	**Related to lung vessels/heart**	**Related to systemic disease**	**Unclear RDS in mature neonate**
**Number of values**	11	10	2	18	5	8	9	4	19	7	7	13
**Minimum**	0,64	0,83	1,28	0,76	0,96	0,48	0,1	1,09	0,57	0,75	1,47	0,79
**25% Percentile**	0,71	0,9		1,045	1,05	0,775	0,39	1,165	1,17	0,79	1,69	1,51
**Median**	0,96	1,03	5,385	1,46	1,65	1,16	0,67	1,365	1,94	1,56	2,03	1,85
**75% Percentile**	1,28	2,33		3,215	2,14	1,845	1,185	1,51	4,28	3,28	4,36	3,33
**Maximum**	1,7	3,13	9,49	6,55	2,24	3,06	1,72	1,53	7,67	5,48	5,68	6,36
**Mean**	1,04	1,492	5,385	2,147	1,606	1,388	0,7589	1,338	2,773	2,157	2,773	2,564
**Std. Deviation**	0,3545	0,8222	5,805	1,538	0,5538	0,8442	0,5148	0,209	2,058	1,688	1,601	1,826
**Std. Error**	0,1069	0,26	4,105	0,3625	0,2477	0,2985	0,1716	0,1045	0,4721	0,6381	0,6051	0,5064
**Lower 95% CI of mean**	0,8018	0,9039	-46,77	1,382	0,9184	0,6817	0,3632	1,005	1,781	0,5958	1,292	1,461
**Upper 95% CI of mean**	1,278	2,08	57,54	2,911	2,294	2,093	1,155	1,67	3,765	3,719	4,253	3,667
**Sum**	11,44	14,92	10,77	38,64	8,03	11,1	6,83	5,35	52,68	15,1	19,41	33,33

Ceramide

	**Controls healthy**	**Controls Bronchitis**	**Diffuse development. disorder**	**Growth abn. + defic. alveolar.**	**Immuno-intact host**	**Immuno-compromised host**	**Chronic tachypnea of infancy**	**Reactive lymphoid lesions**	**Related to alv. surf. region**	**Related to lung vessels/heart**	**Related to systemic disease**	**Unclear RDS in mature neonate**
**Number of values**	11	10	2	18	5	8	9	4	19	7	7	13
**Minimum**	0,06	0,11	0,83	0,05	0,11	0,13	0,06	0,09	0,14	0,05	0,1	0,09
**25% Percentile**	0,08	0,21		0,205	0,135	0,13	0,085	0,12	0,25	0,11	0,19	0,51
**Median**	0,15	0,38	0,96	0,525	0,21	0,52	0,18	0,15	0,86	0,33	0,4	0,78

**75% Percentile**	0,24	0,66		1,135	0,385	1,5	0,275	0,21	3,4	0,65	1,14	1,165
**Maximum**	0,37	3,2	1,09	2,06	0,52	2,6	2,05	0,27	8,4	2,48	2,25	1,73
**Mean**	0,1718	0,656	0,96	0,6656	0,25	0,8788	0,3744	0,165	1,847	0,5929	0,7786	0,8485
**Std. Deviation**	0,09325	0,9123	0,1838	0,5494	0,1598	0,9401	0,6334	0,0755	2,367	0,8562	0,7556	0,4759
**Std. Error**	0,02812	0,2885	0,13	0,1295	0,07148	0,3324	0,2111	0,03775	0,543	0,3236	0,2856	0,132
**Lower 95% CI of mean**	0,1092	0,003391	-0,6918	0,3923	0,05153	0,09282	-0,1124	0,04486	0,706	-0,199	0,07972	0,5609
**Upper 95% CI of mean**	0,2345	1,309	2,612	0,9388	0,4485	1,665	0,8613	0,2851	2,988	1,385	1,477	1,136
**Sum**	1,89	6,56	1,92	11,98	1,25	7,03	3,37	0,66	35,09	4,15	5,45	11,03

<u>Hexosylceramide</u>

	Controls healthy	Controls Bronchitis	Diffuse development. disorder	Growth abn. + defic. alveolar.	Immuno-intact host	Immuno-compromised host	Chronic tachypnea of infancy	Reactive lymphoid lesions	Related to alv. surf. region	Related to lung vessels/heart	Related to systemic disease	Unclear RDS in mature neonate
**Number of values**	11	10	2	18	5	8	9	4	19	7	7	13
**Minimum**	0,011	0,017	0,101	0,008	0,028	0,016	0,01	0,014	0	0,009	0,016	0,014
**25% Percentile**	0,012	0,0235		0,029	0,0335	0,0225	0,012	0,017	0,028	0,017	0,026	0,052
**Median**	0,018	0,0435	0,1255	0,059	0,043	0,0685	0,021	0,0255	0,099	0,036	0,075	0,079
**75% Percentile**	0,022	0,103		0,125	0,053	0,1865	0,0305	0,033	0,372	0,061	0,162	0,1135
**Maximum**	0,055	0,409	0,15	0,185	0,054	0,366	0,15	0,035	0,836	0,235	0,258	0,147
**Mean**	0,02073	0,0852	0,1255	0,07594	0,0432	0,1171	0,03422	0,025	0,2183	0,06014	0,1023	0,08254
**Std. Deviation**	0,01255	0,1187	0,03465	0,05395	0,01052	0,1238	0,04414	0,009695	0,2709	0,07897	0,08442	0,03759
**Std. Error**	0,003785	0,03754	0,0245	0,01272	0,004705	0,04377	0,01471	0,004848	0,06216	0,02985	0,03191	0,01043
**Lower 95% CI of mean**	0,01229	0,0002749	-0,1858	0,04911	0,03014	0,01363	0,0002902	0,009572	0,08773	-0,0129	0,02421	0,05982
**Upper 95% CI of mean**	0,02916	0,1701	0,4368	0,1028	0,05626	0,2206	0,06815	0,04043	0,3489	0,1332	0,1804	0,1053
**Sum**	0,228	0,852	0,251	1,367	0,216	0,937	0,308	0,1	4,148	0,421	0,716	1,073

SM 32:1

	Controls healthy	Controls Bronchitis	Diffuse development. disorder	Growth abn. + defic. alveolar.	Immuno-intact host	Immuno-compromised host	Chronic tachypnea of infancy	Reactive lymphoid lesions	Related to alv. surf. region	Related to lung vessels/heart	Related to systemic disease	Unclear RDS in mature neonate
**Number of values**	11	10	2	18	5	8	9	4	21	7	7	13
**Minimum**	0	0,49	0	0	0,43	0,63	0,38	0	0	0	0,58	0
**25% Percentile**	0	0,965		0,35	0,565	1,68	0,8	0	0,42	0	0,83	0,725
**Median**	1,27	1,905	1,135	1,52	0,94	2	1,85	0,775	1,2	0,84	1,14	1,24
**75% Percentile**	2,98	2,455		2,305	2,375	2,37	2,215	2,875	2,17	1,28	2,12	1,73
**Maximum**	5,18	3,04	2,27	3,38	2,81	3,52	2,27	4,2	3,85	1,72	2,67	3,73
**Mean**	1,73	1,779	1,135	1,454	1,364	2,031	1,584	1,438	1,427	0,8143	1,39	1,362
**Std. Deviation**	1,732	0,8255	1,605	1,055	0,9894	0,8215	0,7369	1,981	1,128	0,6516	0,7447	0,9165
**Std. Error**	0,5222	0,2611	1,135	0,2487	0,4425	0,2904	0,2456	0,9907	0,2462	0,2463	0,2815	0,2542
**Lower 95% CI of mean**	0,5665	1,188	-13,29	0,9291	0,1355	1,344	1,018	-1,715	0,9132	0,2116	0,7012	0,8077
**Upper 95% CI of mean**	2,893	2,37	15,56	1,979	2,593	2,718	2,151	4,59	1,94	1,417	2,079	1,915
**Sum**	19,03	17,79	2,27	26,17	6,82	16,25	14,26	5,75	29,96	5,7	9,73	17,7

SM 33:1

	Controls healthy	Controls Bronchitis	Diffuse development. disorder	Growth abn. + defic. alveolar.	Immuno-intact host	Immuno-compromised host	Chronic tachypnea of infancy	Reactive lymphoid lesions	Related to alv. surf. region	Related to lung vessels/heart	Related to systemic disease	Unclear RDS in mature neonate
**Number of values**	11	10	2	18	5	8	9	4	21	7	7	13
**Minimum**	0	0	0	0	0,95	0,34	0	0	0	0	0,52	0
**25% Percentile**	0	0,31		0	1,07	0,45	0	0,83	0,16	0	0,58	0,5
**Median**	0,96	0,905	0,255	0,54	2,04	0,78	0,78	1,81	0,83	0,34	0,93	0,61
**75% Percentile**	2,11	1,425		1,01	2,145	1,165	1,6	2,15	1,92	0,74	1,48	1,16
**Maximum**	5,1	1,71	0,51	1,57	2,17	1,64	2,62	2,34	4,01	0,84	2,11	1,64
**Mean**	1,375	0,891	0,255	0,5744	1,694	0,8463	0,9022	1,49	1,089	0,38	1,084	0,7477
**Std. Deviation**	1,497	0,5695	0,3606	0,5212	0,5778	0,4653	0,9205	1,032	1,031	0,3885	0,5578	0,5217
**Std. Error**	0,4513	0,1801	0,255	0,1228	0,2584	0,1645	0,3068	0,5158	0,2249	0,1468	0,2108	0,1447
**Lower 95% CI of mean**	0,3698	0,4836	-2,985	0,3153	0,9766	0,4572	0,1947	-0,1515	0,6199	0,02069	0,5684	0,4325
**Upper 95% CI of mean**	2,381	1,298	3,495	0,8336	2,411	1,235	1,61	3,131	1,558	0,7393	1,6	1,063
**Sum**	15,13	8,91	0,51	10,34	8,47	6,77	8,12	5,96	22,87	2,66	7,59	9,72

SM 34:2

	Controls healthy	Controls Bronchitis	Diffuse development. disorder	Growth abn. + defic. alveolar.	Immuno-intact host	Immuno-compromised host	Chronic tachypnea of infancy	Reactive lymphoid lesions	Related to alv. surf. region	Related to lung vessels/heart	Related to systemic disease	Unclear RDS in mature neonate
**Number of values**	11	10	2	18	5	8	9	4	21	7	7	13
**Minimum**	0	0	2,27	0	1,51	0,71	0	0	0	0	0,95	0
**25% Percentile**	0	0,39		0,42	1,8	1,015	0,455	0	0	0,34	0,96	0,8
**Median**	1,61	1,395	2,42	1,555	2,73	1,42	1,41	1,52	1,53	1,45	1,8	1,96
**75% Percentile**	3,77	2,335		2,35	3,535	2,665	1,605	3,315	2,15	3,35	3,18	2,67
**Maximum**	4,98	2,99	2,57	4,7	3,77	3,42	4,22	3,59	3,82	3,56	3,22	3,18
**Mean**	2,145	1,394	2,42	1,551	2,68	1,791	1,369	1,658	1,373	1,767	1,944	1,755
**Std. Deviation**	1,876	0,9974	0,2121	1,246	0,9072	1,001	1,238	1,927	1,136	1,381	0,9395	1,077
**Std. Error**	0,5656	0,3154	0,15	0,2938	0,4057	0,3539	0,4128	0,9635	0,2479	0,5219	0,3551	0,2988
**Lower 95% CI of mean**	0,8843	0,6805	0,5141	0,9313	1,554	0,9544	0,4169	-1,409	0,8558	0,4901	1,075	1,104
**Upper 95% CI of mean**	3,405	2,108	4,326	2,171	3,806	2,628	2,321	4,724	1,89	3,044	2,813	2,406
**Sum**	23,59	13,94	4,84	27,92	13,4	14,33	12,32	6,63	28,83	12,37	13,61	22,82

SM 34:1

	Controls healthy	Controls Bronchitis	Diffuse development. disorder	Growth abn. + defic. alveolar.	Immuno-intact host	Immuno-compromised host	Chronic tachypnea of infancy	Reactive lymphoid lesions	Related to alv. surf. region	Related to lung vessels/heart	Related to systemic disease	Unclear RDS in mature neonate
**Number of values**	11	10	2	18	5	8	9	4	21	7	7	13
**Minimum**	24,02	27,72	30,8	0	30,57	29,07	22,2	32,07	12,86	32,64	26,03	30,67
**25% Percentile**	30,35	33,75		29,31	34,36	31,25	27,15	37,43	29,18	33,76	27,09	32,18
**Median**	32,14	39,12	34,2	32,6	39,38	34,13	34,85	44,83	35,32	43,27	33,14	34,23
**75% Percentile**	38,25	45,59		40,19	47,5	42,51	37,77	51,55	41,76	46,41	39,26	39,58
**Maximum**	48,42	47,7	37,59	51,75	50,15	50,88	41,48	56,22	50,07	49,62	40,09	42,37
**Mean**	34,13	39,19	34,2	33,17	40,62	36,96	32,58	44,49	35,62	41,77	33,05	35,51
**Std. Deviation**	7,163	6,259	4,801	10,45	7,37	7,925	6,369	10,01	9,426	6,463	5,688	4,075
**Std. Error**	2,16	1,979	3,395	2,464	3,296	2,802	2,123	5,004	2,057	2,443	2,15	1,13
**Lower 95% CI of mean**	29,32	34,71	-8,943	27,98	31,47	30,34	27,69	28,56	31,33	35,79	27,79	33,05
**Upper 95% CI of mean**	38,94	43,67	77,33	38,37	49,77	43,59	37,48	60,41	39,91	47,74	38,31	37,97
**Sum**	375,4	391,9	68,39	597,1	203,1	295,7	293,3	177,9	747,9	292,4	231,4	461,6

SM 34:0

	Controls healthy	Controls Bronchitis	Diffuse development. disorder	Growth abn. + defic. alveolar.	Immuno-intact host	Immuno-compromised host	Chronic tachypnea of infancy	Reactive lymphoid lesions	Related to alv. surf. region	Related to lung vessels/heart	Related to systemic disease	Unclear RDS in mature neonate
**Number of values**	11	10	2	18	6	8	9	4	21	7	7	13

**Minimum**	0	0,42	1,66	0	0,33	0,99	0	0,6	0	0	1,44	0,61
**25% Percentile**	0	1,26		1,355	0,46	2,995	0	1,205	1,005	0,39	2,38	0,985
**Median**	0,72	2,455	2,285	3,245	1,3	3,595	0	1,935	2,79	1,32	2,55	1,56
**75% Percentile**	2,31	3,095		5,145	3,27	4,33	0,335	2,21	3,745	1,47	3,06	3,775
**Maximum**	6,66	3,7	2,91	11,05	3,47	5,26	0,72	2,36	8,41	26,88	5,55	4,84
**Mean**	1,59	2,265	2,285	3,856	1,677	3,511	0,1544	1,708	2,799	4,591	2,857	2,311
**Std. Deviation**	2,032	1,014	0,8839	3,285	1,364	1,277	0,3067	0,7718	2,184	9,843	1,283	1,554
**Std. Error**	0,6126	0,3208	0,625	0,7743	0,557	0,4515	0,1022	0,3859	0,4766	3,72	0,485	0,4309
**Lower 95% CI of mean**	0,225	1,539	-5,656	2,222	0,2449	2,444	-0,08132	0,4794	1,805	-4,512	1,67	1,372
**Upper 95% CI of mean**	2,955	2,991	10,23	5,489	3,108	4,579	0,3902	2,936	3,793	13,7	4,044	3,25
**Sum**	17,49	22,65	4,57	69,4	10,06	28,09	1,39	6,83	58,78	32,14	20	30,04

<u>SM36:2</u>

	Controls healthy	Controls Bronchitis	Diffuse development. disorder	Growth abn. + defic. alveolar.	Immuno-intact host	Immuno-compromised host	Chronic tachypnea of infancy	Reactive lymphoid lesions	Related to alv. surf. region	Related to lung vessels/heart	Related to systemic disease	Unclear RDS in mature neonate
**Number of values**	11	10	2	18	5	8	9	4	21	7	7	13
**Minimum**	0	0	0	0	0	0	0	0	0	0	0,31	0
**25% Percentile**	0	0		0	0,265	0	0,08	0	0	0,14	0,32	0,225
**Median**	0	0,33	0,745	0,115	1,23	0,305	0,58	0	0,46	0,47	0,5	0,94
**75% Percentile**	0,38	0,985		0,65	1,545	0,58	1,265	0,725	0,72	1,69	1,4	1,72
**Maximum**	1,8	4,26	1,49	2,12	1,85	1,57	3,18	1,45	2,49	7,84	1,93	2,42
**Mean**	0,2664	0,743	0,745	0,4311	0,97	0,4175	0,8489	0,3625	0,5271	1,566	0,7857	1,021
**Std. Deviation**	0,5627	1,293	1,054	0,6331	0,7158	0,5254	1,014	0,725	0,6201	2,821	0,6314	0,8051
**Std. Error**	0,1697	0,409	0,745	0,1492	0,3201	0,1858	0,3378	0,3625	0,1353	1,066	0,2386	0,2233
**Lower 95% CI of mean**	-0,1117	-0,1822	-8,721	0,1163	0,08123	-0,02178	0,06982	-0,7911	0,2449	-1,044	0,2018	0,5343
**Upper 95% CI of mean**	0,6444	1,668	10,21	0,7459	1,859	0,8568	1,628	1,516	0,8094	4,175	1,37	1,507
**Sum**	2,93	7,43	1,49	7,76	4,85	3,34	7,64	1,45	11,07	10,96	5,5	13,27

<u>SM 36:1</u>

	Controls healthy	Controls Bronchitis	Diffuse development. disorder	Growth abn. + defic. alveolar.	Immuno-intact host	Immuno-compromised host	Chronic tachypnea of infancy	Reactive lymphoid lesions	Related to alv. surf. region	Related to lung vessels/heart	Related to systemic disease	Unclear RDS in mature neonate
**Number of values**	11	10	2	18	5	8	9	4	21	7	7	13
**Minimum**	0	0	2,49	0	0,95	0,68	0	0	0	0	1,23	1,44
**25% Percentile**	1,76	1,895		1,35	1,62	2,14	2,51	0	2,945	1,1	2,85	3,465
**Median**	2,55	3,165	4,055	4,835	2,82	3,35	2,95	1,255	4,45	2,06	4,41	4,34
**75% Percentile**	3,74	5,04		5,655	4,965	4,975	5,735	3,2	5,525	2,37	5,07	5,495
**Maximum**	6,38	5,93	5,62	12,65	5,31	5,46	9,03	3,89	8,21	7,43	5,64	6,56
**Mean**	2,802	3,317	4,055	4,425	3,198	3,384	3,781	1,6	4,022	2,407	3,811	4,38
**Std. Deviation**	2,045	1,862	2,213	3,525	1,768	1,686	2,636	1,932	2,352	2,364	1,531	1,381
**Std. Error**	0,6167	0,5887	1,565	0,8307	0,7907	0,596	0,8785	0,9658	0,5133	0,8935	0,5788	0,3831
**Lower 95% CI of mean**	1,428	1,985	-15,83	2,672	1,003	1,974	1,755	-1,473	2,951	0,2207	2,395	3,545
**Upper 95% CI of mean**	4,176	4,649	23,94	6,178	5,393	4,793	5,807	4,673	5,093	4,594	5,228	5,215
**Sum**	30,82	33,17	8,11	79,65	15,99	27,07	34,03	6,4	84,46	16,85	26,68	56,94

<u>SM 36:0</u>

	Controls healthy	Controls Bronchitis	Diffuse development. disorder	Growth abn. + defic. alveolar.	Immuno-intact host	Immuno-compromised host	Chronic tachypnea of infancy	Reactive lymphoid lesions	Related to alv. surf. region	Related to lung vessels/heart	Related to systemic disease	Unclear RDS in mature neonate
**Number of values**	11	10	2	18	5	8	9	4	21	7	7	13
**Minimum**	0	0	0	0	0	0	0	0	0	0	0	0
**25% Percentile**	0	0		0	0	0	0	0	0	0	0	0
**Median**	0	0	0	0	0	0	0	0	0,47	0	0	0
**75% Percentile**	1,19	1,085		3,915	0,995	0	0,915	0	2,715	2,51	1,73	2,06
**Maximum**	10,13	10,3	0	22,39	1,99	1,28	7,87	0	10,48	12,07	2,6	3,19
**Mean**	1,553	1,247	0	3,344	0,398	0,16	1,078	0	2,211	2,15	0,6771	0,8646
**Std. Deviation**	3,327	3,223	0	6,93	0,89	0,4525	2,618	0	3,557	4,469	1,058	1,243
**Std. Error**	1,003	1,019	0	1,633	0,398	0,16	0,8727	0	0,7762	1,689	0,3997	0,3447
**Lower 95% CI of mean**	-0,6824	-1,058	0	-0,1018	-0,707	-0,2183	-0,9346	0	0,5918	-1,983	-0,301	0,1137
**Upper 95% CI of mean**	3,788	3,552	0	6,791	1,503	0,5383	3,09	0	3,83	6,283	1,655	1,616
**Sum**	17,08	12,47	0	60,2	1,99	1,28	9,7	0	46,43	15,05	4,74	11,24

<u>SM 38:2</u>

	Controls healthy	Controls Bronchitis	Diffuse development. disorder	Growth abn. + defic. alveolar.	Immuno-intact host	Immuno-compromised host	Chronic tachypnea of infancy	Reactive lymphoid lesions	Related to alv. surf. region	Related to lung vessels/heart	Related to systemic disease	Unclear RDS in mature neonate
**Number of values**	11	10	2	18	5	8	9	4	21	7	7	13
**Minimum**	0	0	0	0	0,88	0	0	0	0	0	0	0
**25% Percentile**	0	0		0	0,945	0	0	0,355	0	0	0	0
**Median**	1,13	0,435	0	0	1,38	0	0	0,935	0,17	0	0,33	0
**75% Percentile**	2,46	1,475		0,35	2,615	0,355	0,885	4,015	0,645	2,07	1,22	0,455

	Controls healthy	Controls Bronchitis	Diffuse development. disorder	Growth abn. + defic. alveolar.	Immuno-intact host	Immuno-compromised host	Chronic tachypnea of infancy	Reactive lymphoid lesions	Related to alv. surf. region	Related to lung vessels/heart	Related to systemic disease	Unclear RDS in mature neonate
**Maximum**	6,62	3,59	0	1,92	3,36	1,34	5,18	6,87	2,24	3,52	2,88	1,25
**Mean**	1,606	0,819	0	0,2278	1,7	0,2563	0,8233	2,185	0,4567	0,7986	0,7557	0,2369
**Std. Deviation**	1,935	1,173	0	0,4959	1,004	0,4664	1,682	3,16	0,6873	1,427	1,025	0,41
**Std. Error**	0,5834	0,3711	0	0,1169	0,4492	0,1649	0,5607	1,58	0,15	0,5392	0,3874	0,1137
**Lower 95% CI of mean**	0,3066	-0,02042	0	-0,01881	0,4528	-0,1337	-0,4698	-2,843	0,1438	-0,5208	-0,1922	-0,01085
**Upper 95% CI of mean**	2,906	1,658	0	0,4744	2,947	0,6462	2,116	7,213	0,7695	2,118	1,704	0,4847
**Sum**	17,67	8,19	0	4,1	8,5	2,05	7,41	8,74	9,59	5,59	5,29	3,08

SM 38:1

	Controls healthy	Controls Bronchitis	Diffuse development. disorder	Growth abn. + defic. alveolar.	Immuno-intact host	Immuno-compromised host	Chronic tachypnea of infancy	Reactive lymphoid lesions	Related to alv. surf. region	Related to lung vessels/heart	Related to systemic disease	Unclear RDS in mature neonate
**Number of values**	11	10	2	18	5	8	9	4	21	7	7	13
**Minimum**	0	0	4,73	0	0	0	0	0	0	0	0,86	0
**25% Percentile**	0	0,92		1,06	0	0,36	0	0	1,835	0	1,39	1,26
**Median**	0	2,585	5,51	3,975	3,96	2,205	5,2	0	3,24	0	2,19	3,47
**75% Percentile**	0	4,89		7,27	8,05	3,075	10,35	2	6,085	3,64	5,21	5,375
**Maximum**	4,36	7,93	6,29	15,95	11,96	8,43	13,42	4	13,64	6,12	6,59	8,81
**Mean**	0,6109	3,04	5,51	4,832	4,012	2,464	5,409	1	4,221	1,871	2,901	3,558
**Std. Deviation**	1,431	2,361	1,103	4,633	4,883	2,724	5,051	2	3,356	2,495	2,163	2,67
**Std. Error**	0,4314	0,7468	0,78	1,092	2,184	0,9631	1,684	1	0,7324	0,9429	0,8176	0,7406
**Lower 95% CI of mean**	-0,3504	1,351	-4,401	2,527	-2,051	0,1864	1,527	-2,182	2,694	-0,4359	0,9007	1,945
**Upper 95% CI of mean**	1,572	4,729	15,42	7,136	10,08	4,741	9,291	4,182	5,749	4,179	4,902	5,172
**Sum**	6,72	30,4	11,02	86,97	20,06	19,71	48,68	4	88,65	13,1	20,31	46,26

SM 40:2

	Controls healthy	Controls Bronchitis	Diffuse development. disorder	Growth abn. + defic. alveolar.	Immuno-intact host	Immuno-compromised host	Chronic tachypnea of infancy	Reactive lymphoid lesions	Related to alv. surf. region	Related to lung vessels/heart	Related to systemic disease	Unclear RDS in mature neonate
**Number of values**	11	10	2	18	5	8	9	4	21	7	7	13
**Minimum**	0	0	1,69	0	0	0	0	0	0	0	0	0
**25% Percentile**	0	0		0	0,35	0	0	0	0,665	0	0	0,35
**Median**	1,49	0,885	2,465	0,815	1,2	0,445	0	0,705	1,81	0,19	2,28	2,02
**75% Percentile**	3,64	6,85		3,155	2,46	1,19	4,055	4,58	2,74	4,65	3,78	3,455
**Maximum**	7,73	9,75	3,24	9,25	3,12	2,49	5,93	7,75	16,16	4,68	5,75	3,92
**Mean**	2,092	2,723	2,465	2,071	1,364	0,72	1,632	2,29	2,554	1,533	2,197	2,012
**Std. Deviation**	2,572	3,73	1,096	2,906	1,183	0,9011	2,367	3,7	3,51	2,182	2,163	1,473
**Std. Error**	0,7756	1,18	0,775	0,6849	0,5292	0,3186	0,7889	1,85	0,7658	0,8249	0,8177	0,4086
**Lower 95% CI of mean**	0,3637	0,05445	-7,382	0,6255	-0,1054	-0,03338	-0,1869	-3,598	0,9568	-0,4856	0,1963	1,121
**Upper 95% CI of mean**	3,82	5,392	12,31	3,516	2,833	1,473	3,451	8,178	4,152	3,551	4,198	2,902
**Sum**	23,01	27,23	4,93	37,27	6,82	5,76	14,69	9,16	53,64	10,73	15,38	26,15

SM 40:1

	Controls healthy	Controls Bronchitis	Diffuse development. disorder	Growth abn. + defic. alveolar.	Immuno-intact host	Immuno-compromised host	Chronic tachypnea of infancy	Reactive lymphoid lesions	Related to alv. surf. region	Related to lung vessels/heart	Related to systemic disease	Unclear RDS in mature neonate
**Number of values**	11	10	2	18	5	8	9	4	21	7	7	13
**Minimum**	0	0	7,7	0	0,37	0	0	0	2	0	6,94	4,45
**25% Percentile**	1,41	2,195		3,46	0,475	2,065	2,395	0,97	4,65	0	6,95	5,34
**Median**	8,26	5,685	8,82	6,045	3,45	5,995	5,23	2,43	6,69	3,54	8,22	6,25
**75% Percentile**	9,89	7,47		8,73	6,025	6,705	10,45	4,5	8,495	4,67	9,57	7,605
**Maximum**	22,75	9,88	9,94	13,47	6,28	7,32	11,42	6,08	25,96	6,01	11,62	9,3
**Mean**	7,403	5,057	8,82	6,317	3,29	4,606	5,877	2,735	7,642	2,621	8,433	6,492
**Std. Deviation**	6,412	3,121	1,584	3,744	2,783	2,994	4,263	2,539	5,165	2,563	1,691	1,424
**Std. Error**	1,933	0,9871	1,12	0,8824	1,245	1,059	1,421	1,269	1,127	0,9687	0,6392	0,3948
**Lower 95% CI of mean**	3,095	2,824	-5,411	4,456	-0,1659	2,103	2,6	-1,305	5,291	0,251	6,869	5,631
**Upper 95% CI of mean**	11,71	7,29	23,05	8,179	6,746	7,11	9,153	6,775	9,993	4,992	9,997	7,352
**Sum**	81,43	50,57	17,64	113,7	16,45	36,85	52,89	10,94	160,5	18,35	59,03	84,39

SM 41:2

	Controls healthy	Controls Bronchitis	Diffuse development. disorder	Growth abn. + defic. alveolar.	Immuno-intact host	Immuno-compromised host	Chronic tachypnea of infancy	Reactive lymphoid lesions	Related to alv. surf. region	Related to lung vessels/heart	Related to systemic disease	Unclear RDS in mature neonate
**Number of values**	11	10	2	18	5	8	9	4	21	7	7	13
**Minimum**	0	0	0	0	0	0	0	0	0	0,39	0,3	0
**25% Percentile**	0	0,125		0	0	0,52	0	0	0	0,48	0,52	0
**Median**	0	0,755	0,62	0,515	0	0,71	0	0,53	0,62	0,8	1,08	0,73
**75% Percentile**	1,18	1,685		1,035	1,265	0,925	0,945	1,12	1,425	1,87	1,46	1,305
**Maximum**	1,69	4,34	1,24	3,03	1,83	1,01	1,41	1,18	9,28	2,57	1,62	3,32
**Mean**	0,4045	1,1	0,62	0,6639	0,506	0,665	0,4	0,56	1,205	1,171	1	0,8569
**Std. Deviation**	0,7032	1,313	0,8768	0,8005	0,7998	0,3277	0,5525	0,6485	2,002	0,8026	0,5259	0,9608
**Std. Error**	0,212	0,4153	0,62	0,1887	0,3577	0,1159	0,1842	0,3242	0,437	0,3034	0,1988	0,2665

	Controls healthy	Controls Bronchitis	Diffuse development. disorder	Growth abn. + defic. alveolar.	Immuno-intact host	Immuno-compromised host	Chronic tachypnea of infancy	Reactive lymphoid lesions	Related to alv. surf. region	Related to lung vessels/heart	Related to systemic disease	Unclear RDS in mature neonate
**Lower 95% CI of mean**	-0,06786	0,1605	-7,258	0,2658	-0,4871	0,391	-0,0247	-0,4719	0,2933	0,4291	0,5137	0,2763
**Upper 95% CI of mean**	0,8769	2,04	8,498	1,062	1,499	0,939	0,8247	1,592	2,116	1,914	1,486	1,438
**Sum**	4,45	11	1,24	11,95	2,53	5,32	3,6	2,24	25,3	8,2	7	11,14

<u>SM 41:1</u>

	Controls healthy	Controls Bronchitis	Diffuse development. disorder	Growth abn. + defic. alveolar.	Immuno-intact host	Immuno-compromised host	Chronic tachypnea of infancy	Reactive lymphoid lesions	Related to alv. surf. region	Related to lung vessels/heart	Related to systemic disease	Unclear RDS in mature neonate
**Number of values**	11	10	2	18	5	8	9	4	21	7	7	13
**Minimum**	0,68	0,96	1,39	0	1,36	1,18	0,99	0	0,63	0	1,46	1,28
**25% Percentile**	1,36	1,695		1,065	1,365	1,32	1,535	1,06	1,905	0	1,55	1,705
**Median**	2,45	2,255	2,5	1,765	1,41	1,655	2,07	2,72	2,41	2,33	2,15	2,15
**75% Percentile**	3,78	3,01		2,415	2,155	1,975	3,525	3,635	3,255	2,58	3,3	2,47
**Maximum**	6,9	3,48	3,61	3,86	2,33	2,28	5,67	3,95	3,8	2,71	3,33	2,96
**Mean**	2,8	2,288	2,5	1,729	1,69	1,67	2,562	2,348	2,453	1,654	2,349	2,101
**Std. Deviation**	1,895	0,774	1,57	0,9725	0,4425	0,3927	1,548	1,739	0,8876	1,187	0,8284	0,4947
**Std. Error**	0,5714	0,2448	1,11	0,2292	0,1979	0,1388	0,5159	0,8697	0,1937	0,4485	0,3131	0,1372
**Lower 95% CI of mean**	1,527	1,734	-11,6	1,246	1,141	1,342	1,372	-0,4202	2,049	0,5567	1,582	1,802
**Upper 95% CI of mean**	4,073	2,842	16,6	2,213	2,24	1,998	3,752	5,115	2,857	2,752	3,115	2,4
**Sum**	30,8	22,88	5	31,13	8,45	13,36	23,06	9,39	51,52	11,58	16,44	27,31

<u>SM 42:3</u>

	Controls healthy	Controls Bronchitis	Diffuse development. disorder	Growth abn. + defic. alveolar.	Immuno-intact host	Immuno-compromised host	Chronic tachypnea of infancy	Reactive lymphoid lesions	Related to alv. surf. region	Related to lung vessels/heart	Related to systemic disease	Unclear RDS in mature neonate
**Number of values**	11	10	2	18	5	8	9	4	21	7	7	13
**Minimum**	0	0,68	1,38	0	3,78	2,21	0,54	1,12	0	0	2,22	0,77
**25% Percentile**	1,76	1,485		1,57	3,9	2,86	0,94	2,57	3,29	0	2,27	3,315
**Median**	2,68	2,94	2,425	3,465	4,45	3,575	2,47	6,385	3,88	2,16	3,42	4,17
**75% Percentile**	4,69	4,54		5,025	8,49	4,145	7,16	11,33	4,805	2,67	4,99	4,62
**Maximum**	6,58	5,51	3,47	7,3	11,13	7,45	11,61	13,9	6,42	3,85	5,74	6,02
**Mean**	2,963	3,017	2,425	3,218	5,846	3,853	4,02	6,948	3,715	1,829	3,803	3,944
**Std. Deviation**	1,905	1,541	1,478	2,093	3,061	1,603	4,015	5,601	1,568	1,398	1,423	1,288
**Std. Error**	0,5744	0,4874	1,045	0,4932	1,369	0,5666	1,338	2,801	0,3422	0,5283	0,5379	0,3573
**Lower 95% CI of mean**	1,683	1,914	-10,85	2,177	2,046	2,513	0,9341	-1,965	3,001	0,5358	2,487	3,165
**Upper 95% CI of mean**	4,243	4,12	15,7	4,258	9,646	5,192	7,106	15,86	4,429	3,121	5,119	4,722
**Sum**	32,59	30,17	4,85	57,92	29,23	30,82	36,18	27,79	78,01	12,8	26,62	51,27

<u>SM 42:2</u>

	Controls healthy	Controls Bronchitis	Diffuse development. disorder	Growth abn. + defic. alveolar.	Immuno-intact host	Immuno-compromised host	Chronic tachypnea of infancy	Reactive lymphoid lesions	Related to alv. surf. region	Related to lung vessels/heart	Related to systemic disease	Unclear RDS in mature neonate
**Number of values**	11	10	2	18	5	8	9	4	21	7	7	13
**Minimum**	10,45	13,12	18,81	0	11,31	18,46	14,01	10,33	7,49	16,05	15,86	14,29
**25% Percentile**	14,2	16,22		16,23	13,27	22,08	15,23	11,67	16,51	17,48	17,53	18,53
**Median**	17,1	17,75	19,42	19,82	16,39	24,99	17,7	15,52	18,95	19,16	20,89	19,67
**75% Percentile**	18,3	24,58		23,86	18,52	28,56	24,33	18,41	21	25,59	25,98	23,79
**Maximum**	23,56	27,77	20,03	29,01	19,42	34,11	25,87	18,77	26,69	25,68	27,29	27,41
**Mean**	16,94	19,54	19,42	19,53	15,99	25,48	19,02	15,04	18,98	20,66	21,31	20,84
**Std. Deviation**	3,921	4,834	0,8627	6,556	3,043	5,016	4,634	4,052	4,206	3,788	4,221	3,63
**Std. Error**	1,182	1,529	0,61	1,545	1,361	1,773	1,545	2,026	0,9179	1,432	1,596	1,007
**Lower 95% CI of mean**	14,3	16,08	11,67	16,27	12,21	21,28	15,46	8,587	17,07	17,15	17,41	18,65
**Upper 95% CI of mean**	19,57	23	27,17	22,79	19,77	29,67	22,58	21,48	20,9	24,16	25,21	23,03
**Sum**	186,3	195,4	38,84	351,5	79,95	203,8	171,2	60,14	398,6	144,6	149,2	270,9

<u>SM 42:1</u>

	Controls healthy	Controls Bronchitis	Diffuse development. disorder	Growth abn. + defic. alveolar.	Immuno-intact host	Immuno-compromised host	Chronic tachypnea of infancy	Reactive lymphoid lesions	Related to alv. surf. region	Related to lung vessels/heart	Related to systemic disease	Unclear RDS in mature neonate
**Number of values**	11	10	2	18	5	8	9	4	21	7	7	13
**Minimum**	4,92	5,64	7,57	0	2,58	6,08	7,53	5,22	0	7,24	6,65	6,18
**25% Percentile**	7,47	6,68		4,475	4,155	7,58	8,59	6,085	7,335	7,46	7,44	7,615
**Median**	9,21	9,105	8,07	7,68	6,42	8,355	9,51	7,485	9,28	10,82	10,02	8,59
**75% Percentile**	12,55	10,37		9,315	7,475	10,62	10,17	9,465	11,37	13,29	12,22	11,7
**Maximum**	17,29	13,09	8,57	13,91	8,01	11,29	14,53	10,91	22,67	15,71	14,57	15,87
**Mean**	10,23	8,871	8,07	7,434	5,936	8,809	9,758	7,775	9,54	10,83	10,09	9,7
**Std. Deviation**	3,682	2,292	0,7071	3,492	2,052	1,849	1,997	2,387	4,552	3,009	2,699	2,68
**Std. Error**	1,11	0,7249	0,5	0,823	0,9178	0,6539	0,6656	1,194	0,9934	1,137	1,02	0,7434
**Lower 95% CI of mean**	7,759	7,231	1,717	5,698	3,388	7,263	8,223	3,976	7,468	8,047	7,591	8,08
**Upper 95% CI of mean**	12,71	10,51	14,42	9,171	8,484	10,35	11,29	11,57	11,61	13,61	12,58	11,32
**Sum**	112,6	88,71	16,14	133,8	29,68	70,47	87,82	31,1	200,4	75,81	70,61	126,1

PC 30:1

	Controls healthy	Controls Bronchitis	Diffuse development. disorder	Growth abn.+ defic. alveolar.	Immuno-intact host	Immuno-compromised host	Chronic tachypnea of infancy	Reactive lymphoid lesions	Related to alv. surf. region	Related to lung vessels/heart	Related to systemic disease	Unclear RDS in mature neonate
**Number of values**	11	10	2	18	5	8	9	4	21	7	7	13
**Minimum**	0,2	0,16	0,14	0	0,35	0,31	0,34	0,4	0	0	0,29	0,13
**25% Percentile**	0,22	0,34		0,42	0,4	0,315	0,37	0,43	0,205	0,37	0,31	0,305
**Median**	0,34	0,435	0,335	0,69	0,48	0,505	0,5	0,59	0,34	0,46	0,48	0,51
**75% Percentile**	0,43	0,59		0,83	0,535	0,845	0,585	0,75	0,485	0,62	0,98	0,63
**Maximum**	0,58	0,69	0,53	1,6	0,59	1,12	0,96	0,78	1,12	0,84	1,43	0,7
**Mean**	0,3418	0,444	0,335	0,6967	0,47	0,595	0,5233	0,59	0,3905	0,4643	0,63	0,4692
**Std. Deviation**	0,1208	0,1594	0,2758	0,3733	0,08573	0,3249	0,1885	0,188	0,2976	0,2567	0,4261	0,1881
**Std. Error**	0,03643	0,0504	0,195	0,08798	0,03834	0,1149	0,06285	0,09399	0,06494	0,09702	0,1611	0,05216
**Lower 95% CI of mean**	0,2607	0,33	-2,143	0,511	0,3635	0,3234	0,3784	0,2909	0,255	0,2269	0,2359	0,3556
**Upper 95% CI of mean**	0,423	0,558	2,813	0,8823	0,5765	0,8666	0,6683	0,8891	0,5259	0,7017	1,024	0,5829
**Sum**	3,76	4,44	0,67	12,54	2,35	4,76	4,71	2,36	8,2	3,25	4,41	6,1

PC 30:0

	Controls healthy	Controls Bronchitis	Diffuse development. disorder	Growth abn.+ defic. alveolar.	Immuno-intact host	Immuno-compromised host	Chronic tachypnea of infancy	Reactive lymphoid lesions	Related to alv. surf. region	Related to lung vessels/heart	Related to systemic disease	Unclear RDS in mature neonate
**Number of values**	11	10	2	18	5	8	9	4	21	7	7	13
**Minimum**	5,11	5,95	7,14	3,09	9,03	5,69	9,38	8,63	0,33	1,41	5,78	3,44
**25% Percentile**	7,76	6,375		7,49	9,67	7,005	9,87	9,235	4,33	4,32	6,6	6,78
**Median**	8,37	11,22	8,13	9,995	10,43	8,6	11,09	9,875	8,36	9,19	6,95	7,55
**75% Percentile**	9,52	11,9		10,82	12,28	11,35	12,74	10,85	10,11	11,64	13,72	8,865
**Maximum**	10,77	12,38	9,12	12,07	12,68	12,75	13,89	11,79	13,08	12,79	14,86	11,57
**Mean**	8,225	9,87	8,13	9,207	10,86	9,044	11,35	10,04	7,299	8,239	9,19	7,799
**Std. Deviation**	1,771	2,6	1,4	2,229	1,429	2,558	1,604	1,305	3,678	4,26	3,715	2,044
**Std. Error**	0,5341	0,8223	0,99	0,5253	0,6391	0,9042	0,5347	0,6524	0,8027	1,61	1,404	0,5669
**Lower 95% CI of mean**	7,034	8,01	-4,449	8,099	9,09	6,906	10,12	7,966	5,624	4,299	5,754	6,564
**Upper 95% CI of mean**	9,415	11,73	20,71	10,32	12,64	11,18	12,59	12,12	8,973	12,18	12,63	9,034
**Sum**	90,47	98,7	16,26	165,7	54,32	72,35	102,2	40,17	153,3	57,67	64,33	101,4

PC O 32:1

	Controls healthy	Controls Bronchitis	Diffuse development. disorder	Growth abn.+ defic. alveolar.	Immuno-intact host	Immuno-compromised host	Chronic tachypnea of infancy	Reactive lymphoid lesions	Related to alv. surf. region	Related to lung vessels/heart	Related to systemic disease	Unclear RDS in mature neonate
**Number of values**	11	10	2	18	5	8	9	4	21	7	7	13
**Minimum**	0,37	0,53	0,78	0	0,46	0,38	0,36	0,58	0,19	0,3	0,56	0,33
**25% Percentile**	0,45	0,6		0,405	0,505	0,655	0,375	0,605	0,585	0,34	0,9	0,57
**Median**	0,54	0,67	0,82	0,495	0,83	0,855	0,47	0,7	1,06	0,6	1,17	0,68
**75% Percentile**	0,59	1,785		0,645	0,895	1,91	0,49	0,975	1,71	1,15	1,48	1,165
**Maximum**	0,75	13,19	0,86	2,06	0,91	13,98	0,67	1,18	3,93	1,28	1,52	2,17
**Mean**	0,5373	2,122	0,82	0,6017	0,726	2,65	0,4644	0,79	1,299	0,6914	1,149	0,8815
**Std. Deviation**	0,108	3,947	0,05657	0,4189	0,2062	4,622	0,09462	0,2722	0,9923	0,3805	0,3635	0,4929
**Std. Error**	0,03256	1,248	0,04	0,09872	0,09223	1,634	0,03154	0,1361	0,2165	0,1438	0,1374	0,1367
**Lower 95% CI of mean**	0,4647	-0,7016	0,3118	0,3934	0,4699	-1,214	0,3917	0,3569	0,8469	0,3396	0,8124	0,5837
**Upper 95% CI of mean**	0,6098	4,946	1,328	0,81	0,9821	6,514	0,5372	1,223	1,75	1,043	1,485	1,179
**Sum**	5,91	21,22	1,64	10,83	3,63	21,2	4,18	3,16	27,27	4,84	8,04	11,46

PC O 32:0

	Controls healthy	Controls Bronchitis	Diffuse development. disorder	Growth abn.+ defic. alveolar.	Immuno-intact host	Immuno-compromised host	Chronic tachypnea of infancy	Reactive lymphoid lesions	Related to alv. surf. region	Related to lung vessels/heart	Related to systemic disease	Unclear RDS in mature neonate
**Number of values**	11	10	2	18	5	8	9	4	21	7	7	13
**Minimum**	0,87	1,79	1,73	0,88	2,89	1,61	1,05	1,56	0,31	0,71	1,98	0,93
**25% Percentile**	1,73	2,615		1,08	2,895	1,895	1,365	1,97	1,575	1,07	2,18	1,08
**Median**	2,95	3,825	1,825	1,36	2,9	2,535	1,74	2,535	2,7	2,1	2,79	1,65
**75% Percentile**	3,04	4,79		1,935	3,485	3,68	2,265	2,905	3,45	3,44	3,82	1,865
**Maximum**	3,68	8,92	1,92	6,07	3,49	9,42	2,87	3,12	6,19	3,93	4,13	3,1
**Mean**	2,508	4,067	1,825	1,811	3,132	3,406	1,85	2,438	2,703	2,259	2,92	1,688
**Std. Deviation**	0,8314	1,988	0,1344	1,315	0,3223	2,566	0,5861	0,659	1,412	1,174	0,7984	0,6564
**Std. Error**	0,2507	0,6286	0,095	0,3099	0,1441	0,9072	0,1954	0,3295	0,3081	0,4438	0,3018	0,182
**Lower 95% CI of mean**	1,95	2,645	0,6179	1,157	2,732	1,261	1,399	1,389	2,06	1,173	2,182	1,291
**Upper 95% CI of mean**	3,067	5,489	3,032	2,464	3,532	5,552	2,301	3,486	3,346	3,344	3,658	2,084
**Sum**	27,59	40,67	3,65	32,59	15,66	27,25	16,65	9,75	56,76	15,81	20,44	21,94

PC 32:2

	Controls healthy	Controls Bronchitis	Diffuse development. disorder	Growth abn.+ defic. alveolar.	Immuno-intact host	Immuno-compromised host	Chronic tachypnea of infancy	Reactive lymphoid lesions	Related to alv. surf. region	Related to lung vessels/heart	Related to systemic disease	Unclear RDS in mature neonate
**Number of values**	11	10	2	18	5	8	9	4	21	7	7	13

**Minimum**	0,18	0,17	0,62	0	0,19	0,23	0,19	0,61	0,2	0,17	0,35	0,21
**25% Percentile**	0,24	0,25		0,415	0,31	0,5	0,35	0,625	0,425	0,4	0,69	0,31
**Median**	0,29	0,375	0,705	0,765	0,47	0,57	0,45	0,66	0,57	0,59	0,89	0,56
**75% Percentile**	0,55	0,55		0,94	0,555	0,76	0,555	0,685	0,765	0,74	1,14	0,69
**Maximum**	0,91	0,85	0,79	1,07	0,62	1,06	0,85	0,69	2,08	0,77	1,35	1,11
**Mean**	0,3873	0,41	0,705	0,6978	0,44	0,6188	0,4633	0,655	0,6871	0,5429	0,8786	0,5423
**Std. Deviation**	0,2195	0,2018	0,1202	0,2926	0,1568	0,2507	0,1857	0,03697	0,4602	0,2099	0,3287	0,2534
**Std. Error**	0,0662	0,06382	0,085	0,06897	0,07014	0,08865	0,06189	0,01848	0,1004	0,07933	0,1242	0,07028
**Lower 95% CI of mean**	0,2398	0,2656	-0,375	0,5523	0,2453	0,4091	0,3206	0,5962	0,4777	0,3487	0,5746	0,3892
**Upper 95% CI of mean**	0,5348	0,5544	1,785	0,8433	0,6347	0,8284	0,6061	0,7138	0,8966	0,737	1,183	0,6954
**Sum**	4,26	4,1	1,41	12,56	2,2	4,95	4,17	2,62	14,43	3,8	6,15	7,05

PC 32:1

	Controls healthy	Controls Bronchitis	Diffuse development. disorder	Growth abn.+ defic. alveolar.	Immuno-intact host	Immuno-compromised host	Chronic tachypnea of infancy	Reactive lymphoid lesions	Related to alv. surf. region	Related to lung vessels/heart	Related to systemic disease	Unclear RDS in mature neonate
**Number of values**	11	10	2	18	5	8	9	4	21	7	7	13
**Minimum**	6,3	4,09	9,82	1,57	8,07	4,5	5,4	7,77	1,07	3,12	7,49	5,09
**25% Percentile**	6,77	7,06		6,995	8,115	7,38	7,51	9	5,53	7,43	7,49	6,17
**Median**	8,06	8,7	10,51	8,385	8,35	9,175	8,44	10,78	8,36	9,8	10,13	7,55
**75% Percentile**	10,31	9,55		11,26	9,225	9,685	9,59	11,48	10,18	10,2	12,81	10,56
**Maximum**	13,86	10,69	11,2	12,18	9,48	10,4	10,1	11,62	16,19	11,85	12,86	11,76
**Mean**	8,795	8,274	10,51	8,583	8,606	8,423	8,326	10,24	7,984	8,717	10,15	8,065
**Std. Deviation**	2,54	1,89	0,9758	2,795	0,6017	1,925	1,47	1,751	3,834	2,812	2,285	2,295
**Std. Error**	0,7659	0,5977	0,69	0,6589	0,2691	0,6805	0,4899	0,8753	0,8366	1,063	0,8637	0,6364
**Lower 95% CI of mean**	7,088	6,922	1,743	7,193	7,859	6,813	7,196	7,452	6,239	6,117	8,039	6,678
**Upper 95% CI of mean**	10,5	9,626	19,28	9,973	9,353	10,03	9,455	13,02	9,729	11,32	12,27	9,451
**Sum**	96,74	82,74	21,02	154,5	43,03	67,38	74,93	40,95	167,7	61,02	71,07	104,8

PC 32:0

	Controls healthy	Controls Bronchitis	Diffuse development. disorder	Growth abn.+ defic. alveolar.	Immuno-intact host	Immuno-compromised host	Chronic tachypnea of infancy	Reactive lymphoid lesions	Related to alv. surf. region	Related to lung vessels/heart	Related to systemic disease	Unclear RDS in mature neonate
**Number of values**	11	10	2	18	5	8	9	4	21	7	7	14
**Minimum**	40,72	25,57	46,59	31,88	36,75	32,02	38,99	34,25	0,7	8,25	26,09	31,33
**25% Percentile**	43,8	33,68		36,95	38,16	32,57	45,69	35,36	21,56	36,04	29,55	37,15
**Median**	44,91	37,06	49,4	40,83	40,33	37,91	47,76	41,26	36,81	44,22	36,4	42,42
**75% Percentile**	47,71	45,75		46,3	41,64	48,01	49,1	46,44	44,3	44,86	44,62	46,22
**Maximum**	51,97	49,23	52,21	48,7	42,91	49,82	50,87	46,81	48,09	48,95	49,56	51,68
**Mean**	45,75	38,61	49,4	41,01	39,98	39,85	46,91	40,9	32,06	37,95	37,05	41,78
**Std. Deviation**	3,632	7,22	3,974	5,073	2,206	7,661	3,444	6,468	14,01	13,78	8,076	5,803
**Std. Error**	1,095	2,283	2,81	1,196	0,9866	2,709	1,148	3,234	3,058	5,21	3,053	1,551
**Lower 95% CI of mean**	43,31	33,44	13,7	38,49	37,24	33,45	44,27	30,6	25,68	25,2	29,58	38,43
**Upper 95% CI of mean**	48,19	43,77	85,1	43,54	42,72	46,25	49,56	51,19	38,44	50,7	44,52	45,13
**Sum**	503,3	386,1	98,8	738,3	199,9	318,8	422,2	163,6	673,2	265,7	259,3	585

PC O 34:2

	Controls healthy	Controls Bronchitis	Diffuse development. disorder	Growth abn.+ defic. alveolar.	Immuno-intact host	Immuno-compromised host	Chronic tachypnea of infancy	Reactive lymphoid lesions	Related to alv. surf. region	Related to lung vessels/heart	Related to systemic disease	Unclear RDS in mature neonate
**Number of values**	11	10	2	18	5	8	9	4	21	7	7	13
**Minimum**	0,13	0,14	0,3	0	0,17	0,14	0,13	0,09	0,1	0,16	0,22	0,21
**25% Percentile**	0,17	0,235		0,18	0,21	0,28	0,14	0,175	0,26	0,22	0,34	0,265
**Median**	0,21	0,325	0,31	0,245	0,28	0,355	0,15	0,265	0,44	0,3	0,5	0,35
**75% Percentile**	0,25	0,915		0,445	0,4	0,945	0,245	0,375	1,365	0,55	0,63	0,54
**Maximum**	0,26	2,53	0,32	0,88	0,47	2,41	0,25	0,48	2,97	2,49	1,09	1,02
**Mean**	0,2073	0,642	0,31	0,3217	0,3	0,7138	0,1811	0,275	0,9148	0,6271	0,5357	0,4262
**Std. Deviation**	0,04338	0,739	0,01414	0,2282	0,1114	0,7633	0,0511	0,1597	0,8782	0,8327	0,2894	0,2174
**Std. Error**	0,01308	0,2337	0,01	0,05379	0,0498	0,2699	0,01703	0,07984	0,1916	0,3147	0,1094	0,06029
**Lower 95% CI of mean**	0,1781	0,1134	0,1829	0,2082	0,1617	0,07561	0,1418	0,0209	0,515	-0,1429	0,268	0,2948
**Upper 95% CI of mean**	0,2364	1,171	0,4371	0,4352	0,4383	1,352	0,2204	0,5291	1,315	1,397	0,8034	0,5575
**Sum**	2,28	6,42	0,62	5,79	1,5	5,71	1,63	1,1	19,21	4,39	3,75	5,54

PC O 34:1

	Controls healthy	Controls Bronchitis	Diffuse development. disorder	Growth abn.+ defic. alveolar.	Immuno-intact host	Immuno-compromised host	Chronic tachypnea of infancy	Reactive lymphoid lesions	Related to alv. surf. region	Related to lung vessels/heart	Related to systemic disease	Unclear RDS in mature neonate
**Number of values**	11	10	2	18	5	8	9	4	21	7	7	13
**Minimum**	0,78	0,84	0,87	0	1,27	0,92	0,64	0,87	0,57	0,55	0,91	0,62
**25% Percentile**	0,92	1,22		0,595	1,29	1,05	0,725	1,025	0,995	0,86	0,93	0,82
**Median**	1,17	1,745	1,06	0,805	1,48	1,355	0,85	1,34	1,28	1,19	1,29	0,98
**75% Percentile**	1,48	2,9		1,035	1,615	2,32	0,99	1,535	2,39	1,21	1,85	1,32

**Maximum**	1,97	5,42	1,25	1,88	1,71	5,18	1,08	1,57	4,46	5,89	2,62	1,95
**Mean**	1,213	2,136	1,06	0,8411	1,458	1,944	0,8633	1,28	1,79	1,724	1,503	1,099
**Std. Deviation**	0,3358	1,342	0,2687	0,3896	0,1768	1,43	0,1517	0,3218	1,216	1,853	0,6145	0,3613
**Std. Error**	0,1012	0,4243	0,19	0,09183	0,07908	0,5054	0,05055	0,1609	0,2655	0,7005	0,2322	0,1002
**Lower 95% CI of mean**	0,9871	1,176	-1,354	0,6474	1,238	0,7486	0,7468	0,768	1,237	0,0101	0,9346	0,8809
**Upper 95% CI of mean**	1,438	3,096	3,474	1,035	1,678	3,139	0,9799	1,792	2,344	3,438	2,071	1,318
**Sum**	13,34	21,36	2,12	15,14	7,29	15,55	7,77	5,12	37,6	12,07	10,52	14,29

PC 34:3

	**Controls healthy**	**Controls Bronchitis**	**Diffuse development. disorder**	**Growth abn.+ defic. alveolar.**	**Immuno-intact host**	**Immuno-compromised host**	**Chronic tachypnea of infancy**	**Reactive lymphoid lesions**	**Related to alv. surf. region**	**Related to lung vessels/heart**	**Related to systemic disease**	**Unclear RDS in mature neonate**
**Number of values**	11	10	2	18	5	8	9	4	21	7	7	13
**Minimum**	0,31	0,22	0,19	0	0,3	0,27	0,35	0,58	0	0,29	0,42	0,13
**25% Percentile**	0,35	0,335		0,56	0,4	0,435	0,42	0,605	0,23	0,35	0,53	0,405
**Median**	0,4	0,39	0,435	0,79	0,52	0,54	0,62	0,74	0,5	0,58	0,95	0,62
**75% Percentile**	0,8	0,675		1,125	0,555	0,91	0,71	0,88	0,65	0,69	1,03	0,855
**Maximum**	0,95	0,8	0,68	1,33	0,59	1,21	0,77	0,91	1,9	1,59	1,08	0,92
**Mean**	0,5373	0,462	0,435	0,7983	0,486	0,6563	0,5822	0,7425	0,5352	0,6686	0,7986	0,5923
**Std. Deviation**	0,2357	0,19	0,3465	0,3514	0,1095	0,3533	0,1536	0,1619	0,4571	0,4311	0,2847	0,2469
**Std. Error**	0,07106	0,06007	0,245	0,08283	0,04895	0,1249	0,0512	0,08097	0,09976	0,163	0,1076	0,06846
**Lower 95% CI of mean**	0,3789	0,3261	-2,678	0,6236	0,3501	0,3608	0,4642	0,4848	0,3272	0,2698	0,5352	0,4431
**Upper 95% CI of mean**	0,6956	0,5979	3,548	0,9731	0,6219	0,9517	0,7003	1	0,7433	1,067	1,062	0,7415
**Sum**	5,91	4,62	0,87	14,37	2,43	5,25	5,24	2,97	11,24	4,68	5,59	7,7

PC 34:2

	**Controls healthy**	**Controls Bronchitis**	**Diffuse development. disorder**	**Growth abn.+ defic. alveolar.**	**Immuno-intact host**	**Immuno-compromised host**	**Chronic tachypnea of infancy**	**Reactive lymphoid lesions**	**Related to alv. surf. region**	**Related to lung vessels/heart**	**Related to systemic disease**	**Unclear RDS in mature neonate**
**Number of values**	11	10	2	18	5	8	9	4	21	7	7	13
**Minimum**	3,58	1,64	2,53	0,92	4,42	1,86	3,67	5,11	3,14	2,79	3,74	3,06
**25% Percentile**	4,33	3,9		4,625	4,525	4,2	4,39	5,195	3,985	3,23	4,8	4,965
**Median**	5,58	4,545	3,755	5,845	4,84	5,025	4,84	5,83	5,61	4,69	5,69	5,16
**75% Percentile**	6,01	5,47		7,02	5,065	5,75	5,56	6,71	6,25	7,8	7,31	5,645
**Maximum**	7,75	5,58	4,98	7,99	5,23	7,34	7,36	7,04	29,99	9,57	8	6,47
**Mean**	5,407	4,438	3,755	5,566	4,804	4,894	5,026	5,953	6,377	5,371	5,817	5,225
**Std. Deviation**	1,249	1,171	1,732	1,761	0,304	1,644	1,082	0,9179	5,597	2,519	1,443	0,8117
**Std. Error**	0,3767	0,3704	1,225	0,4151	0,136	0,5811	0,3608	0,4589	1,221	0,952	0,5452	0,2251
**Lower 95% CI of mean**	4,568	3,6	-11,81	4,69	4,427	3,52	4,194	4,492	3,829	3,042	4,483	4,735
**Upper 95% CI of mean**	6,247	5,276	19,32	6,441	5,181	6,268	5,858	7,413	8,925	7,701	7,151	5,716
**Sum**	59,48	44,38	7,51	100,2	24,02	39,15	45,23	23,81	133,9	37,6	40,72	67,93

PC 34:1

	**Controls healthy**	**Controls Bronchitis**	**Diffuse development. disorder**	**Growth abn.+ defic. alveolar.**	**Immuno-intact host**	**Immuno-compromised host**	**Chronic tachypnea of infancy**	**Reactive lymphoid lesions**	**Related to alv. surf. region**	**Related to lung vessels/heart**	**Related to systemic disease**	**Unclear RDS in mature neonate**
**Number of values**	11	10	2	18	5	8	9	4	21	7	7	13
**Minimum**	8,26	7,3	8,86	5,06	9,8	7,26	8,88	8,34	8,67	8,35	7,02	8,91
**25% Percentile**	9,61	9,585		9,215	9,865	8,63	9,655	9,56	10,02	8,54	7,47	9,825
**Median**	10,72	12,03	9,65	11,01	10,16	10,26	10,81	11,12	11,57	11,31	12,06	11,97
**75% Percentile**	13,37	13,81		11,84	12,67	12,21	11,45	11,75	12,35	15,14	15,13	14,48
**Maximum**	16,15	15,41	10,44	13,21	14,67	13,14	12,31	12,04	19,71	21,75	15,19	15,51
**Mean**	11,14	11,75	9,65	10,54	11,05	10,33	10,65	10,65	12	12,53	11,3	12,2
**Std. Deviation**	2,326	2,404	1,117	1,9	2,053	2,09	1,103	1,625	3,049	4,916	3,582	2,388
**Std. Error**	0,7013	0,7601	0,79	0,4479	0,9181	0,7389	0,3675	0,8127	0,6654	1,858	1,354	0,6623
**Lower 95% CI of mean**	9,576	10,03	-0,3879	9,6	8,497	8,578	9,804	8,066	10,61	7,987	7,99	10,75
**Upper 95% CI of mean**	12,7	13,46	19,69	11,49	13,6	12,07	11,5	13,24	13,38	17,08	14,62	13,64
**Sum**	122,5	117,5	19,3	189,8	55,23	82,6	95,86	42,61	251,9	87,73	79,12	158,6

PC 34:0

	**Controls healthy**	**Controls Bronchitis**	**Diffuse development. disorder**	**Growth abn.+ defic. alveolar.**	**Immuno-intact host**	**Immuno-compromised host**	**Chronic tachypnea of infancy**	**Reactive lymphoid lesions**	**Related to alv. surf. region**	**Related to lung vessels/heart**	**Related to systemic disease**	**Unclear RDS in mature neonate**
**Number of values**	11	10	2	18	5	8	9	4	21	7	7	13
**Minimum**	0,84	1,09	0,93	0	1,29	0,67	1,1	1	0,2	0,98	0,67	0,42
**25% Percentile**	1,37	1,26		1,11	1,43	1,07	1,38	1,1	0,955	1,17	0,74	1,41
**Median**	1,43	1,375	1,01	1,64	1,8	1,24	1,7	1,355	1,27	1,29	0,96	1,61
**75% Percentile**	2,06	1,575		2,175	2	1,42	1,78	1,715	1,69	2,02	1,38	1,995
**Maximum**	2,74	2,1	1,09	2,56	2,04	1,45	2,36	1,92	2,4	2,04	1,93	2,24
**Mean**	1,61	1,439	1,01	1,588	1,732	1,198	1,637	1,408	1,264	1,466	1,089	1,588
**Std. Deviation**	0,5392	0,2789	0,1131	0,6478	0,3054	0,2643	0,3556	0,4009	0,5364	0,4174	0,4433	0,5051
**Std. Error**	0,1626	0,08818	0,08	0,1527	0,1366	0,09344	0,1185	0,2005	0,1171	0,1577	0,1676	0,1401

**Lower 95% CI of mean**	1,248	1,24	-0,006495	1,266	1,353	0,9765	1,363	0,7695	1,02	1,08	0,6786	1,282
**Upper 95% CI of mean**	1,972	1,638	2,026	1,91	2,111	1,418	1,91	2,046	1,508	1,852	1,499	1,893
**Sum**	17,71	14,39	2,02	28,59	8,66	9,58	14,73	5,63	26,54	10,26	7,62	20,64

PC O 36:4

	Controls healthy	Controls Bronchitis	Diffuse development. disorder	Growth abn.+ defic. alveolar.	Immuno-intact host	Immuno-compromised host	Chronic tachypnea of infancy	Reactive lymphoid lesions	Related to alv. surf. region	Related to lung vessels/heart	Related to systemic disease	Unclear RDS in mature neonate
**Number of values**	11	10	2	18	5	8	9	4	21	7	7	13
**Minimum**	0,05	0	0,22	0	0,2	0,07	0,03	0,07	0	0,07	0,22	0,05
**25% Percentile**	0,09	0,155		0,105	0,2	0,295	0,05	0,135	0,115	0,08	0,31	0,17
**Median**	0,15	0,24	0,285	0,185	0,46	0,575	0,13	0,425	0,73	0,15	0,56	0,3
**75% Percentile**	0,23	0,655		0,32	1,215	0,94	0,175	0,745	1,055	0,92	1,21	0,615
**Maximum**	0,32	2,26	0,35	2,14	1,22	2,37	0,36	0,84	3,37	0,93	1,25	4,46
**Mean**	0,1609	0,48	0,285	0,3661	0,658	0,7575	0,1344	0,44	0,7581	0,3714	0,6914	0,6854
**Std. Deviation**	0,08288	0,6803	0,09192	0,5342	0,5194	0,7304	0,1013	0,3645	0,7718	0,3879	0,4146	1,177
**Std. Error**	0,02499	0,2151	0,065	0,1259	0,2323	0,2582	0,03375	0,1823	0,1684	0,1466	0,1567	0,3266
**Lower 95% CI of mean**	0,1052	-0,006646	-0,5409	0,1004	0,01302	0,1469	0,05661	-0,14	0,4068	0,0127	0,308	-0,02613
**Upper 95% CI of mean**	0,2166	0,9666	1,111	0,6318	1,303	1,368	0,2123	1,02	1,109	0,7302	1,075	1,397
**Sum**	1,77	4,8	0,57	6,59	3,29	6,06	1,21	1,76	15,92	2,6	4,84	8,91

PC 36:4

	Controls healthy	Controls Bronchitis	Diffuse development. disorder	Growth abn.+ defic. alveolar.	Immuno-intact host	Immuno-compromised host	Chronic tachypnea of infancy	Reactive lymphoid lesions	Related to alv. surf. region	Related to lung vessels/heart	Related to systemic disease	Unclear RDS in mature neonate
**Number of values**	11	10	2	18	5	8	9	4	21	7	7	13
**Minimum**	0,74	0,71	1,98	0	1,07	0,59	0,73	1,22	0,29	0,49	1,27	0,68
**25% Percentile**	1,17	0,97		1,335	1,09	1,205	0,89	1,26	1,175	0,77	1,51	1,665
**Median**	1,41	1,175	2,17	1,745	1,63	1,605	1,26	1,41	1,91	1,3	1,84	1,98
**75% Percentile**	1,81	1,655		2,275	2,01	1,95	1,33	1,78	2,495	1,83	2,04	2,725
**Maximum**	2,04	2,05	2,36	2,95	2,28	2,1	2,23	2,04	7,75	2,31	2,2	7,69
**Mean**	1,419	1,272	2,17	1,728	1,566	1,526	1,23	1,52	2,146	1,361	1,791	2,394
**Std. Deviation**	0,3863	0,4019	0,2687	0,6667	0,4995	0,5195	0,4379	0,3691	1,685	0,6561	0,3191	1,713
**Std. Error**	0,1165	0,1271	0,19	0,1571	0,2234	0,1837	0,146	0,1846	0,3677	0,248	0,1206	0,4751
**Lower 95% CI of mean**	1,16	0,9845	-0,2442	1,396	0,9457	1,092	0,8934	0,9326	1,379	0,7547	1,496	1,359
**Upper 95% CI of mean**	1,679	1,559	4,584	2,059	2,186	1,961	1,567	2,107	2,913	1,968	2,087	3,429
**Sum**	15,61	12,72	4,34	31,1	7,83	12,21	11,07	6,08	45,06	9,53	12,54	31,12

PC 36:3

	Controls healthy	Controls Bronchitis	Diffuse development. disorder	Growth abn.+ defic. alveolar.	Immuno-intact host	Immuno-compromised host	Chronic tachypnea of infancy	Reactive lymphoid lesions	Related to alv. surf. region	Related to lung vessels/heart	Related to systemic disease	Unclear RDS in mature neonate
**Number of values**	11	10	2	18	5	8	9	4	21	7	7	13
**Minimum**	0,89	0,73	0,7	0,92	1,42	0,33	0,9	1,4	0,58	0,62	1,18	0,47
**25% Percentile**	1,13	0,78		1,57	1,5	1,295	1,065	1,515	1,015	1,06	1,56	1,505
**Median**	1,51	1,395	1,14	2,37	1,75	1,64	1,76	1,855	1,52	1,32	1,71	1,97
**75% Percentile**	2,09	1,625		2,695	1,805	2,155	1,81	2,235	2,36	3,11	2,25	2,565
**Maximum**	2,81	2,48	1,58	4,21	1,83	3,47	2,28	2,39	7,51	6,04	2,32	3,02
**Mean**	1,648	1,315	1,14	2,279	1,672	1,748	1,569	1,875	2,109	2,347	1,84	1,999
**Std. Deviation**	0,5651	0,5459	0,6223	0,7651	0,1693	0,928	0,4599	0,4446	1,647	1,891	0,4309	0,7017
**Std. Error**	0,1704	0,1726	0,44	0,1803	0,07572	0,3281	0,1533	0,2223	0,3595	0,7146	0,1628	0,1946
**Lower 95% CI of mean**	1,269	0,9245	-4,451	1,898	1,462	0,9716	1,215	1,168	1,359	0,5987	1,442	1,575
**Upper 95% CI of mean**	2,028	1,705	6,731	2,659	1,882	2,523	1,922	2,582	2,859	4,096	2,238	2,423
**Sum**	18,13	13,15	2,28	41,02	8,36	13,98	14,12	7,5	44,29	16,43	12,88	25,99

PC 36.2

	Controls healthy	Controls Bronchitis	Diffuse development. disorder	Growth abn.+ defic. alveolar.	Immuno-intact host	Immuno-compromised host	Chronic tachypnea of infancy	Reactive lymphoid lesions	Related to alv. surf. region	Related to lung vessels/heart	Related to systemic disease	Unclear RDS in mature neonate
**Number of values**	11	10	2	18	5	8	9	4	21	7	7	13
**Minimum**	1,44	1,08	1,1	0,82	2,12	1,22	1,58	1,86	1,24	1,36	1,46	1,61
**25% Percentile**	1,75	1,645		2,15	2,17	2,11	1,95	2,16	1,84	1,64	1,76	2,71
**Median**	2,24	2,135	1,45	2,89	2,47	2,48	2,34	2,505	2,64	2,91	2,23	3,03
**75% Percentile**	2,83	2,565		3,35	2,795	2,96	2,62	2,74	4,305	6,03	3,82	3,37
**Maximum**	3,87	4,46	1,8	6,12	2,86	3,97	2,67	2,93	13,95	13,24	3,87	4,16
**Mean**	2,411	2,257	1,45	2,834	2,48	2,536	2,258	2,45	3,72	4,449	2,551	2,985
**Std. Deviation**	0,7791	0,9022	0,495	1,093	0,3179	0,8256	0,3898	0,4429	3,176	4,179	1,001	0,5989
**Std. Error**	0,2349	0,2853	0,35	0,2576	0,1422	0,2919	0,1299	0,2215	0,693	1,579	0,3784	0,1661
**Lower 95% CI of mean**	1,887	1,612	-2,997	2,29	2,085	1,846	1,958	1,745	2,274	0,5839	1,625	2,623
**Upper 95% CI of mean**	2,934	2,902	5,897	3,377	2,875	3,227	2,557	3,155	5,166	8,313	3,477	3,347
**Sum**	26,52	22,57	2,9	51,01	12,4	20,29	20,32	9,8	78,12	31,14	17,86	38,81

PC 36:1

	Controls healthy	Controls Bronchitis	Diffuse development. disorder	Growth abn.+ defic. alveolar.	Immuno-intact host	Immuno-compromised host	Chronic tachypnea of infancy	Reactive lymphoid lesions	Related to alv. surf. region	Related to lung vessels/heart	Related to systemic disease	Unclear RDS in mature neonate
**Number of values**	11	10	2	18	5	8	9	4	21	7	7	13
**Minimum**	0,76	0,61	0,66	0	0,92	0,66	0,72	0,73	0,48	0,85	0,47	0,9
**25% Percentile**	0,82	0,895		0,68	1,015	0,705	0,875	0,765	0,845	0,88	0,53	0,97
**Median**	0,96	1,405	0,695	1,025	1,2	0,78	0,95	0,805	1,57	1,14	1,1	1,16
**75% Percentile**	1,3	1,865		1,225	1,84	0,92	1,06	0,955	2,675	1,94	1,34	1,69
**Maximum**	1,65	2,25	0,73	2,43	2,25	1,49	1,23	1,1	6,49	6,49	1,39	2,28
**Mean**	1,065	1,408	0,695	1,011	1,382	0,87	0,9711	0,86	1,953	1,911	0,94	1,368
**Std. Deviation**	0,2975	0,5234	0,0495	0,505	0,5187	0,2703	0,145	0,1639	1,566	2,054	0,3975	0,4754
**Std. Error**	0,0897	0,1655	0,035	0,119	0,232	0,09556	0,04835	0,08196	0,3418	0,7763	0,1502	0,1319
**Lower 95% CI of mean**	0,8647	1,034	0,2503	0,76	0,7379	0,644	0,8596	0,5992	1,24	0,01178	0,5724	1,08
**Upper 95% CI of mean**	1,264	1,782	1,14	1,262	2,026	1,096	1,083	1,121	2,666	3,811	1,308	1,655
**Sum**	11,71	14,08	1,39	18,2	6,91	6,96	8,74	3,44	41,02	13,38	6,58	17,78

PC 38:5

	Controls healthy	Controls Bronchitis	Diffuse development. disorder	Growth abn.+ defic. alveolar.	Immuno-intact host	Immuno-compromised host	Chronic tachypnea of infancy	Reactive lymphoid lesions	Related to alv. surf. region	Related to lung vessels/heart	Related to systemic disease	Unclear RDS in mature neonate
**Number of values**	11	10	2	18	5	8	9	4	21	7	7	13
**Minimum**	0,13	0,16	0,34	0	0,33	0,15	0,19	0,29	0	0,14	0,24	0,09
**25% Percentile**	0,2	0,265		0,295	0,355	0,25	0,205	0,29	0,235	0,23	0,33	0,335
**Median**	0,28	0,295	0,375	0,385	0,49	0,45	0,21	0,35	0,51	0,33	0,39	0,44
**75% Percentile**	0,4	0,365		0,59	0,58	0,57	0,27	0,425	0,6	0,44	0,53	0,67
**Maximum**	0,45	0,47	0,41	0,81	0,59	0,65	0,63	0,44	2,59	0,75	0,78	1,22
**Mean**	0,2864	0,308	0,375	0,4144	0,472	0,4175	0,2689	0,3575	0,5824	0,3643	0,44	0,5054
**Std. Deviation**	0,1091	0,08176	0,0495	0,1992	0,1145	0,1823	0,1388	0,0789	0,557	0,1956	0,1742	0,2927
**Std. Error**	0,0329	0,02585	0,035	0,04695	0,05122	0,06444	0,04626	0,03945	0,1215	0,07393	0,06583	0,08117
**Lower 95% CI of mean**	0,2131	0,2495	-0,06972	0,3154	0,3298	0,2651	0,1622	0,232	0,3288	0,1834	0,2789	0,3285
**Upper 95% CI of mean**	0,3597	0,3665	0,8197	0,5135	0,6142	0,5699	0,3756	0,483	0,8359	0,5452	0,6011	0,6822
**Sum**	3,15	3,08	0,75	7,46	2,36	3,34	2,42	1,43	12,23	2,55	3,08	6,57

PC 38:4

	Controls healthy	Controls Bronchitis	Diffuse development. disorder	Growth abn.+ defic. alveolar.	Immuno-intact host	Immuno-compromised host	Chronic tachypnea of infancy	Reactive lymphoid lesions	Related to alv. surf. region	Related to lung vessels/heart	Related to systemic disease	Unclear RDS in mature neonate
**Number of values**	11	10	2	18	5	8	9	4	21	7	7	13
**Minimum**	0,14	0,14	0,45	0	0,53	0,31	0,18	0,23	0,13	0,22	0,32	0,24
**25% Percentile**	0,31	0,265		0,35	0,57	0,42	0,25	0,27	0,35	0,26	0,45	0,435
**Median**	0,42	0,345	0,485	0,56	0,66	0,525	0,29	0,4	0,89	0,37	0,6	0,62
**75% Percentile**	0,58	0,505		0,905	0,865	0,715	0,35	0,56	1,27	0,56	0,78	1,22
**Maximum**	0,72	0,63	0,52	1,11	0,9	1,17	0,72	0,63	3,26	0,98	1	3,24
**Mean**	0,4364	0,373	0,485	0,5928	0,706	0,6	0,33	0,415	1,04	0,4543	0,6057	0,93
**Std. Deviation**	0,1832	0,1461	0,0495	0,3107	0,1544	0,2728	0,1564	0,1799	0,8347	0,2583	0,2255	0,7766
**Std. Error**	0,05522	0,04619	0,035	0,07323	0,06904	0,09644	0,05215	0,08995	0,1821	0,09764	0,08524	0,2154
**Lower 95% CI of mean**	0,3133	0,2685	0,04028	0,4383	0,5143	0,372	0,2097	0,1287	0,6605	0,2154	0,3971	0,4607
**Upper 95% CI of mean**	0,5594	0,4775	0,9297	0,7473	0,8977	0,828	0,4503	0,7013	1,42	0,6932	0,8143	1,399
**Sum**	4,8	3,73	0,97	10,67	3,53	4,8	2,97	1,66	21,85	3,18	4,24	12,09

PC O 34:0

	Controls healthy	Controls Bronchitis	Diffuse development. disorder	Growth abn.+ defic. alveolar.	Immuno-intact host	Immuno-compromised host	Chronic tachypnea of infancy	Reactive lymphoid lesions	Related to alv. surf. region	Related to lung vessels/heart	Related to systemic disease	Unclear RDS in mature neonate
**Number of values**	11	10	2	18	5	8	9	4	21	7	7	13
**Minimum**	0,6	1	0,5	0	1,6	0,5	0,4	0,3	0,1	0,4	0,5	0,3
**25% Percentile**	1	1,1		0,5	1,6	0,65	0,7	0,8	0,75	0,6	1	0,55
**Median**	2	1,85	0,55	0,6	1,6	1,05	1,1	1,5	1,1	0,7	1,2	0,7
**75% Percentile**	2,3	2,65		0,95	1,95	1,15	1,45	1,85	1,6	1,1	1,2	0,9
**Maximum**	2,5	2,8	0,6	1,5	2,2	1,3	1,9	2	2,3	2,2	1,7	1,1
**Mean**	1,745	1,86	0,55	0,7111	1,74	0,9375	1,133	1,325	1,195	0,9571	1,129	0,7
**Std. Deviation**	0,6832	0,7106	0,07071	0,327	0,2608	0,2973	0,4743	0,7411	0,6037	0,6079	0,3546	0,238
**Std. Error**	0,206	0,2247	0,05	0,07707	0,1166	0,1051	0,1581	0,3705	0,1317	0,2298	0,134	0,06602
**Lower 95% CI of mean**	1,286	1,352	-0,08531	0,5485	1,416	0,6889	0,7687	0,1458	0,9204	0,3949	0,8007	0,5562
**Upper 95% CI of mean**	2,204	2,368	1,185	0,8737	2,064	1,186	1,498	2,504	1,47	1,519	1,456	0,8439
**Sum**	19,2	18,6	1,1	12,8	8,7	7,5	10,2	5,3	25,1	6,7	7,9	9,1

PE 32:2

	Controls healthy	Controls Bronchitis	Diffuse development. disorder	Growth abn.+ defic. alveolar.	Immuno-intact host	Immuno-compromised host	Chronic tachypnea of infancy	Reactive lymphoid lesions	Related to alv. surf. region	Related to lung vessels/heart	Related to systemic disease	Unclear RDS in mature neonate
**Number of values**	11	10	2	18	5	8	9	4	21	7	7	13
**Minimum**	0,38	0,29	0,34	0,19	0,31	0,29	0,33	0,41	0	0,21	0,3	0,23
**25% Percentile**	0,45	0,345		0,345	0,395	0,385	0,38	0,6	0,34	0,27	0,32	0,36
**Median**	0,54	0,485	0,505	0,395	0,54	0,485	0,45	0,87	0,47	0,37	0,57	0,45
**75% Percentile**	0,62	0,6		0,865	0,705	0,67	0,545	1,015	0,65	0,55	0,74	0,6
**Maximum**	0,77	0,72	0,67	2,31	0,71	0,84	0,62	1,08	1,3	1,04	1,26	0,78
**Mean**	0,5427	0,476	0,505	0,625	0,548	0,5263	0,4611	0,8075	0,5267	0,4614	0,6257	0,4746
**Std. Deviation**	0,1117	0,1403	0,2333	0,5021	0,1663	0,1912	0,09623	0,2903	0,3246	0,2811	0,3261	0,1578
**Std. Error**	0,03369	0,04438	0,165	0,1183	0,07439	0,06761	0,03208	0,1452	0,07084	0,1062	0,1232	0,04377
**Lower 95% CI of mean**	0,4677	0,3756	-1,592	0,3753	0,3415	0,3664	0,3871	0,3455	0,3789	0,2015	0,3241	0,3792
**Upper 95% CI of mean**	0,6178	0,5764	2,602	0,8747	0,7545	0,6861	0,5351	1,269	0,6744	0,7214	0,9273	0,57
**Sum**	5,97	4,76	1,01	11,25	2,74	4,21	4,15	3,23	11,06	3,23	4,38	6,17

PE 32:1

	Controls healthy	Controls Bronchitis	Diffuse development. disorder	Growth abn.+ defic. alveolar.	Immuno-intact host	Immuno-compromised host	Chronic tachypnea of infancy	Reactive lymphoid lesions	Related to alv. surf. region	Related to lung vessels/heart	Related to systemic disease	Unclear RDS in mature neonate
**Number of values**	11	10	2	18	5	8	9	4	21	7	7	13
**Minimum**	1,44	1,65	1,73	1,11	2,22	1,86	1,63	2,08	0,47	1,13	1,79	1,4
**25% Percentile**	1,74	2,1		1,87	2,28	2,25	2,125	2,66	1,27	1,41	1,98	1,56
**Median**	1,92	2,97	2,065	2,545	2,67	2,87	2,42	3,36	1,97	2,26	2,68	1,89
**75% Percentile**	3,48	3,825		3,31	3,055	3,375	2,875	4,03	2,7	3,41	4,64	2,95
**Maximum**	4,09	4,2	2,4	3,7	3,27	3,93	3,43	4,58	6,91	3,62	5,49	4,59
**Mean**	2,334	2,944	2,065	2,515	2,668	2,848	2,472	3,345	2,292	2,291	3,057	2,275
**Std. Deviation**	0,8993	0,873	0,4738	0,7687	0,4184	0,709	0,5525	1,025	1,517	0,9875	1,443	0,9148
**Std. Error**	0,2712	0,2761	0,335	0,1812	0,1871	0,2507	0,1842	0,5127	0,3311	0,3732	0,5453	0,2537
**Lower 95% CI of mean**	1,729	2,319	-2,192	2,133	2,148	2,255	2,048	1,713	1,602	1,378	1,723	1,722
**Upper 95% CI of mean**	2,938	3,569	6,322	2,897	3,188	3,44	2,897	4,977	2,983	3,205	4,392	2,827
**Sum**	25,67	29,44	4,13	45,27	13,34	22,78	22,25	13,38	48,14	16,04	21,4	29,57

PE 32:0

	Controls healthy	Controls Bronchitis	Diffuse development. disorder	Growth abn.+ defic. alveolar.	Immuno-intact host	Immuno-compromised host	Chronic tachypnea of infancy	Reactive lymphoid lesions	Related to alv. surf. region	Related to lung vessels/heart	Related to systemic disease	Unclear RDS in mature neonate
**Number of values**	11	10	2	18	5	8	8	3	21	7	7	13
**Minimum**	0,85	0,78	1,49	1,1	0,94	1,33	0,95	1,17	0	1,06	1	1,19
**25% Percentile**	1,04	1,065		1,44	1,18	1,645	1,25		0,83	1,08	1,59	1,395
**Median**	1,45	1,21	1,6	1,68	1,42	1,825	1,37	1,57	1,39	2,02	1,81	1,75
**75% Percentile**	1,74	2,13		2,185	1,825	2,145	1,555		1,935	2,05	2,24	2,18
**Maximum**	3,58	3,11	1,71	3,49	2,03	3,19	1,71	1,59	2,85	2,42	2,81	2,73
**Mean**	1,642	1,514	1,6	1,873	1,486	1,969	1,376	1,443	1,39	1,721	1,906	1,795
**Std. Deviation**	0,784	0,7297	0,1556	0,6146	0,3939	0,5772	0,2427	0,2369	0,7331	0,5404	0,5642	0,4847
**Std. Error**	0,2364	0,2308	0,11	0,1449	0,1762	0,2041	0,08579	0,1368	0,16	0,2042	0,2132	0,1344
**Lower 95% CI of mean**	1,115	0,992	0,2023	1,567	0,9969	1,486	1,173	0,8548	1,056	1,222	1,384	1,502
**Upper 95% CI of mean**	2,169	2,036	2,998	2,178	1,975	2,451	1,579	2,032	1,724	2,221	2,427	2,087
**Sum**	18,06	15,14	3,2	33,71	7,43	15,75	11,01	4,33	29,19	12,05	13,34	23,33

PE 34:3

	Controls healthy	Controls Bronchitis	Diffuse development. disorder	Growth abn.+ defic. alveolar.	Immuno-intact host	Immuno-compromised host	Chronic tachypnea of infancy	Reactive lymphoid lesions	Related to alv. surf. region	Related to lung vessels/heart	Related to systemic disease	Unclear RDS in mature neonate
**Number of values**	11	10	2	18	5	8	9	4	21	7	7	13
**Minimum**	0,73	0,47	0,44	0,54	0,54	0,5	0,75	1,02	0	0,29	0,53	0,39
**25% Percentile**	0,84	0,735		0,695	0,71	0,69	0,835	1,09	0,495	0,72	0,64	0,55
**Median**	0,95	0,78	0,64	0,905	0,94	0,865	0,94	1,29	1,07	0,86	0,92	0,74
**75% Percentile**	1,18	1,07		1,165	1,17	1,125	1,135	1,44	1,26	1,13	1,04	0,91
**Maximum**	1,55	1,24	0,84	1,52	1,37	1,3	1,3	1,46	2,23	1,13	1,67	1,13
**Mean**	1,022	0,854	0,64	0,9489	0,94	0,895	0,9911	1,265	0,9195	0,8686	0,9443	0,7323
**Std. Deviation**	0,2451	0,2233	0,2828	0,2961	0,2956	0,2839	0,181	0,2106	0,5513	0,3007	0,3679	0,2148
**Std. Error**	0,07391	0,07062	0,2	0,06979	0,1322	0,1004	0,06033	0,1053	0,1203	0,1137	0,1391	0,05957
**Lower 95% CI of mean**	0,8571	0,6942	-1,901	0,8016	0,573	0,6576	0,852	0,9298	0,6686	0,5904	0,604	0,6025
**Upper 95% CI of mean**	1,187	1,014	3,181	1,096	1,307	1,132	1,13	1,6	1,17	1,147	1,285	0,8621
**Sum**	11,24	8,54	1,28	17,08	4,7	7,16	8,92	5,06	19,31	6,08	6,61	9,52

PE 34:2

	Controls healthy	Controls Bronchitis	Diffuse development. disorder	Growth abn.+ defic. alveolar.	Immuno-intact host	Immuno-compromised host	Chronic tachypnea of infancy	Reactive lymphoid lesions	Related to alv. surf. region	Related to lung vessels/heart	Related to systemic disease	Unclear RDS in mature neonate
**Number of values**	11	10	2	18	5	8	9	4	21	7	7	13

**Minimum**	7,4	4,58	5,01	2,32	8,33	5,92	8,86	10,61	3,23	2,59	6,51	4,73
**25% Percentile**	8,04	6,89		7,3	8,705	7,11	9,555	11,48	4,6	7,91	7,3	6,245
**Median**	9,8	9,475	5,465	8,25	9,31	10,58	12,22	12,43	6,97	10,01	9,45	7,5
**75% Percentile**	11,86	10,36		11,47	10,22	11,23	12,95	12,61	8,8	10,88	9,73	9,165
**Maximum**	13,23	10,53	5,92	14,21	10,76	13,75	13,12	12,69	18,52	11,44	12,38	12,5
**Mean**	9,809	8,813	5,465	8,763	9,432	9,686	11,37	12,04	7,63	8,756	9,064	7,768
**Std. Deviation**	2,119	2,011	0,6435	2,815	0,8914	2,714	1,709	0,964	3,834	3,012	1,9	2,146
**Std. Error**	0,639	0,6359	0,455	0,6635	0,3986	0,9596	0,5696	0,482	0,8367	1,138	0,7182	0,5953
**Lower 95% CI of mean**	8,385	7,375	-0,3163	7,364	8,325	7,417	10,06	10,51	5,885	5,97	7,307	6,471
**Upper 95% CI of mean**	11,23	10,25	11,25	10,16	10,54	11,96	12,68	13,57	9,375	11,54	10,82	9,065
**Sum**	107,9	88,13	10,93	157,7	47,16	77,49	102,3	48,16	160,2	61,29	63,45	101

PE 34:1

	Controls healthy	Controls Bronchitis	Diffuse development. disorder	Growth abn.+ defic. alveolar.	Immuno-intact host	Immuno-compromised host	Chronic tachypnea of infancy	Reactive lymphoid lesions	Related to alv. surf. region	Related to lung vessels/heart	Related to systemic disease	Unclear RDS in mature neonate
**Number of values**	11	11	2	18	5	8	9	4	21	7	7	13
**Minimum**	16,18	17,44	23,56	4,85	17,58	20,19	21,11	19,25	6,44	18,18	18,8	15,12
**25% Percentile**	20	20,86		20,21	17,74	22,19	21,64	19,3	16,48	19,81	22,96	19,18
**Median**	22,12	22,08	23,66	22,6	18,98	23,93	22,81	19,95	20,19	22,83	24,58	20,79
**75% Percentile**	24,42	24,1		24,91	23,56	25,73	27,28	22,34	23,78	24,52	28,1	23,23
**Maximum**	27,82	25,95	23,75	27,23	26,11	28,29	29,28	24,13	29,14	26,17	28,8	32,49
**Mean**	22,3	22,21	23,66	21,59	20,31	24,02	24,18	20,82	19,74	22,51	24,68	21,89
**Std. Deviation**	3,279	2,57	0,1344	4,958	3,507	2,709	3,003	2,285	5,918	2,731	3,371	4,405
**Std. Error**	0,9887	0,7748	0,095	1,169	1,568	0,9578	1,001	1,142	1,291	1,032	1,274	1,222
**Lower 95% CI of mean**	20,1	20,49	22,45	19,13	15,96	21,76	21,87	17,18	17,04	19,98	21,56	19,22
**Upper 95% CI of mean**	24,51	23,94	24,86	24,06	24,67	26,29	26,49	24,45	22,43	25,03	27,79	24,55
**Sum**	245,4	244,4	47,31	388,7	101,6	192,2	217,6	83,27	414,5	157,6	172,7	284,5

PE 34:0

	Controls healthy	Controls Bronchitis	Diffuse development. disorder	Growth abn.+ defic. alveolar.	Immuno-intact host	Immuno-compromised host	Chronic tachypnea of infancy	Reactive lymphoid lesions	Related to alv. surf. region	Related to lung vessels/heart	Related to systemic disease	Unclear RDS in mature neonate
**Number of values**	11	10	2	18	5	8	9	4	21	7	7	13
**Minimum**	0,72	0,99	1,19	0,6	1,31	1,08	0,95	0,71	0	0,79	0,51	0,56
**25% Percentile**	1,01	1,075		0,87	1,43	1,135	1,01	1,135	0,73	0,81	0,81	0,96
**Median**	1,2	1,395	1,31	1,375	1,92	1,99	1,33	1,655	1,12	1,39	1,42	1,16
**75% Percentile**	1,48	1,91		1,695	2,1	2,36	1,695	2,625	2	1,68	1,77	1,41
**Maximum**	2,54	2,1	1,43	3,57	2,11	2,6	1,98	3,5	4,4	1,69	2,41	1,69
**Mean**	1,343	1,459	1,31	1,45	1,796	1,831	1,368	1,88	1,377	1,253	1,414	1,161
**Std. Deviation**	0,4911	0,4248	0,1697	0,7248	0,3525	0,6297	0,3808	1,171	1,017	0,4069	0,6325	0,3291
**Std. Error**	0,1481	0,1343	0,12	0,1708	0,1577	0,2226	0,1269	0,5854	0,222	0,1538	0,2391	0,09128
**Lower 95% CI of mean**	1,013	1,155	-0,2147	1,09	1,358	1,305	1,075	0,01692	0,9137	0,8765	0,8293	0,9619
**Upper 95% CI of mean**	1,673	1,763	2,835	1,81	2,234	2,358	1,661	3,743	1,84	1,629	1,999	1,36
**Sum**	14,77	14,59	2,62	26,1	8,98	14,65	12,31	7,52	28,91	8,77	9,9	15,09

PE 36:5

	Controls healthy	Controls Bronchitis	Diffuse development. disorder	Growth abn.+ defic. alveolar.	Immuno-intact host	Immuno-compromised host	Chronic tachypnea of infancy	Reactive lymphoid lesions	Related to alv. surf. region	Related to lung vessels/heart	Related to systemic disease	Unclear RDS in mature neonate
**Number of values**	11	10	2	18	5	8	9	4	21	7	7	13
**Minimum**	0,32	0,41	0,4	0,31	0,35	0,39	0,35	0,49	0,06	0,29	0,52	0,42
**25% Percentile**	0,45	0,46		0,515	0,425	0,48	0,36	0,57	0,305	0,31	0,52	0,49
**Median**	0,54	0,6	0,62	0,615	0,64	0,61	0,47	0,75	0,5	0,47	0,6	0,61
**75% Percentile**	0,71	0,76		0,74	0,87	0,8	0,695	0,97	0,86	1	0,93	0,705
**Maximum**	1,03	0,82	0,84	1,08	1,08	1,05	0,86	1,09	1,1	1,13	1,1	0,88
**Mean**	0,59	0,614	0,62	0,6289	0,646	0,6525	0,5289	0,77	0,5652	0,6071	0,6957	0,6138
**Std. Deviation**	0,1924	0,1517	0,3111	0,1895	0,2727	0,233	0,1842	0,2592	0,3083	0,331	0,2265	0,146
**Std. Error**	0,05801	0,04799	0,22	0,04468	0,122	0,08237	0,06141	0,1296	0,06728	0,1251	0,0856	0,04049
**Lower 95% CI of mean**	0,4607	0,5054	-2,175	0,5346	0,3074	0,4577	0,3873	0,3575	0,4249	0,301	0,4862	0,5256
**Upper 95% CI of mean**	0,7193	0,7226	3,415	0,7231	0,9846	0,8473	0,6705	1,182	0,7056	0,9133	0,9052	0,7021
**Sum**	6,49	6,14	1,24	11,32	3,23	5,22	4,76	3,08	11,87	4,25	4,87	7,98

PE 36:4

	Controls healthy	Controls Bronchitis	Diffuse development. disorder	Growth abn.+ defic. alveolar.	Immuno-intact host	Immuno-compromised host	Chronic tachypnea of infancy	Reactive lymphoid lesions	Related to alv. surf. region	Related to lung vessels/heart	Related to systemic disease	Unclear RDS in mature neonate
**Number of values**	11	10	2	18	5	8	8	3	21	7	7	9
**Minimum**	1,89	2,65	2,77	1,3	2,64	2,41	2,97	3,22	0	1,39	2,76	3,09
**25% Percentile**	3,13	2,805		2,79	2,97	4,205	3,275		1,57	2,8	4,02	3,575
**Median**	4,09	3,48	3,51	3,64	4,68	4,51	3,815	3,7	2,72	4,06	5,22	3,79
**75% Percentile**	4,56	4,105		4,995	5,61	4,935	4,585		4	4,4	5,98	4,545

	Controls healthy	Controls Bronchitis	Diffuse development. disorder	Growth abn.+ defic. alveolar.	Immuno-intact host	Immuno-compromised host	Chronic tachypnea of infancy	Reactive lymphoid lesions	Related to alv. surf. region	Related to lung vessels/heart	Related to systemic disease	Unclear RDS in mature neonate
**Maximum**	4,78	4,7	4,25	6,34	6,08	5,78	5,61	5,9	7,82	5,06	6,54	6,74
**Mean**	3,793	3,468	3,51	3,823	4,368	4,436	3,991	4,273	3,117	3,603	4,884	4,173
**Std. Deviation**	0,9627	0,6882	1,047	1,481	1,392	0,971	0,9454	1,429	2,06	1,241	1,284	1,075
**Std. Error**	0,2903	0,2176	0,74	0,3491	0,6225	0,3433	0,3342	0,8251	0,4495	0,469	0,4853	0,3583
**Lower 95% CI of mean**	3,146	2,976	-5,893	3,086	2,64	3,624	3,201	0,7234	2,179	2,455	3,697	3,347
**Upper 95% CI of mean**	4,439	3,96	12,91	4,559	6,096	5,248	4,782	7,823	4,055	4,751	6,072	5
**Sum**	41,72	34,68	7,02	68,81	21,84	35,49	31,93	12,82	65,46	25,22	34,19	37,56

PE 36:3

	Controls healthy	Controls Bronchitis	Diffuse development. disorder	Growth abn.+ defic. alveolar.	Immuno-intact host	Immuno-compromised host	Chronic tachypnea of infancy	Reactive lymphoid lesions	Related to alv. surf. region	Related to lung vessels/heart	Related to systemic disease	Unclear RDS in mature neonate
**Number of values**	11	10	2	18	5	8	9	4	21	7	7	13
**Minimum**	6,66	1,64	2,5	1,54	7,3	3,73	6,49	7,71	1,61	1,83	3,51	1,48
**25% Percentile**	7,27	3,835		4,31	7,425	4,46	7,845	7,935	2,36	5,28	3,59	2,915
**Median**	9,02	8,29	2,98	7,265	8,42	7,605	10,62	8,165	3,45	5,77	4,8	5,15
**75% Percentile**	12,06	9,955		10,57	10,35	9,81	13,1	11,14	6,9	15,84	7,32	9,28
**Maximum**	14,59	10,77	3,46	15,09	12	11,23	14,95	14,11	12,54	18,93	9,84	13,72
**Mean**	9,692	7,253	2,98	7,469	8,792	7,339	10,69	9,537	4,76	8,539	5,571	6,068
**Std. Deviation**	2,543	3,172	0,6788	3,556	1,885	2,867	2,962	3,056	3,05	6,292	2,292	3,785
**Std. Error**	0,7666	1,003	0,48	0,8381	0,8429	1,014	0,9874	1,528	0,6656	2,378	0,8663	1,05
**Lower 95% CI of mean**	7,984	4,984	-3,119	5,701	6,452	4,942	8,41	4,675	3,372	2,719	3,452	3,781
**Upper 95% CI of mean**	11,4	9,522	9,079	9,238	11,13	9,735	12,96	14,4	6,149	14,36	7,691	8,356
**Sum**	106,6	72,53	5,96	134,5	43,96	58,71	96,18	38,15	99,97	59,77	39	78,89

PE 36:2

	Controls healthy	Controls Bronchitis	Diffuse development. disorder	Growth abn.+ defic. alveolar.	Immuno-intact host	Immuno-compromised host	Chronic tachypnea of infancy	Reactive lymphoid lesions	Related to alv. surf. region	Related to lung vessels/heart	Related to systemic disease	Unclear RDS in mature neonate
**Number of values**	11	10	2	18	5	8	9	4	21	7	7	13
**Minimum**	13,64	9,95	9,36	1,69	11,66	11,55	14,9	12,86	5,61	11,02	8,28	7,16
**25% Percentile**	16,16	13,09		10,69	12,29	12,53	16,41	12,92	9,575	12,53	10,99	10,65
**Median**	17,1	16,38	10,66	15,4	13,42	15,97	17,38	13,1	11,78	13,61	12,09	12,59
**75% Percentile**	18,17	18,39		20,11	19	16,56	20,18	16,74	13,12	20,59	13,96	17,91
**Maximum**	22,76	18,92	11,95	22,2	19,42	21,91	22,86	20,24	25,9	28,85	19,26	19,92
**Mean**	17,42	15,71	10,66	14,73	15,2	15,45	18,27	14,83	11,94	16,69	12,85	13,39
**Std. Deviation**	2,288	2,985	1,831	5,435	3,538	3,326	2,53	3,613	4,424	6,284	3,412	4,047
**Std. Error**	0,6898	0,9439	1,295	1,281	1,582	1,176	0,8434	1,807	0,9654	2,375	1,29	1,122
**Lower 95% CI of mean**	15,88	13,57	-5,8	12,03	10,81	12,67	16,32	9,075	9,926	10,88	9,696	10,95
**Upper 95% CI of mean**	18,96	17,85	27,11	17,43	19,59	18,23	20,21	20,57	13,95	22,51	16,01	15,84
**Sum**	191,6	157,1	21,31	265,1	75,99	123,6	164,4	59,3	250,7	116,9	89,96	174,1

PE 36:1

	Controls healthy	Controls Bronchitis	Diffuse development. disorder	Growth abn.+ defic. alveolar.	Immuno-intact host	Immuno-compromised host	Chronic tachypnea of infancy	Reactive lymphoid lesions	Related to alv. surf. region	Related to lung vessels/heart	Related to systemic disease	Unclear RDS in mature neonate
**Number of values**	11	10	2	18	5	8	9	4	21	7	7	13
**Minimum**	7,15	10,31	13,01	2,23	7,33	6,02	7,59	6,03	5,06	7,17	7,49	7,28
**25% Percentile**	8,14	10,73		8,685	7,84	7,875	8,92	6,15	7,275	8,49	8,24	11,63
**Median**	9,06	11,76	15,13	11	8,58	9,785	9,17	7,93	10,02	13,76	10,27	14,66
**75% Percentile**	10,13	21,12		13,41	11,27	13,25	10,37	9,925	20,82	14,81	15,48	16,64
**Maximum**	11,99	31,25	17,24	17,79	13,09	15,37	11,8	10,26	39,21	30,39	16,24	22,05
**Mean**	9,156	15,48	15,13	11,13	9,358	10,4	9,56	8,038	14,77	13,94	11,45	14,22
**Std. Deviation**	1,353	7,024	2,991	3,628	2,217	3,287	1,195	2,199	9,667	7,865	3,687	3,757
**Std. Error**	0,4078	2,221	2,115	0,8552	0,9917	1,162	0,3983	1,099	2,11	2,973	1,394	1,042
**Lower 95% CI of mean**	8,248	10,46	-11,75	9,325	6,605	7,652	8,642	4,539	10,37	6,666	8,038	11,95
**Upper 95% CI of mean**	10,07	20,51	42	12,93	12,11	13,15	10,48	11,54	19,17	21,21	14,86	16,49
**Sum**	100,7	154,8	30,25	200,3	46,79	83,2	86,04	32,15	310,2	97,58	80,14	184,8

PE 38:5

	Controls healthy	Controls Bronchitis	Diffuse development. disorder	Growth abn.+ defic. alveolar.	Immuno-intact host	Immuno-compromised host	Chronic tachypnea of infancy	Reactive lymphoid lesions	Related to alv. surf. region	Related to lung vessels/heart	Related to systemic disease	Unclear RDS in mature neonate
**Number of values**	11	10	2	18	5	8	9	4	21	7	7	13
**Minimum**	0,81	1,28	1,94	0,67	1,47	2,1	1,23	1,17	0,65	1,08	1,79	0,79
**25% Percentile**	1,97	1,475		1,54	1,7	2,135	1,405	1,415	1,25	1,51	1,92	1,88
**Median**	2,43	1,87	2,21	1,88	2,92	2,55	1,73	1,805	1,63	1,71	2,75	2,2
**75% Percentile**	2,74	2,49		2,72	3,565	3,155	2,435	2,285	1,975	1,84	3	3,19
**Maximum**	3,41	3,53	2,48	4,26	3,85	3,68	4,23	2,62	5,08	2,24	3,08	6,53
**Mean**	2,329	2,041	2,21	2,099	2,69	2,683	2,038	1,85	1,809	1,689	2,587	2,547
**Std. Deviation**	0,7594	0,677	0,3818	0,9287	0,9763	0,6393	0,9349	0,6059	0,9652	0,35	0,5239	1,472
**Std. Error**	0,229	0,2141	0,27	0,2189	0,4366	0,226	0,3116	0,303	0,2106	0,1323	0,198	0,4082

**Lower 95% CI of mean**	1,819	1,557	-1,221	1,637	1,478	2,148	1,319	0,8858	1,369	1,365	2,103	1,658
**Upper 95% CI of mean**	2,839	2,525	5,641	2,561	3,902	3,217	2,756	2,814	2,248	2,012	3,072	3,436
**Sum**	25,62	20,41	4,42	37,78	13,45	21,46	18,34	7,4	37,98	11,82	18,11	33,11

PE 38:4

	Controls healthy	Controls Bronchitis	Diffuse development. disorder	Growth abn.+ defic. alveolar.	Immuno-intact host	Immuno-compromised host	Chronic tachypnea of infancy	Reactive lymphoid lesions	Related to alv. surf. region	Related to lung vessels/heart	Related to systemic disease	Unclear RDS in mature neonate
**Number of values**	11	10	2	18	5	8	9	4	21	7	7	13
**Minimum**	1,16	2,73	4,66	1,2	4,44	3,5	1,81	2,86	1,59	1,76	4,47	4,06
**25% Percentile**	3,22	3,585		3,06	4,57	4,195	2,69	3,275	3,505	3,15	4,63	4,655
**Median**	4,55	4,29	5,06	4,455	5,27	5,215	3,19	4,02	4,82	4,53	5,51	4,98
**75% Percentile**	4,93	4,98		5,315	7,145	6,255	4,615	4,66	6,28	4,71	5,68	7,52
**Maximum**	6,07	11,38	5,46	8,39	8,31	6,74	6,23	4,97	9,81	7,55	10,73	13,41
**Mean**	4,1	4,84	5,06	4,443	5,74	5,196	3,626	3,968	4,99	4,253	5,921	6,562
**Std. Deviation**	1,339	2,412	0,5657	1,861	1,554	1,175	1,349	0,9046	1,943	1,796	2,182	3,105
**Std. Error**	0,4037	0,7628	0,4	0,4388	0,6948	0,4154	0,4496	0,4523	0,4239	0,6786	0,8245	0,8612
**Lower 95% CI of mean**	3,2	3,114	-0,02248	3,517	3,811	4,214	2,589	2,528	4,106	2,592	3,904	4,685
**Upper 95% CI of mean**	5	6,566	10,14	5,368	7,669	6,179	4,662	5,407	5,874	5,913	7,939	8,438
**Sum**	45,1	48,4	10,12	79,97	28,7	41,57	32,63	15,87	104,8	29,77	41,45	85,3

PE 38:3

	Controls healthy	Controls Bronchitis	Diffuse development. disorder	Growth abn.+ defic. alveolar.	Immuno-intact host	Immuno-compromised host	Chronic tachypnea of infancy	Reactive lymphoid lesions	Related to alv. surf. region	Related to lung vessels/heart	Related to systemic disease	Unclear RDS in mature neonate
**Number of values**	11	10	2	18	5	8	9	4	21	7	7	13
**Minimum**	0,53	0,63	1,1	0,61	0,91	0,74	0,61	0,49	0,52	0,71	0,65	0,96
**25% Percentile**	0,64	0,89		1,045	0,95	0,89	0,65	0,615	0,86	0,83	0,75	1,27
**Median**	0,9	1,17	1,795	1,27	1,01	1,075	0,71	0,81	1,34	1,03	1,39	1,57
**75% Percentile**	1,05	1,6		1,685	1,495	1,535	1,14	0,9	2,485	1,6	1,57	2,02
**Maximum**	1,72	1,75	2,49	2,58	1,5	2,74	1,45	0,92	3,54	2,53	2,65	2,45
**Mean**	0,9182	1,231	1,795	1,379	1,18	1,31	0,8633	0,7575	1,662	1,311	1,326	1,638
**Std. Deviation**	0,3479	0,3756	0,9829	0,5162	0,29	0,6652	0,3186	0,1943	0,9591	0,628	0,6854	0,4889
**Std. Error**	0,1049	0,1188	0,695	0,1217	0,1297	0,2352	0,1062	0,09716	0,2093	0,2374	0,2591	0,1356
**Lower 95% CI of mean**	0,6844	0,9623	-7,036	1,122	0,8199	0,7539	0,6184	0,4483	1,226	0,7306	0,6918	1,342
**Upper 95% CI of mean**	1,152	1,5	10,63	1,636	1,54	1,866	1,108	1,067	2,099	1,892	1,96	1,933
**Sum**	10,1	12,31	3,59	24,82	5,9	10,48	7,77	3,03	34,91	9,18	9,28	21,29

PE 38:2

	Controls healthy	Controls Bronchitis	Diffuse development. disorder	Growth abn.+ defic. alveolar.	Immuno-intact host	Immuno-compromised host	Chronic tachypnea of infancy	Reactive lymphoid lesions	Related to alv. surf. region	Related to lung vessels/heart	Related to systemic disease	Unclear RDS in mature neonate
**Number of values**	11	10	2	18	5	8	9	4	21	7	7	13
**Minimum**	0,33	0,42	1	0,38	0,54	0,42	0,39	0,32	0,37	0,46	0,39	0,47
**25% Percentile**	0,47	0,535		0,565	0,54	0,49	0,415	0,39	0,475	0,52	0,43	0,58
**Median**	0,54	0,73	1,01	0,705	0,57	0,645	0,6	0,48	0,83	0,7	0,48	0,72
**75% Percentile**	0,65	0,81		0,82	0,615	0,865	0,675	0,525	1,195	0,79	0,99	0,85
**Maximum**	0,93	0,84	1,02	1,2	0,64	1,18	0,72	0,55	1,67	1,42	1,14	0,99
**Mean**	0,5645	0,687	1,01	0,7111	0,576	0,7	0,5533	0,4575	0,8448	0,7371	0,6414	0,7215
**Std. Deviation**	0,156	0,1423	0,01414	0,1993	0,04159	0,257	0,1358	0,09878	0,384	0,3231	0,3014	0,1613
**Std. Error**	0,04703	0,045	0,01	0,04697	0,0186	0,09085	0,04528	0,04939	0,08379	0,1221	0,1139	0,04473
**Lower 95% CI of mean**	0,4598	0,5852	0,8829	0,612	0,5244	0,4852	0,4489	0,3003	0,67	0,4383	0,3627	0,6241
**Upper 95% CI of mean**	0,6693	0,7888	1,137	0,8102	0,6276	0,9148	0,6577	0,6147	1,02	1,036	0,9202	0,819
**Sum**	6,21	6,87	2,02	12,8	2,88	5,6	4,98	1,83	17,74	5,16	4,49	9,38

PE 40:6

	Controls healthy	Controls Bronchitis	Diffuse development. disorder	Growth abn.+ defic. alveolar.	Immuno-intact host	Immuno-compromised host	Chronic tachypnea of infancy	Reactive lymphoid lesions	Related to alv. surf. region	Related to lung vessels/heart	Related to systemic disease	Unclear RDS in mature neonate
**Number of values**	11	10	2	18	5	8	9	4	21	7	7	13
**Minimum**	0	0,41	1,82	0	0,6	0,53	0,33	0,64	0	0	0,48	0,68
**25% Percentile**	0,33	0,6		0,55	0,65	0,59	0,335	0,67	0,52	0,3	0,57	0,905
**Median**	0,7	0,7	2,225	0,92	0,79	0,8	0,57	0,775	0,93	0,48	0,81	1,41
**75% Percentile**	0,82	0,99		1,545	1,095	0,885	0,85	0,995	2,27	0,72	1,12	2,36
**Maximum**	0,93	1,45	2,63	3	1,11	1,38	1,62	1,14	4,53	1,25	1,32	4,44
**Mean**	0,5836	0,802	2,225	1,068	0,856	0,8075	0,6567	0,8325	1,435	0,55	0,8443	1,772
**Std. Deviation**	0,3104	0,2994	0,5728	0,7142	0,2285	0,2711	0,4221	0,2232	1,243	0,3972	0,3046	1,084
**Std. Error**	0,0936	0,09468	0,405	0,1683	0,1022	0,09586	0,1407	0,1116	0,2712	0,1501	0,1151	0,3005
**Lower 95% CI of mean**	0,3751	0,5878	-2,921	0,7126	0,5722	0,5808	0,3322	0,4773	0,869	0,1826	0,5626	1,118
**Upper 95% CI of mean**	0,7922	1,016	7,371	1,423	1,14	1,034	0,9811	1,188	2,001	0,9174	1,126	2,427
**Sum**	6,42	8,02	4,45	19,22	4,28	6,46	5,91	3,33	30,13	3,85	5,91	23,04

PE 40:5

	Controls healthy	Controls Bronchitis	Diffuse development. disorder	Growth abn.+ defic. alveolar.	Immuno-intact host	Immuno-compromised host	Chronic tachypnea of infancy	Reactive lymphoid lesions	Related to alv. surf. region	Related to lung vessels/heart	Related to systemic disease	Unclear RDS in mature neonate
**Number of values**	11	10	2	18	5	8	9	4	21	7	7	13
**Minimum**	0,18	0,46	1,53	0,26	0,73	0,5	0,28	0,45	0,28	0,27	0,52	0,6
**25% Percentile**	0,46	0,55		0,57	0,745	0,685	0,33	0,46	0,565	0,28	0,66	0,695
**Median**	0,61	0,675	1,78	0,875	0,81	1,02	0,44	0,545	0,97	0,65	1,15	0,88
**75% Percentile**	0,79	0,94		1,14	0,935	1,235	0,58	0,625	1,4	1,17	1,78	1,335
**Maximum**	1,01	1,19	2,03	1,55	1,06	1,64	0,75	0,63	2,3	1,19	2	2,63
**Mean**	0,6136	0,75	1,78	0,855	0,834	1,003	0,4633	0,5425	1,043	0,6914	1,184	1,085
**Std. Deviation**	0,2289	0,2317	0,3536	0,3694	0,1309	0,3831	0,1522	0,0957	0,5441	0,3745	0,5469	0,5538
**Std. Error**	0,06902	0,07327	0,25	0,08706	0,05853	0,1354	0,05074	0,04785	0,1187	0,1415	0,2067	0,1536
**Lower 95% CI of mean**	0,4598	0,5842	-1,397	0,6713	0,6715	0,6822	0,3463	0,3902	0,7957	0,3451	0,6785	0,7499
**Upper 95% CI of mean**	0,7674	0,9158	4,957	1,039	0,9965	1,323	0,5804	0,6948	1,291	1,038	1,69	1,419
**Sum**	6,75	7,5	3,56	15,39	4,17	8,02	4,17	2,17	21,91	4,84	8,29	14,1

PE 40:4

	Controls healthy	Controls Bronchitis	Diffuse development. disorder	Growth abn.+ defic. alveolar.	Immuno-intact host	Immuno-compromised host	Chronic tachypnea of infancy	Reactive lymphoid lesions	Related to alv. surf. region	Related to lung vessels/heart	Related to systemic disease	Unclear RDS in mature neonate
**Number of values**	11	10	2	18	5	8	9	4	21	7	7	13
**Minimum**	0,13	0,26	1,73	0,19	0,47	0,34	0,2	0,38	0,31	0,24	0,44	0,36
**25% Percentile**	0,22	0,43		0,655	0,49	0,585	0,285	0,42	0,66	0,4	0,74	0,815
**Median**	0,44	0,675	2,685	0,9	1,05	0,86	0,36	0,52	1,08	0,76	0,97	1,35
**75% Percentile**	0,59	1,06		1,325	1,08	1,355	0,545	0,67	2,105	1,24	1,57	1,545
**Maximum**	0,79	1,47	3,64	1,8	1,09	1,64	0,55	0,76	3,71	1,72	4,47	3,62
**Mean**	0,4373	0,763	2,685	0,9639	0,838	0,9475	0,3878	0,545	1,459	0,8186	1,49	1,388
**Std. Deviation**	0,2114	0,3746	1,351	0,46	0,3183	0,4791	0,1333	0,1652	1,018	0,5154	1,372	0,8376
**Std. Error**	0,06375	0,1185	0,955	0,1084	0,1424	0,1694	0,04443	0,08261	0,2221	0,1948	0,5184	0,2323
**Lower 95% CI of mean**	0,2952	0,495	-9,449	0,7351	0,4428	0,547	0,2853	0,2821	0,9953	0,3419	0,2214	0,8823
**Upper 95% CI of mean**	0,5793	1,031	14,82	1,193	1,233	1,348	0,4902	0,8079	1,922	1,295	2,759	1,895
**Sum**	4,81	7,63	5,37	17,35	4,19	7,58	3,49	2,18	30,63	5,73	10,43	18,05

PE P 16:0/16:1

	Controls healthy	Controls Bronchitis	Diffuse development. disorder	Growth abn. + defic. alveolar.	Immuno-intact host	Immuno-compromised host	Chronic tachypnea of infancy	Reactive lymphoid lesions	Related to alv. surf. region	Related to lung vessels/heart	Related to systemic disease	Unclear RDS in mature neonate
**Number of values**	11	10	2	18	5	8	8	4	17	7	7	13
**Minimum**	0,69	0,3	0,68	0,3	0,57	0,52	0,46	0,76	0,13	0,37	0,51	0,24
**25% Percentile**	0,83	0,45		0,535	0,725	0,615	0,55	0,835	0,455	0,59	0,52	0,475
**Median**	1,09	0,59	0,68	0,895	1,02	1	0,75	1,065	0,68	0,61	0,82	0,58
**75% Percentile**	1,18	1,18		1,705	1,315	1,275	1,11	1,67	1,55	0,79	1,3	0,845
**Maximum**	1,46	1,5	0,68	2,84	1,44	1,52	1,21	2,12	2,47	1,71	1,65	1,18
**Mean**	1,042	0,746	0,68	1,109	1,02	0,9775	0,8113	1,253	0,9653	0,7643	0,9171	0,6615
**Std. Deviation**	0,2481	0,4237	0	0,7134	0,3269	0,3687	0,2983	0,6092	0,6622	0,436	0,429	0,2843
**Std. Error**	0,07481	0,134	0	0,1681	0,1462	0,1304	0,1055	0,3046	0,1606	0,1648	0,1622	0,07884
**Lower 95% CI of mean**	0,8751	0,4429	0,68	0,7541	0,6141	0,6693	0,5618	0,2831	0,6248	0,3611	0,5204	0,4898
**Upper 95% CI of mean**	1,208	1,049	0,68	1,464	1,426	1,286	1,061	2,222	1,306	1,168	1,314	0,8333
**Sum**	11,46	7,46	1,36	19,96	5,1	7,82	6,49	5,01	16,41	5,35	6,42	8,6

PE P 16:0/16:0

	Controls healthy	Controls Bronchitis	Diffuse development. disorder	Growth abn. + defic. alveolar.	Immuno-intact host	Immuno-compromised host	Chronic tachypnea of infancy	Reactive lymphoid lesions	Related to alv. surf. region	Related to lung vessels/heart	Related to systemic disease	Unclear RDS in mature neonate
**Number of values**	11	10	2	18	5	8	8	4	17	7	7	13
**Minimum**	0,51	0,47	1,05	0,62	0,56	1,29	0,8	0,95	0,12	0,7	0,81	0,74
**25% Percentile**	0,87	0,56		0,905	0,87	1,345	0,88	1,215	0,815	1,16	1,14	0,94
**Median**	1,02	1,065	1,155	1,615	1,39	1,92	1,07	1,53	1,15	1,34	1,22	1,1
**75% Percentile**	1,41	1,58		1,93	1,58	2,425	1,27	1,82	1,755	1,56	1,41	1,33
**Maximum**	4,01	4,53	1,26	2,88	1,71	3,85	1,51	2,06	5,08	1,85	1,5	2,1
**Mean**	1,331	1,353	1,155	1,559	1,258	2,065	1,094	1,518	1,394	1,353	1,217	1,17
**Std. Deviation**	0,9412	1,204	0,1485	0,6526	0,4336	0,8708	0,2526	0,4552	1,124	0,3639	0,2222	0,3754
**Std. Error**	0,2838	0,3806	0,105	0,1538	0,1939	0,3079	0,0893	0,2276	0,2725	0,1375	0,08397	0,1041
**Lower 95% CI of mean**	0,6986	0,492	-0,1791	1,234	0,7197	1,337	0,8826	0,7931	0,8159	1,016	1,012	0,9432
**Upper 95% CI of mean**	1,963	2,214	2,489	1,883	1,796	2,793	1,305	2,242	1,971	1,689	1,423	1,397
**Sum**	14,64	13,53	2,31	28,06	6,29	16,52	8,75	6,07	23,69	9,47	8,52	15,21

PE P 16:0/18:2

	Controls healthy	Controls Bronchitis	Diffuse development. disorder	Growth abn. + defic. alveolar.	Immuno-intact host	Immuno-compromised host	Chronic tachypnea of infancy	Reactive lymphoid lesions	Related to alv. surf. region	Related to lung vessels/heart	Related to systemic disease	Unclear RDS in mature neonate
**Number of values**	11	11	2	18	5	8	8	4	17	7	7	13
**Minimum**	1,47	1,65	1,17	1,37	1,95	1,37	2,16	2,18	1,03	1,47	1,85	1,19
**25% Percentile**	2,15	2,05		2,405	1,975	2,54	3,19	2,42	2,065	2,01	2,22	1,875
**Median**	3,19	2,63	1,455	3,13	2,41	3,69	4,57	3,01	3,18	2,51	2,55	2,41
**75% Percentile**	3,77	3,54		3,5	2,945	4,425	5,37	3,555	3,915	3,63	2,85	3,545
**Maximum**	5,69	4,05	1,74	5,27	3,07	5,53	5,66	3,75	6,65	7,8	3,5	5,64
**Mean**	3,143	2,807	1,455	3,078	2,45	3,526	4,26	2,988	3,068	3,343	2,58	2,69
**Std. Deviation**	1,15	0,8284	0,4031	1,015	0,4938	1,389	1,277	0,7023	1,326	2,109	0,5208	1,28
**Std. Error**	0,3466	0,2498	0,285	0,2392	0,2208	0,4912	0,4516	0,3511	0,3216	0,7969	0,1968	0,3551
**Lower 95% CI of mean**	2,37	2,251	-2,166	2,574	1,837	2,365	3,192	1,87	2,386	1,393	2,098	1,916
**Upper 95% CI of mean**	3,915	3,364	5,076	3,583	3,063	4,688	5,328	4,105	3,749	5,293	3,062	3,464
**Sum**	34,57	30,88	2,91	55,41	12,25	28,21	34,08	11,95	52,15	23,4	18,06	34,97

PE P 16:0/18:1

	Controls healthy	Controls Bronchitis	Diffuse development. disorder	Growth abn. + defic. alveolar.	Immuno-intact host	Immuno-compromised host	Chronic tachypnea of infancy	Reactive lymphoid lesions	Related to alv. surf. region	Related to lung vessels/heart	Related to systemic disease	Unclear RDS in mature neonate
**Number of values**	11	10	2	18	5	8	8	4	17	7	7	13
**Minimum**	3,95	8,24	9,59	2,8	4,62	5,58	6,5	4,58	2,74	5,6	5,72	4,77
**25% Percentile**	4,71	8,72		7,33	5,07	7,02	7,9	5,095	5,69	6,6	6,03	7,795
**Median**	5,87	10,03	10,51	10,34	5,64	7,58	8,61	5,92	10,93	8,6	6,84	12,89
**75% Percentile**	9,75	13		12,6	8,625	9,145	9,555	6,95	15,87	11,98	9,03	14,15
**Maximum**	10,2	16,75	11,42	15,92	11,3	10,47	10,8	7,67	17,64	20,75	12,77	16,76
**Mean**	6,835	10,83	10,51	10,04	6,606	7,943	8,679	6,023	10,69	10,38	7,751	11,6
**Std. Deviation**	2,245	2,729	1,294	3,413	2,67	1,696	1,328	1,292	5,302	5,066	2,502	3,771
**Std. Error**	0,677	0,8629	0,915	0,8044	1,194	0,5995	0,4694	0,646	1,286	1,915	0,9458	1,046
**Lower 95% CI of mean**	5,327	8,875	-1,121	8,342	3,29	6,525	7,569	3,967	7,96	5,693	5,437	9,324
**Upper 95% CI of mean**	8,344	12,78	22,13	11,74	9,922	9,36	9,789	8,078	13,41	15,06	10,07	13,88
**Sum**	75,19	108,3	21,01	180,7	33,03	63,54	69,43	24,09	181,7	72,65	54,26	150,8

PE P 16:0/20:4

	Controls healthy	Controls Bronchitis	Diffuse development. disorder	Growth abn. + defic. alveolar.	Immuno-intact host	Immuno-compromised host	Chronic tachypnea of infancy	Reactive lymphoid lesions	Related to alv. surf. region	Related to lung vessels/heart	Related to systemic disease	Unclear RDS in mature neonate
**Number of values**	11	10	2	18	5	8	8	4	17	7	7	13

	Controls healthy	Controls Bronchitis	Diffuse development. disorder	Growth abn. + defic. alveolar.	Immuno-intact host	Immuno-compromised host	Chronic tachypnea of infancy	Reactive lymphoid lesions	Related to alv. surf. region	Related to lung vessels/heart	Related to systemic disease	Unclear RDS in mature neonate
**Minimum**	6,13	8,66	16,2	3,84	11,47	16,64	5,79	10,46	5,02	7,4	12,19	12,9
**25% Percentile**	10,54	9,495		8,685	11,6	19,16	10,48	12,62	11,96	9,88	13,28	14,82
**Median**	11,79	12,35	17,7	14,58	12,49	21,83	12,21	16,31	12,54	13,98	21,22	15,97
**75% Percentile**	14,83	16,88		20,01	19,64	25,1	16,04	20,17	15,13	16,76	23,63	21,66
**Maximum**	19,07	24,98	19,19	29,55	21,24	29,72	20,81	22,48	36,94	28,88	24,71	24,06
**Mean**	12,49	13,7	17,7	14,83	14,99	22,32	13,01	16,39	14,34	14,69	19,05	17,51
**Std. Deviation**	3,51	4,943	2,114	6,603	4,407	4,316	4,812	5,067	6,915	7,033	5,441	3,707
**Std. Error**	1,058	1,563	1,495	1,556	1,971	1,526	1,701	2,533	1,677	2,658	2,056	1,028
**Lower 95% CI of mean**	10,13	10,16	-1,301	11,54	9,52	18,71	8,984	8,328	10,79	8,189	14,02	15,27
**Upper 95% CI of mean**	14,85	17,23	36,69	18,11	20,46	25,92	17,03	24,45	17,9	21,2	24,09	19,75
**Sum**	137,4	137	35,39	266,9	74,96	178,5	104,1	65,56	243,8	102,9	133,4	227,6

PE P 16:0/20:3

	Controls healthy	Controls Bronchitis	Diffuse development. disorder	Growth abn. + defic. alveolar.	Immuno-intact host	Immuno-compromised host	Chronic tachypnea of infancy	Reactive lymphoid lesions	Related to alv. surf. region	Related to lung vessels/heart	Related to systemic disease	Unclear RDS in mature neonate
**Number of values**	11	10	2	18	5	8	8	4	17	7	7	13
**Minimum**	0,95	1,08	1,16	1,63	1,52	1,8	1,25	1,25	1,04	0,85	1,6	1,26
**25% Percentile**	1,33	1,285		1,79	1,58	2,28	1,39	1,255	1,26	1,53	1,75	1,48
**Median**	1,65	1,565	1,82	2,065	1,66	2,405	1,535	1,39	1,76	2,27	2,06	1,85
**75% Percentile**	2,34	1,93		2,85	2,055	2,575	1,715	1,565	2,14	2,42	2,79	2,675
**Maximum**	2,89	2,74	2,48	3,51	2,19	2,81	2,87	1,61	5,82	2,89	4,61	3,1
**Mean**	1,77	1,652	1,82	2,25	1,786	2,391	1,675	1,41	1,969	2,024	2,506	2,032
**Std. Deviation**	0,5652	0,4856	0,9334	0,577	0,2688	0,3061	0,5143	0,1828	1,113	0,6702	1,033	0,6357
**Std. Error**	0,1704	0,1536	0,66	0,136	0,1202	0,1082	0,1818	0,09138	0,2699	0,2533	0,3905	0,1763
**Lower 95% CI of mean**	1,39	1,305	-6,566	1,963	1,452	2,135	1,245	1,119	1,397	1,404	1,55	1,647
**Upper 95% CI of mean**	2,15	1,999	10,21	2,537	2,12	2,647	2,105	1,701	2,541	2,644	3,461	2,416
**Sum**	19,47	16,52	3,64	40,5	8,93	19,13	13,4	5,64	33,47	14,17	17,54	26,41

PE P 16:0/22:6

	Controls healthy	Controls Bronchitis	Diffuse development. disorder	Growth abn. + defic. alveolar.	Immuno-intact host	Immuno-compromised host	Chronic tachypnea of infancy	Reactive lymphoid lesions	Related to alv. surf. region	Related to lung vessels/heart	Related to systemic disease	Unclear RDS in mature neonate
**Number of values**	11	10	2	18	5	8	8	4	17	7	7	13
**Minimum**	1,23	1,74	7,4	1,46	2,36	1,61	1,43	2,18	1,59	1,25	1,55	3,24
**25% Percentile**	2,01	1,97		2,51	3,04	2,63	1,92	2,59	2,68	2,09	1,82	3,73
**Median**	2,61	2,585	7,58	4,6	3,74	3,725	2,855	3,17	5,06	2,86	2,61	6,77
**75% Percentile**	3,57	3,415		6,83	4,355	4,065	4,445	3,62	7,62	4,86	3,05	8,405
**Maximum**	3,85	4,68	7,76	12,68	4,72	5,06	5,66	3,9	13,17	4,93	3,25	11,61
**Mean**	2,623	2,767	7,58	5,089	3,706	3,439	3,191	3,105	5,381	3,107	2,489	6,461
**Std. Deviation**	0,9248	0,9028	0,2546	3,02	0,8547	1,087	1,56	0,7197	3,268	1,357	0,6147	2,627
**Std. Error**	0,2788	0,2855	0,18	0,7118	0,3822	0,3842	0,5515	0,3598	0,7925	0,513	0,2323	0,7286
**Lower 95% CI of mean**	2,001	2,121	5,293	3,587	2,645	2,53	1,887	1,96	3,701	1,852	1,92	4,873
**Upper 95% CI of mean**	3,244	3,413	9,867	6,591	4,767	4,347	4,495	4,25	7,061	4,362	3,057	8,048
**Sum**	28,85	27,67	15,16	91,6	18,53	27,51	25,53	12,42	91,48	21,75	17,42	83,99

PE P 16:0/22:5

	Controls healthy	Controls Bronchitis	Diffuse development. disorder	Growth abn. + defic. alveolar.	Immuno-intact host	Immuno-compromised host	Chronic tachypnea of infancy	Reactive lymphoid lesions	Related to alv. surf. region	Related to lung vessels/heart	Related to systemic disease	Unclear RDS in mature neonate
**Number of values**	11	10	2	18	5	8	8	4	17	7	7	13
**Minimum**	1,11	1,81	1,82	1,93	2,43	2,56	1,44	1,9	1,41	0,99	2,32	1,58
**25% Percentile**	1,67	1,94		2,125	2,635	3,105	1,665	2,165	2,195	1,35	2,7	2,315
**Median**	1,83	2,43	2,96	2,47	3,63	3,395	1,745	2,48	2,86	2,43	3,13	2,54
**75% Percentile**	2,54	3,145		3,525	4,16	3,8	2,14	2,705	3,24	3,03	5,4	2,875
**Maximum**	3,38	3,81	4,1	4,12	4,56	4,2	2,51	2,88	7,41	4,45	5,46	3,91
**Mean**	2,028	2,543	2,96	2,784	3,444	3,42	1,881	2,435	3	2,396	3,627	2,63
**Std. Deviation**	0,6407	0,6637	1,612	0,7708	0,8326	0,5292	0,3656	0,4055	1,345	1,173	1,299	0,611
**Std. Error**	0,1932	0,2099	1,14	0,1817	0,3724	0,1871	0,1292	0,2028	0,3263	0,4432	0,4909	0,1695
**Lower 95% CI of mean**	1,598	2,068	-11,53	2,401	2,41	2,978	1,576	1,79	2,308	1,311	2,426	2,261
**Upper 95% CI of mean**	2,459	3,018	17,45	3,168	4,478	3,862	2,187	3,08	3,692	3,48	4,828	2,999
**Sum**	22,31	25,43	5,92	50,12	17,22	27,36	15,05	9,74	51	16,77	25,39	34,19

PE P 16:0/22:4

	Controls healthy	Controls Bronchitis	Diffuse development. disorder	Growth abn. + defic. alveolar.	Immuno-intact host	Immuno-compromised host	Chronic tachypnea of infancy	Reactive lymphoid lesions	Related to alv. surf. region	Related to lung vessels/heart	Related to systemic disease	Unclear RDS in mature neonate
**Number of values**	11	10	2	18	5	8	8	4	17	7	7	13
**Minimum**	1,02	1,92	3,91	2,26	2,39	2,78	2,1	2,84	1,37	2,05	2,72	2,56
**25% Percentile**	2,58	2,6		3,03	2,915	3,39	2,375	3,365	2,665	2,07	4,28	3,985
**Median**	2,92	2,94	7,22	3,625	3,71	4,595	3,11	4,115	4,02	3,5	5,23	4,28
**75% Percentile**	3,87	4,465		4,91	4,785	4,855	3,715	4,39	4,775	5,17	5,92	5,715

**Maximum**	4,53	5,39	10,53	5,74	5,02	6,1	6,2	4,44	7,98	6,48	11,51	11,3
**Mean**	2,964	3,303	7,22	3,903	3,822	4,32	3,338	3,878	4,01	3,786	5,726	5,259
**Std. Deviation**	1,066	1,169	4,681	1,015	1,022	1,072	1,318	0,7319	1,808	1,668	2,779	2,422
**Std. Error**	0,3215	0,3696	3,31	0,2393	0,4569	0,3791	0,4661	0,3659	0,4386	0,6306	1,051	0,6717
**Lower 95% CI of mean**	2,247	2,467	-34,84	3,398	2,554	3,424	2,235	2,713	3,08	2,243	3,155	3,796
**Upper 95% CI of mean**	3,68	4,139	49,28	4,408	5,09	5,216	4,44	5,042	4,94	5,329	8,296	6,723
**Sum**	32,6	33,03	14,44	70,25	19,11	34,56	26,7	15,51	68,17	26,5	40,08	68,37

PE P 18:1/16:0

	**Controls healthy**	**Controls Bronchitis**	**Diffuse development. disorder**	**Growth abn. + defic. alveolar.**	**Immuno-intact host**	**Immuno-compromised host**	**Chronic tachypnea of infancy**	**Reactive lymphoid lesions**	**Related to alv. surf. region**	**Related to lung vessels/heart**	**Related to systemic disease**	**Unclear RDS in mature neonate**
**Number of values**	11	10	2	18	5	8	8	4	17	7	7	13
**Minimum**	0,9	0,49	1,41	1,04	0,78	1,05	0,95	0,82	0,33	1,18	0,83	0,87
**25% Percentile**	1,14	0,72		1,155	0,82	1,275	1,195	1,145	0,85	1,27	0,94	0,99
**Median**	1,27	0,92	1,465	1,52	1,21	2,395	1,37	1,835	1,16	1,42	1,09	1,35
**75% Percentile**	1,73	1,59		2,12	1,795	2,565	1,545	2,365	1,77	2,01	1,74	1,965
**Maximum**	9,62	2,38	1,52	3,75	1,96	3,35	2,17	2,53	3,33	8,12	2,3	2,4
**Mean**	2,085	1,131	1,465	1,728	1,288	2,109	1,418	1,755	1,422	2,436	1,306	1,502
**Std. Deviation**	2,516	0,5802	0,07778	0,7429	0,504	0,8146	0,3683	0,7647	0,8453	2,522	0,5254	0,5392
**Std. Error**	0,7586	0,1835	0,055	0,1751	0,2254	0,288	0,1302	0,3823	0,205	0,9532	0,1986	0,1495
**Lower 95% CI of mean**	0,3943	0,7159	0,7662	1,359	0,6623	1,428	1,11	0,5383	0,9877	0,1033	0,8198	1,176
**Upper 95% CI of mean**	3,775	1,546	2,164	2,098	1,914	2,79	1,725	2,972	1,857	4,768	1,792	1,827
**Sum**	22,93	11,31	2,93	31,11	6,44	16,87	11,34	7,02	24,18	17,05	9,14	19,52

PE P 18:1/18:2

	**Controls healthy**	**Controls Bronchitis**	**Diffuse development. disorder**	**Growth abn. + defic. alveolar.**	**Immuno-intact host**	**Immuno-compromised host**	**Chronic tachypnea of infancy**	**Reactive lymphoid lesions**	**Related to alv. surf. region**	**Related to lung vessels/heart**	**Related to systemic disease**	**Unclear RDS in mature neonate**
**Number of values**	11	10	2	18	5	8	8	4	17	7	7	13
**Minimum**	0,92	0,46	0,51	0,48	1	0,7	1,15	1,14	0,3	0,4	0,75	0,45
**25% Percentile**	1,44	0,83		0,88	1	0,96	1,335	1,42	0,55	0,48	0,76	0,53
**Median**	1,68	1,305	0,645	1,305	1,04	1,305	1,875	1,88	1,05	1,54	0,77	0,67
**75% Percentile**	2,35	1,66		1,805	1,88	2,02	2,755	2,405	1,925	4,29	1,28	1,65
**Maximum**	4,61	1,94	0,78	3,48	1,89	2,57	3,34	2,75	4,8	6,36	1,56	2,22
**Mean**	1,972	1,244	0,645	1,425	1,36	1,48	2,053	1,913	1,344	2,224	1,016	1,051
**Std. Deviation**	0,9948	0,4685	0,1909	0,7743	0,475	0,6685	0,8244	0,6746	1,118	2,247	0,3351	0,6433
**Std. Error**	0,2999	0,1481	0,135	0,1825	0,2124	0,2363	0,2915	0,3373	0,2711	0,8494	0,1267	0,1784
**Lower 95% CI of mean**	1,304	0,9089	-1,07	1,04	0,7702	0,9211	1,363	0,8391	0,7689	0,1459	0,7058	0,662
**Upper 95% CI of mean**	2,64	1,579	2,36	1,81	1,95	2,039	2,742	2,986	1,918	4,303	1,326	1,44
**Sum**	21,69	12,44	1,29	25,65	6,8	11,84	16,42	7,65	22,84	15,57	7,11	13,66

PE P 18:1/18:1

	**Controls healthy**	**Controls Bronchitis**	**Diffuse development. disorder**	**Growth abn. + defic. alveolar.**	**Immuno-intact host**	**Immuno-compromised host**	**Chronic tachypnea of infancy**	**Reactive lymphoid lesions**	**Related to alv. surf. region**	**Related to lung vessels/heart**	**Related to systemic disease**	**Unclear RDS in mature neonate**
**Number of values**	11	10	2	18	5	8	8	4	17	7	7	13
**Minimum**	2,19	1,91	1,92	1,56	1,26	1,38	2,09	1,48	0,54	1,55	1,38	1,27
**25% Percentile**	2,74	2,41		2,065	1,86	1,865	2,545	1,94	2,12	1,99	1,52	2,03
**Median**	3,14	2,965	2,325	2,595	2,47	2,5	2,87	2,545	2,7	2,91	2,41	2,55
**75% Percentile**	3,63	3,19		3,285	2,91	2,725	4,245	2,845	3,1	4,07	2,48	2,95
**Maximum**	8,04	5,04	2,73	5,36	2,96	4,08	5,9	3	4,29	9,17	2,9	3,67
**Mean**	3,61	2,996	2,325	2,806	2,402	2,455	3,414	2,393	2,603	3,763	2,2	2,494
**Std. Deviation**	1,611	0,828	0,5728	0,9507	0,677	0,8172	1,34	0,6558	0,9264	2,561	0,5492	0,6317
**Std. Error**	0,4858	0,2618	0,405	0,2241	0,3028	0,2889	0,4738	0,3279	0,2247	0,9681	0,2076	0,1752
**Lower 95% CI of mean**	2,528	2,404	-2,821	2,333	1,561	1,772	2,293	1,349	2,127	1,394	1,692	2,112
**Upper 95% CI of mean**	4,692	3,588	7,471	3,278	3,243	3,138	4,534	3,436	3,079	6,132	2,708	2,876
**Sum**	39,71	29,96	4,65	50,5	12,01	19,64	27,31	9,57	44,25	26,34	15,4	32,42

PE P 18:1/20:5

	**Controls healthy**	**Controls Bronchitis**	**Diffuse development. disorder**	**Growth abn. + defic. alveolar.**	**Immuno-intact host**	**Immuno-compromised host**	**Chronic tachypnea of infancy**	**Reactive lymphoid lesions**	**Related to alv. surf. region**	**Related to lung vessels/heart**	**Related to systemic disease**	**Unclear RDS in mature neonate**
**Number of values**	11	10	2	18	5	8	8	4	17	7	7	13
**Minimum**	0,64	0,21	0,31	0,29	0,41	0,26	0,36	0,75	0,08	0,26	0,25	0,18
**25% Percentile**	0,75	0,265		0,43	0,61	0,465	0,495	0,835	0,295	0,29	0,42	0,3
**Median**	1,12	0,52	0,54	0,735	0,83	0,62	0,915	0,995	0,5	0,71	0,56	0,61
**75% Percentile**	1,26	0,94		1,415	1,135	0,775	1,025	1,415	1,46	1,23	0,87	0,725
**Maximum**	1,6	1,08	0,77	3,33	1,16	1,46	1,2	1,76	2,46	1,89	1,25	0,86
**Mean**	1,071	0,579	0,54	1,068	0,864	0,68	0,8038	1,125	0,8788	0,8071	0,6571	0,5269
**Std. Deviation**	0,3004	0,3239	0,3253	0,8663	0,2993	0,364	0,3135	0,4431	0,7622	0,5781	0,3334	0,2234
**Std. Error**	0,09057	0,1024	0,23	0,2042	0,1339	0,1287	0,1109	0,2215	0,1849	0,2185	0,126	0,06197

**Lower 95% CI of mean**	0,8691	0,3473	-2,382	0,6375	0,4924	0,3757	0,5416	0,42	0,487	0,2725	0,3488	0,3919
**Upper 95% CI of mean**	1,273	0,8107	3,462	1,499	1,236	0,9843	1,066	1,83	1,271	1,342	0,9654	0,6619
**Sum**	11,78	5,79	1,08	19,23	4,32	5,44	6,43	4,5	14,94	5,65	4,6	6,85

PE P 18:1/20:4

	Controls healthy	Controls Bronchitis	Diffuse development. disorder	Growth abn. + defic. alveolar.	Immuno-intact host	Immuno-compromised host	Chronic tachypnea of infancy	Reactive lymphoid lesions	Related to alv. surf. region	Related to lung vessels/heart	Related to systemic disease	Unclear RDS in mature neonate
**Number of values**	11	10	2	18	5	8	8	4	17	7	7	13
**Minimum**	4,68	2,41	3,85	2,43	2,42	5,37	2,96	3,32	1,58	2,64	2,84	1,37
**25% Percentile**	5,19	3,235		2,845	4,03	6,115	3,425	4,29	2,93	3,37	4,29	2,765
**Median**	5,88	4,15	4,28	4,245	6,04	7,07	5,175	6,175	3,8	3,75	5,32	5,1
**75% Percentile**	7,05	5,235		6,28	6,37	7,935	6,18	7,58	4,89	6,22	6,8	6,135
**Maximum**	9,44	8,53	4,71	7,25	6,43	9,67	10,04	8,07	8,29	10,68	7,42	11,03
**Mean**	6,255	4,533	4,28	4,507	5,368	7,16	5,32	5,935	4,181	5,191	5,324	4,879
**Std. Deviation**	1,382	1,714	0,6081	1,605	1,676	1,409	2,323	2,097	1,857	2,77	1,609	2,476
**Std. Error**	0,4167	0,5419	0,43	0,3782	0,7494	0,4981	0,8212	1,048	0,4503	1,047	0,6083	0,6867
**Lower 95% CI of mean**	5,326	3,307	-1,184	3,709	3,287	5,982	3,378	2,599	3,227	2,63	3,836	3,383
**Upper 95% CI of mean**	7,183	5,759	9,744	5,305	7,449	8,338	7,262	9,271	5,136	7,753	6,813	6,376
**Sum**	68,8	45,33	8,56	81,12	26,84	57,28	42,56	23,74	71,08	36,34	37,27	63,43

PE P 18:1/22:6

	Controls healthy	Controls Bronchitis	Diffuse development. disorder	Growth abn. + defic. alveolar.	Immuno-intact host	Immuno-compromised host	Chronic tachypnea of infancy	Reactive lymphoid lesions	Related to alv. surf. region	Related to lung vessels/heart	Related to systemic disease	Unclear RDS in mature neonate
**Number of values**	11	10	2	18	5	8	8	4	17	7	7	12
**Minimum**	0,77	0,31	1,62	0,64	0,95	0,62	0,54	1,04	0,27	0,22	0,55	0,99
**25% Percentile**	1,19	0,595		0,89	1,075	0,68	0,835	1,1	1,185	0,82	0,64	1,09
**Median**	1,29	0,965	1,68	1,31	1,3	0,85	1,2	1,4	1,62	1,19	0,72	1,36
**75% Percentile**	1,47	1,05		1,805	1,38	1,38	1,32	1,76	2,095	1,33	1,16	1,655
**Maximum**	1,87	1,7	1,74	3,21	1,46	1,53	1,86	1,88	2,59	1,86	1,62	1,98
**Mean**	1,308	0,903	1,68	1,461	1,242	0,9963	1,139	1,43	1,562	1,099	0,8957	1,395
**Std. Deviation**	0,2938	0,3799	0,08485	0,6742	0,1879	0,3728	0,4255	0,3965	0,7067	0,5103	0,3761	0,3483
**Std. Error**	0,08859	0,1201	0,06	0,1589	0,08405	0,1318	0,1504	0,1982	0,1714	0,1929	0,1422	0,1006
**Lower 95% CI of mean**	1,111	0,6313	0,9176	1,126	1,009	0,6846	0,783	0,7991	1,198	0,6266	0,5479	1,174
**Upper 95% CI of mean**	1,506	1,175	2,442	1,796	1,475	1,308	1,495	2,061	1,925	1,571	1,244	1,616
**Sum**	14,39	9,03	3,36	26,3	6,21	7,97	9,11	5,72	26,55	7,69	6,27	16,74

PE P 18:0/16:0

	Controls healthy	Controls Bronchitis	Diffuse development. disorder	Growth abn. + defic. alveolar.	Immuno-intact host	Immuno-compromised host	Chronic tachypnea of infancy	Reactive lymphoid lesions	Related to alv. surf. region	Related to lung vessels/heart	Related to systemic disease	Unclear RDS in mature neonate
**Number of values**	11	10	2	18	5	8	8	4	17	7	7	13
**Minimum**	1,15	0,7	0,72	0,61	0,83	0,56	0,72	0,82	0,2	0,73	0,78	0,56
**25% Percentile**	1,46	0,845		0,985	1,025	1,01	1,47	0,895	0,465	0,95	0,86	0,71
**Median**	1,6	1,12	0,825	1,15	1,37	1,185	1,765	1,64	1,19	1,25	0,94	0,95
**75% Percentile**	1,95	1,765		1,99	1,95	1,81	2,155	2,485	1,525	2,34	1,29	1,355
**Maximum**	5	3	0,93	2,89	2,46	2,68	2,2	2,66	5,15	3,52	1,76	1,74
**Mean**	1,968	1,358	0,825	1,449	1,464	1,406	1,713	1,69	1,415	1,673	1,116	1,02
**Std. Deviation**	1,053	0,6991	0,1485	0,6272	0,6048	0,6633	0,5188	0,9311	1,434	0,9794	0,3418	0,3831
**Std. Error**	0,3175	0,2211	0,105	0,1478	0,2705	0,2345	0,1834	0,4655	0,3477	0,3702	0,1292	0,1063
**Lower 95% CI of mean**	1,261	0,8579	-0,5091	1,137	0,7131	0,8517	1,279	0,2085	0,6782	0,767	0,7996	0,7885
**Upper 95% CI of mean**	2,676	1,858	2,159	1,761	2,215	1,961	2,146	3,172	2,152	2,579	1,432	1,252
**Sum**	21,65	13,58	1,65	26,08	7,32	11,25	13,7	6,76	24,06	11,71	7,81	13,26

PE P 18:0/18:2

	Controls healthy	Controls Bronchitis	Diffuse development. disorder	Growth abn. + defic. alveolar.	Immuno-intact host	Immuno-compromised host	Chronic tachypnea of infancy	Reactive lymphoid lesions	Related to alv. surf. region	Related to lung vessels/heart	Related to systemic disease	Unclear RDS in mature neonate
**Number of values**	11	10	2	18	5	8	8	4	17	7	7	13
**Minimum**	2,36	2,29	1,06	1,21	2,41	1,07	3,11	2,6	0,5	1,38	1,53	1,09
**25% Percentile**	2,89	3,25		2,235	2,545	1,66	4	2,72	1,91	1,67	1,82	1,68
**Median**	4,79	3,925	1,22	2,59	2,99	2,715	5,895	3,325	3,18	2,8	2,32	1,98
**75% Percentile**	6,08	6,06		3,395	4,92	2,92	8,57	4,64	3,94	5,93	3,24	2,965
**Maximum**	8,31	6,13	1,38	5,36	5,59	3,57	9,79	5,47	9,07	7,93	4,07	4,63
**Mean**	4,829	4,442	1,22	2,834	3,584	2,404	6,229	3,68	3,515	3,863	2,533	2,388
**Std. Deviation**	1,991	1,446	0,2263	1,029	1,324	0,8452	2,6	1,303	2,357	2,417	0,8686	1,128
**Std. Error**	0,6004	0,4572	0,16	0,2425	0,5923	0,2988	0,9193	0,6515	0,5718	0,9136	0,3283	0,3127
**Lower 95% CI of mean**	3,491	3,408	-0,813	2,323	1,94	1,697	4,055	1,607	2,303	1,627	1,73	1,707
**Upper 95% CI of mean**	6,167	5,476	3,253	3,346	5,228	3,11	8,403	5,753	4,727	6,098	3,336	3,07
**Sum**	53,12	44,42	2,44	51,02	17,92	19,23	49,83	14,72	59,76	27,04	17,73	31,05

PE P 18:0/18:1

	Controls healthy	Controls Bronchitis	Diffuse development. disorder	Growth abn. + defic. alveolar.	Immuno-intact host	Immuno-compromised host	Chronic tachypnea of infancy	Reactive lymphoid lesions	Related to alv. surf. region	Related to lung vessels/heart	Related to systemic disease	Unclear RDS in mature neonate
**Number of values**	11	10	2	18	5	8	8	4	17	7	7	13
**Minimum**	4,2	5,44	4,13	2,99	3,44	3,04	4,48	3,76	1,63	3,97	3,29	2,66
**25% Percentile**	5	5,865		3,985	3,87	3,455	5,905	4,31	3,76	4,77	3,69	4,16
**Median**	7,27	9,85	4,94	5,385	6,26	3,98	9,505	5,27	6,05	7,03	5,16	6,13
**75% Percentile**	8,71	12,68		6,855	8,41	5,13	9,985	5,85	7,53	8,16	7,08	6,855
**Maximum**	9,58	15,65	5,75	10,04	9,37	6,31	11,52	6,02	17,89	8,31	7,77	8,32
**Mean**	7,016	9,581	4,94	5,669	6,164	4,31	8,349	5,08	6,149	6,58	5,316	5,558
**Std. Deviation**	1,829	3,555	1,146	1,918	2,389	1,152	2,541	1,006	3,707	1,635	1,736	1,872
**Std. Error**	0,5514	1,124	0,81	0,4522	1,069	0,4072	0,8985	0,5029	0,8991	0,6179	0,656	0,5192
**Lower 95% CI of mean**	5,788	7,038	-5,352	4,715	3,197	3,347	6,224	3,48	4,243	5,068	3,711	4,427
**Upper 95% CI of mean**	8,245	12,12	15,23	6,623	9,131	5,273	10,47	6,68	8,055	8,092	6,921	6,689
**Sum**	77,18	95,81	9,88	102,1	30,82	34,48	66,79	20,32	104,5	46,06	37,21	72,25

PE P 18:0/20:4

	Controls healthy	Controls Bronchitis	Diffuse development. disorder	Growth abn. + defic. alveolar.	Immuno-intact host	Immuno-compromised host	Chronic tachypnea of infancy	Reactive lymphoid lesions	Related to alv. surf. region	Related to lung vessels/heart	Related to systemic disease	Unclear RDS in mature neonate
**Number of values**	11	10	2	18	5	8	8	4	17	7	7	13
**Minimum**	5,93	11,03	8,42	3,75	13,43	8,42	7,51	6,66	3,93	8,71	9,31	9,91
**25% Percentile**	11,37	14,02		8,345	13,93	10,8	12,22	10,55	8,95	10,48	12,99	10,95
**Median**	16,3	15,75	11,39	10,33	15,69	11,88	13,65	14,99	11,66	12,86	16,52	11,69
**75% Percentile**	18,58	18,9		12,45	16,02	12,27	14,89	17	13,55	15,59	19,34	12,7
**Maximum**	20,61	23,16	14,35	14,05	16,1	13,69	17,41	18,45	17,79	16,88	19,59	17,08
**Mean**	14,65	16,32	11,39	10,08	15,12	11,5	13,3	13,77	11,25	12,83	15,45	12,27
**Std. Deviation**	4,89	3,422	4,193	2,857	1,151	1,555	2,917	5,034	3,397	2,899	3,793	2,137
**Std. Error**	1,474	1,082	2,965	0,6734	0,515	0,5499	1,031	2,517	0,8238	1,096	1,434	0,5928
**Lower 95% CI of mean**	11,37	13,88	-26,29	8,656	13,69	10,2	10,86	5,762	9,499	10,15	11,94	10,97
**Upper 95% CI of mean**	17,94	18,77	49,06	11,5	16,55	12,8	15,74	21,78	12,99	15,52	18,95	13,56
**Sum**	161,2	163,2	22,77	181,4	75,58	91,98	106,4	55,09	191,2	89,84	108,1	159,5

PS 32:1

	Controls healthy	Controls Bronchitis	Diffuse development. disorder	Growth abn.+ defic. alveolar.	Immuno-intact host	Immuno-compromised host	Chronic tachypnea of infancy	Reactive lymphoid lesions	Related to alv. surf. region	Related to lung vessels/heart	Related to systemic disease	Unclear RDS in mature neonate
**Number of values**	11	10	2	18	5	8	9	4	21	7	7	13
**Minimum**	0,26	0,23	0,36	0,14	0,37	0,26	0,23	0,29	0,04	0,2	0,33	0,24
**25% Percentile**	0,35	0,28		0,36	0,395	0,425	0,32	0,41	0,225	0,31	0,34	0,24
**Median**	0,39	0,31	0,435	0,48	0,46	0,525	0,41	0,58	0,42	0,38	0,51	0,42
**75% Percentile**	0,59	0,515		0,55	0,5	0,62	0,47	0,67	0,575	0,69	0,72	0,53
**Maximum**	0,87	1,22	0,51	0,79	0,52	0,92	0,53	0,71	3,63	0,71	1,1	0,85
**Mean**	0,4709	0,444	0,435	0,4672	0,45	0,54	0,3978	0,54	0,6586	0,43	0,5629	0,4131
**Std. Deviation**	0,1934	0,2921	0,1061	0,1586	0,05745	0,1989	0,09985	0,1822	0,8417	0,1953	0,2773	0,1818
**Std. Error**	0,05832	0,09235	0,075	0,03737	0,02569	0,07033	0,03328	0,0911	0,1837	0,07381	0,1048	0,05041
**Lower 95% CI of mean**	0,341	0,2351	-0,518	0,3884	0,3787	0,3737	0,321	0,2501	0,2754	0,2494	0,3064	0,3032
**Upper 95% CI of mean**	0,6008	0,6529	1,388	0,5461	0,5213	0,7063	0,4745	0,8299	1,042	0,6106	0,8193	0,5229
**Sum**	5,18	4,44	0,87	8,41	2,25	4,32	3,58	2,16	13,83	3,01	3,94	5,37

PS 32:0

	Controls healthy	Controls Bronchitis	Diffuse development. disorder	Growth abn.+ defic. alveolar.	Immuno-intact host	Immuno-compromised host	Chronic tachypnea of infancy	Reactive lymphoid lesions	Related to alv. surf. region	Related to lung vessels/heart	Related to systemic disease	Unclear RDS in mature neonate
**Number of values**	11	10	2	18	5	8	9	4	21	7	7	13
**Minimum**	0,34	0,18	0,79	0,42	0,37	0,57	0,26	0,33	0	0,38	0,35	0,37
**25% Percentile**	0,36	0,225		0,6	0,405	0,67	0,455	0,425	0,305	0,38	0,44	0,625
**Median**	0,5	0,305	1,03	0,81	0,57	0,855	0,62	0,53	0,58	0,65	0,81	0,83
**75% Percentile**	0,63	0,99		1,15	0,69	1,025	0,645	0,61	1,105	1,05	1,16	1,165
**Maximum**	2,5	2,03	1,27	1,92	0,73	2,34	1,16	0,68	2,77	1,48	1,23	1,71
**Mean**	0,6736	0,601	1,03	0,9317	0,552	1,001	0,6033	0,5175	0,7986	0,7457	0,8114	0,9031
**Std. Deviation**	0,6195	0,5944	0,3394	0,3942	0,1477	0,5689	0,247	0,1438	0,6839	0,3954	0,3297	0,3732
**Std. Error**	0,1868	0,188	0,24	0,09292	0,06606	0,2012	0,08234	0,07192	0,1492	0,1494	0,1246	0,1035
**Lower 95% CI of mean**	0,2575	0,1758	-2,019	0,7356	0,3686	0,5256	0,4134	0,2886	0,4872	0,38	0,5065	0,6776
**Upper 95% CI of mean**	1,09	1,026	4,079	1,128	0,7354	1,477	0,7932	0,7464	1,11	1,111	1,116	1,129
**Sum**	7,41	6,01	2,06	16,77	2,76	8,01	5,43	2,07	16,77	5,22	5,68	11,74

PS 34:2

	Controls healthy	Controls Bronchitis	Diffuse development. disorder	Growth abn.+ defic. alveolar.	Immuno-intact host	Immuno-compromised host	Chronic tachypnea of infancy	Reactive lymphoid lesions	Related to alv. surf. region	Related to lung vessels/heart	Related to systemic disease	Unclear RDS in mature neonate
**Number of values**	11	10	2	18	5	8	9	4	21	7	7	13
**Minimum**	0,87	0,49	0,72	0,77	0,88	0,45	1,03	1,37	0,17	0,31	0,81	0,54
**25% Percentile**	1,01	0,945		0,805	0,895	1,345	1,405	1,475	0,415	0,83	0,98	0,66
**Median**	1,29	1,135	0,73	1,03	0,93	1,525	1,82	1,64	0,99	1,42	1,44	0,78
**75% Percentile**	1,55	1,475		1,68	1,195	1,58	2,31	2,14	1,825	1,62	1,56	1,38
**Maximum**	1,92	1,6	0,74	2,89	1,22	1,7	2,71	2,58	5,62	1,8	1,82	2,85
**Mean**	1,328	1,167	0,73	1,317	1,022	1,381	1,858	1,808	1,322	1,27	1,344	1,042
**Std. Deviation**	0,3245	0,3367	0,01414	0,6641	0,1599	0,3989	0,5361	0,5328	1,294	0,5189	0,3489	0,6438
**Std. Error**	0,09783	0,1065	0,01	0,1565	0,07151	0,141	0,1787	0,2664	0,2823	0,1961	0,1319	0,1786
**Lower 95% CI of mean**	1,11	0,9261	0,6029	0,987	0,8234	1,048	1,446	0,9598	0,733	0,7901	1,022	0,6533
**Upper 95% CI of mean**	1,546	1,408	0,8571	1,647	1,221	1,715	2,27	2,655	1,911	1,75	1,667	1,431
**Sum**	14,61	11,67	1,46	23,71	5,11	11,05	16,72	7,23	27,76	8,89	9,41	13,55

PS 34:1

	Controls healthy	Controls Bronchitis	Diffuse development. disorder	Growth abn.+ defic. alveolar.	Immuno-intact host	Immuno-compromised host	Chronic tachypnea of infancy	Reactive lymphoid lesions	Related to alv. surf. region	Related to lung vessels/heart	Related to systemic disease	Unclear RDS in mature neonate
**Number of values**	11	10	2	18	5	8	9	4	21	7	7	13
**Minimum**	3,19	3,16	4,68	2,42	4,04	3,55	4,98	4,69	1,41	2,62	3,69	3,04
**25% Percentile**	4,1	3,69		3,16	4,235	5,025	5,465	4,69	2,24	3,65	3,92	4,215
**Median**	4,57	4,865	4,725	4,88	4,66	5,755	6,44	4,69	4,11	4,43	5,94	4,35
**75% Percentile**	5,1	6,11		6,63	5,735	7,48	7,47	5,81	5,76	5,35	6,04	5,385
**Maximum**	8,67	10,5	4,77	7,89	5,77	10,24	8,22	6,93	10,85	6,92	7,25	6,09
**Mean**	4,846	5,24	4,725	4,907	4,92	6,289	6,514	5,25	4,311	4,587	5,337	4,661
**Std. Deviation**	1,531	2,117	0,06364	1,742	0,7767	2,096	1,096	1,12	2,448	1,35	1,313	0,8963
**Std. Error**	0,4617	0,6696	0,045	0,4105	0,3473	0,741	0,3653	0,56	0,5342	0,5102	0,4963	0,2486
**Lower 95% CI of mean**	3,818	3,725	4,153	4,041	3,956	4,536	5,672	3,468	3,197	3,339	4,123	4,119
**Upper 95% CI of mean**	5,875	6,755	5,297	5,773	5,884	8,041	7,357	7,032	5,425	5,836	6,552	5,202
**Sum**	53,31	52,4	9,45	88,32	24,6	50,31	58,63	21	90,53	32,11	37,36	60,59

PS 34:0

	Controls healthy	Controls Bronchitis	Diffuse development. disorder	Growth abn.+ defic. alveolar.	Immuno-intact host	Immuno-compromised host	Chronic tachypnea of infancy	Reactive lymphoid lesions	Related to alv. surf. region	Related to lung vessels/heart	Related to systemic disease	Unclear RDS in mature neonate
**Number of values**	11	10	2	18	5	8	9	4	21	7	7	13

**Minimum**	0,24	0,24	0,57	0,33	0,4	0,34	0,11	0,29	0,22	0,49	0,26	0,39
**25% Percentile**	0,36	0,27		0,51	0,485	0,645	0,265	0,355	0,495	0,57	0,37	0,52
**Median**	0,49	0,38	0,675	0,695	0,67	0,805	0,46	0,515	0,72	0,69	0,91	0,79
**75% Percentile**	0,6	1,07		0,99	0,985	0,92	0,5	0,75	1,13	1,26	1,04	1,055
**Maximum**	1,87	1,64	0,78	1,32	1,29	1,58	0,83	0,89	2,22	1,66	1,16	1,27
**Mean**	0,6018	0,609	0,675	0,7544	0,722	0,8325	0,4233	0,5525	0,8495	0,8971	0,7729	0,7946
**Std. Deviation**	0,4469	0,494	0,1485	0,291	0,3369	0,357	0,2093	0,2606	0,4993	0,4259	0,3439	0,2851
**Std. Error**	0,1347	0,1562	0,105	0,06859	0,1506	0,1262	0,06978	0,1303	0,109	0,161	0,13	0,07907
**Lower 95% CI of mean**	0,3016	0,2556	-0,6591	0,6097	0,3037	0,534	0,2624	0,1379	0,6222	0,5032	0,4548	0,6223
**Upper 95% CI of mean**	0,902	0,9624	2,009	0,8992	1,14	1,131	0,5842	0,9671	1,077	1,291	1,091	0,9669
**Sum**	6,62	6,09	1,35	13,58	3,61	6,66	3,81	2,21	17,84	6,28	5,41	10,33

PS 36:4

	**Controls healthy**	**Controls Bronchitis**	**Diffuse development. disorder**	**Growth abn.+ defic. alveolar.**	**Immuno-intact host**	**Immuno-compromised host**	**Chronic tachypnea of infancy**	**Reactive lymphoid lesions**	**Related to alv. surf. region**	**Related to lung vessels/heart**	**Related to systemic disease**	**Unclear RDS in mature neonate**
**Number of values**	11	10	2	18	5	8	9	4	21	7	7	13
**Minimum**	0,56	0,41	0,69	0,62	0,96	0,62	0,87	1,04	0,1	0,21	0,55	0,53
**25% Percentile**	1,1	0,725		0,855	1,015	0,9	1,015	1,07	0,37	0,88	0,67	0,655
**Median**	1,16	1,15	0,98	1,15	1,36	1,415	1,25	1,285	1,04	0,97	1,38	0,99
**75% Percentile**	1,34	1,605		1,32	1,415	1,755	1,865	1,785	1,79	1,45	1,83	1,295
**Maximum**	1,98	1,98	1,27	1,98	1,42	1,89	2,4	2,1	3,51	1,47	1,88	1,79
**Mean**	1,21	1,149	0,98	1,134	1,244	1,331	1,447	1,428	1,109	0,9929	1,253	1,009
**Std. Deviation**	0,3692	0,4891	0,4101	0,3564	0,2138	0,474	0,5185	0,487	0,8856	0,4244	0,56	0,3869
**Std. Error**	0,1113	0,1547	0,29	0,08401	0,09563	0,1676	0,1728	0,2435	0,1933	0,1604	0,2117	0,1073
**Lower 95% CI of mean**	0,9619	0,7991	-2,705	0,9572	0,9785	0,935	1,048	0,6526	0,7059	0,6003	0,7349	0,7754
**Upper 95% CI of mean**	1,458	1,499	4,665	1,312	1,51	1,727	1,845	2,202	1,512	1,385	1,771	1,243
**Sum**	13,31	11,49	1,96	20,42	6,22	10,65	13,02	5,71	23,29	6,95	8,77	13,12

PS 36:3

	**Controls healthy**	**Controls Bronchitis**	**Diffuse development. disorder**	**Growth abn.+ defic. alveolar.**	**Immuno-intact host**	**Immuno-compromised host**	**Chronic tachypnea of infancy**	**Reactive lymphoid lesions**	**Related to alv. surf. region**	**Related to lung vessels/heart**	**Related to systemic disease**	**Unclear RDS in mature neonate**
**Number of values**	11	10	2	18	5	8	9	4	21	7	7	13
**Minimum**	1,37	0,65	0,89	0,95	1,3	0,81	1,75	1,37	0	0,53	1,11	0,54
**25% Percentile**	1,99	1,525		1,215	1,32	1,64	2,2	1,61	0,535	1,37	1,42	0,805
**Median**	2,16	1,715	0,965	1,66	1,56	2,015	2,6	2,35	1,01	1,65	1,67	1,15
**75% Percentile**	2,52	2,265		2,365	2,06	2,33	3,155	2,955	2,345	3,02	2,48	2,375
**Maximum**	3,07	2,74	1,04	4,18	2,42	3,09	3,47	3,06	4,88	3,65	3,14	2,67
**Mean**	2,18	1,804	0,965	1,957	1,664	1,984	2,638	2,283	1,509	1,949	1,929	1,444
**Std. Deviation**	0,5101	0,5675	0,1061	0,9176	0,4531	0,6709	0,5586	0,8055	1,213	1,051	0,6898	0,7947
**Std. Error**	0,1538	0,1794	0,075	0,2163	0,2026	0,2372	0,1862	0,4027	0,2646	0,3974	0,2607	0,2204
**Lower 95% CI of mean**	1,837	1,398	0,01204	1,501	1,101	1,423	2,208	1,001	0,9571	0,9762	1,291	0,9636
**Upper 95% CI of mean**	2,523	2,21	1,918	2,414	2,227	2,545	3,067	3,564	2,061	2,921	2,567	1,924
**Sum**	23,98	18,04	1,93	35,23	8,32	15,87	23,74	9,13	31,69	13,64	13,5	18,77

PS 36:2

	**Controls healthy**	**Controls Bronchitis**	**Diffuse development. disorder**	**Growth abn.+ defic. alveolar.**	**Immuno-intact host**	**Immuno-compromised host**	**Chronic tachypnea of infancy**	**Reactive lymphoid lesions**	**Related to alv. surf. region**	**Related to lung vessels/heart**	**Related to systemic disease**	**Unclear RDS in mature neonate**
**Number of values**	11	10	2	18	5	8	9	4	21	7	7	13
**Minimum**	9,29	8,21	7,04	6,01	9,66	7,51	10,89	9,65	3,55	6,33	8,99	4,91
**25% Percentile**	10,04	11,21		8,53	10,03	11,1	14,46	10,94	6,775	9,42	9,61	7,54
**Median**	13,36	13,25	7,785	10,49	11,19	12,52	15,85	13,7	8,37	12,78	10,21	8,98
**75% Percentile**	17,03	13,82		14,05	14,23	15,6	19,69	17,37	11,35	20,84	11,86	13,2
**Maximum**	20,62	16,73	8,53	24,95	15,64	16,08	21,95	19,56	18,22	23,77	13,22	15,75
**Mean**	14,18	12,66	7,785	12,03	11,94	12,75	16,7	14,15	9,277	13,81	10,78	9,652
**Std. Deviation**	3,962	2,264	1,054	5,058	2,376	3,027	3,528	4,252	3,778	6,301	1,537	3,373
**Std. Error**	1,195	0,7158	0,745	1,192	1,063	1,07	1,176	2,126	0,8243	2,382	0,5811	0,9355
**Lower 95% CI of mean**	11,51	11,04	-1,681	9,512	8,99	10,22	13,99	7,386	7,557	7,979	9,357	7,613
**Upper 95% CI of mean**	16,84	14,28	17,25	14,54	14,89	15,28	19,42	20,92	11	19,64	12,2	11,69
**Sum**	155,9	126,6	15,57	216,5	59,7	102	150,3	56,61	194,8	96,65	75,45	125,5

PS 36:1

	**Controls healthy**	**Controls Bronchitis**	**Diffuse development. disorder**	**Growth abn.+ defic. alveolar.**	**Immuno-intact host**	**Immuno-compromised host**	**Chronic tachypnea of infancy**	**Reactive lymphoid lesions**	**Related to alv. surf. region**	**Related to lung vessels/heart**	**Related to systemic disease**	**Unclear RDS in mature neonate**
**Number of values**	11	10	2	18	5	8	9	4	21	7	7	13
**Minimum**	30,03	35	40,77	27,87	29,38	32,08	30,6	26,01	18,16	33,07	25,39	34,86
**25% Percentile**	31,72	36,62		34,42	31,83	34,21	34,06	31,26	29,88	33,2	30,47	40,3
**Median**	33,6	40,41	42,56	38,56	37,87	40,13	35,4	36,94	36,01	43,36	32,88	44,65
**75% Percentile**	38,05	55,84		43,63	41,28	41,72	39,17	39,96	52,65	52,34	43,1	50,58

**Maximum**	41,16	63,61	44,34	51,73	43,34	45,95	48,43	42,54	71,25	56,89	46,15	53,67
**Mean**	34,74	44,79	42,56	39,37	36,82	38,77	36,99	35,61	40,69	43,69	35,87	44,8
**Std. Deviation**	3,776	10,18	2,524	6,496	5,273	4,783	5,068	6,931	14,71	8,942	7,471	5,605
**Std. Error**	1,138	3,219	1,785	1,531	2,358	1,691	1,689	3,465	3,211	3,38	2,824	1,555
**Lower 95% CI of mean**	32,2	37,5	19,87	36,14	30,27	34,77	33,1	24,58	34	35,42	28,96	41,41
**Upper 95% CI of mean**	37,28	52,07	65,24	42,6	43,36	42,76	40,89	46,64	47,39	51,96	42,78	48,19
**Sum**	382,1	447,9	85,11	708,7	184,1	310,1	332,9	142,4	854,6	305,8	251,1	582,4

PS 38:5

	Controls healthy	Controls Bronchitis	Diffuse development. disorder	Growth abn.+ defic. alveolar.	Immuno-intact host	Immuno-compromised host	Chronic tachypnea of infancy	Reactive lymphoid lesions	Related to alv. surf. region	Related to lung vessels/heart	Related to systemic disease	Unclear RDS in mature neonate
**Number of values**	11	10	2	18	5	8	9	4	21	7	7	13
**Minimum**	0,5	0,29	0,53	0,41	0,81	0,47	0,69	0,82	0,18	0,32	0,48	0,25
**25% Percentile**	0,94	0,665		0,625	0,84	0,645	0,835	0,905	0,305	0,51	0,82	0,45
**Median**	1,2	0,86	0,635	0,85	0,97	0,905	1,18	1,08	0,74	0,78	1,28	0,66
**75% Percentile**	1,3	1,655		1,105	1,285	1,3	1,42	1,56	1,195	1,11	1,62	0,935
**Maximum**	1,86	1,79	0,74	1,63	1,34	1,68	1,76	1,95	1,98	1,11	1,78	1,9
**Mean**	1,144	1,023	0,635	0,8889	1,044	0,9812	1,162	1,233	0,8114	0,77	1,209	0,7362
**Std. Deviation**	0,3728	0,5059	0,1485	0,316	0,2306	0,4324	0,3563	0,4992	0,5814	0,3072	0,4489	0,4215
**Std. Error**	0,1124	0,16	0,105	0,07448	0,1031	0,1529	0,1188	0,2496	0,1269	0,1161	0,1697	0,1169
**Lower 95% CI of mean**	0,8932	0,6611	-0,6991	0,7318	0,7577	0,6198	0,8884	0,4381	0,5468	0,4858	0,7934	0,4814
**Upper 95% CI of mean**	1,394	1,385	1,969	1,046	1,33	1,343	1,436	2,027	1,076	1,054	1,624	0,9909
**Sum**	12,58	10,23	1,27	16	5,22	7,85	10,46	4,93	17,04	5,39	8,46	9,57

PS 38:4

	Controls healthy	Controls Bronchitis	Diffuse development. disorder	Growth abn.+ defic. alveolar.	Immuno-intact host	Immuno-compromised host	Chronic tachypnea of infancy	Reactive lymphoid lesions	Related to alv. surf. region	Related to lung vessels/heart	Related to systemic disease	Unclear RDS in mature neonate
**Number of values**	11	10	2	18	5	8	9	4	21	7	7	13
**Minimum**	2,89	4,03	4,96	2,9	5,77	3,98	4,35	4,23	1,85	2,59	2,94	3,86
**25% Percentile**	5,85	4,57		4,51	6,53	6,48	6,01	4,755	3,235	5,03	4,9	4,775
**Median**	7,3	6,375	5,44	6,215	7,59	7,1	7,88	6,275	5,44	5,49	10,08	5,23
**75% Percentile**	9,35	8,22		8,1	8,895	8,155	9,56	8,445	7,335	7,54	11,78	7,8
**Maximum**	9,96	12,78	5,92	12,41	9,06	9,62	11,74	9,62	16,35	8,28	18,97	13,53
**Mean**	7,247	6,776	5,44	6,371	7,688	7,134	7,782	6,6	5,982	5,864	9,646	6,375
**Std. Deviation**	2,323	2,592	0,6788	2,388	1,305	1,701	2,351	2,375	3,486	1,932	5,216	2,724
**Std. Error**	0,7005	0,8198	0,48	0,5629	0,5837	0,6013	0,7837	1,188	0,7607	0,7303	1,971	0,7554
**Lower 95% CI of mean**	5,687	4,922	-0,659	5,183	6,067	5,712	5,975	2,82	4,396	4,077	4,822	4,73
**Upper 95% CI of mean**	8,808	8,63	11,54	7,558	9,309	8,556	9,589	10,38	7,569	7,651	14,47	8,021
**Sum**	79,72	67,76	10,88	114,7	38,44	57,07	70,04	26,4	125,6	41,05	67,52	82,88

PS 38:3

	Controls healthy	Controls Bronchitis	Diffuse development. disorder	Growth abn.+ defic. alveolar.	Immuno-intact host	Immuno-compromised host	Chronic tachypnea of infancy	Reactive lymphoid lesions	Related to alv. surf. region	Related to lung vessels/heart	Related to systemic disease	Unclear RDS in mature neonate
**Number of values**	11	10	2	18	5	8	9	4	21	7	7	13
**Minimum**	2,34	2,63	4,08	3,25	3,68	3,65	2,21	2,82	0	2,49	3,93	3,44
**25% Percentile**	2,88	3,035		4,51	3,82	4,64	2,62	3,305	3,4	2,84	4,18	3,875
**Median**	3,17	4	5,07	5,025	4,69	4,9	3,57	3,8	4,75	3,95	4,72	4,59
**75% Percentile**	4,36	5,15		5,815	5,03	6,66	4,03	4,11	5,245	5,37	6,88	6,195
**Maximum**	4,41	6,85	6,06	7,88	5,28	8,13	7,07	4,41	8,54	5,39	7,08	8,39
**Mean**	3,448	4,181	5,07	5,178	4,478	5,523	3,654	3,708	4,516	4,169	5,42	5,042
**Std. Deviation**	0,7937	1,306	1,4	1,062	0,6489	1,534	1,447	0,6579	1,841	1,222	1,334	1,459
**Std. Error**	0,2393	0,413	0,99	0,2503	0,2902	0,5423	0,4824	0,3289	0,4016	0,4617	0,5043	0,4046
**Lower 95% CI of mean**	2,915	3,247	-7,509	4,65	3,672	4,24	2,542	2,661	3,678	3,039	4,186	4,161
**Upper 95% CI of mean**	3,981	5,115	17,65	5,706	5,284	6,805	4,767	4,754	5,354	5,298	6,654	5,924
**Sum**	37,93	41,81	10,14	93,2	22,39	44,18	32,89	14,83	94,84	29,18	37,94	65,55

PS 38:2

	Controls healthy	Controls Bronchitis	Diffuse development. disorder	Growth abn.+ defic. alveolar.	Immuno-intact host	Immuno-compromised host	Chronic tachypnea of infancy	Reactive lymphoid lesions	Related to alv. surf. region	Related to lung vessels/heart	Related to systemic disease	Unclear RDS in mature neonate
**Number of values**	11	10	2	18	5	8	9	4	21	7	7	13
**Minimum**	1,54	1,54	2,77	1,6	1,9	2,1	1,91	1,64	1,1	1,55	1,68	1,67
**25% Percentile**	1,95	1,745		2,085	1,965	2,27	1,99	1,835	1,965	2,01	1,76	2,085
**Median**	2,23	2,15	3,17	2,57	2,26	2,59	2,53	2,095	2,58	2,25	2,04	2,58
**75% Percentile**	2,59	2,305		3,13	2,945	3,015	2,74	2,42	3,31	3,26	3	3,065
**Maximum**	3,86	2,59	3,57	3,55	3,44	3,85	2,82	2,68	5,16	3,91	3,73	3,39
**Mean**	2,324	2,059	3,17	2,614	2,416	2,713	2,409	2,128	2,724	2,514	2,357	2,581
**Std. Deviation**	0,6057	0,3281	0,5657	0,59	0,6102	0,58	0,3593	0,4295	0,9676	0,8022	0,7523	0,5618
**Std. Error**	0,1826	0,1038	0,4	0,1391	0,2729	0,2051	0,1198	0,2148	0,2111	0,3032	0,2844	0,1558

**Lower 95% CI of mean**	1,917	1,824	-1,912	2,321	1,658	2,228	2,133	1,444	2,284	1,772	1,661	2,241
**Upper 95% CI of mean**	2,731	2,294	8,252	2,907	3,174	3,197	2,685	2,811	3,165	3,256	3,053	2,92
**Sum**	25,56	20,59	6,34	47,05	12,08	21,7	21,68	8,51	57,21	17,6	16,5	33,55

PS 38:1

	Controls healthy	Controls Bronchitis	Diffuse development. disorder	Growth abn.+ defic. alveolar.	Immuno-intact host	Immuno-compromised host	Chronic tachypnea of infancy	Reactive lymphoid lesions	Related to alv. surf. region	Related to lung vessels/heart	Related to systemic disease	Unclear RDS in mature neonate
**Number of values**	11	10	2	18	5	8	9	4	21	7	7	13
**Minimum**	1,63	1,66	2,47	1,5	2,06	1,82	1,81	1,37	1,31	1,88	1,88	1,54
**25% Percentile**	2,14	1,76		2,085	2,085	2,085	2,075	1,46	1,78	1,97	1,97	2,155
**Median**	2,35	2,07	2,545	2,545	2,31	2,41	2,43	1,795	2,51	2,09	2,36	2,32
**75% Percentile**	2,73	2,535		2,765	2,79	2,63	2,77	2,14	3,07	2,82	2,75	2,795
**Maximum**	3,58	3	2,62	3,91	3,07	3,06	2,98	2,24	4,3	3,19	2,96	3,51
**Mean**	2,391	2,164	2,545	2,484	2,412	2,391	2,413	1,8	2,483	2,367	2,367	2,41
**Std. Deviation**	0,5344	0,4331	0,1061	0,5403	0,4087	0,4158	0,3853	0,4077	0,8581	0,4959	0,3875	0,5448
**Std. Error**	0,1611	0,1369	0,075	0,1274	0,1828	0,147	0,1284	0,2038	0,1872	0,1874	0,1464	0,1511
**Lower 95% CI of mean**	2,032	1,854	1,592	2,215	1,905	2,044	2,117	1,151	2,093	1,908	2,009	2,081
**Upper 95% CI of mean**	2,75	2,474	3,498	2,753	2,919	2,739	2,709	2,449	2,874	2,826	2,725	2,739
**Sum**	26,3	21,64	5,09	44,71	12,06	19,13	21,72	7,2	52,15	16,57	16,57	31,33

PS 40:6

	Controls healthy	Controls Bronchitis	Diffuse development. disorder	Growth abn.+ defic. alveolar.	Immuno-intact host	Immuno-compromised host	Chronic tachypnea of infancy	Reactive lymphoid lesions	Related to alv. surf. region	Related to lung vessels/heart	Related to systemic disease	Unclear RDS in mature neonate
**Number of values**	11	10	2	18	5	8	9	4	21	7	7	13
**Minimum**	0,57	0,76	4,05	0,65	1,32	0,89	0,73	1,71	0	0,37	0,94	1,89
**25% Percentile**	0,91	1,255		1,025	1,635	1,355	1,24	1,77	1,73	0,72	1,7	2,605
**Median**	1,35	1,895	4,05	2,8	2,11	1,93	1,5	1,975	2,26	1,83	1,93	3,29
**75% Percentile**	1,73	2,32		4,28	3,11	2,615	2,41	2,72	4,285	3,36	2,51	5,09
**Maximum**	2,97	2,68	4,05	5,93	3,5	3,6	2,52	3,32	7,58	3,67	3,91	6,35
**Mean**	1,425	1,801	4,05	2,76	2,32	2,036	1,692	2,245	3,044	1,821	2,129	3,846
**Std. Deviation**	0,6516	0,5955	0	1,731	0,8266	0,8931	0,6316	0,737	1,99	1,297	0,9153	1,46
**Std. Error**	0,1965	0,1883	0	0,4079	0,3697	0,3158	0,2105	0,3685	0,4342	0,4901	0,3459	0,4048
**Lower 95% CI of mean**	0,9868	1,375	4,05	1,899	1,294	1,29	1,207	1,072	2,138	0,6223	1,282	2,964
**Upper 95% CI of mean**	1,862	2,227	4,05	3,621	3,346	2,783	2,178	3,418	3,95	3,021	2,975	4,728
**Sum**	15,67	18,01	8,1	49,68	11,6	16,29	15,23	8,98	63,92	12,75	14,9	50

PS 40:5

	Controls healthy	Controls Bronchitis	Diffuse development. disorder	Growth abn.+ defic. alveolar.	Immuno-intact host	Immuno-compromised host	Chronic tachypnea of infancy	Reactive lymphoid lesions	Related to alv. surf. region	Related to lung vessels/heart	Related to systemic disease	Unclear RDS in mature neonate
**Number of values**	11	10	2	18	5	8	9	4	21	7	7	13
**Minimum**	0,64	1,17	1,87	0,79	1,49	1,55	0,85	1,34	0	0,52	1,41	0,62
**25% Percentile**	1,19	1,32		1,23	1,56	1,735	0,995	1,435	1,52	0,82	1,84	1,42
**Median**	1,31	1,565	2,015	1,66	1,74	2,38	1,1	1,585	1,9	1	2,22	1,7
**75% Percentile**	1,6	2,065		2,01	2,21	2,75	1,495	1,645	2,86	1,93	3,5	2,08
**Maximum**	1,9	2,64	2,16	2,91	2,42	2,81	1,69	1,65	3,63	2,29	4,02	2,79
**Mean**	1,352	1,698	2,015	1,656	1,856	2,261	1,213	1,54	2,063	1,271	2,613	1,732
**Std. Deviation**	0,343	0,4526	0,2051	0,5598	0,3665	0,5217	0,298	0,144	0,9139	0,6473	0,9737	0,5344
**Std. Error**	0,1034	0,1431	0,145	0,1319	0,1639	0,1844	0,09932	0,072	0,1994	0,2447	0,368	0,1482
**Lower 95% CI of mean**	1,121	1,374	0,1726	1,377	1,401	1,825	0,9843	1,311	1,647	0,6728	1,712	1,409
**Upper 95% CI of mean**	1,582	2,022	3,857	1,934	2,311	2,697	1,442	1,769	2,479	1,87	3,513	2,055
**Sum**	14,87	16,98	4,03	29,8	9,28	18,09	10,92	6,16	43,33	8,9	18,29	22,52

PS 40:4

	Controls healthy	Controls Bronchitis	Diffuse development. disorder	Growth abn.+ defic. alveolar.	Immuno-intact host	Immuno-compromised host	Chronic tachypnea of infancy	Reactive lymphoid lesions	Related to alv. surf. region	Related to lung vessels/heart	Related to systemic disease	Unclear RDS in mature neonate
**Number of values**	11	10	2	18	5	8	9	4	21	7	7	13
**Minimum**	0,84	1,19	3,04	0,76	1,61	1,96	0,99	1,5	1,15	0,93	1,86	1,36
**25% Percentile**	1,39	1,705		2,06	1,95	2,105	1,115	1,7	1,935	1,15	2,42	2,105
**Median**	1,7	2,125	4,1	2,57	2,69	2,91	1,66	1,92	2,23	1,69	3,19	2,99
**75% Percentile**	2,04	2,36		3,145	2,935	3,445	2,155	2,36	4,435	3,14	3,86	4,38
**Maximum**	2,56	2,92	5,16	4,92	2,97	3,77	2,29	2,78	6,15	3,64	7,19	5,86
**Mean**	1,721	2,066	4,1	2,566	2,492	2,831	1,676	2,03	3,085	2,003	3,61	3,192
**Std. Deviation**	0,4694	0,4785	1,499	0,9362	0,5597	0,7037	0,5043	0,538	1,532	1,01	1,73	1,309
**Std. Error**	0,1415	0,1513	1,06	0,2207	0,2503	0,2488	0,1681	0,269	0,3342	0,3816	0,6539	0,3631
**Lower 95% CI of mean**	1,406	1,724	-9,369	2,101	1,797	2,243	1,288	1,174	2,388	1,069	2,01	2,401
**Upper 95% CI of mean**	2,036	2,408	17,57	3,032	3,187	3,42	2,063	2,886	3,782	2,937	5,21	3,983
**Sum**	18,93	20,66	8,2	46,19	12,46	22,65	15,08	8,12	64,79	14,02	25,27	41,5

PS 40:3

	Controls healthy	Controls Bronchitis	Diffuse development. disorder	Growth abn.+ defic. alveolar.	Immuno-intact host	Immuno-compromised host	Chronic tachypnea of infancy	Reactive lymphoid lesions	Related to alv. surf. region	Related to lung vessels/heart	Related to systemic disease	Unclear RDS in mature neonate
**Number of values**	11	10	2	18	5	8	9	4	21	7	7	13
**Minimum**	0,53	0,39	1,16	0,58	0,59	0,68	0,44	0,49	0,44	0,33	0,46	0,35
**25% Percentile**	0,7	0,49		0,795	0,63	0,775	0,53	0,63	0,685	0,61	0,59	0,725
**Median**	0,75	0,56	1,17	0,965	0,87	0,9	0,65	0,915	0,84	0,72	1,08	0,83
**75% Percentile**	0,84	0,63		1,105	1,16	1,08	0,825	1,07	1,36	0,88	1,28	0,945
**Maximum**	1	0,79	1,18	1,71	1,25	1,63	1,04	1,08	5,33	1,43	1,49	1,43
**Mean**	0,7627	0,567	1,17	0,9806	0,89	0,9775	0,6767	0,85	1,197	0,7657	0,97	0,8377
**Std. Deviation**	0,1351	0,1064	0,01414	0,2638	0,2742	0,3051	0,1894	0,2787	1,064	0,3396	0,377	0,2552
**Std. Error**	0,04072	0,03363	0,01	0,06217	0,1226	0,1079	0,06314	0,1393	0,2322	0,1283	0,1425	0,07078
**Lower 95% CI of mean**	0,672	0,4909	1,043	0,8494	0,5495	0,7224	0,5311	0,4065	0,7127	0,4517	0,6213	0,6835
**Upper 95% CI of mean**	0,8535	0,6431	1,297	1,112	1,23	1,233	0,8223	1,293	1,682	1,08	1,319	0,9919
**Sum**	8,39	5,67	2,34	17,65	4,45	7,82	6,09	3,4	25,14	5,36	6,79	10,89

PG 30:0

	Controls healthy	Controls Bronchitis	Diffuse development. disorder	Growth abn.+ defic. alveolar.	Immuno-intact host	Immuno-compromised host	Chronic tachypnea of infancy	Reactive lymphoid lesions	Related to alv. surf. region	Related to lung vessels/heart	Related to systemic disease	Unclear RDS in mature neonate
**Number of values**	11	10	2	18	5	8	8	4	17	7	7	13
**Minimum**	0,42	0,62	1,5	0,36	0,77	0,45	0,5	0,59	0	0,13	0,58	0,47
**25% Percentile**	0,54	0,64		0,59	0,815	0,935	0,72	0,655	0,78	0,64	0,88	0,845
**Median**	0,66	0,995	2,715	1,115	1,28	1,115	0,845	0,88	1,88	0,95	2,34	1,02
**75% Percentile**	1,18	1,255		2,44	1,655	1,43	0,94	1,14	3,21	1,47	4,24	2,925
**Maximum**	1,65	5,27	3,93	3,5	1,79	1,85	1,31	1,24	4,75	4,03	5,07	4,64
**Mean**	0,88	1,366	2,715	1,473	1,244	1,158	0,8525	0,8975	2,02	1,306	2,579	1,772
**Std. Deviation**	0,4232	1,397	1,718	1,091	0,4324	0,4279	0,2369	0,2965	1,518	1,268	1,661	1,349
**Std. Error**	0,1276	0,4416	1,215	0,2571	0,1934	0,1513	0,08375	0,1482	0,3682	0,4794	0,6278	0,3742
**Lower 95% CI of mean**	0,5957	0,3669	-12,72	0,9309	0,7072	0,7998	0,6545	0,4258	1,239	0,1327	1,042	0,9562
**Upper 95% CI of mean**	1,164	2,365	18,15	2,016	1,781	1,515	1,051	1,369	2,801	2,479	4,115	2,587
**Sum**	9,68	13,66	5,43	26,52	6,22	9,26	6,82	3,59	34,34	9,14	18,05	23,03

PG 32:1

	Controls healthy	Controls Bronchitis	Diffuse development. disorder	Growth abn.+ defic. alveolar.	Immuno-intact host	Immuno-compromised host	Chronic tachypnea of infancy	Reactive lymphoid lesions	Related to alv. surf. region	Related to lung vessels/heart	Related to systemic disease	Unclear RDS in mature neonate
**Number of values**	11	10	2	18	5	8	9	4	20	7	7	13
**Minimum**	1,35	1,45	1,95	0	1,27	2,04	0	1,82	0	0	1,82	0
**25% Percentile**	1,51	1,775		1,6	1,625	2,2	1,71	2,23	0	0,96	2,29	0,865
**Median**	1,95	2,315	2	2,35	2	2,415	2,04	2,94	1,18	2,33	3,05	2,03
**75% Percentile**	2,9	2,8		3,1	2,75	2,645	2,34	3,485	2,31	3,12	4,64	3,31
**Maximum**	3,84	3,05	2,05	6,33	3,49	2,83	2,67	3,73	7,14	3,79	5,6	6,96
**Mean**	2,161	2,262	2	2,487	2,15	2,424	1,863	2,858	1,496	2,081	3,31	2,365
**Std. Deviation**	0,796	0,5424	0,07071	1,508	0,8126	0,2767	0,7744	0,8229	1,724	1,298	1,376	1,958
**Std. Error**	0,24	0,1715	0,05	0,3554	0,3634	0,09783	0,2581	0,4114	0,3854	0,4905	0,5202	0,5429
**Lower 95% CI of mean**	1,626	1,874	1,365	1,737	1,141	2,192	1,268	1,548	0,6888	0,8813	2,037	1,182
**Upper 95% CI of mean**	2,696	2,65	2,635	3,237	3,159	2,655	2,459	4,167	2,302	3,282	4,583	3,548
**Sum**	23,77	22,62	4	44,77	10,75	19,39	16,77	11,43	29,91	14,57	23,17	30,75

PG 32:0

	Controls healthy	Controls Bronchitis	Diffuse development. disorder	Growth abn.+ defic. alveolar.	Immuno-intact host	Immuno-compromised host	Chronic tachypnea of infancy	Reactive lymphoid lesions	Related to alv. surf. region	Related to lung vessels/heart	Related to systemic disease	Unclear RDS in mature neonate
**Number of values**	11	10	2	18	5	8	9	4	20	7	7	13
**Minimum**	3,86	4,05	8,07	2,4	4,43	4,07	0	5,12	0	1,94	4,12	1,36
**25% Percentile**	4,19	4,555		4,89	4,655	5,98	4,325	5,145	0,985	2,5	4,32	3,53
**Median**	4,85	5,43	9,005	6,08	5,55	7,275	5,22	6,14	4,13	5,53	7,56	7,27
**75% Percentile**	5,74	7,135		7,355	7,11	8,925	7,895	7,33	6,475	6,6	10,15	8,765
**Maximum**	6,37	16,37	9,94	13,88	7,52	15,89	10,07	7,55	31,99	9,91	16,95	14,45
**Mean**	4,973	6,569	9,005	6,243	5,816	8,04	5,719	6,238	5,414	5,261	8,391	6,817
**Std. Deviation**	0,8487	3,624	1,322	2,602	1,28	3,596	2,91	1,274	7,019	2,67	4,436	3,748
**Std. Error**	0,2559	1,146	0,935	0,6133	0,5724	1,271	0,97	0,6372	1,57	1,009	1,677	1,039
**Lower 95% CI of mean**	4,403	3,977	-2,875	4,949	4,227	5,034	3,482	4,21	2,129	2,792	4,288	4,552
**Upper 95% CI of mean**	5,543	9,161	20,89	7,537	7,405	11,05	7,956	8,265	8,699	7,73	12,49	9,082
**Sum**	54,7	65,69	18,01	112,4	29,08	64,32	51,47	24,95	108,3	36,83	58,74	88,62

PG 34:2

	Controls healthy	Controls Bronchitis	Diffuse development. disorder	Growth abn.+ defic. alveolar.	Immuno-intact host	Immuno-compromised host	Chronic tachypnea of infancy	Reactive lymphoid lesions	Related to alv. surf. region	Related to lung vessels/heart	Related to systemic disease	Unclear RDS in mature neonate
**Number of values**	11	10	2	18	5	8	9	4	21	7	7	13
**Minimum**	2,42	2,79	2,13	0	3,22	3,14	0	5,13	0	2,17	3,55	0
**25% Percentile**	3,15	2,93		3,475	3,335	4,36	3,15	5,135	0,59	3,31	4,07	2,51
**Median**	4,6	3,64	3,98	5,89	3,69	5,63	3,85	5,27	3,51	4,41	6,28	4,83
**75% Percentile**	5,26	4,775		6,68	4,45	6,51	4,725	5,865	5,475	6,21	8,5	6,6
**Maximum**	6,17	8,64	5,83	8,39	5,03	7,6	5,81	6,33	14,74	6,55	12,36	9,77
**Mean**	4,277	4,136	3,98	5,173	3,852	5,468	3,681	5,5	4,112	4,524	6,953	4,763
**Std. Deviation**	1,29	1,744	2,616	2,057	0,7027	1,466	1,622	0,5673	3,914	1,568	2,991	3,083
**Std. Error**	0,3889	0,5516	1,85	0,4848	0,3143	0,5181	0,5406	0,2836	0,854	0,5925	1,131	0,855
**Lower 95% CI of mean**	3,411	2,888	-19,53	4,15	2,979	4,242	2,434	4,597	2,33	3,074	4,186	2,9
**Upper 95% CI of mean**	5,144	5,384	27,49	6,196	4,725	6,693	4,928	6,403	5,893	5,974	9,719	6,626
**Sum**	47,05	41,36	7,96	93,12	19,26	43,74	33,13	22	86,35	31,67	48,67	61,92

PG 34:1

	Controls healthy	Controls Bronchitis	Diffuse development. disorder	Growth abn.+ defic. alveolar.	Immuno-intact host	Immuno-compromised host	Chronic tachypnea of infancy	Reactive lymphoid lesions	Related to alv. surf. region	Related to lung vessels/heart	Related to systemic disease	Unclear RDS in mature neonate
**Number of values**	11	10	2	18	5	8	9	4	21	7	7	13

**Minimum**	22,88	16,09	12,1	2,74	26,44	20,19	26,22	25,01	1,78	11,93	18,6	9,36
**25% Percentile**	27,03	27,98		21,2	26,69	23,23	30,84	25,3	7,99	17,68	22,71	17,42
**Median**	29,02	32,48	19,56	28,02	27,33	24,93	33,89	28,74	18,49	30,04	24,65	25,79
**75% Percentile**	32,58	36,64		29,37	30,72	28,26	37,84	33,59	25,64	32,65	30,43	29,74
**Maximum**	37,18	39,73	27,02	35,47	33,89	31,34	39,76	35,29	100	36,95	34,42	31,43
**Mean**	29,79	31,45	19,56	25,03	28,43	25,54	34,11	29,44	21,02	26,82	26	23,73
**Std. Deviation**	4,38	6,781	10,55	8,492	3,082	3,675	4,4	4,992	20,67	8,847	5,237	7,507
**Std. Error**	1,321	2,144	7,46	2,002	1,379	1,299	1,467	2,496	4,51	3,344	1,98	2,082
**Lower 95% CI of mean**	26,85	26,6	-75,23	20,81	24,6	22,47	30,73	21,5	11,61	18,64	21,16	19,19
**Upper 95% CI of mean**	32,73	36,3	114,3	29,25	32,26	28,62	37,49	37,39	30,43	35	30,85	28,26
**Sum**	327,7	314,5	39,12	450,6	142,1	204,4	307	117,8	441,4	187,7	182	308,4

PG 34:0

	**Controls healthy**	**Controls Bronchitis**	**Diffuse development. disorder**	**Growth abn.+ defic. alveolar.**	**Immuno-intact host**	**Immuno-compromised host**	**Chronic tachypnea of infancy**	**Reactive lymphoid lesions**	**Related to alv. surf. region**	**Related to lung vessels/heart**	**Related to systemic disease**	**Unclear RDS in mature neonate**
**Number of values**	11	10	2	18	5	8	9	4	21	7	7	13
**Minimum**	1,24	1,17	0	0	0,47	1,35	0	0,65	0	0,72	0,7	0
**25% Percentile**	1,25	1,59		0,96	0,85	1,415	1,275	1,125	0	1,07	0,98	0,4
**Median**	1,77	1,88	0,9	1,645	1,45	1,53	1,57	1,725	1,03	1,35	1,81	1,44
**75% Percentile**	2,81	2,75		2,145	2,65	2,245	2,665	1,995	2,42	1,78	3,05	2,275
**Maximum**	2,96	3,29	1,8	3,49	2,84	4,14	3,7	2,14	8,83	2,34	3,35	3,74
**Mean**	1,935	2,074	0,9	1,649	1,69	1,984	1,818	1,56	1,585	1,409	1,94	1,558
**Std. Deviation**	0,699	0,6592	1,273	0,797	0,9583	0,9786	1,065	0,6455	2,294	0,5261	1,022	1,229
**Std. Error**	0,2108	0,2085	0,9	0,1879	0,4285	0,346	0,355	0,3228	0,5006	0,1988	0,3862	0,3409
**Lower 95% CI of mean**	1,465	1,602	-10,54	1,253	0,5002	1,166	0,9992	0,5328	0,541	0,922	0,9949	0,8157
**Upper 95% CI of mean**	2,404	2,546	12,34	2,045	2,88	2,802	2,636	2,587	2,629	1,895	2,885	2,301
**Sum**	21,28	20,74	1,8	29,68	8,45	15,87	16,36	6,24	33,29	9,86	13,58	20,26

PG 35:1

	**Controls healthy**	**Controls Bronchitis**	**Diffuse development. disorder**	**Growth abn.+ defic. alveolar.**	**Immuno-intact host**	**Immuno-compromised host**	**Chronic tachypnea of infancy**	**Reactive lymphoid lesions**	**Related to alv. surf. region**	**Related to lung vessels/heart**	**Related to systemic disease**	**Unclear RDS in mature neonate**
**Number of values**	11	10	2	18	5	8	8	4	18	7	7	13
**Minimum**	0,43	0	0	0	0,93	0	0,34	0	0	0	0	0
**25% Percentile**	0,64	0,735		0,13	0,96	0,36	0,605	0,26	0	0,48	0,53	0
**Median**	1,39	1,365	0	0,46	1,15	0,43	0,96	0,875	0	0,5	0,8	0,34
**75% Percentile**	1,56	1,89		0,745	1,25	0,69	1,16	1,305	0,8	0,78	1,14	0,625
**Maximum**	1,92	2,78	0	1,16	1,34	0,9	1,33	1,38	4,92	2,51	1,48	0,85
**Mean**	1,209	1,323	0	0,4872	1,114	0,4825	0,89	0,7825	0,5578	0,7729	0,8086	0,3515
**Std. Deviation**	0,4855	0,7885	0	0,3396	0,161	0,2751	0,3437	0,6425	1,243	0,8032	0,4681	0,3256
**Std. Error**	0,1464	0,2493	0	0,08005	0,07201	0,09726	0,1215	0,3213	0,2931	0,3036	0,1769	0,09032
**Lower 95% CI of mean**	0,8829	0,7589	0	0,3183	0,9141	0,2525	0,6026	-0,2399	-0,0606	0,03006	0,3757	0,1548
**Upper 95% CI of mean**	1,535	1,887	0	0,6561	1,314	0,7125	1,177	1,805	1,176	1,516	1,241	0,5483
**Sum**	13,3	13,23	0	8,77	5,57	3,86	7,12	3,13	10,04	5,41	5,66	4,57

PG 36:4

	**Controls healthy**	**Controls Bronchitis**	**Diffuse development. disorder**	**Growth abn.+ defic. alveolar.**	**Immuno-intact host**	**Immuno-compromised host**	**Chronic tachypnea of infancy**	**Reactive lymphoid lesions**	**Related to alv. surf. region**	**Related to lung vessels/heart**	**Related to systemic disease**	**Unclear RDS in mature neonate**
**Number of values**	11	10	2	18	5	8	9	4	21	7	7	13
**Minimum**	1,39	1,42	0	0	1,11	0,42	0	1,78	0	0	0	0
**25% Percentile**	1,69	1,685		1,495	1,24	1,64	1,175	1,885	0	1,2	1,56	0,275
**Median**	1,97	1,89	0,55	1,81	1,85	2,66	1,97	2,115	1,55	1,82	2,17	1,93
**75% Percentile**	2,39	2,29		2,325	2,45	3,655	2,23	2,605	2,41	2,56	2,59	2,44
**Maximum**	3,14	2,56	1,1	3,01	2,89	4,47	2,42	2,97	6,03	3,01	4,34	2,99
**Mean**	2,054	1,956	0,55	1,763	1,846	2,6	1,697	2,245	1,599	1,759	2,181	1,524
**Std. Deviation**	0,5039	0,3586	0,7778	0,8675	0,6865	1,398	0,7777	0,5186	1,672	0,9901	1,301	1,098
**Std. Error**	0,1519	0,1134	0,55	0,2045	0,307	0,4942	0,2592	0,2593	0,3648	0,3742	0,4917	0,3045
**Lower 95% CI of mean**	1,715	1,699	-6,438	1,331	0,9936	1,431	1,099	1,42	0,838	0,8428	0,9783	0,8605
**Upper 95% CI of mean**	2,392	2,213	7,538	2,194	2,698	3,769	2,294	3,07	2,36	2,674	3,385	2,187
**Sum**	22,59	19,56	1,1	31,73	9,23	20,8	15,27	8,98	33,58	12,31	15,27	19,81

PG 36:3

	**Controls healthy**	**Controls Bronchitis**	**Diffuse development. disorder**	**Growth abn.+ defic. alveolar.**	**Immuno-intact host**	**Immuno-compromised host**	**Chronic tachypnea of infancy**	**Reactive lymphoid lesions**	**Related to alv. surf. region**	**Related to lung vessels/heart**	**Related to systemic disease**	**Unclear RDS in mature neonate**
**Number of values**	11	10	2	18	5	8	9	4	20	7	7	13
**Minimum**	0,8	0,98	2,64	0	1,55	2,43	0	1,29	0	1,09	2,06	0
**25% Percentile**	1,52	1,27		2,65	2,04	3,1	1,06	1,665	0	1,6	2,24	0,425
**Median**	2,05	2,105	3,17	3,98	2,54	4,23	2,15	3,335	0,685	3,71	2,63	3,59
**75% Percentile**	3,29	3,23		5,265	3,12	4,84	3,3	5,145	4,59	5,15	3,74	4,76

**Maximum**	6,74	7,16	3,7	7,26	3,5	6,28	4,82	5,66	25,5	10,2	5,91	6,42
**Mean**	2,802	2,517	3,17	3,901	2,572	4,131	2,217	3,405	3,549	3,927	3,106	2,955
**Std. Deviation**	1,964	1,828	0,7495	1,785	0,6958	1,261	1,479	2,075	6,117	3,111	1,352	2,208
**Std. Error**	0,5921	0,5779	0,53	0,4208	0,3112	0,4459	0,493	1,038	1,368	1,176	0,5108	0,6125
**Lower 95% CI of mean**	1,483	1,21	-3,564	3,013	1,708	3,077	1,08	0,1025	0,686	1,05	1,856	1,62
**Upper 95% CI of mean**	4,121	3,824	9,904	4,788	3,436	5,186	3,353	6,707	6,412	6,804	4,356	4,289
**Sum**	30,82	25,17	6,34	70,21	12,86	33,05	19,95	13,62	70,98	27,49	21,74	38,41

PG 36:2

	**Controls healthy**	**Controls Bronchitis**	**Diffuse development. disorder**	**Growth abn.+ defic. alveolar.**	**Immuno-intact host**	**Immuno-compromised host**	**Chronic tachypnea of infancy**	**Reactive lymphoid lesions**	**Related to alv. surf. region**	**Related to lung vessels/heart**	**Related to systemic disease**	**Unclear RDS in mature neonate**
**Number of values**	11	10	2	18	5	8	9	4	20	7	7	13
**Minimum**	10,99	7,83	4,24	2,3	13,23	7,93	9,1	11,59	0	4,44	10,46	1,1
**25% Percentile**	12,71	10,68		10,82	13,75	14,87	12,25	11,6	2,37	11,59	11	7,09
**Median**	17,42	13,25	9,005	18,83	16,12	16,72	17,45	16,48	7,81	18,44	12,71	17,38
**75% Percentile**	19,84	21,42		25,33	20,46	21,55	25,26	22,98	15,92	22,2	13,73	22,08
**Maximum**	33,71	22,42	13,77	29,17	21,79	31,28	37,18	24,6	21,96	40,43	17,98	28,47
**Mean**	18,09	15,34	9,005	18,21	16,9	18,18	19,28	17,29	8,828	18,8	13,03	15,38
**Std. Deviation**	6,325	5,495	6,739	7,998	3,532	6,944	8,84	6,703	7,522	11,2	2,46	8,806
**Std. Error**	1,907	1,738	4,765	1,885	1,579	2,455	2,947	3,351	1,682	4,234	0,9297	2,442
**Lower 95% CI of mean**	13,84	11,41	-51,54	14,23	12,52	12,38	12,49	6,619	5,307	8,439	10,75	10,06
**Upper 95% CI of mean**	22,34	19,27	69,55	22,19	21,29	23,99	26,08	27,95	12,35	29,16	15,3	20,7
**Sum**	199	153,4	18,01	327,8	84,52	145,5	173,5	69,14	176,6	131,6	91,18	199,9

PG 36:1

	**Controls healthy**	**Controls Bronchitis**	**Diffuse development. disorder**	**Growth abn.+ defic. alveolar.**	**Immuno-intact host**	**Immuno-compromised host**	**Chronic tachypnea of infancy**	**Reactive lymphoid lesions**	**Related to alv. surf. region**	**Related to lung vessels/heart**	**Related to systemic disease**	**Unclear RDS in mature neonate**
**Number of values**	11	10	2	18	5	8	9	4	20	7	7	13
**Minimum**	11,88	9,44	4,42	1,81	13,16	8,14	13,48	9,96	0	5,82	7,37	3,17
**25% Percentile**	13,5	11,95		8,2	13,47	11,29	16,73	11,43	2,055	11,68	8,58	6,7
**Median**	19,08	18,46	5,39	11,45	17,21	13,3	18,22	13,09	6,35	13,83	11,91	9,32
**75% Percentile**	20,14	20,66		12,83	21,44	14,01	20,26	14,44	10,2	16	12,94	12,05
**Maximum**	22,85	21,5	6,36	14,93	24,23	14,22	22,85	15,61	16,32	17,22	20,1	13,31
**Mean**	17,24	16,9	5,39	10,6	17,4	12,44	18,25	12,94	6,32	13,29	11,89	9,05
**Std. Deviation**	4	4,348	1,372	3,245	4,453	2,111	2,799	2,318	5,124	3,737	4,161	3,323
**Std. Error**	1,206	1,375	0,97	0,7648	1,991	0,7463	0,9331	1,159	1,146	1,413	1,573	0,9217
**Lower 95% CI of mean**	14,55	13,79	-6,935	8,982	11,88	10,68	16,1	9,246	3,922	9,832	8,043	7,042
**Upper 95% CI of mean**	19,93	20,01	17,72	12,21	22,93	14,21	20,4	16,62	8,718	16,75	15,74	11,06
**Sum**	189,7	169	10,78	190,7	87,02	99,54	164,3	51,74	126,4	93,02	83,24	117,7

PG 38:6

	**Controls healthy**	**Controls Bronchitis**	**Diffuse development. disorder**	**Growth abn.+ defic. alveolar.**	**Immuno-intact host**	**Immuno-compromised host**	**Chronic tachypnea of infancy**	**Reactive lymphoid lesions**	**Related to alv. surf. region**	**Related to lung vessels/heart**	**Related to systemic disease**	**Unclear RDS in mature neonate**
**Number of values**	11	10	2	18	5	8	8	4	17	7	7	13
**Minimum**	0,21	0	0	0	0	0	0,28	0,46	0	0	0	0
**25% Percentile**	0,48	0,27		0,14	0,15	0,33	0,405	0,705	0	0,18	0	0
**Median**	0,89	0,585	1,345	0,83	0,43	0,62	0,525	0,96	0	0,38	0,48	0,61
**75% Percentile**	1,15	0,94		1,075	0,715	0,83	0,84	0,99	0,545	0,73	0,99	0,91
**Maximum**	1,36	1,39	2,69	1,38	0,97	1,11	1,58	1,01	2,52	0,82	1,12	1,17
**Mean**	0,8182	0,604	1,345	0,7006	0,432	0,5838	0,675	0,8475	0,3535	0,4186	0,5286	0,4569
**Std. Deviation**	0,3614	0,4079	1,902	0,4658	0,3515	0,3676	0,42	0,2595	0,6661	0,2873	0,4365	0,471
**Std. Error**	0,109	0,129	1,345	0,1098	0,1572	0,13	0,1485	0,1298	0,1616	0,1086	0,165	0,1306
**Lower 95% CI of mean**	0,5754	0,3122	-15,74	0,4689	-0,004478	0,2764	0,3238	0,4345	0,01103	0,1529	0,1249	0,1723
**Upper 95% CI of mean**	1,061	0,8958	18,43	0,9322	0,8685	0,8911	1,026	1,26	0,696	0,6842	0,9323	0,7416
**Sum**	9	6,04	2,69	12,61	2,16	4,67	5,4	3,39	6,01	2,93	3,7	5,94

PG 38:5

	**Controls healthy**	**Controls Bronchitis**	**Diffuse development. disorder**	**Growth abn.+ defic. alveolar.**	**Immuno-intact host**	**Immuno-compromised host**	**Chronic tachypnea of infancy**	**Reactive lymphoid lesions**	**Related to alv. surf. region**	**Related to lung vessels/heart**	**Related to systemic disease**	**Unclear RDS in mature neonate**
**Number of values**	11	10	2	18	5	8	9	4	21	7	7	13
**Minimum**	0,99	0,76	0	0	0,88	0	0	1,36	0	0	0,41	0
**25% Percentile**	1,21	0,925		0,885	1,175	1,59	0,685	1,415	0	0,58	0,92	0
**Median**	1,47	1,275	0,57	1,49	1,74	2,325	1,33	1,74	0,94	0,98	1,1	1,36
**75% Percentile**	1,64	1,805		1,985	2,085	2,83	1,77	2,125	1,855	1,97	1,88	1,65
**Maximum**	2,07	6,62	1,14	2,73	2,28	3,38	2,55	2,24	6,7	2,2	1,96	2,44
**Mean**	1,517	1,78	0,57	1,431	1,652	2,109	1,239	1,77	1,347	1,14	1,297	0,9392
**Std. Deviation**	0,3244	1,75	0,8061	0,7505	0,5215	1,076	0,7586	0,4229	1,9	0,7749	0,6009	0,9445
**Std. Error**	0,0978	0,5533	0,57	0,1769	0,2332	0,3805	0,2529	0,2115	0,4147	0,2929	0,2271	0,2619

**Lower 95% CI of mean**	1,299	0,5284	-6,673	1,057	1,004	1,209	0,6557	1,097	0,4821	0,4233	0,7414	0,3685
**Upper 95% CI of mean**	1,735	3,032	7,813	1,804	2,3	3,008	1,822	2,443	2,212	1,857	1,853	1,51
**Sum**	16,69	17,8	1,14	25,75	8,26	16,87	11,15	7,08	28,29	7,98	9,08	12,21

PG 38:4

	Controls healthy	Controls Bronchitis	Diffuse development. disorder	Growth abn.+ defic. alveolar.	Immuno-intact host	Immuno-compromised host	Chronic tachypnea of infancy	Reactive lymphoid lesions	Related to alv. surf. region	Related to lung vessels/heart	Related to systemic disease	Unclear RDS in mature neonate
**Number of values**	11	10	2	18	5	8	9	4	21	7	7	13
**Minimum**	0,7	0	0	0	0,98	1,28	0	1,13	0	0	1,04	0
**25% Percentile**	1,12	1,15		0,59	1,18	1,715	1,035	1,43	0	1,12	1,06	0,53
**Median**	1,76	1,525	0	0,955	1,87	2,355	1,64	1,79	0,44	1,31	1,6	1,11
**75% Percentile**	1,97	2,035		1,535	2,365	3,345	1,74	1,98	1,33	1,74	2,24	1,66
**Maximum**	2,52	2,24	0	2,37	2,82	3,99	2,35	2,11	2,45	1,82	2,33	2,04
**Mean**	1,609	1,466	0	1,011	1,792	2,513	1,42	1,705	0,6648	1,234	1,694	1,092
**Std. Deviation**	0,5732	0,648	0	0,6503	0,6903	0,9702	0,6668	0,4148	0,8058	0,6012	0,5343	0,5969
**Std. Error**	0,1728	0,2049	0	0,1533	0,3087	0,343	0,2223	0,2074	0,1758	0,2272	0,2019	0,1656
**Lower 95% CI of mean**	1,224	1,002	0	0,6872	0,9349	1,701	0,9075	1,045	0,2979	0,6782	1,2	0,7308
**Upper 95% CI of mean**	1,994	1,93	0	1,334	2,649	3,324	1,933	2,365	1,032	1,79	2,188	1,452
**Sum**	17,7	14,66	0	18,19	8,96	20,1	12,78	6,82	13,96	8,64	11,86	14,19

LPC 15:0

	Controls healthy	Controls Bronchitis	Diffuse development. disorder	Growth abn. + alveolar. defic.	Immuno-intact host	Immuno-compromised host	Chronic tachypnea of infancy	Reactive lymphoid lesions	Related to alv. surf. region	Related to lung vessels/heart	Related to systemic disease	Unclear RDS in mature neonate
**Number of values**	11	10	2	18	5	8	9	4	21	7	7	13
**Minimum**	0,54	0,49	0,7	0,33	1,17	0,84	0,55	1,49	0,55	0,32	0,59	0,5
**25% Percentile**	1,64	1,46		0,605	1,29	1,325	0,895	1,595	1,015	1,29	0,86	0,72
**Median**	2,04	1,995	1,725	1,27	1,73	2,115	1,23	2,04	2,09	1,8	1,92	1,11
**75% Percentile**	2,91	2,94		2,75	2,49	2,3	1,985	3,21	3,05	2,58	2,1	1,975
**Maximum**	3,54	4,86	2,75	6,32	2,59	5,53	2,75	4,04	11,49	2,79	3,54	3,34
**Mean**	2,133	2,275	1,725	1,813	1,858	2,231	1,451	2,403	2,47	1,729	1,719	1,469
**Std. Deviation**	0,855	1,197	1,45	1,559	0,6143	1,44	0,717	1,156	2,354	0,8324	1,007	0,9553
**Std. Error**	0,2578	0,3787	1,025	0,3676	0,2747	0,5091	0,239	0,5779	0,5136	0,3146	0,3805	0,265
**Lower 95% CI of mean**	1,558	1,418	-11,3	1,037	1,095	1,027	0,9	0,5632	1,399	0,9587	0,7874	0,8919
**Upper 95% CI of mean**	2,707	3,132	14,75	2,588	2,621	3,435	2,002	4,242	3,541	2,498	2,65	2,047
**Sum**	23,46	22,75	3,45	32,63	9,29	17,85	13,06	9,61	51,87	12,1	12,03	19,1

LPC 16:1

	Controls healthy	Controls Bronchitis	Diffuse development. disorder	Growth abn. + alveolar. defic.	Immuno-intact host	Immuno-compromised host	Chronic tachypnea of infancy	Reactive lymphoid lesions	Related to alv. surf. region	Related to lung vessels/heart	Related to systemic disease	Unclear RDS in mature neonate
**Number of values**	11	10	2	18	5	8	9	4	21	7	7	13
**Minimum**	2,69	1,79	1,99	0	2,8	1,64	2,6	3,49	0,54	1,29	1,55	1,65
**25% Percentile**	3,04	2,18		3,195	2,93	2,075	3,635	3,705	1,42	3,56	1,97	2,15
**Median**	3,91	3,33	2,685	3,665	3,07	3,75	4,47	4,3	2,8	3,9	2,65	3,55
**75% Percentile**	4,4	3,735		5,31	3,595	4,895	5,535	5,68	3,935	9,05	3,98	4,49
**Maximum**	5,23	4,72	3,38	6,52	3,68	6,27	7,83	6,68	7,9	15,26	6,41	5,09
**Mean**	3,82	3,154	2,685	3,987	3,224	3,669	4,699	4,693	2,966	6,03	3,28	3,362
**Std. Deviation**	0,8031	0,8967	0,9829	1,54	0,3606	1,735	1,523	1,413	1,74	4,705	1,643	1,219
**Std. Error**	0,2421	0,2835	0,695	0,3631	0,1613	0,6134	0,5076	0,7067	0,3796	1,778	0,621	0,3382
**Lower 95% CI of mean**	3,28	2,513	-6,146	3,221	2,776	2,218	3,528	2,443	2,174	1,679	1,76	2,625
**Upper 95% CI of mean**	4,36	3,795	11,52	4,753	3,672	5,119	5,869	6,942	3,758	10,38	4,8	4,098
**Sum**	42,02	31,54	5,37	71,76	16,12	29,35	42,29	18,77	62,29	42,21	22,96	43,7

LPC 16:0

	Controls healthy	Controls Bronchitis	Diffuse development. disorder	Growth abn. + alveolar. defic.	Immuno-intact host	Immuno-compromised host	Chronic tachypnea of infancy	Reactive lymphoid lesions	Related to alv. surf. region	Related to lung vessels/heart	Related to systemic disease	Unclear RDS in mature neonate
**Number of values**	11	10	2	18	5	8	9	4	21	7	7	13
**Minimum**	38,22	47,78	46,34	12,86	49,98	38,34	45,94	37,34	21,67	34,77	45,15	35,43
**25% Percentile**	44,11	51,19		43,15	51,19	45,88	47,14	37,57	42,12	41,86	51,01	49,13
**Median**	49,01	59,15	61,55	54,75	54,46	57,48	54,87	44,44	53,66	50,29	59,96	54,94
**75% Percentile**	59,42	63,81		59,85	56,33	61,83	59,81	56,93	66,51	56,11	64,3	65,77
**Maximum**	67,63	78,74	76,75	80,97	57,07	65,07	60,78	62,78	80,94	61,99	79,47	70,42
**Mean**	49,93	59,47	61,55	50,53	53,9	54,22	54,2	47,25	52,69	49,85	59,53	55,26
**Std. Deviation**	8,98	8,823	21,5	15,95	2,776	9,881	6,057	12,16	15,56	9,069	11,08	10,5
**Std. Error**	2,707	2,79	15,21	3,76	1,242	3,493	2,019	6,078	3,396	3,428	4,189	2,911
**Lower 95% CI of mean**	43,9	53,15	-131,7	42,6	50,45	45,96	49,55	27,91	45,6	41,46	49,28	48,91
**Upper 95% CI of mean**	55,96	65,78	254,7	58,47	57,35	62,48	58,86	66,59	59,77	58,24	69,78	61,6
**Sum**	549,2	594,7	123,1	909,6	269,5	433,8	487,8	189	1106	349	416,7	718,3

LPC 18:2

	Controls healthy	Controls Bronchitis	Diffuse development. disorder	Growth abn. + alveolar. defic.	Immuno-intact host	Immuno-compromised host	Chronic tachypnea of infancy	Reactive lymphoid lesions	Related to alv. surf. region	Related to lung vessels/heart	Related to systemic disease	Unclear RDS in mature neonate
**Number of values**	11	10	2	18	5	8	9	4	21	7	7	13
**Minimum**	1,47	0,99	1,1	0	2,48	1,07	2,81	3,22	0,64	1,21	1,47	0,73
**25% Percentile**	3,42	1,525		2,525	2,77	1,61	4,325	3,565	1,13	2,17	1,51	1,98
**Median**	4,84	2,99	1,205	2,95	3,75	1,98	4,84	4,27	2,01	5,24	2,47	2,61
**75% Percentile**	6,77	4,09		4,795	4,4	2,95	6,185	6,61	4,965	7,66	3,8	4,13
**Maximum**	9,53	5,01	1,31	17,31	4,85	3,88	6,62	8,59	14,31	9,88	4,88	5,15
**Mean**	4,878	2,881	1,205	3,987	3,618	2,254	5,027	5,088	3,189	5,129	2,841	2,937
**Std. Deviation**	2,324	1,382	0,1485	3,646	0,9016	0,9807	1,202	2,405	3,161	3,057	1,283	1,288
**Std. Error**	0,7008	0,4371	0,105	0,8595	0,4032	0,3467	0,4008	1,202	0,6897	1,155	0,4849	0,3574
**Lower 95% CI of mean**	3,317	1,892	-0,1291	2,174	2,498	1,434	4,103	1,261	1,75	2,301	1,655	2,158
**Upper 95% CI of mean**	6,44	3,87	2,539	5,801	4,738	3,074	5,951	8,914	4,627	7,956	4,028	3,716
**Sum**	53,66	28,81	2,41	71,77	18,09	18,03	45,24	20,35	66,96	35,9	19,89	38,18

LPC 18:1

	Controls healthy	Controls Bronchitis	Diffuse development. disorder	Growth abn. + alveolar. defic.	Immuno-intact host	Immuno-compromised host	Chronic tachypnea of infancy	Reactive lymphoid lesions	Related to alv. surf. region	Related to lung vessels/heart	Related to systemic disease	Unclear RDS in mature neonate
**Number of values**	11	10	2	18	5	8	9	4	21	7	7	13

**Minimum**	5,61	4,39	3,61	4,5	5,98	4,22	7,2	6,25	3,25	8,75	5,26	5,63
**25% Percentile**	6,85	6,11		6,76	6,05	5,215	8,665	6,505	5,54	9,93	6,51	6,435
**Median**	7,85	7,215	5,945	9,995	8,24	8,16	9,95	6,83	6,2	11,58	9,06	9,34
**75% Percentile**	10,81	10,66		14,61	9,33	11,37	11,86	9,005	9,12	17,27	10,53	13,04
**Maximum**	14,66	12,46	8,28	20,1	9,78	17,26	15,97	11,11	17,87	19,74	13,02	16,68
**Mean**	8,759	7,994	5,945	10,77	7,8	8,87	10,41	7,755	7,593	13,48	8,681	9,697
**Std. Deviation**	2,926	2,684	3,302	4,693	1,689	4,523	2,619	2,254	3,243	4,42	2,682	3,744
**Std. Error**	0,8822	0,8488	2,335	1,106	0,7555	1,599	0,8731	1,127	0,7076	1,671	1,014	1,038
**Lower 95% CI of mean**	6,793	6,074	-23,72	8,434	5,702	5,089	8,393	4,168	6,117	9,394	6,201	7,434
**Upper 95% CI of mean**	10,72	9,914	35,61	13,1	9,898	12,65	12,42	11,34	9,069	17,57	11,16	11,96
**Sum**	96,35	79,94	11,89	193,8	39	70,96	93,66	31,02	159,5	94,37	60,77	126,1

LPC 18:0

	**Controls healthy**	**Controls Bronchitis**	**Diffuse development. disorder**	**Growth abn. + alveolar. defic.**	**Immuno-intact host**	**Immuno-compromised host**	**Chronic tachypnea of infancy**	**Reactive lymphoid lesions**	**Related to alv. surf. region**	**Related to lung vessels/heart**	**Related to systemic disease**	**Unclear RDS in mature neonate**
**Number of values**	11	10	2	18	5	8	9	4	21	7	7	13
**Minimum**	7	9,65	7,91	6,38	11,01	7,78	7,71	10,32	5,9	5,07	8,47	8,89
**25% Percentile**	9,53	10,5		8,885	12,1	8,935	11,8	11,35	11,75	6,45	9,15	11,41
**Median**	10,84	11,78	11,64	12,14	13,34	13,87	12,2	12,61	13,03	11,16	10,65	12,75
**75% Percentile**	16,16	13,75		13,65	15,68	17,39	14,84	13,55	20,98	12,86	15,73	16,32
**Maximum**	16,43	15,86	15,36	21,18	16,46	38,08	17,3	14,25	37,03	24,69	22,43	24,23
**Mean**	12,1	12,09	11,64	12,19	13,78	15,78	12,84	12,45	15,97	11,82	12,82	14,18
**Std. Deviation**	3,318	1,964	5,268	3,847	2,039	9,822	2,706	1,626	7,731	6,356	4,975	4,27
**Std. Error**	1	0,6211	3,725	0,9067	0,9118	3,473	0,9021	0,8132	1,687	2,402	1,88	1,184
**Lower 95% CI of mean**	9,867	10,68	-35,7	10,27	11,25	7,569	10,76	9,857	12,45	5,937	8,223	11,6
**Upper 95% CI of mean**	14,33	13,49	58,97	14,1	16,31	23,99	14,92	15,03	19,49	17,69	17,43	16,76
**Sum**	133,1	120,9	23,27	219,4	68,89	126,2	115,6	49,78	335,4	82,71	89,77	184,3

LPC 20:4

	**Controls healthy**	**Controls Bronchitis**	**Diffuse development. disorder**	**Growth abn. + alveolar. defic.**	**Immuno-intact host**	**Immuno-compromised host**	**Chronic tachypnea of infancy**	**Reactive lymphoid lesions**	**Related to alv. surf. region**	**Related to lung vessels/heart**	**Related to systemic disease**	**Unclear RDS in mature neonate**
**Number of values**	11	10	2	20	5	8	9	4	21	7	7	13
**Minimum**	0,16	0,27	0,29	0	1,46	0,99	0,99	1,01	0,18	0,7	0,2	0,32
**25% Percentile**	1,39	1,05		0,825	1,52	1,12	1,09	1,92	0,63	1,21	0,67	0,585
**Median**	1,68	1,675	1,19	1,18	1,72	1,365	1,28	2,97	0,97	2,03	1,4	1,81
**75% Percentile**	3,79	2,435		1,585	2,82	1,94	2,145	3,87	1,705	2,27	1,58	2,31
**Maximum**	3,98	2,85	2,09	3,45	2,86	3,52	2,41	4,63	3,33	3,87	2,08	5,1
**Mean**	2,216	1,668	1,19	1,369	2,08	1,67	1,567	2,895	1,278	1,967	1,263	1,936
**Std. Deviation**	1,307	0,7877	1,273	0,9483	0,6823	0,8512	0,5516	1,485	0,9049	1,004	0,6259	1,487
**Std. Error**	0,3942	0,2491	0,9	0,212	0,3052	0,301	0,1839	0,7424	0,1975	0,3794	0,2366	0,4123
**Lower 95% CI of mean**	1,338	1,105	-10,25	0,9247	1,233	0,9584	1,143	0,5323	0,8662	1,039	0,684	1,038
**Upper 95% CI of mean**	3,095	2,231	12,63	1,812	2,927	2,382	1,991	5,258	1,69	2,895	1,842	2,835
**Sum**	24,38	16,68	2,38	27,37	10,4	13,36	14,1	11,58	26,84	13,77	8,84	25,17

LPC 20:3

	**Controls healthy**	**Controls Bronchitis**	**Diffuse development. disorder**	**Growth abn. + alveolar. defic.**	**Immuno-intact host**	**Immuno-compromised host**	**Chronic tachypnea of infancy**	**Reactive lymphoid lesions**	**Related to alv. surf. region**	**Related to lung vessels/heart**	**Related to systemic disease**	**Unclear RDS in mature neonate**
**Number of values**	11	10	2	18	5	8	9	4	21	7	7	13
**Minimum**	0	0,11	0	0	0,38	0,34	0	0,35	0	0,2	0,25	0
**25% Percentile**	0	0,36		0,305	0,425	0,395	0,145	0,505	0	0,4	0,32	0,255
**Median**	0,52	0,455	0,095	0,46	0,57	0,555	0,33	0,66	0,32	0,5	0,73	0,5
**75% Percentile**	0,85	0,6		0,595	0,695	0,645	0,64	0,825	0,695	0,79	0,76	0,69
**Maximum**	1,01	1,01	0,19	1,49	0,76	0,98	0,79	0,99	1,5	0,82	0,99	1,19
**Mean**	0,46	0,49	0,095	0,4861	0,562	0,5638	0,3911	0,665	0,4129	0,5529	0,6343	0,5038
**Std. Deviation**	0,3826	0,2338	0,1344	0,3465	0,1462	0,2072	0,2782	0,2613	0,4382	0,2372	0,2607	0,3121
**Std. Error**	0,1154	0,07394	0,095	0,08167	0,06538	0,07326	0,09274	0,1307	0,09562	0,08965	0,09853	0,08655
**Lower 95% CI of mean**	0,203	0,3227	-1,112	0,3138	0,3805	0,3905	0,1772	0,2491	0,2134	0,3335	0,3932	0,3153
**Upper 95% CI of mean**	0,717	0,6573	1,302	0,6584	0,7435	0,737	0,605	1,081	0,6123	0,7722	0,8754	0,6924
**Sum**	5,06	4,9	0,19	8,75	2,81	4,51	3,52	2,66	8,67	3,87	4,44	6,55

LPC 20:0

	**Controls healthy**	**Controls Bronchitis**	**Diffuse development. disorder**	**Growth abn. + alveolar. defic.**	**Immuno-intact host**	**Immuno-compromised host**	**Chronic tachypnea of infancy**	**Reactive lymphoid lesions**	**Related to alv. surf. region**	**Related to lung vessels/heart**	**Related to systemic disease**	**Unclear RDS in mature neonate**
**Number of values**	11	10	2	18	5	8	9	4	21	7	7	13
**Minimum**	0,88	0,17	0,13	0,1	0,83	0,36	0	1,12	0,09	0,59	0,27	0,26
**25% Percentile**	1,02	0,57		0,54	0,935	0,91	0,675	1,27	0,5	0,61	0,35	0,48
**Median**	1,56	0,985	1,65	1,26	1,11	1,195	1	1,505	1,03	1,06	0,78	1,1
**75% Percentile**	2,68	1,79		2,675	1,385	1,43	2,245	2,375	2,365	1,91	1,15	1,82

**Maximum**	3,31	2,35	3,17	9,46	1,45	2,17	2,85	3,16	7,22	2,65	1,98	3,39
**Mean**	1,835	1,122	1,65	1,957	1,15	1,2	1,35	1,823	1,65	1,269	0,8686	1,298
**Std. Deviation**	0,9394	0,6785	2,15	2,222	0,2424	0,5377	0,951	0,9126	1,772	0,7593	0,5726	0,9705
**Std. Error**	0,2832	0,2146	1,52	0,5238	0,1084	0,1901	0,317	0,4563	0,3867	0,287	0,2164	0,2692
**Lower 95% CI of mean**	1,204	0,6366	-17,66	0,8522	0,849	0,7505	0,619	0,3704	0,8439	0,5663	0,339	0,7112
**Upper 95% CI of mean**	2,467	1,607	20,96	3,062	1,451	1,65	2,081	3,275	2,457	1,971	1,398	1,884
**Sum**	20,19	11,22	3,3	35,23	5,75	9,6	12,15	7,29	34,66	8,88	6,08	16,87

LPC 22:6

	Controls healthy	Controls Bronchitis	Diffuse development. disorder	Growth abn. + alveolar. defic.	Immuno-intact host	Immuno-compromised host	Chronic tachypnea of infancy	Reactive lymphoid lesions	Related to alv. surf. region	Related to lung vessels/heart	Related to systemic disease	Unclear RDS in mature neonate
**Number of values**	11	10	2	18	5	8	9	4	21	7	7	13
**Minimum**	0	0,13	0,16	0	0,36	0,39	0	0,42	0	0,2	0,08	0
**25% Percentile**	0,41	0,245		0,44	0,43	0,425	0,43	0,575	0,185	0,25	0,24	0,325
**Median**	0,65	0,55	0,715	0,54	0,54	0,505	0,64	0,885	0,47	0,57	0,38	0,56
**75% Percentile**	0,9	0,775		0,775	0,635	0,67	0,715	1,075	0,94	0,92	0,74	0,8
**Maximum**	1,14	1,02	1,27	1,54	0,66	0,88	1	1,11	1,75	1,09	1,11	1,02
**Mean**	0,6055	0,537	0,715	0,6561	0,534	0,5588	0,5689	0,825	0,5533	0,5929	0,4586	0,5477
**Std. Deviation**	0,3438	0,3002	0,7849	0,4208	0,1152	0,177	0,2752	0,3165	0,4678	0,3279	0,3528	0,3018
**Std. Error**	0,1036	0,09494	0,555	0,09919	0,05154	0,06258	0,09172	0,1582	0,1021	0,1239	0,1333	0,08371
**Lower 95% CI of mean**	0,3745	0,3222	-6,337	0,4468	0,3909	0,4108	0,3574	0,3214	0,3404	0,2896	0,1323	0,3653
**Upper 95% CI of mean**	0,8364	0,7518	7,767	0,8654	0,6771	0,7067	0,7804	1,329	0,7663	0,8961	0,7848	0,7301
**Sum**	6,66	5,37	1,43	11,81	2,67	4,47	5,12	3,3	11,62	4,15	3,21	7,12

LPC 22:4

	Controls healthy	Controls Bronchitis	Diffuse development. disorder	Growth abn. + alveolar. defic.	Immuno-intact host	Immuno-compromised host	Chronic tachypnea of infancy	Reactive lymphoid lesions	Related to alv. surf. region	Related to lung vessels/heart	Related to systemic disease	Unclear RDS in mature neonate
**Number of values**	11	10	2	18	5	8	9	4	21	7	7	13
**Minimum**	0,1	0,08	0,3	0,08	1,31	0,62	0,43	1,26	0	0,12	0,27	0,13
**25% Percentile**	1,07	0,765		0,255	1,48	0,84	0,715	1,65	0,215	0,41	0,58	0,295
**Median**	1,31	1,1	1,725	1,155	2,46	2,08	0,92	2,285	1,08	1,09	2,13	1,08
**75% Percentile**	3,92	1,76		1,89	3,295	2,855	1,495	3,06	1,565	2,05	2,69	2,97
**Maximum**	4,78	2,64	3,15	6,15	3,92	6,91	3,22	3,59	4,84	3,01	3,23	7,8
**Mean**	2,135	1,225	1,725	1,535	2,402	2,385	1,2	2,355	1,207	1,237	1,76	1,947
**Std. Deviation**	1,603	0,7227	2,015	1,587	1,017	2,054	0,8661	0,9754	1,213	0,9937	1,196	2,231
**Std. Error**	0,4834	0,2285	1,425	0,374	0,4548	0,7263	0,2887	0,4877	0,2647	0,3756	0,4521	0,6187
**Lower 95% CI of mean**	1,058	0,708	-16,38	0,746	1,139	0,6675	0,5342	0,8029	0,6544	0,3181	0,6537	0,5988
**Upper 95% CI of mean**	3,213	1,742	19,83	2,324	3,665	4,103	1,866	3,907	1,759	2,156	2,866	3,295
**Sum**	23,49	12,25	3,45	27,63	12,01	19,08	10,8	9,42	25,34	8,66	12,32	25,31

Cer 16:0

	Controls healthy	Controls Bronchitis	Diffuse development. disorder	Growth abn.+ alveolar. defic.	Immuno-intact host	Immuno-compromised host	Chronic tachypnea of infancy	Reactive lymphoid lesions	Related to alv. surf. region	Related to lung vessels/heart	Related to systemic disease	Unclear RDS in mature neonate
**Number of values**	11	10	2	18	5	8	9	4	21	7	7	13
**Minimum**	31,11	31,97	46,18	24,37	37,82	35,3	35,65	34,53	10,66	37,52	21,07	35,34
**25% Percentile**	33,73	37,47		41,84	38,55	39,83	39,34	34,83	35,57	37,69	29,22	40,11
**Median**	39,97	44,7	47,2	48,16	41,19	42,91	41,87	37,17	45,68	40,39	36,71	52,53
**75% Percentile**	42,45	54,46		53,34	49,08	48,25	45,11	39,53	52,8	52,28	47,22	54,83
**Maximum**	48,12	57,26	48,22	58,56	52,53	50	48,84	39,83	57,96	58,97	54,87	58,48
**Mean**	39,07	45,39	47,2	46,51	43,29	43,41	42,35	37,18	42,86	44,66	37,73	49,2
**Std. Deviation**	5,604	9,106	1,442	8,619	5,944	5,227	3,972	2,736	11,74	8,323	11,25	8,305
**Std. Error**	1,69	2,88	1,02	2,031	2,658	1,848	1,324	1,368	2,562	3,146	4,253	2,304
**Lower 95% CI of mean**	35,3	38,88	34,24	42,22	35,91	39,04	39,3	32,82	37,51	36,97	27,32	44,18
**Upper 95% CI of mean**	42,83	51,91	60,16	50,79	50,67	47,78	45,41	41,53	48,2	52,36	48,13	54,22
**Sum**	429,7	453,9	94,4	837,1	216,4	347,3	381,2	148,7	900	312,6	264,1	639,6

Cer 18:0

	Controls healthy	Controls Bronchitis	Diffuse development. disorder	Growth abn.+ alveolar. defic.	Immuno-intact host	Immuno-compromised host	Chronic tachypnea of infancy	Reactive lymphoid lesions	Related to alv. surf. region	Related to lung vessels/heart	Related to systemic disease	Unclear RDS in mature neonate
**Number of values**	11	10	2	18	5	8	9	4	21	7	7	13
**Minimum**	5,86	2,1	21,63	8,27	12,05	7,73	7,5	7,18	2,66	5,22	7,23	8,6
**25% Percentile**	13,32	8,245		13,18	12,24	8,475	11,79	8,87	7,145	8,49	7,38	12,59
**Median**	15,03	12,95	22,19	16,81	13	10,79	15,19	13,13	15,96	12,73	8,5	16,78
**75% Percentile**	17,69	15,83		18,77	19,25	12,88	18,65	18,34	18,88	19,52	13	19,28
**Maximum**	20,65	16,22	22,75	23,44	21,41	13,48	20,02	20,98	29,22	21,07	17,34	23,81
**Mean**	15,1	11,52	22,19	16,31	15,2	10,69	15,07	13,6	13,81	13,41	10,26	16,24
**Std. Deviation**	4,157	4,632	0,792	3,873	4,018	2,274	4,134	6,036	7,343	5,922	3,698	4,171
**Std. Error**	1,253	1,465	0,56	0,9128	1,797	0,804	1,378	3,018	1,602	2,238	1,398	1,157
**Lower 95% CI of mean**	12,3	8,202	15,07	14,39	10,21	8,784	11,89	3,998	10,47	7,932	6,844	13,72
**Upper 95% CI of mean**	17,89	14,83	29,31	18,24	20,18	12,59	18,25	23,21	17,16	18,89	13,68	18,76
**Sum**	166,1	115,2	44,38	293,7	75,98	85,48	135,7	54,41	290,1	93,86	71,85	211,1

Cer 20:0

	Controls healthy	Controls Bronchitis	Diffuse development. disorder	Growth abn.+ alveolar. defic.	Immuno-intact host	Immuno-compromised host	Chronic tachypnea of infancy	Reactive lymphoid lesions	Related to alv. surf. region	Related to lung vessels/heart	Related to systemic disease	Unclear RDS in mature neonate
**Number of values**	11	10	2	18	5	8	9	4	21	7	7	13
**Minimum**	4,39	1,04	7,62	3,32	4,03	3,51	4,49	3,65	2,04	2,25	3,13	3,89
**25% Percentile**	5,29	4,12		5,18	4,49	4,075	5,165	3,775	4,025	4,01	3,31	4,815
**Median**	6,66	5,11	8,485	6,52	6,11	4,89	6,57	4,59	5,56	4,95	4,76	5,97
**75% Percentile**	8,51	6,16		7,825	7,745	6,06	8,255	6,01	7,345	6,58	6,64	6,855
**Maximum**	9,32	9,51	9,35	15,09	7,99	6,97	8,89	6,74	8,63	8,66	7,23	7,53
**Mean**	6,875	5,162	8,485	6,838	6,116	5,066	6,61	4,893	5,622	5,184	4,98	5,751
**Std. Deviation**	1,739	2,165	1,223	2,516	1,669	1,219	1,618	1,425	1,976	2,013	1,567	1,15
**Std. Error**	0,5243	0,6846	0,865	0,5931	0,7463	0,431	0,5393	0,7125	0,4311	0,7608	0,5922	0,319
**Lower 95% CI of mean**	5,706	3,613	-2,506	5,587	4,044	4,047	5,366	2,625	4,723	3,323	3,531	5,056
**Upper 95% CI of mean**	8,043	6,711	19,48	8,09	8,188	6,085	7,854	7,16	6,522	7,046	6,429	6,446
**Sum**	75,62	51,62	16,97	123,1	30,58	40,53	59,49	19,57	118,1	36,29	34,86	74,76

Cer 22:0

	Controls healthy	Controls Bronchitis	Diffuse development. disorder	Growth abn.+ alveolar. defic.	Immuno-intact host	Immuno-compromised host	Chronic tachypnea of infancy	Reactive lymphoid lesions	Related to alv. surf. region	Related to lung vessels/heart	Related to systemic disease	Unclear RDS in mature neonate
**Number of values**	11	10	2	18	5	8	9	4	21	7	7	13
**Minimum**	6,11	5,29	4,95	4,06	5,64	6,5	5,79	5,57	2,92	5,72	6,54	3,08
**25% Percentile**	6,66	6,095		4,8	7,34	7,5	6,665	6,565	5,125	5,83	8,18	4,275
**Median**	8,35	6,825	5,775	7,36	9,09	9,085	8,57	8,325	8,03	6,24	9,31	5,81
**75% Percentile**	10,14	10,26		8,135	11,57	9,66	9,605	9,66	10,47	9,97	12,13	7,565
**Maximum**	12,16	11,04	6,6	11,23	12,06	12,2	10,06	10,23	14,61	10,69	13,51	9,6
**Mean**	8,738	7,876	5,775	7,045	9,382	8,899	8,224	8,113	7,948	7,414	9,989	5,85
**Std. Deviation**	1,896	2,172	1,167	2,137	2,463	1,791	1,557	2,017	3,274	2,06	2,444	1,89
**Std. Error**	0,5717	0,6869	0,825	0,5037	1,101	0,6332	0,519	1,009	0,7145	0,7786	0,9239	0,5242
**Lower 95% CI of mean**	7,464	6,322	-4,708	5,982	6,324	7,401	7,028	4,902	6,457	5,509	7,728	4,708
**Upper 95% CI of mean**	10,01	9,43	16,26	8,108	12,44	10,4	9,421	11,32	9,438	9,319	12,25	6,992
**Sum**	96,12	78,76	11,55	126,8	46,91	71,19	74,02	32,45	166,9	51,9	69,92	76,05

Cer 23:0

	Controls healthy	Controls Bronchitis	Diffuse development. disorder	Growth abn.+ alveolar. defic.	Immuno-intact host	Immuno-compromised host	Chronic tachypnea of infancy	Reactive lymphoid lesions	Related to alv. surf. region	Related to lung vessels/heart	Related to systemic disease	Unclear RDS in mature neonate
**Number of values**	11	10	2	18	5	8	9	4	21	7	7	13

**Minimum**	3,06	1,5	1,38	1,05	0	1,83	1,56	0	0	1,69	1,42	1,04
**25% Percentile**	3,52	1,675		1,185	0	2,145	2,05	2,94	1,38	1,89	2,39	1,355
**Median**	5,5	2,835	1,705	2,595	1,63	2,51	2,61	6,045	2,21	2,43	3,83	1,67
**75% Percentile**	6,06	4,45		3,31	4,075	3,19	4,015	6,23	4,635	4,13	5,38	3,61
**Maximum**	7,14	9,9	2,03	11,66	5,02	4,57	5,04	6,25	13,87	5,15	5,59	5,71
**Mean**	5,123	3,587	1,705	2,955	1,956	2,761	2,982	4,585	3,467	2,976	3,944	2,453
**Std. Deviation**	1,437	2,495	0,4596	2,436	2,152	0,8872	1,145	3,061	3,153	1,3	1,607	1,549
**Std. Error**	0,4333	0,7889	0,325	0,5742	0,9624	0,3137	0,3818	1,531	0,6881	0,4915	0,6075	0,4296
**Lower 95% CI of mean**	4,157	1,802	-2,425	1,744	-0,7161	2,02	2,102	-0,286	2,031	1,773	2,458	1,517
**Upper 95% CI of mean**	6,088	5,372	5,835	4,166	4,628	3,503	3,863	9,456	4,902	4,178	5,431	3,389
**Sum**	56,35	35,87	3,41	53,19	9,78	22,09	26,84	18,34	72,8	20,83	27,61	31,89

Cer 24:1

	Controls healthy	Controls Bronchitis	Diffuse development. disorder	Growth abn.+ alveolar. defic.	Immuno-intact host	Immuno-compromised host	Chronic tachypnea of infancy	Reactive lymphoid lesions	Related to alv. surf. region	Related to lung vessels/heart	Related to systemic disease	Unclear RDS in mature neonate
**Number of values**	11	10	2	18	5	8	9	4	21	7	7	13
**Minimum**	9,89	11,71	8,78	5,93	7,41	16,14	7,17	14,14	7,94	11,06	8,6	7,72
**25% Percentile**	10,55	13,4		9,91	10,81	17,3	10,5	15,81	9,305	11,11	14,8	8,58
**Median**	13,57	14,87	10,05	12,4	16,56	20,2	12,8	17,68	13,56	15,71	19,11	10,52
**75% Percentile**	17,38	20,42		17,73	17,51	21,97	19,68	19,88	18,07	17,31	23,79	16,82
**Maximum**	25,24	31,37	11,31	31	18,36	22,35	23,58	21,87	26,68	24,69	33,12	23,77
**Mean**	14,36	16,97	10,05	13,67	14,64	19,68	14,21	17,84	13,98	15,67	19,61	12,88
**Std. Deviation**	4,563	6,093	1,789	5,871	4,303	2,545	5,471	3,166	4,841	4,686	7,835	4,949
**Std. Error**	1,376	1,927	1,265	1,384	1,924	0,8998	1,824	1,583	1,056	1,771	2,961	1,372
**Lower 95% CI of mean**	11,3	12,61	-6,028	10,75	9,297	17,55	10	12,8	11,78	11,34	12,36	9,885
**Upper 95% CI of mean**	17,43	21,33	26,12	16,58	19,98	21,8	18,41	22,88	16,19	20	26,86	15,87
**Sum**	158	169,7	20,09	246	73,2	157,4	127,9	71,36	293,7	109,7	137,3	167,4

Cer 24_0

	Controls healthy	Controls Bronchitis	Diffuse development. disorder	Growth abn.+ alveolar. defic.	Immuno-intact host	Immuno-compromised host	Chronic tachypnea of infancy	Reactive lymphoid lesions	Related to alv. surf. region	Related to lung vessels/heart	Related to systemic disease	Unclear RDS in mature neonate
**Number of values**	11	10	2	18	5	8	9	4	21	7	7	13
**Minimum**	6,8	5,34	3,77	2,46	3,87	4,58	6,93	11,48	1,84	4,91	4	2,89
**25% Percentile**	7,9	6,26		3,89	6,955	8,175	7,07	12,23	5,055	6,46	8,3	3,88
**Median**	10,76	8,56	4,605	6,2	10,24	9,31	10,37	13,52	10,46	8,92	13,84	6,07
**75% Percentile**	13,16	13,34		9,26	11,48	11,98	12,59	15,36	18,7	14,92	18,14	11,05
**Maximum**	14,2	15,6	5,44	13,04	12,06	12,5	18,48	16,66	40,95	19,29	19,85	14,77
**Mean**	10,73	9,497	4,605	6,678	9,42	9,501	10,55	13,79	12,31	10,68	13,49	7,632
**Std. Deviation**	2,518	3,715	1,181	3,185	3,201	2,666	3,715	2,185	9,617	5,089	5,785	4,258
**Std. Error**	0,7593	1,175	0,835	0,7506	1,432	0,9425	1,238	1,092	2,099	1,923	2,186	1,181
**Lower 95% CI of mean**	9,041	6,839	-6,005	5,094	5,445	7,273	7,698	10,32	7,928	5,976	8,136	5,059
**Upper 95% CI of mean**	12,42	12,15	15,21	8,261	13,39	11,73	13,41	17,27	16,68	15,39	18,84	10,21
**Sum**	118,1	94,97	9,21	120,2	47,1	76,01	94,98	55,17	258,4	74,78	94,4	99,22

HexCer 16:0

	Controls healthy	Controls Bronchitis	Diffuse development. disorder	Growth abn.+ alveolar. defic.	Immuno-intact host	Immuno-compromised host	Chronic tachypnea of infancy	Reactive lymphoid lesions	Related to alv. surf. region	Related to lung vessels/heart	Related to systemic disease	Unclear RDS in mature neonate
**Number of values**	11	10	2	18	5	8	9	4	21	7	7	13
**Minimum**	5,45	6,18	9,92	5,74	8,46	6,27	4,4	7,15	0	5,72	6,92	2,75
**25% Percentile**	5,93	6,5		7,665	10,2	7,56	6,52	7,265	6,535	6,22	7,73	6,795
**Median**	6,95	8,365	10,32	8,565	14,48	8,49	7,88	7,89	8,34	7,62	9,52	8,24
**75% Percentile**	8,22	9,02		10,57	14,83	9,955	8,34	9,985	9,515	9,7	10,14	9,005
**Maximum**	11,07	9,88	10,72	13,74	14,97	10,24	13,4	11,57	15,86	10,2	10,66	9,85
**Mean**	7,35	7,979	10,32	9,047	12,91	8,565	7,893	8,625	8,148	7,927	9,05	7,712
**Std. Deviation**	1,672	1,278	0,5657	2,179	2,767	1,437	2,428	2,037	3,138	1,787	1,333	1,932
**Std. Error**	0,5042	0,4042	0,4	0,5135	1,237	0,508	0,8093	1,019	0,6849	0,6754	0,5037	0,5359
**Lower 95% CI of mean**	6,227	7,065	5,238	7,963	9,473	7,364	6,027	5,384	6,72	6,275	7,817	6,544
**Upper 95% CI of mean**	8,473	8,893	15,4	10,13	16,34	9,766	9,76	11,87	9,577	9,58	10,28	8,879
**Sum**	80,85	79,79	20,64	162,8	64,54	68,52	71,04	34,5	171,1	55,49	63,35	100,3

HexCer 24:1

	Controls healthy	Controls Bronchitis	Diffuse development. disorder	Growth abn.+ alveolar. defic.	Immuno-intact host	Immuno-compromised host	Chronic tachypnea of infancy	Reactive lymphoid lesions	Related to alv. surf. region	Related to lung vessels/heart	Related to systemic disease	Unclear RDS in mature neonate
**Number of values**	11	11	2	18	5	8	9	4	21	7	7	13
**Minimum**	3,29	1,83	2,32	0	1,47	2,43	2,51	5,19	0	2,56	1,94	1,1
**25% Percentile**	3,79	3		1,68	3,42	4,06	2,965	5,525	2,23	3,28	4,07	1,69
**Median**	4,44	4,04	2,67	3,595	9,28	6,13	3,92	6,6	3,65	4,19	6,5	2,27
**75% Percentile**	6,22	4,67		6,025	10,57	8,635	5,465	8,05	7,605	5,89	7,44	5,075

**Maximum**	11,28	10,96	3,02	8,27	11,21	9,5	8,45	8,76	27,19	6,89	8,57	8,85
**Mean**	5,389	4,488	2,67	3,829	7,452	6,198	4,467	6,788	5,475	4,461	5,764	3,355
**Std. Deviation**	2,296	2,445	0,495	2,351	3,991	2,582	1,862	1,593	5,849	1,569	2,247	2,504
**Std. Error**	0,6923	0,7372	0,35	0,5541	1,785	0,9128	0,6206	0,7963	1,276	0,593	0,8493	0,6946
**Lower 95% CI of mean**	3,847	2,846	-1,777	2,66	2,496	4,039	3,036	4,253	2,813	3,01	3,686	1,842
**Upper 95% CI of mean**	6,932	6,131	7,117	4,998	12,41	8,356	5,898	9,322	8,138	5,912	7,842	4,869
**Sum**	59,28	49,37	5,34	68,92	37,26	49,58	40,2	27,15	115	31,23	40,35	43,62

CE 14:0

	Controls healthy	Controls Bronchitis	Diffuse development. disorder	Growth abn.+ alveol. defic.	Immuno-intact host	Immuno-compromised host	Chronic tachypnea of infancy	Reactive lymphoid lesions	Related to alv. surf. region	Related to lung vessels/heart	Related to systemic disease	Unclear RDS in mature neonate
**Number of values**	11	10	2	18	5	8	9	4	21	7	7	13
**Minimum**	1	0,86	2,02	0,65	1,45	0,44	0,91	1,23	0	0	0,43	0,98
**25% Percentile**	1,08	1		1,295	1,495	1,045	1,07	1,23	0,75	0,92	0,9	1,15
**Median**	1,22	1,29	2,21	1,415	1,68	1,265	1,3	1,29	1,16	1,13	1,21	1,46
**75% Percentile**	1,53	1,675		1,97	2,365	1,565	1,74	2,285	1,335	1,5	1,81	1,825
**Maximum**	2,12	1,7	2,4	2,24	2,9	1,99	2,98	3,22	2,47	1,91	2,36	2,42
**Mean**	1,357	1,326	2,21	1,559	1,88	1,273	1,499	1,758	1,08	1,103	1,274	1,523
**Std. Deviation**	0,3754	0,3248	0,2687	0,4353	0,5881	0,4796	0,6334	0,9766	0,6063	0,5861	0,6398	0,4174
**Std. Error**	0,1132	0,1027	0,19	0,1026	0,263	0,1696	0,2111	0,4883	0,1323	0,2215	0,2418	0,1158
**Lower 95% CI of mean**	1,105	1,094	-0,2042	1,343	1,15	0,8715	1,012	0,2034	0,804	0,5608	0,6826	1,271
**Upper 95% CI of mean**	1,609	1,558	4,624	1,776	2,61	1,673	1,986	3,312	1,356	1,645	1,866	1,775
**Sum**	14,93	13,26	4,42	28,07	9,4	10,18	13,49	7,03	22,68	7,72	8,92	19,8

CE 15:0

	Controls healthy	Controls Bronchitis	Diffuse development. disorder	Growth abn.+ alveol. defic.	Immuno-intact host	Immuno-compromised host	Chronic tachypnea of infancy	Reactive lymphoid lesions	Related to alv. surf. region	Related to lung vessels/heart	Related to systemic disease	Unclear RDS in mature neonate
**Number of values**	11	10	2	18	5	8	9	4	21	7	7	13
**Minimum**	0,65	0,43	1,58	0,39	0,86	0,51	0,93	0,86	0	0,27	0,37	0
**25% Percentile**	1,13	0,545		0,48	0,915	0,665	0,985	0,96	0,495	1,19	0,39	0,595
**Median**	1,22	1	2,17	1,095	0,98	0,765	1,67	1,5	0,73	1,35	0,59	0,75
**75% Percentile**	1,77	1,7		1,755	1,08	0,85	2,155	2,06	1,62	2,14	1,22	1,675
**Maximum**	2,29	2,3	2,76	3,11	1,15	1,1	2,88	2,18	3,33	2,82	1,58	3,36
**Mean**	1,375	1,139	2,17	1,248	0,994	0,7713	1,672	1,51	1,07	1,507	0,8	1,216
**Std. Deviation**	0,4773	0,6158	0,8344	0,7208	0,1041	0,1796	0,696	0,6478	0,888	0,8041	0,4572	1,001
**Std. Error**	0,1439	0,1947	0,59	0,1699	0,04654	0,06348	0,232	0,3239	0,1938	0,3039	0,1728	0,2777
**Lower 95% CI of mean**	1,054	0,6985	-5,327	0,8899	0,8648	0,6211	1,137	0,4793	0,6658	0,7635	0,3771	0,6111
**Upper 95% CI of mean**	1,695	1,58	9,667	1,607	1,123	0,9214	2,207	2,541	1,474	2,251	1,223	1,821
**Sum**	15,12	11,39	4,34	22,47	4,97	6,17	15,05	6,04	22,47	10,55	5,6	15,81

CE 16:1

	Controls healthy	Controls Bronchitis	Diffuse development. disorder	Growth abn.+ alveol. defic.	Immuno-intact host	Immuno-compromised host	Chronic tachypnea of infancy	Reactive lymphoid lesions	Related to alv. surf. region	Related to lung vessels/heart	Related to systemic disease	Unclear RDS in mature neonate
**Number of values**	11	10	2	18	5	8	9	4	21	7	7	13
**Minimum**	1,63	3,33	3,12	1,55	4,36	3,26	2,3	3,6	1,66	3,09	3,22	3,24
**25% Percentile**	3,09	4,25		3,76	4,69	3,68	2,885	3,715	3,305	3,19	3,63	4,33
**Median**	4,09	4,96	5,37	4,52	5,98	4,91	3,62	6,675	4,08	4,13	5,13	5,39
**75% Percentile**	5,24	6,995		5,375	6,92	5,98	4,265	9,6	5,605	5,62	6,18	7,865
**Maximum**	7,56	7,43	7,62	6,56	6,94	7,7	5,18	9,68	15,43	6,5	6,32	10,72
**Mean**	4,326	5,351	5,37	4,391	5,84	5,013	3,64	6,658	4,993	4,473	4,894	6,082
**Std. Deviation**	1,778	1,385	3,182	1,206	1,142	1,526	0,9314	3,4	2,967	1,256	1,175	2,324
**Std. Error**	0,536	0,4379	2,25	0,2843	0,5107	0,5394	0,3105	1,7	0,6475	0,4748	0,444	0,6446
**Lower 95% CI of mean**	3,132	4,36	-23,22	3,791	4,422	3,737	2,924	1,248	3,643	3,311	3,808	4,678
**Upper 95% CI of mean**	5,521	6,342	33,96	4,99	7,258	6,288	4,356	12,07	6,344	5,635	5,981	7,487
**Sum**	47,59	53,51	10,74	79,03	29,2	40,1	32,76	26,63	104,9	31,31	34,26	79,07

CE 16:0

	Controls healthy	Controls Bronchitis	Diffuse development. disorder	Growth abn.+ alveol. defic.	Immuno-intact host	Immuno-compromised host	Chronic tachypnea of infancy	Reactive lymphoid lesions	Related to alv. surf. region	Related to lung vessels/heart	Related to systemic disease	Unclear RDS in mature neonate
**Number of values**	11	10	2	18	5	8	9	4	21	7	7	13
**Minimum**	8,73	10,27	18,44	4,52	13,11	12,23	7,92	8,11	3,92	7,67	9,95	11,51
**25% Percentile**	9,37	11,17		10,42	15,17	14,48	11,46	12,02	11,12	9,92	12,86	16,1
**Median**	9,62	15,5	18,81	14,06	17,74	19	15	17,02	17,55	12,56	17,76	20,81
**75% Percentile**	12,74	19,45		17,53	18,58	20,41	20,61	20,52	35,67	13,27	29,14	24,35
**Maximum**	17,03	19,97	19,17	20,58	18,8	30,65	31,25	22,92	85,11	31,33	49,72	28,5
**Mean**	11,18	15,58	18,81	14	17,05	18,83	16,49	16,27	24,69	14,21	22,24	20,31
**Std. Deviation**	2,884	3,777	0,5162	4,272	2,28	5,776	6,959	6,173	20,69	7,811	13,62	5,263
**Std. Error**	0,8697	1,194	0,365	1,007	1,019	2,042	2,32	3,086	4,515	2,952	5,147	1,46
**Lower 95% CI of mean**	9,243	12,88	14,17	11,88	14,22	14	11,14	6,442	15,27	6,989	9,651	17,13
**Upper 95% CI of mean**	13,12	18,28	23,44	16,13	19,88	23,66	21,84	26,09	34,11	21,44	34,84	23,49
**Sum**	123	155,8	37,61	252,1	85,23	150,7	148,4	65,06	518,6	99,49	155,7	264,1

CE 18:3

	Controls healthy	Controls Bronchitis	Diffuse development. disorder	Growth abn.+ alveol. defic.	Immuno-intact host	Immuno-compromised host	Chronic tachypnea of infancy	Reactive lymphoid lesions	Related to alv. surf. region	Related to lung vessels/heart	Related to systemic disease	Unclear RDS in mature neonate
**Number of values**	11	10	2	18	5	8	9	4	21	7	7	13

**Minimum**	0	0,4	0,76	0	0,71	0,63	0	0	0	0,38	0,21	0
**25% Percentile**	0,62	0,545		0,675	0,83	0,75	0	0	0,175	0,6	0,55	0,58
**Median**	0,82	0,875	1,035	1,06	0,98	1,025	0,39	0,225	0,67	1,13	1,18	0,76
**75% Percentile**	1,03	1,5		1,6	1,465	1,11	0,715	1,39	1,69	1,51	2,09	1,215
**Maximum**	1,15	1,98	1,31	3,84	1,8	1,51	1,66	2,33	3,02	1,71	2,1	1,75
**Mean**	0,7873	1,012	1,035	1,248	1,114	0,9888	0,4744	0,695	0,95	1,099	1,161	0,8315
**Std. Deviation**	0,3146	0,5175	0,3889	0,8237	0,412	0,2852	0,5417	1,11	0,9272	0,4717	0,7379	0,54
**Std. Error**	0,09486	0,1637	0,275	0,1941	0,1842	0,1008	0,1806	0,5552	0,2023	0,1783	0,2789	0,1498
**Lower 95% CI of mean**	0,5759	0,6418	-2,459	0,8382	0,6025	0,7503	0,05805	-1,072	0,5279	0,6623	0,479	0,5052
**Upper 95% CI of mean**	0,9986	1,382	4,529	1,657	1,626	1,227	0,8908	2,462	1,372	1,535	1,844	1,158
**Sum**	8,66	10,12	2,07	22,46	5,57	7,91	4,27	2,78	19,95	7,69	8,13	10,81

CE 18:2

	**Controls healthy**	**Controls Bronchitis**	**Diffuse development. disorder**	**Growth abn.+ alveol. defic.**	**Immuno-intact host**	**Immuno-compromised host**	**Chronic tachypnea of infancy**	**Reactive lymphoid lesions**	**Related to alv. surf. region**	**Related to lung vessels/heart**	**Related to systemic disease**	**Unclear RDS in mature neonate**
**Number of values**	11	10	2	18	5	8	9	4	21	7	7	13
**Minimum**	2,38	6,75	2,75	1,69	17,49	7,34	2,86	3,94	2,1	3,62	5,16	3,24
**25% Percentile**	8,4	7,775		7,95	19,7	10,59	6,14	4,66	6,34	5,05	6,89	8,235
**Median**	11,05	17,81	7,21	15,82	23,09	17,22	11,21	5,425	11,36	9,41	15,42	10,47
**75% Percentile**	21,36	26,51		24,61	30,11	29,11	17	18,94	23,45	29,01	38,43	21,5
**Maximum**	30,32	35,04	11,67	36,9	33,36	37,05	41,54	32,4	61,1	33,14	49,95	31,43
**Mean**	14,72	17,78	7,21	16,58	24,54	19,78	13,5	11,8	15,99	16,53	22,69	14,48
**Std. Deviation**	9,078	9,985	6,307	9,948	5,956	11,07	11,78	13,75	14,36	12,94	16,85	9,391
**Std. Error**	2,737	3,158	4,46	2,345	2,664	3,915	3,927	6,876	3,133	4,889	6,368	2,605
**Lower 95% CI of mean**	8,619	10,64	-49,46	11,64	17,14	10,52	4,447	-10,09	9,453	4,565	7,109	8,802
**Upper 95% CI of mean**	20,82	24,93	63,88	21,53	31,93	29,03	22,56	33,68	22,52	28,49	38,27	20,15
**Sum**	161,9	177,8	14,42	298,5	122,7	158,2	121,5	47,19	335,7	115,7	158,8	188,2

CE 18:1

	**Controls healthy**	**Controls Bronchitis**	**Diffuse development. disorder**	**Growth abn.+ alveol. defic.**	**Immuno-intact host**	**Immuno-compromised host**	**Chronic tachypnea of infancy**	**Reactive lymphoid lesions**	**Related to alv. surf. region**	**Related to lung vessels/heart**	**Related to systemic disease**	**Unclear RDS in mature neonate**
**Number of values**	11	10	2	18	5	8	9	4	21	7	7	13
**Minimum**	4,99	9,13	7,33	2,99	12,42	12,79	6,37	7,63	3,39	9,11	11,89	11,86
**25% Percentile**	7,83	10,36		9,745	14,73	15,77	7,4	8,305	8,965	11,12	13,61	15,17
**Median**	10,65	18,35	10,74	14,68	17,84	19,39	9,51	12,22	15,65	15,78	16,93	18
**75% Percentile**	12,55	27,33		21,03	19,07	21,08	18,12	21,84	17,31	17,15	19,61	23,22
**Maximum**	23,25	37,13	14,15	26,04	19,96	21,61	20,58	28,22	37,36	31,89	23,53	27,96
**Mean**	11,24	19,58	10,74	15,17	17,09	18,36	11,96	15,07	14,59	16,54	17,01	19,34
**Std. Deviation**	4,959	9,098	4,822	6,608	2,82	3,474	5,455	9,408	7,504	7,361	3,949	4,734
**Std. Error**	1,495	2,877	3,41	1,558	1,261	1,228	1,818	4,704	1,638	2,782	1,492	1,313
**Lower 95% CI of mean**	7,904	13,07	-32,59	11,89	13,59	15,45	7,771	0,1026	11,17	9,728	13,36	16,48
**Upper 95% CI of mean**	14,57	26,09	54,07	18,46	20,59	21,26	16,16	30,04	18	23,34	20,66	22,2
**Sum**	123,6	195,8	21,48	273,1	85,44	146,9	107,7	60,29	306,3	115,8	119,1	251,5

CE 18:0

	**Controls healthy**	**Controls Bronchitis**	**Diffuse development. disorder**	**Growth abn.+ alveol. defic.**	**Immuno-intact host**	**Immuno-compromised host**	**Chronic tachypnea of infancy**	**Reactive lymphoid lesions**	**Related to alv. surf. region**	**Related to lung vessels/heart**	**Related to systemic disease**	**Unclear RDS in mature neonate**
**Number of values**	11	10	2	18	5	8	9	4	21	7	7	13
**Minimum**	2,57	1,85	3,86	1,56	1,29	1,62	1,31	1,39	0,42	1,68	0,75	1,05
**25% Percentile**	3,06	2,07		2,035	1,48	2,085	1,795	1,815	1,405	1,83	1,55	1,955
**Median**	4,19	3,63	3,995	2,315	2,02	2,59	1,97	2,33	2,53	1,86	1,98	3,28
**75% Percentile**	7,23	5,36		3,985	2,295	3,49	2,91	3,745	4,42	5,48	3,34	5,32
**Maximum**	10,17	7,75	4,13	5,42	2,4	4,18	4,2	5,07	17,8	7,42	5,35	6
**Mean**	5,118	3,861	3,995	2,783	1,914	2,766	2,373	2,78	3,691	3,163	2,56	3,569
**Std. Deviation**	2,465	1,935	0,1909	1,098	0,4395	0,8937	0,8749	1,591	3,857	2,316	1,532	1,78
**Std. Error**	0,7431	0,612	0,135	0,2587	0,1965	0,316	0,2916	0,7957	0,8417	0,8755	0,5792	0,4937
**Lower 95% CI of mean**	3,462	2,477	2,28	2,237	1,368	2,019	1,701	0,2478	1,936	1,021	1,143	2,494
**Upper 95% CI of mean**	6,774	5,245	5,71	3,329	2,46	3,513	3,046	5,312	5,447	5,305	3,977	4,645
**Sum**	56,3	38,61	7,99	50,09	9,57	22,13	21,36	11,12	77,52	22,14	17,92	46,4

CE 20:4

	**Controls healthy**	**Controls Bronchitis**	**Diffuse development. disorder**	**Growth abn.+ alveol. defic.**	**Immuno-intact host**	**Immuno-compromised host**	**Chronic tachypnea of infancy**	**Reactive lymphoid lesions**	**Related to alv. surf. region**	**Related to lung vessels/heart**	**Related to systemic disease**	**Unclear RDS in mature neonate**
**Number of values**	11	10	2	18	5	8	9	4	21	7	7	13
**Minimum**	1,14	1,08	2,42	0	4,53	3,27	0	1,29	0	1,52	3,62	1,35
**25% Percentile**	1,23	2,475		3,05	4,75	4,905	1,055	1,385	1,85	1,96	3,69	2,9
**Median**	2,13	4,875	3,935	4,37	6,23	6,65	1,7	2,495	5,09	3,44	5,39	4,32
**75% Percentile**	3,66	7,21		5,93	7,42	6,82	4,255	5,36	7,31	4,74	7,68	7,335

**Maximum**	6,48	14,36	5,45	10,37	7,5	6,91	8,69	7,21	12,44	9,37	8,1	11,05
**Mean**	2,817	5,379	3,935	4,476	6,114	5,866	2,727	3,373	4,981	4,05	5,619	5,028
**Std. Deviation**	1,756	3,836	2,143	2,56	1,347	1,356	2,657	2,749	3,636	2,618	1,916	2,912
**Std. Error**	0,5295	1,213	1,515	0,6034	0,6023	0,4794	0,8857	1,374	0,7934	0,9895	0,7242	0,8076
**Lower 95% CI of mean**	1,637	2,635	-15,31	3,202	4,442	4,733	0,6843	-1,001	3,326	1,629	3,846	3,268
**Upper 95% CI of mean**	3,997	8,123	23,18	5,749	7,786	7	4,769	7,746	6,636	6,471	7,391	6,787
**Sum**	30,99	53,79	7,87	80,56	30,57	46,93	24,54	13,49	104,6	28,35	39,33	65,36

CE 20:3

	Controls healthy	Controls Bronchitis	Diffuse development. disorder	Growth abn.+ alveol. defic.	Immuno-intact host	Immuno-compromised host	Chronic tachypnea of infancy	Reactive lymphoid lesions	Related to alv. surf. region	Related to lung vessels/heart	Related to systemic disease	Unclear RDS in mature neonate
**Number of values**	11	10	2	18	5	8	9	4	21	7	7	13
**Minimum**	0,53	0	0	0	0,69	0,89	0	1,04	0	0,55	1,03	0
**25% Percentile**	0,63	1,035		0,735	0,88	1,075	0	1,04	0,15	0,91	1,1	1,035
**Median**	0,88	1,54	0,89	1,275	1,11	1,655	0,37	1,105	1,05	1,01	1,68	1,42
**75% Percentile**	1,04	2,98		2,19	2,16	2,695	1,645	2,055	2,475	2,74	1,88	2,715
**Maximum**	1,19	6,62	1,78	5,42	2,54	3,68	3,86	2,94	5,21	4,69	6,08	4,83
**Mean**	0,8455	2,064	0,89	1,576	1,438	1,928	0,9322	1,548	1,52	1,73	2,154	1,856
**Std. Deviation**	0,2433	1,855	1,259	1,302	0,7304	1,016	1,286	0,9304	1,557	1,483	1,761	1,295
**Std. Error**	0,07335	0,5867	0,89	0,3068	0,3266	0,3594	0,4288	0,4652	0,3397	0,5605	0,6656	0,3593
**Lower 95% CI of mean**	0,682	0,7367	-10,42	0,9288	0,5311	1,078	-0,05651	0,06709	0,8118	0,3585	0,5256	1,073
**Upper 95% CI of mean**	1,009	3,391	12,2	2,223	2,345	2,777	1,921	3,028	2,229	3,101	3,783	2,639
**Sum**	9,3	20,64	1,78	28,37	7,19	15,42	8,39	6,19	31,93	12,11	15,08	24,13

CE 20:1

	Controls healthy	Controls Bronchitis	Diffuse development. disorder	Growth abn.+ alveol. defic.	Immuno-intact host	Immuno-compromised host	Chronic tachypnea of infancy	Reactive lymphoid lesions	Related to alv. surf. region	Related to lung vessels/heart	Related to systemic disease	Unclear RDS in mature neonate
**Number of values**	11	10	2	18	5	8	9	4	21	7	7	13
**Minimum**	0	0,33	1,25	0	0,23	0,14	0	0,13	0	0	0,11	0,25
**25% Percentile**	0	0,615		0,425	0,335	0,48	0,27	0,415	0,105	0,51	0,26	0,515
**Median**	0,61	0,835	1,255	0,67	0,49	0,635	0,66	0,885	0,34	0,76	0,69	0,89
**75% Percentile**	0,89	1,53		1,01	0,625	1,02	1,025	1,27	0,555	1,24	0,8	1,34
**Maximum**	1,49	1,96	1,26	1,23	0,64	1,03	1,9	1,47	1,3	1,94	1,19	1,72
**Mean**	0,6282	1,008	1,255	0,6589	0,482	0,68	0,7367	0,8425	0,4043	0,8571	0,6329	0,9246
**Std. Deviation**	0,5185	0,5179	0,007071	0,3691	0,1633	0,3247	0,5822	0,5696	0,3676	0,6215	0,3607	0,4683
**Std. Error**	0,1563	0,1638	0,005	0,087	0,07303	0,1148	0,1941	0,2848	0,08021	0,2349	0,1363	0,1299
**Lower 95% CI of mean**	0,2798	0,6375	1,191	0,4753	0,2792	0,4085	0,2892	-0,06393	0,237	0,2824	0,2993	0,6416
**Upper 95% CI of mean**	0,9765	1,378	1,319	0,8424	0,6848	0,9515	1,184	1,749	0,5716	1,432	0,9664	1,208
**Sum**	6,91	10,08	2,51	11,86	2,41	5,44	6,63	3,37	8,49	6	4,43	12,02

CE 20:0

	Controls healthy	Controls Bronchitis	Diffuse development. disorder	Growth abn. + alveol. defic.	Immuno-intact host	Immuno-compromised host	Chronic tachypnea of infancy	Reactive lymphoid lesions	Related to alv. surf. region	Related to lung vessels/heart	Related to systemic disease	Unclear RDS in mature neonate
**Number of values**	11	10	2	18	5	8	9	4	21	7	7	13
**Minimum**	0	0,34	0	0	0,29	0,34	0	0	0	0	0,2	0,32
**25% Percentile**	0	0,48		0,545	0,36	0,425	0,325	0	0,13	0	0,31	0,58
**Median**	0,48	0,625	0,315	0,785	0,46	0,49	0,47	0,18	0,55	0,37	0,48	0,8
**75% Percentile**	0,6	0,79		0,875	0,575	0,52	0,93	0,42	0,65	0,79	0,56	0,95
**Maximum**	1,11	1,05	0,63	1,44	0,68	0,62	1,08	0,48	1,94	0,95	0,68	1,05
**Mean**	0,4509	0,642	0,315	0,6917	0,466	0,4788	0,58	0,21	0,5452	0,4071	0,4471	0,7577
**Std. Deviation**	0,341	0,2047	0,4455	0,3833	0,1397	0,08357	0,3658	0,2474	0,5034	0,3665	0,1654	0,234
**Std. Error**	0,1028	0,06474	0,315	0,09033	0,0625	0,02955	0,1219	0,1237	0,1099	0,1385	0,06252	0,06489
**Lower 95% CI of mean**	0,2219	0,4956	-3,687	0,5011	0,2925	0,4089	0,2988	-0,1836	0,3161	0,06822	0,2942	0,6163
**Upper 95% CI of mean**	0,68	0,7884	4,317	0,8823	0,6395	0,5486	0,8612	0,6036	0,7744	0,7461	0,6001	0,8991
**Sum**	4,96	6,42	0,63	12,45	2,33	3,83	5,22	0,84	11,45	2,85	3,13	9,85

CE 22:6

	Controls healthy	Controls Bronchitis	Diffuse development. disorder	Growth abn. + alveol. defic.	Immuno-intact host	Immuno-compromised host	Chronic tachypnea of infancy	Reactive lymphoid lesions	Related to alv. surf. region	Related to lung vessels/heart	Related to systemic disease	Unclear RDS in mature neonate
**Number of values**	11	10	2	18	5	8	9	4	21	7	7	13
**Minimum**	0	0,26	0,68	0	0	0,22	0	0	0	0	0,12	0
**25% Percentile**	0	0,455		0,085	0,15	0,395	0	0,11	0,215	0	0,15	0,125
**Median**	0,4	0,6	0,92	0,375	0,45	0,515	0,27	0,425	0,37	0,36	0,56	0,41
**75% Percentile**	0,59	0,84		0,62	0,525	0,66	0,805	0,965	0,58	0,79	0,75	0,555
**Maximum**	0,62	1,08	1,16	0,74	0,57	0,8	1,11	1,3	0,92	1,72	0,88	1,28
**Mean**	0,3464	0,649	0,92	0,3839	0,36	0,52	0,4156	0,5375	0,4048	0,5543	0,4986	0,4431
**Std. Deviation**	0,2423	0,2416	0,3394	0,2626	0,2235	0,1878	0,411	0,5715	0,2691	0,5935	0,2875	0,4044
**Std. Error**	0,07307	0,07641	0,24	0,06189	0,09995	0,06641	0,137	0,2857	0,05873	0,2243	0,1087	0,1122

	Controls healthy	Controls Bronchitis	Diffuse development. disorder	Growth abn. + alveol. defic.	Immuno-intact host	Immuno-compromised host	Chronic tachypnea of infancy	Reactive lymphoid lesions	Related to alv. surf. region	Related to lung vessels/heart	Related to systemic disease	Unclear RDS in mature neonate
**Lower 95% CI of mean**	0,1836	0,4761	-2,129	0,2533	0,08249	0,363	0,09963	-0,3718	0,2823	0,00537	0,2327	0,1987
**Upper 95% CI of mean**	0,5092	0,8219	3,969	0,5145	0,6375	0,677	0,7315	1,447	0,5273	1,103	0,7645	0,6875
**Sum**	3,81	6,49	1,84	6,91	1,8	4,16	3,74	2,15	8,5	3,88	3,49	5,76

CE 22:5

	Controls healthy	Controls Bronchitis	Diffuse development. disorder	Growth abn. + alveol. defic.	Immuno-intact host	Immuno-compromised host	Chronic tachypnea of infancy	Reactive lymphoid lesions	Related to alv. surf. region	Related to lung vessels/heart	Related to systemic disease	Unclear RDS in mature neonate
**Number of values**	11	10	2	19	5	8	9	4	23	7	7	13
**Minimum**	0	0,26	0,68	0	0	0,22	0	0	0	0	0,12	0
**25% Percentile**	0	0,455		0,17	0,15	0,395	0	0,11	0,19	0	0,15	0,125
**Median**	0,4	0,6	0,92	0,4	0,45	0,515	0,27	0,425	0,37	0,36	0,56	0,41
**75% Percentile**	0,59	0,84		0,64	0,525	0,66	0,805	0,965	0,63	0,79	0,75	0,555
**Maximum**	0,62	1,08	1,16	1,21	0,57	0,8	1,11	1,3	0,92	1,72	0,88	1,28
**Mean**	0,3464	0,649	0,92	0,4274	0,36	0,52	0,4156	0,5375	0,4022	0,5543	0,4986	0,4431
**Std. Deviation**	0,2423	0,2416	0,3394	0,3179	0,2235	0,1878	0,411	0,5715	0,2806	0,5935	0,2875	0,4044
**Std. Error**	0,07307	0,07641	0,24	0,07292	0,09995	0,06641	0,137	0,2857	0,0585	0,2243	0,1087	0,1122
**Lower 95% CI of mean**	0,1836	0,4761	-2,129	0,2742	0,08249	0,363	0,09963	-0,3718	0,2809	0,00537	0,2327	0,1987
**Upper 95% CI of mean**	0,5092	0,8219	3,969	0,5806	0,6375	0,677	0,7315	1,447	0,5235	1,103	0,7645	0,6875
**Sum**	3,81	6,49	1,84	8,12	1,8	4,16	3,74	2,15	9,25	3,88	3,49	5,76

CE 22:4

	Controls healthy	Controls Bronchitis	Diffuse development. disorder	Growth abn. + alveol. defic.	Immuno-intact host	Immuno-compromised host	Chronic tachypnea of infancy	Reactive lymphoid lesions	Related to alv. surf. region	Related to lung vessels/heart	Related to systemic disease	Unclear RDS in mature neonate
**Number of values**	11	10	2	18	5	8	9	4	21	7	7	13
**Minimum**	0	0	0	0	0,23	0,21	0	0	0	0	0,06	0
**25% Percentile**	0	0		0,09	0,31	0,365	0	0	0,24	0	0,22	0,36
**Median**	0	0,555	0,275	0,585	0,51	0,705	0,26	0,105	0,58	0	0,71	0,6
**75% Percentile**	0,38	1,55		0,755	0,74	1,285	0,755	0,605	1,15	1,02	1,25	1,455
**Maximum**	0,55	3,83	0,55	1,49	0,96	1,4	0,87	1	2,19	1,54	1,66	1,66
**Mean**	0,1245	0,902	0,275	0,5272	0,522	0,79	0,3611	0,3025	0,7086	0,4143	0,83	0,7823
**Std. Deviation**	0,2168	1,228	0,3889	0,4525	0,2714	0,4952	0,3831	0,4754	0,6243	0,6227	0,5797	0,5918
**Std. Error**	0,06536	0,3884	0,275	0,1067	0,1214	0,1751	0,1277	0,2377	0,1362	0,2353	0,2191	0,1641
**Lower 95% CI of mean**	-0,02108	0,0233	-3,219	0,3022	0,185	0,376	0,06661	-0,454	0,4244	-0,1616	0,2939	0,4247
**Upper 95% CI of mean**	0,2702	1,781	3,769	0,7522	0,859	1,204	0,6556	1,059	0,9927	0,9901	1,366	1,14
**Sum**	1,37	9,02	0,55	9,49	2,61	6,32	3,25	1,21	14,88	2,9	5,81	10,17

CE 22:1

	**Controls healthy**	**Controls Bronchitis**	**Diffuse development. disorder**	**Growth abn. + alveol. defic.**	**Immuno-intact host**	**Immuno-compromised host**	**Chronic tachypnea of infancy**	**Reactive lymphoid lesions**	**Related to alv. surf. region**	**Related to lung vessels/heart**	**Related to systemic disease**	**Unclear RDS in mature neonate**
**Number of values**	11	10	2	18	5	8	9	4	21	7	7	13
**Minimum**	0,32	0,47	0	0	0,22	0,21	0	0	0	0	0,08	0
**25% Percentile**	0,54	0,495		0,25	0,24	0,225	0,07	0,05	0,14	0,34	0,18	0,27
**Median**	0,61	0,56	0,145	0,445	0,29	0,325	0,42	0,365	0,25	0,65	0,39	0,47
**75% Percentile**	0,7	0,85		0,665	0,49	0,455	0,64	0,795	0,605	1,15	0,59	0,77
**Maximum**	0,86	1,05	0,29	1,18	0,64	0,66	0,81	0,96	1,13	1,54	0,69	1,32
**Mean**	0,6109	0,646	0,145	0,4567	0,35	0,36	0,3856	0,4225	0,3676	0,7471	0,3686	0,5315
**Std. Deviation**	0,1495	0,2053	0,2051	0,2902	0,1679	0,1618	0,2995	0,4526	0,3153	0,5112	0,2243	0,3483
**Std. Error**	0,04507	0,06493	0,145	0,06839	0,0751	0,0572	0,09985	0,2263	0,06881	0,1932	0,08478	0,09661
**Lower 95% CI of mean**	0,5105	0,4991	-1,697	0,3124	0,1415	0,2248	0,1553	-0,2977	0,2241	0,2744	0,1611	0,321
**Upper 95% CI of mean**	0,7113	0,7929	1,987	0,601	0,5585	0,4952	0,6158	1,143	0,5112	1,22	0,576	0,742
**Sum**	6,72	6,46	0,29	8,22	1,75	2,88	3,47	1,69	7,72	5,23	2,58	6,91

Cholesteryl Ester [nmol/ml]

	Controls healthy	Controls Bronchitis	Diffuse development. disorder	Growth abn.	Immuno-intact host	Immuno-compromised host	Chronic tachypnea of infancy	Reactive lymphoid lesions	Related to alv. surf. region	Related to lung vessels/heart	Related to systemic disease	Unclear RDS in mature neonate
Number of values	11	10	2	18	5	8	9	4	19	7	7	13
Minimum	0,5	0,6	0,5	0,4	0,9	1,3	0,5	0,5	0,4	0,6	2,5	1
25% Percentile	0,6	0,65		0,8	1,5	1,6	0,7	0,5	1	0,7	2,9	1,2
Median	0,8	2,4	1,55	1,5	2,4	2,25	1,1	1,55	2,2	1,5	4,5	1,5
75% Percentile	0,9	9,35		4,25	2,9	3,5	3,25	3,6	9,6	5,6	10	3,8
Maximum	2	79	2,6	31	2,9	4,8	3,9	4,6	1179	63	11,5	10,4
Mean	0,9182	11,01	1,55	3,844	2,24	2,6	1,803	2,05	68,92	10,63	5,714	2,846
Std. Deviation	0,4665	24,13	1,485	7,141	0,8234	1,265	1,329	1,967	269,2	23,16	3,64	2,739
Std. Error	0,1407	7,631	1,05	1,683	0,3682	0,4472	0,4429	0,9836	61,76	8,752	1,376	0,7598
Lower 95% CI of mean	0,6048	-6,252	-11,79	0,2932	1,218	1,542	0,7821	-1,08	-60,83	-10,79	2,348	1,191
Upper 95% CI of mean	1,232	28,27	14,89	7,396	3,262	3,658	2,825	5,18	198,7	32,04	9,081	4,502
Sum	10,1	110,1	3,1	69,2	11,2	20,8	16,23	8,2	1309	74,4	40	37

Free Cholesterol [nmol/ml]

	Controls healthy	Controls Bronchitis	Diffuse development. disorder	Growth abn.	Immuno-intact host	Immuno-compromised host	Chronic tachypnea of infancy	Reactive lymphoid lesions	Related to alv. surf. region	Related to lung vessels/heart	Related to systemic disease	Unclear RDS in mature neonate
Number of values	11	10	2	18	5	8	9	4	21	7	7	13
Minimum	1,9	3,5	3,7	0,6	3,1	6,1	2,7	1,8	1	1,9	7,5	2,7
25% Percentile	3,4	4,4		3,05	4,95	9,6	3,9	1,9	4,6	5	7,9	4,55
Median	4,7	14,65	13,75	7,95	6,9	11,35	4,9	3,7	9,5	5,5	11,5	11,3
75% Percentile	4,9	54,8		40,6	10,2	22,15	30,35	9,45	23,55	79,2	21,5	24,4
Maximum	38,5	98	23,8	276,2	13	48,6	48,7	13,5	1050	96,5	52,4	46,7
Mean	7,209	28,07	13,75	30,11	7,44	17,61	15,76	5,675	69,1	30,87	17,81	15,84
Std. Deviation	10,46	33,56	14,21	64,23	3,551	14,08	18,54	5,472	227	39,82	16,09	14,27
Std. Error	3,153	10,61	10,05	15,14	1,588	4,976	6,181	2,736	49,54	15,05	6,08	3,958
Lower 95% CI of mean	0,183	4,063	-113,9	-1,828	3,03	5,845	1,508	-3,032	-34,24	-5,953	2,936	7,216
Upper 95% CI of mean	14,24	52,08	141,4	62,05	11,85	29,38	30,01	14,38	172,4	67,7	32,69	24,46
Sum	79,3	280,7	27,5	542	37,2	140,9	141,9	22,7	1451	216,1	124,7	205,9

All analyzed PL classes [nmol/ml]

	Controls healthy	Controls Bronchitis	Diffuse development. disorder	Growth abn.	Immuno-intact host	Immuno-compromised host	Chronic tachypnea of infancy	Reactive lymphoid lesions	Related to alv. surf. region	Related to lung vessels/heart	Related to systemic disease	Unclear RDS in mature neonate
Number of values	11	10	2	18	5	8	9	4	21	7	7	13
Minimum	13,46	21,86	13,69	1,12	14,82	21,7	27,32	15,84	2,8	12,88	22,94	6,29
25% Percentile	22,31	34		16,02	17,52	28,88	50,22	16,42	12,75	20,69	25,21	28,58
Median	28,82	50,54	46,87	48,14	20,61	50,49	97,29	22,75	23,98	37,25	38,98	53,94
75% Percentile	44,88	161		312,3	58,09	116,2	247,5	59,73	67,4	390,1	70,75	117,2
Maximum	415,2	614,6	80,04	1492	66,24	189,6	375,8	90,95	2973	1474	126,7	282,4
Mean	65,4	127,8	46,87	212,4	34,36	75,32	140,7	38,07	196,2	290,8	54,9	84,24
Std. Deviation	116,7	180,2	46,92	382,3	22,53	61,23	135,7	35,71	642,1	538,5	36,59	89,29
Std. Error	35,19	56,99	33,18	90,1	10,07	21,65	45,24	17,86	140,1	203,5	13,83	24,76
Lower 95% CI of mean	-13,01	-1,144	-374,7	22,32	6,395	24,12	36,4	-18,76	-96,07	-207,3	21,07	30,29
Upper 95% CI of mean	143,8	256,7	468,4	402,5	62,33	126,5	245	94,9	488,5	788,8	88,74	138,2
Sum	719,4	1278	93,73	3824	171,8	602,5	1266	152,3	4121	2035	384,3	1095

All analyzed lipids [nmol/ml]

	Controls healthy	Controls Bronchitis	Diffuse development. disorder	Growth abn.	Immuno-intact host	Immuno-compromised host	Chronic tachypnea of infancy	Reactive lymphoid lesions	Related to alv. surf. region	Related to lung vessels/heart	Related to systemic disease	Unclear RDS in mature neonate
Number of values	11	10	2	18	5	8	9	4	21	7	7	13
Minimum	16,19	26,3	17,86	2,07	18,75	33,63	31,04	18,13	4,09	15,36	39,03	10,44
25% Percentile	26,47	38,91		21,03	23,92	39,24	58,32	18,82	24,95	27,21	43,92	35,1
Median	34,33	74	62,17	59,09	30,44	70,71	102,9	29	36,95	61,37	54,64	65,34
75% Percentile	50,25	250,2		365,6	70,88	133,9	281,6	72,79	98,37	532,2	102,2	147,3
Maximum	455,7	723,6	106,5	1800	82,1	243,1	427,4	107,1	5202	1576	181,5	314,3
Mean	73,51	166,9	62,17	246,4	44	95,54	158,3	45,81	331	332,3	78,44	102,9
Std. Deviation	127,5	219,3	62,66	450,3	26,18	72,92	154,8	41,9	1123	578,2	50,55	103,4
Std. Error	38,44	69,36	44,31	106,1	11,71	25,78	51,61	20,95	245	218,5	19,11	28,68
Lower 95% CI of mean	-12,15	9,966	-500,8	22,45	11,5	34,58	39,28	-20,88	-180,1	-202,5	31,69	40,42
Upper 95% CI of mean	159,2	323,8	625,1	470,3	76,51	156,5	277,3	112,5	842,1	867	125,2	165,4
Sum	808,6	1669	124,3	4435	220	764,3	1425	183,2	6951	2326	549,1	1338

All analyzed PL [%of tot. Lip.]

	Controls healthy	Controls Bronchitis	Diffuse development. disorder	Growth abn.	Immuno-intact host	Immuno-compromised host	Chronic tachypnea of infancy	Reactive lymphoid lesions	Related to alv. surf. region	Related to lung vessels/heart	Related to systemic disease	Unclear RDS in mature neonate
Number of values	11	10	2	18	5	8	9	4	21	7	7	13
Minimum	79,3	29,7	75,2	54	67,7	8,8	81,4	74,1	9,3	60,7	57,4	60,3
25% Percentile	84	50,25		79,7	68,6	58,75	87,55	79,5	52,5	73,3	58,8	69,55
Median	86,1	68,55	75,95	85,05	79	71,25	88,1	86	61	83,7	69,2	81,4
75% Percentile	88,7	73,75		89,05	82,2	85,95	93,35	87,25	81,4	91,4	78,8	84,95
Maximum	91,1	78	76,7	92,5	83,7	87,6	94,6	87,4	100	93,5	82,3	89,9
Mean	85,95	62,72	75,95	81,56	76,12	66,04	89,57	83,38	65	80,36	68,7	77,68
Std. Deviation	3,3	15,67	1,061	10,87	7,097	26,26	4,231	6,283	20,39	11,35	9,407	9,97
Std. Error	0,9949	4,956	0,75	2,561	3,174	9,284	1,41	3,141	4,45	4,292	3,555	2,765
Lower 95% CI of mean	83,74	51,51	66,42	76,15	67,31	44,08	86,31	73,38	55,72	69,86	60	71,66
Upper 95% CI of mean	88,17	73,93	85,48	86,96	84,93	87,99	92,82	93,37	74,29	90,86	77,4	83,71
Sum	945,5	627,2	151,9	1468	380,6	528,3	806,1	333,5	1365	562,5	480,9	1010

Free Cholesterol [% of tot.Lip.]

	Controls healthy	Controls Bronchitis	Diffuse development. disorder	Growth abn.	Immuno-intact host	Immuno-compromised host	Chronic tachypnea of infancy	Reactive lymphoid lesions	Related to alv. surf. region	Related to lung vessels/heart	Related to systemic disease	Unclear RDS in mature neonate
Number of values	11	10	2	18	5	8	9	4	21	7	7	13
Minimum	8,4	10	20,5	6,97	12,3	10,7	4,6	9,7	7,25	6,1	10,3	8,2
25% Percentile	10	11,7		8,975	14,05	11,65	5,45	10,05	11,78	7,7	14,4	12,1
Median	11,1	16,3	21,45	12,92	16,3	17,2	9,9	11,5	22,38	12,8	21	16,4
75% Percentile	13,2	21,05		16,79	23,05	29,85	11,2	13,25	36,33	18,3	29,6	24,2
Maximum	15,5	38,7	22,4	32,25	23,3	41,1	16,17	13,9	52,69	36,9	36	32,8
Mean	11,62	17,89	21,45	14,47	18,1	21,15	8,941	11,65	23,98	15,6	22,19	18,13
Std. Deviation	2,167	8,355	1,344	7,024	4,778	11,25	3,795	1,943	13,87	10,26	9,501	7,677
Std. Error	0,6535	2,642	0,95	1,656	2,137	3,979	1,265	0,9717	3,026	3,878	3,591	2,129
Lower 95% CI of mean	10,16	11,91	9,379	10,98	12,17	11,74	6,024	8,558	17,67	6,111	13,4	13,49
Upper 95% CI of mean	13,07	23,87	33,52	17,96	24,03	30,56	11,86	14,74	30,3	25,09	30,97	22,77
Sum	127,8	178,9	42,9	260,5	90,5	169,2	80,47	46,6	503,6	109,2	155,3	235,7
